# A silylboronate-mediated strategy for cross-coupling of alkyl fluorides with aryl alkanes: mechanistic insights and scope expansion†

**DOI:** 10.1039/d4sc04357j

**Published:** 2024-10-01

**Authors:** Jun Zhou, Zhengyu Zhao, Tatsuki Kiyono, Ayaka Matsuno, Jorge Escorihuela, Norio Shibata

**Affiliations:** a Department of Nanopharmaceutical Sciences, Nagoya Institute of Technology Gokiso, Showa-ku Nagoya 466-8555 Japan nozshiba@nitech.ac.jp; b Department of Life Science and Applied Chemistry, Nagoya Institute of Technology Gokiso, Showa-ku Nagoya 466-8555 Japan; c Departamento de Química Orgánica, Universitat de València Avda. Vicente Andrés Estellés s/n, Burjassot 46100 Valencia Spain

## Abstract

The construction of C(sp^3^)–C(sp^3^) bonds is pivotal in organic synthesis; however, traditional methods involving alkyl halides are often limited by substrate tolerance and bond dissociation energies, particularly with alkyl fluorides. Herein, we report a silylboronate-mediated cross-coupling strategy that circumvents these challenges, enabling the efficient formation of C(sp^3^)–C(sp^3^) bonds between alkyl fluorides and aryl alkanes under mild conditions. Various alkyl fluorides have also been effectively utilized, demonstrating the versatility and broad applicability of this approach. The use of diglyme is critical for this transformation which encapsulates potassium cations and enhances the reaction efficiency. Conventional alkyl halides, including chlorides, bromides, and iodides, are also suitable for this transformation. Density functional theory (DFT) calculations were conducted on the silylboronate-mediated coupling reactions for the first time. Interestingly, while experimental results suggest a radical mechanism, DFT calculations indicate a preference for an ionic pathway.

## Abstract 2

The silylboronate-mediated cross-coupling strategy enabled efficient formation of C(sp^3^)–C(sp^3^) bonds between alkyl fluorides and aryl alkanes *via* C–F bond cleavage under mild conditions.

## Graphical abstract

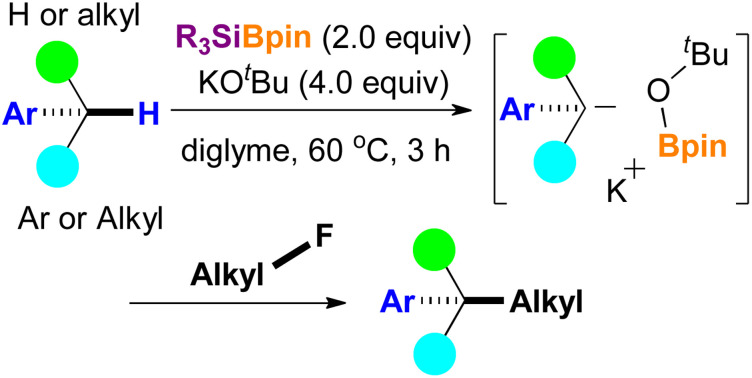

## Introduction

**Fig. 1:**
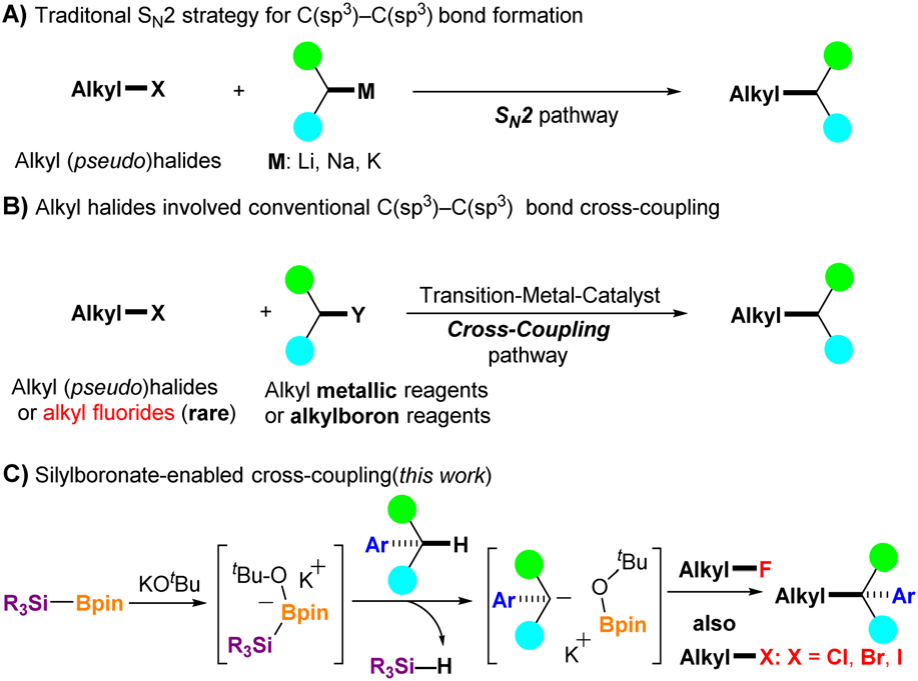
C(sp^3^)–C(sp^3^) bond formation using alkyl halides and their equivalents. (A) Alkali metal-mediated S_N_2-strategy. (B) TM-catalyzed cross-coupling. (C) Silylboronate-enabled cross-coupling (this work).

The construction of C(sp^3^)–C(sp^3^) bonds is a fundamental tool in organic synthetic chemistry.^1^ Nucleophilic C(sp^3^)–C(sp^3^) bond formation of alkali metal compounds with alkyl halides or their equivalents through the S_N_2 process is one of the most straightforward strategies used for this purpose (Fig. 1A).^2^ However, the use of a strong base is often avoided because of the poor functional tolerance of substrates. In addition, the only acceptable alkyl halides are bromo-, iodo-, and chloro-derivatives, but alkyl fluorides are not suitable because of the high bond dissociation energy of the carbon–fluorine (C–F) bond (up to 130 kcal mol^−1^).^3^ In this context, transition metal (TM)-catalyzed C(sp^3^)–C(sp^3^) bond formation under mild conditions has emerged as an attractive alternative. TM-catalyzed cross-coupling between an electrophilic alkyl halide and a nucleophilic organometallic reagent (*e.g.*, *R*-MgX, *R*-ZnX, or *R*-B(OR′)_2_) has progressed significantly over the past decades,^4^ but several limitations and the need for preprepared organometallic reagents are nonnegligible issues in this strategy (Fig. 1B). Moreover, alkyl fluorides were rarely used as electrophilic coupling partners until 2003.^5^ Kambe *et al.* reported a CuCl_2_-catalyzed cross-coupling reaction of primary alkyl fluorides with alkyl Grignard reagents, which efficiently provided C(sp^3^)–C(sp^3^) bond coupling products.^5*a*^ Nonetheless, new methods that avoid the use of transition metal catalysts for constructing C(sp^3^)–C(sp^3^) bonds are increasingly needed, especially in the pharmaceutical industry.^6^

The functionalization of benzylic C(sp^3^)–H in aryl alkanes has received considerable attention in the last decades because many bioactive compounds, including top-selling drugs such as Jardiance, Lynparza, and Advair, contain benzylic moieties.^7^ In addition, C(sp^3^)–H in aryl alkanes can be further functionalized to form new C–C, C–N, C–O, and C–F bonds, which can serve as essential synthons for further synthetic transformations.^8^ Recently, our group reported that *in situ* generated silyl radicals successfully enabled the cross-coupling of alkyl fluorides with styrenes and aryl allenes *via* the activation of inert C–F bonds, with a series of corresponding coupling products obtained in a single step under mild conditions.^9^ When the cross-coupling partners were changed to aryl alkanes, aryl fluorides were also effective; however, alkyl fluorides did not function well enough.^10^ As a continuation of our studies on the C–F bond activation project, we herein report a silylboronate-mediated cross-coupling reaction of alkyl fluorides 1 with aryl alkanes 2 under mild conditions *via* the cleavage of C(sp^3^)–F and C(sp^3^)–H bonds, which produces diaryl alkanes or aryl alkanes 3 with a tertiary or quaternary carbon center (Fig. 1C). Notably, this coupling reaction proceeded smoothly without the need for transition-metal catalysis, providing a series of coupling products in moderate to excellent yields. The scope of the coupling partners was sufficiently broad, and not only diaryl alkanes but also mono-aryl alkanes were effective for the coupling reaction with alkyl fluorides. In addition, site-selective coupling of diaryl alkanes with alkyl fluorides was achieved even in the presence of a mono-aryl alkane moiety in the alkyl fluorides. Moreover, not only alkyl fluorides but also alkyl halides (alkyl-X, X = Cl, Br, and I) provided the desired cross-coupling products in high yields using this reaction system. Our investigations have shown that the use of diglyme as a solvent is critical for successful transformation by encapsulation of potassium cations. Control experiments indicated that the reaction initially proceeds *via* a radical pathway, followed by a nucleophilic reaction. Interestingly, density functional theory (DFT) calculations suggest that an ionic process could also be favorable in the initial step. The reaction mechanism is discussed in further detail.

## Results and discussion

### Silylboronate-mediated cross-coupling reactions of alkyl fluorides and aryl alkanes

Initially, 1-fluorooctane (1a) and diphenylmethane (2b) were chosen as model substrates to investigate the silylboronate-mediated cross-coupling reaction and optimize the reaction conditions (Table 1). Based on the previous results,^9^ we attempted a model reaction in the presence of Et_3_SiBpin (2.0 equiv.), KO^*t*^Bu (4.0 equiv.), and diglyme at room temperature (20 °C) for 12 h. The reaction proceeded as expected, but only the desired defluoroalkylation product, nonane-1,1-diyldibenzene (3ab), was produced in 10% yield, leaving an 87% yield of 1a (entry 1). Subsequently, the reaction temperature was varied (entries 2–5). By gradually increasing the reaction temperature from 30 °C to 60 °C, we obtained a maximum yield of 97% when the reaction was performed at 60 °C (entry 5). Control experiments revealed that no reaction occurred in the absence of KO^*t*^Bu or Et_3_SiBpin (entries 6 and 7, respectively). However, the yield of 3ab decreased to 66% when the amount of 2b was reduced to 1.5 equiv. (entry 8). Remarkably, defluoroalkylation proceeded efficiently, affording the desired cross-coupling product 3ab in up to 97% yield (90% isolated) within 3 h (entries 9 and 10). Finally, we examined these reactions using three different silylboronates (PhMe_2_SiBpin, ^*t*^BuMe_2_SiBpin, and (Me_3_Si)_3_SiBpin). Interestingly, the yield gradually decreased as the steric hindrance of the silylboronate increased (entries 11–13). The use of diglyme is critical and other solvents such as *c*-hexane and THF were not effective for this conversion (14 and 15). Further details on the optimization of the reaction conditions are provided in the ESI (see Tables S1–S4).†

**Table 1:** Optimization of the defluoroalkylation reaction conditions^a^

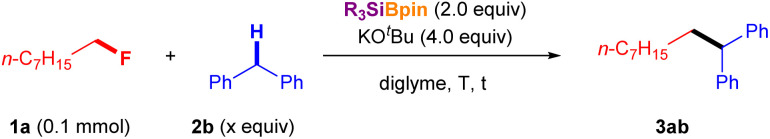				
Entry	2b (*x* equiv.)	*T* (°C)	*t* (h)	3ab^b^ (%)
1	2.0	rt (20)	12	10
2	2.0	30	12	34
3	2.0	40	12	56
4	2.0	50	12	79
5	2.0	60	12	97
6^c^	2.0	60	12	0
7^d^	2.0	60	12	0
8	1.5	60	12	66
9	2.0	60	5	98
10	2.0	60	3	97 (90)
11^e^	2.0	60	3	72
12^f^	2.0	60	3	48
13^g^	2.0	60	3	16
14^h^	2.0	60	3	15
15^i^	2.0	60	3	26

a Unless otherwise noted, reactions were conducted using 1a (27.2 mg, 0.1 mmol), 2b, KO^*t*^Bu (44.8 mg, 0.4 mmol), Et_3_SiBpin (48.4 mg, 0.2 mmol), and diglyme (1.0 mL) and allowed to react at the indicated temperatures for the indicated hours.

b Determined by ^19^F and ^1^H NMR spectroscopy using 3-fluoropyridine (8.6 μL, 0.1 mmol) as internal standard.

c Without Et_3_SiBpin.

d Without KO^*t*^Bu.

e ^*t*^BuMe_2_SiBpin was used.

f PhMe_2_SiBpin was used.

g TMS_3_SiBpin was used. The numbers in parentheses refer to isolated yields.

h *c*-Hexane was used instead of diglyme.

i THF was used instead of diglyme.

### Scope and limitations

**Fig. 2:**
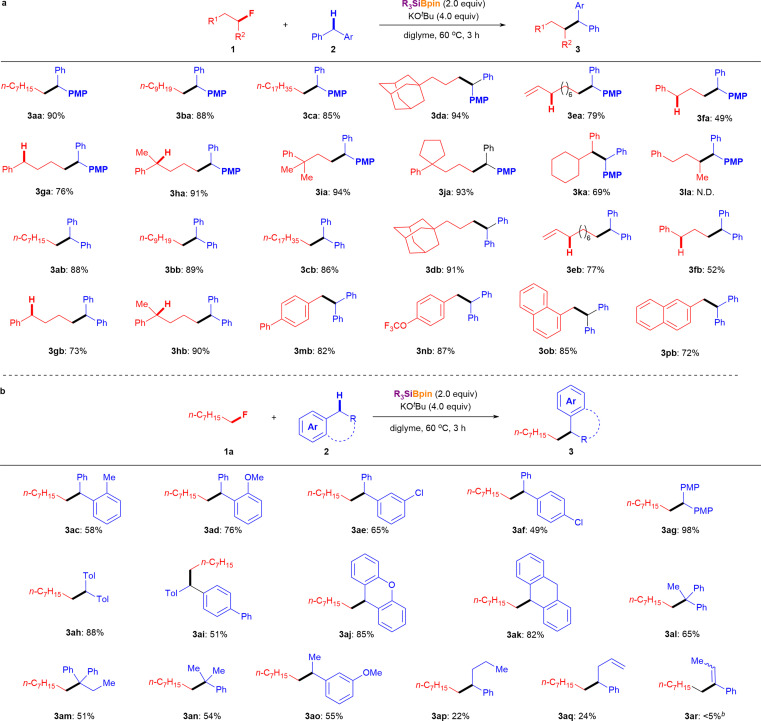
Substrate scope of alkyl fluorides. ^*a*^Unless otherwise noted, reactions were conducted using 1 (0.2 mmol, 1.0 equiv.), 2 (2.0 equiv.), Et_3_SiBpin (96.8 mg, 2.0 equiv.), KO^*t*^Bu (89.6 mg, 4.0 equiv.), and diglyme (2.0 mL) at 60 °C for 3 h. The isolated yields are shown. *^b^*Determined by ^1^H NMR spectroscopy using 3-fluoropyridine as an internal standard.

Having established the optimized reaction conditions for the defluoroalkylation of alkyl fluorides (entry 10, Table 1), we next evaluated the silylboronate-mediated defluorinative cross-coupling reactions using a range of alkyl fluorides 1 with diphenylmethanes (2a or 2b). As shown in Fig. 2a, primary alkyl fluorides with different carbon lengths (C8:1a; C10:1b; C18:1c) were efficiently coupled with *p*-benzylanisole (2a) under the optimized conditions, affording the corresponding cross-coupling products 3 in good-to-excellent yields (3aa: 90%; 3ba: 88%; 3ca: 85%). Similarly, a primary alkyl fluoride substituted with an adamantyl group (1d) afforded desired coupling product 3da in 94% yield. Interestingly, primary alkyl fluorides containing vinyl (1e) or phenyl (1f) groups, which feature active allylic or benzylic C–H bonds, successfully yielded the corresponding diphenylalkanes 3 in moderate to good yields under standard conditions (3ea: 79%; 3fa: 49%). Additionally, comparative experiments clearly showed the influence of the benzylic C–H bond on this coupling reaction using fluoro-aryl alkanes without substituents (1g) and with one or two substituents (1h, 1j) at the benzyl position. The corresponding cross-coupling products (3ga, 76%; 3ha, 91%; 3ia, 94%; 3ja, 93%) were obtained in high yields under the optimized conditions in the presence of 2a. In addition, secondary benzyl fluoride 1k reacted with 2a under standard conditions to provide the desired cross-coupling product 3ka in 69% yield. However, the more challenging secondary alkyl fluoride 1l, which bears benzylic protons, failed to undergo coupling with 2a under the same conditions. Furthermore, a series of primary alkyl fluorides (1a–1h) was chosen for the silylboronate-mediated defluorinative cross-coupling reaction. As expected, similar results were obtained when coupling with 2b afforded the corresponding coupling products (3ab: 88%, 3bb: 89%, 3cb: 86%, 3db: 91%, 3eb: 77%, 3fb: 52%, 3gb: 73%, 3hb: 90%) in up to 91% yield. It should be emphasized again that site-selective coupling of 2b was observed with a series of alkyl fluorides 1 with allylic and benzylic C–H (1e–1p).

The substrate scope of aryl alkane 2, which has different electronic and steric properties, was explored using 1a under the standard conditions (Fig. 2b). Initially, sterically hindered diphenylmethanes (2c and 2d) were evaluated with 1a, which afforded the corresponding cross-coupling products 3ac and 3ad in 58% and 76% yields, respectively. Notably, Cl-substituted diphenylmethanes were tolerated under the reaction conditions (3ae, 65%; 3af, 49%). Furthermore, diphenylmethanes bearing electron-donating groups (2g–2i) reacted smoothly and efficiently with 1a under standard conditions to furnish the desired diaryl alkanes in moderate to good yields (3ag: 98%; 3ah: 88%; 3ai: 51%). 9*H*-Xanthene (2j) and dihydroanthracene (DHA, 2k) could also serve as cross-coupling partners,^10,11^ giving the desired cross-coupling products in good yields (3aj: 85%; 3ak: 82%) through this cross-coupling process with alkyl fluoride 1a under standard conditions.

To further confirm the scope and limitations of aryl alkane 2 using this methodology, the cross-coupling of a variety of aryl alkanes possessing one or two aryl rings with 1a was attempted under the optimal reaction conditions. Remarkably, products featuring a quaternary carbon center were obtained in moderate yields (3al: 65%; 3am: 51%) when tertiary carbon-centered aryl alkanes (2l and 2m) were used. While diarylalkane-selective coupling was observed (Fig. 2, 3ea, 3fa, 3ga, 3ha, 3ka, 3eb, 3fb, 3gb, 3hb, 3mb, 3nb, 3ob, and 3pb), mono-aryl alkanes were also effective in cross-coupling reactions with alkyl fluoride 1a (3an: 54%; 3ao: 55%; 3ap: 22%; 3aq: 24%). When we attempted the reaction of allylbenzene (2r), a similar result was obtained after isomerization (3ar: <5% ^1^H NMR yield) instead of the expected coupling product.

### Mechanistic studies

**Fig. 3:**
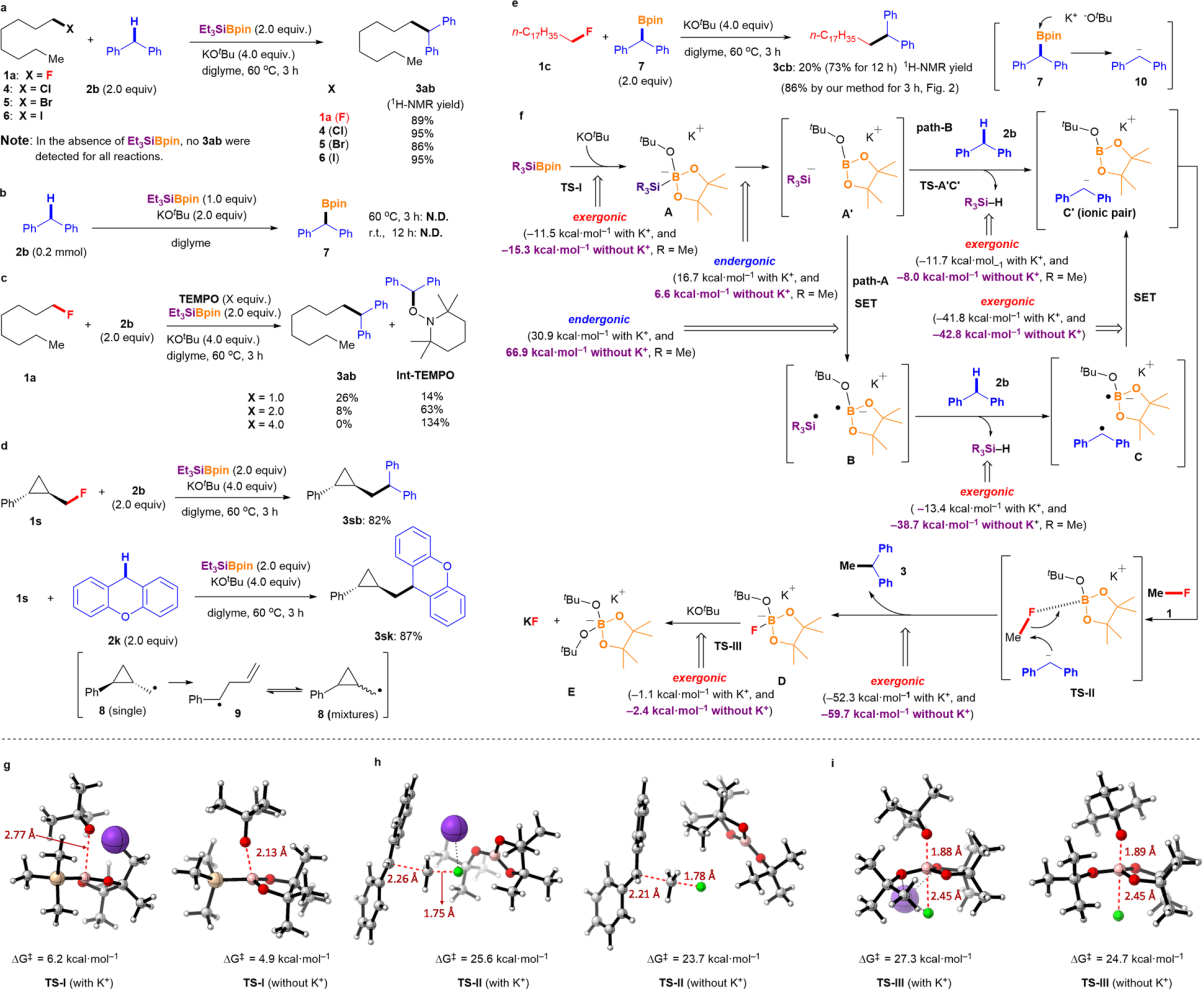
Mechanistic studies. (a) Cross-coupling reactions using alkyl halides (R–X; X = F, Cl, Br, and I). (b) Experiments for the generation of 7. (c) Effect of TEMPO on the silylboronate-mediated coupling reaction. (d) Radical ring-opening experiments. (e) Comparative study of S_N_2 reaction. (f) Proposed mechanistic pathway based on DFT calculations (Gibbs energy values at 298.15 K are given). Me_3_SiBpin was used instead of Et_3_SiBpin, and methyl fluoride (Me–F) was used as alkyl fluoride 1. (g–i) DFT calculations for TS-I, TS-II, TS-III with and without K^+^. Without K^+^ indicates the reaction in diglyme.

To further elucidate the cross-coupling reaction mechanism between the alkyl fluorides and aryl alkanes, several control experiments were conducted (Fig. 3). It should be mentioned that our cross-coupling reaction is effective not only for alkyl fluorides but also for other alkyl halides. The cross-coupling reactions of diphenylmethane (2b) with 1-chlorooctane (4), 1-bromooctane (5), and 1-iodooctane (6) afforded the desired cross-coupling products 3ab in excellent yields under the optimized conditions (Fig. 3a). To determine whether the nucleophilic pathway is involved in these parallel reactions, several of the aforementioned reactions were repeated under identical conditions without using Et_3_SiBpin. Under these conditions, no cross-coupling product 3ab was detected. Therefore, it can be concluded that this reaction did not proceed *via* the conventional S_N_2 pathway from 2b and KO^*t*^Bu. Ohmiya *et al.* reported cross-coupling of aryl fluorides with tertiary benzylic organoboranes driven by the basicity of KO^*t*^Bu at high temperature (120 °C) *via* S_N_Ar.^12^ Therefore, we conducted several control experiments to further verify the reaction mechanism (Fig. 3b). After the treatment of 2b with the Et_3_SiBpin/KO^*t*^Bu/diglyme system at 60 °C for 3 h or at room temperature for 12 h, the expected Ph_2_CHBpin 7 was not obtained. Thus, the formation of benzylic organoborane 7 from 2b was excluded under these reaction conditions. Next, we examined the coupling reaction between 1-fluorooctane 1a and 2b in the presence of (2,2,6,6-tetramethylpiperidin-1-yl)oxyl (TEMPO) (Fig. 3c). While coupling product 3ab was obtained in 89% yield under standard conditions, the yields decreased dramatically from 26% to 0% as the amount of this radical scavenger increased from 1.0 equivalent to 4.0 equivalent, respectively. The ^1^H NMR yields of 1-(benzhydryloxy)-2,2,6,6-tetramethylpiperidine (Int-TEMPO)^10^ ranged from 14% to 63% and 134%, respectively. The reactions of conventional alkyl halides 4, 5, and 6 were also evaluated in the presence of TEMPO (2.0 equivalent). As expected, Int-TEMPO was detected in <10% ^1^H NMR yield with no desired cross-coupling products (see ESI† for details). In addition, an electron spin resonance (ESR) experiment showed that a benzyl-type radical was detected in a previous study.^10^ Therefore, it can be concluded that the cross-coupling reaction involves the generation of radical species along the reaction mechanism.

Radical ring-opening experiments were performed to provide further insight into the reaction process (Fig. 3d). The reactions of *trans*-(2-(fluoromethyl)cyclopropyl)benzene (1s) with 2b and 2k were evaluated under standard conditions, but neither reaction afforded the corresponding ring-opening products. Instead, *trans*-cyclopropyl groups were retained in the coupling products (3sb: 82%; 3sk: 87%). This observation strongly indicates that the cyclopropyl methyl radical 8 (and its ring-opening radical 9) is not involved in the final step of C(sp^3^)–F bond cleavage; thus, the C(sp^3^)–F bond is not radically cleaved. Therefore, the final step of C(sp^3^)–F bond cleavage may proceed *via* an ionic process with the assistance of the interaction with the B atom of BPin.

Besides, we attempted the nucleophilic reaction of 1c with Ph_2_CHBpin 7 under the similar conditions (in the presence of KO^*t*^Bu in diglyme at 60 °C for 3 h) for comparison (Fig. 3e). Although this process should generate Ph_2_CH anion 10, the desired 3cb was obtained in 20% ^1^H-NMR yield. Although the yield was much lower than that obtained using our protocol (86% isolated yield, see Fig. 2), it increased to 73% ^1^H-NMR yield after 12 h. This observation could be explained by the slow generation of the diphenyl methyl anion 10 from 7 (see ESI† for more comparative examples of the S_N_2 reaction). These results strongly support the proposed mechanism involving the nucleophilic pathway in the final step while acknowledging the potential coexistence of radical and ionic processes. Based on our experimental results and those of previous reports,^9,10^ a mechanism involving “a sterically demanding radical pair”-mediated nucleophilic defluorinative cross-coupling reaction was hypothesized (Fig. 3f).

### DFT study

The mechanism underlying the silylboronate-mediated cross-coupling with organic fluorides remains a subject of ongoing debate.^9,10^ Because of its sterically demanding ionic structure, which exhibits radical-like behavior, we have proposed that the reaction proceeds through a sterically demanding radical pair. Our experimental findings, combined with previous reports, suggest a newly hypothesized mechanism involving a sterically demanding radical pair mediating nucleophilic defluorinative cross-coupling reactions (Fig. 3f). To further elucidate the cross-coupling mechanism, DFT calculations were performed using the Gaussian 16 package^13^ at the wB97XD/def2TZVP level of theory using the SMD solvation model.^14^ Trimethylsilyl-Bpin (Me_3_SiBpin) was used instead of Et_3_SiBpin for computational simplicity to reduce the computational cost. As expected, the reaction of silylborane with KO^*t*^Bu to form intermediate A is highly exergonic (−11.5 kcal mol^−1^) and occurs *via* a transition state I (TS-I) with a low activation barrier (6.2 kcal mol^−1^) (Fig. 3g). In the presence of diglyme, the potassium cation is encapsulated,^15^ resulting in the generation of naked ^*t*^BuO^−^ anions, which favors the generation of intermediate A as the reaction becomes more exergonic (−15.3 kcal mol^−1^) and proceeds *via* a TS-I with lower activation barrier than in the absence of diglyme (4.9 kcal mol^−1^) (Fig. 3g). This outcome was in good agreement with the experimental results for the diglyme effect. Next, sterically demanding intermediate A can split into an ionic pair A′. While the process is an endergonic (16.7 and 6.6 kcal mol^−1^, in the absence or the presence of diglyme, respectively), the values are reasonable for the reaction proceeding (path-A). Then, intermediate A splits into the sterically demanding radical pair B, which comprises the trimethylsilyl radical (˙SiMe_3_) and a boron-radical species (B˙), *via* the single electron transfer (SET). This process was found to be endergonic and not favored (30.9 and 66.9 kcal mol^−1^, in the absence or presence of diglyme, respectively). However, hydrogen atom abstraction from diphenylmethane 2b by ˙SiMe_3_ (detected by ESR^10^) in B yields a sterically demanding radical pair consisting of a diphenylmethyl radical (detected by ESR^10^) and boron-radical species, accompanied by the formation of HSiMe_3_ (HSiEt_3_ detected by GC). The formation of diphenylmethyl radical C was found to be exergonic in the absence of diglyme (−13.4 kcal mol^−1^) and highly exergonic in the presence of diglyme (−38.7 kcal mol^−1^). Subsequently, the sterically demanding radical pair C is spontaneously converted to an ion pair C′ due to the exergonic process (in the absence of diglyme, −41.8 kcal mol^−1^; in the presence of diglyme, −42.8 kcal mol^−1^). The sterically demanding ionic pair C′ attracts organic fluoride 1 (Me–F was used for simplicity) in the ionic cage *via* the interaction between the fluorine (F) atom and boron (B) center to afford TS-II. Since the C–F bond of 1 is activated by the interaction of the B atom in TS-II, the diphenylmethyl anion in the sterically demanding ion pair C′ would attack the carbon center of the C–F bond, as shown in TS-II. This reaction process was confirmed to be highly exergonic (−52.3 and −59.7 kcal mol^−1^, in the absence or the presence of diglyme, respectively). The diphenylmethyl anion in TS-II is naked because of the encapsulation of K^+^ by diglyme and the sterically demanding structure of Bpin(O^*t*^Bu). Finally, the desired ethane-1,1-diyldibenzene 3 is obtained *via* nucleophilic C–C bond formation, accompanied by the release of D ([Bpin(O^*t*^Bu)F]K).

In the absence of diglyme, a transition state with a barrier of 25.6 kcal mol^−1^ and a C⋯C bond forming distance of 2.26 Å was located. This transition state is assisted by the potassium cation that interacts with the F atom (K⋯F distance of 2.64 Å). On the other hand, in the presence of diglyme, the potassium cation is encapsulated, making it inaccessible, which is reflected in a later transition state with a barrier of 23.7 kcal mol^−1^ and a C⋯C bond forming distance of 2.21 Å (Fig. 3h). Finally, the reaction of D with an excess amount of KO^*t*^Bu provides stable ([Bpin(O^*t*^Bu)_2_]K) species E (detected using ^11^B NMR spectroscopy) and KF (detected using ^19^F NMR spectroscopy). This reaction proceeds *via* a TS-III with activation barriers of 27.3 and 24.7 kcal mol^−1^ in the absence or the presence of diglyme, respectively (Fig. 3i). The formation of E and KF is exergonic (−1.1 and −2.4 kcal mol^−1^, in the absence or the presence of diglyme, respectively); however, thermodynamic driving force is clearly favored in the presence of diglyme (Fig. 3f).

**Fig. 4:**
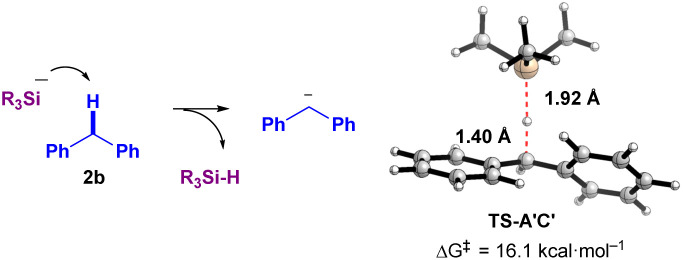
A transition state TS-A′C′ from A′ to C′ (deprotonation of 2b by the SiR_3_ anion (R = Me) (without K^+^)).

However, the reaction mechanism *via*pathway-A involves a highly endergonic process from A′ to B (30.9 and 66.9 kcal mol^−1^ in the absence and presence of diglyme, respectively), as the SET process is not favorable. Therefore, we considered a direct process from A′ to C′ (pathway-B). Notably, this process is exergonic (−11.7 and −8.0 kcal mol^−1^ in the absence and presence of diglyme, respectively). Thus, based on the results of DFT calculations, pathway-B appears to be more plausible. Specifically, the formation of the ionic pair C′ from A′ occurs *via* the deprotonation of diphenylmethane (2b) by the naked SiR_3_ anion (^−^SiR_3_, R = Me), proceeding through the transition state TS-A′C′. This transition state has an activation Gibbs energy barrier of 16.1 kcal mol^−1^, and C⋯H and Si⋯H distances of 1.40 Å and 1.92 Å, respectively, which supports the feasibility of the proposed ionic mechanism (Fig. 4). However, experimental observations (Fig. 3b and c, ESR detection of Si radical and diphenylethylene radical^10^) support pathway-A. These conflicting results suggest the possibility of a mechanism that incorporates both radical and ionic pathways, or alternatively, a secondary pathway that generates the sterically hindered radical pair B. Another explanation is that TEMPO could react with intermediates A or A′, forming TEMPO-SiR_3_ or TEMPO-Bpin(O^*t*^Bu)^−^, which would inhibit the reaction. Additionally, any intermediate radical may react with a benzylic group to form a benzyl radical, which could then be trapped by TEMPO to generate Int-TEMPO, a species that has been experimentally observed.

## Conclusions

In summary, we have developed a feasible silylboronate-mediated cross-coupling reaction of alkyl fluorides with aryl alkanes. Various benzylic C(sp^3^)–H bond alkylated products were efficiently synthesized in moderate-to-excellent yields under mild conditions. We believe that silylboronate-mediated coupling of diverse alkyl halides, including alkyl fluorides, chlorides, bromides, and iodides, with benzylic C–H bonds could be a valuable method for organic synthesis. Mechanistic studies using density functional theory calculations were conducted on the silylboronate-mediated coupling reactions for the first time. The crucial role of diglyme in this cross-coupling transformation was supported by DFT calculations. Further studies are currently in progress to elucidate the proposed reaction mechanism.

## Author contributions

JZ optimized the reaction conditions. JZ, ZZ, TK, and AM surveyed the substrate scope, analyzed the data, and discussed the results with NS. JZ, ZZ, TK, and AM prepared starting materials. JE performed the DFT calculations and analyzed the data. JZ and NS wrote the manuscript. NS supervised the study. All the authors contributed to and approved the final version of the manuscript.

## Conflicts of interest

There are no conflicts to declare.

## Supplementary Material

SC-015-D4SC04357J-s001

## Data Availability

The data that support the findings of this study are available within the article and the ESI.† Details about materials and methods, experimental procedures, characterization data, and NMR spectral are included.
