# Supplementary material for: A silylboronate-mediated strategy for cross-coupling of alkyl fluorides with aryl alkanes: mechanistic insights and scope expansion
Source: Chem Sci. 2024 Oct 1;15(42):17418–24. doi: 10.1039/d4sc04357j (PMC11446385; doi:10.1039/d4sc04357j)

Supplementary Information (SI) for Chemical Science.
This journal is © The Royal Society of Chemistry 2024

Electronic Supplementary Information

A silylboronate-mediated strategy for cross-coupling of alkyl fluorides with aryl alkanes: mechanistic insights and scope expansion

Jun Zhou,[a] Zhengyu Zhao,[a] Tatsuki Kiyono,[b] Ayaka Matsuno,[b] Jorge Escorihuela,[c] and Norio Shibata*[a,b]

[a]*Department of Nanopharmaceutical Sciences, Nagoya Institute of Technology, Gokiso, Showa-ku, Nagoya 466-8555, Japan.*
[b]*Department of Life Science and Applied Chemistry, Nagoya Institute of Technology, Gokiso, Showa-ku, Nagoya 466-8555, Japan.*
[c]*Departamento de Química Orgánica, Universitat de València, Avda. Vicente Andrés Estellés s/n, Burjassot 46100, Valencia, Spain.*

*Correspondence and requests for materials should be addressed to N.S. (email: *nozshiba@nitech.ac.jp*)

Table of Contents

1. General information and materials 3

2. Supplementary experimental procedures for the synthesis of starting materials. 4

2.1 Synthesis of substituted alkyl fluorides **1** 4

2.2 Synthesis of arylalkanes **2**........ 10

3. Supplementary details for condition optimization 13

4. Supplementary experimental procedures and spectral data of products........ 15

4.1. General procedure for the optimization of the cross-coupling of alkyl fluoride with diphenylmethane........ 15

4.2. General procedure for the optimization of the cross-coupling of alkyl fluoride with arylalkanes 15

4.3. Characterization data of products 16

5. Mechanistic control experiments 34

5.1. Cross-coupling reactions using alkyl halides........ 34

5.2. Reaction with radical scavenger TEMPO........ 36

5.3. S_N2 comparative experiments 38

6. Computational details........ 39

7. References 78

8. Supplementary NMR spectra (^{1}H NMR, ^{13}C NMR and ^{19}F NMR)........ 80

1. General information and materials

All reactions were performed in oven-dried glassware under a positive pressure of nitrogen or argon. Solvents were transferred via syringe and were introduced into the reaction vessels through a rubber septum. All the reactions were monitored by thin-layer chromatography (TLC) carried out on 0.25 mm Merck silica gel (60-F254). The TLC plates were visualized with UV light. All the reaction products were purified by column chromatography and was carried out on a column packed with silica gel 60N spherical neutral size 50-63 mm. The ^{1}H NMR (300 MHz), ^{13}C NMR (75 MHz) and ^{19}F NMR (282 MHz) spectra as for solution in $CDCl_3$ were recorded on a Varian Mercury 300. The ^{1}H NMR (500 MHz) and ^{13}C NMR (126 MHz) spectra as for solution in $CDCl_3$ were recorded on a BRUKER 500 Ultra Shield TR. The ^{1}H NMR (700 MHz) and ^{13}C NMR (176 MHz) spectra as for solution in $CDCl_3$ were recorded on a JEOL RESONANCE ECZ700R. The chemical shifts (δ) are expressed in ppm downfield from internal TMS (δ = 0.00) and coupling constants (*J*) are reported in hertz (Hz). The hexafluorobenzene (C_6F_6) [δ = −162.2 ($CDCl_3$)] was used as internal standard for ^{19}F NMR. The following abbreviations were used to explain the multiplicities: s = singlet, d = doublet, t = triplet, q = quartet, m = multiplet, br = broad. Mass spectra were recorded on a JEOL JMS-Q1050GC (EI-MS) and/or SHIMADZU LCMS-2020 (ESI-MS). High resolution mass spectrometry (HRMS) was carried out on an electron impact ionization mass spectrometer with a micro-TOF analyzer and recorded on a Waters, GCT Premier (EI-MS) with a TOF analyzer. Infrared spectra were recorded on a JASCO FT/IR-4100 spectrometer. Melting points were recorded on a BUCHI M-565.

Commercially available chemicals were obtained from Aldrich Chemical Co., Alfa Aesar, TCI and used as received unless otherwise noted. Silylboranates were prepared according to the known procedures. Solvent DCM, Dioxane, and THF were dried and distilled before use.

2. Supplementary experimental procedures for the synthesis of starting materials.

2.1 Synthesis of substituted alkyl fluorides 1

Alkylfluorides **1a** were purchased from Sigma Aldrich. **1b**, **1d**, **1e**, **1f**, **1g**, **1k**, **1n**, **1o**, **1p** and **1q** were prepared according to known methods.[1] A typical experimental procedure for the preparation of **1c**, **1h**, **1i**, **1j**, **1l**, and **1s** were described below.

1-Fluorooctadecane (1c)

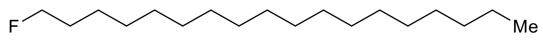

To the solution of DAST (1.7 mL, 13 mmol) in the DCM (10 mL) was added a solution of 1-octadecanol (2.7 g, 10 mmol) in DCM (20 mL) dropwise at –40 °C. Then the mixture was moved to room temperature and stirred for 6 h. After that, the reaction mixture was poured into cooled water, neutralized with saturated $NaHCO_3$, and then extracted with DCM. The combined organic phase was dried over Na_2SO_4, filtered, and evaporated under reduced pressure. The crude was purified by column chromatography on silica gel (*n*-hexane) to give title compound as colorless oil (1.8 g, 66%).

^{1}H NMR (300 MHz, $CDCl_3$) δ 4.44 (dt, *J* = 47.4, 6.2 Hz, 2H), 1.79 – 1.57 (m, 2H), 1.45 – 1.16 (m, 30H), 0.88 (t, *J* = 6.4 Hz, 3H).

^{19}F NMR (282 MHz, $CDCl_3$) δ -218.47 (tt, *J* = 47.8, 24.8 Hz, 1F).

MS(EI): *m/z* 272 $[M]^+$. The chemical shifts were consistent with those reported in the literature.[2]

(5-Fluoropentan-2-yl)benzene (1h)

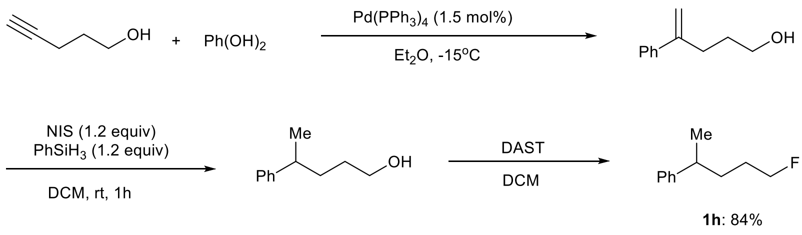

Under a nitrogen atmosphere pent-4-yn-1-ol (1.65 g, 20.0 mmol), $Pd(PPh_3)_4$ (0.35 g, 0.3 mmol) and the boronic acid (3.04 g, 24.0 mmol) were charged into a dry flask. Dry 1,4-dioxane (60 mL) and AcOH (0.22 mL) were added, and the solution was stirred for 15 min at room temperature. Then the flask was heated to 80 °C and stirred for 24 h. The mixture was cooled down to room temperature

and 1,4-dioxane was removed in vacuo. Purification by flash chromatography (DCM) afforded 4-phenylpent-4-en-1-ol (1.7 g, 52%).[3]

A solution of NIS (1.08 g, 4.8 mmol) and $PhSiH_3$ (0.52 g, 4.8 mmol) in DCM (8 mL) was stirred for 30 min at room temperature, a solution of 4-phenylpent-4-en-1-ol (0.65 g, 4.0 mmol) in DCM (4 mL) was added at room temperature. The reaction mixture was stirred for 1 h, and then was quenched with H_2O and extracted with Et_2O. The combined organic layer was washed with brine, dried over Na_2SO_4, filtered, and evaporated under reduced pressure. The crude was purified by column chromatography on silica gel (*n*-hexane/Et_2O = 5/1) to give 4-phenylpentan-1-ol as colorless oil (367 mg, 56%).[4]

To the solution of DAST (0.28 mL, 2.15 mmol) in DCM (1 mL) was added a solution of 4-phenylpentan-1-ol (0.66 g, 4.0 mmol) in DCM (1 mL) dropwise at –40 ^oC. Then the mixture was moved to room temperature and stirred for 6 h. After that, the reaction mixture was poured into cooled water, neutralized with saturated $NaHCO_3$, and then extracted with DCM. The combined organic phase was dried over Na_2SO_4, filtered, and evaporated under reduced pressure. The crude was purified by column chromatography on silica gel (*n*-hexane) to give title compound as colorless oil (200 mg, 84%).

^{1}H NMR (300 MHz, $CDCl_3$) δ 7.36 – 7.28 (m, 2H), 7.24 – 7.17 (m, 3H), 4.69 – 4.18 (m, 2H), 2.74 (h, *J* = 7.0 Hz, 1H), 1.86 – 1.46 (m, 4H), 1.29 (d, *J* = 6.9 Hz, 3H).

^{13}C NMR (75 MHz, $CDCl_3$) δ 147.1, 128.5, 127.1, 126.2, 84.3 (d, *J* = 164.3 Hz), 39.8, 33.9 (d, *J* = 4.8 Hz), 28.8 (d, *J* = 19.6 Hz), 22.5.

^{19}F NMR (282 MHz, $CDCl_3$) δ -218.68 (tt, *J* = 47.1, 24.5 Hz, 1F).

IR (KBr): 3062, 3029, 2963, 2902, 2873, 1604, 1495, 1453, 1389, 1061, 1048, 998, 985, 894, 762, 701 cm^{-1}.

HRMS (EI) [$C_{11}H_{15}F$] $[M]^+$ calculated: 166.1158, found: 166.1170.

(4-Fluoro-2-methylbutan-2-yl)benzene (1i)

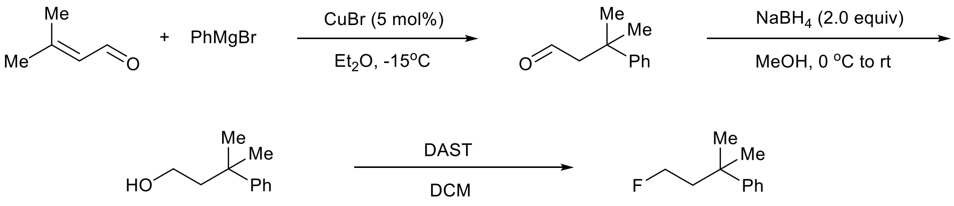

1i: 75%

Under a nitrogen atmosphere, a stirred solution of phenylmagnesium bromide (6.7 mL, 20.0 mmol, 3.0 M in diethyl ether) in 20 mL of tetrahydrofuran is cooled to –15 ºC and copper(I) bromide (140 mg, 1.0 mmol) was added in one portion. Then 3-methyl-2-butenal (2.0 mL, 20.0 mmol) of was added dropwise over 0.5 h. Upon completion of addition, the reaction mixture was allowed to warm to room temperature and keep stirring for 18 h. The reaction mixture is then poured into 50 mL of ice water and extracted with diethyl ether. The combined organic phase was dried over Na_2SO_4, filtered, and evaporated under reduced pressure. The crude was purified by column chromatography on silica gel (*n*-hexane/DCM = 1/1) to give 3-methyl-3-phenylbutanal as a colorless oil (2.0 g, 62%).

To the solution of 3-methyl-3-phenylbutanal (1.0 g, 6.0 mmol) in MeOH (10 mL) in 0 ºC was added $NaBH_4$ (0.50 g, 12 mmol), and then keep stirring for 1 h at room temperature. After completion, the mixture was diluted by water (10 mL), extracted by Et_2O, washed by brine and dried over Na_2SO_4. Then the filtrate was concentrated under reduced pressure. The crude was purified by column chromatography on silica gel (*n*-hexane /EtOAc = 4/1) to give 3-methyl-3-phenylbutan-1-ol as a colorless oil (0.95 g, 96%).

To the solution of DAST (0.66 mL, 5.0 mmol) in DCM (5.0 mL) was added a solution of 3-methyl-3-phenylbutan-1-ol (0.66 g, 4.0 mmol) in DCM (4.0 mL) dropwise at –40 ºC. Then the mixture was moved to room temperature and stirred for 6 h. After that, the reaction mixture was poured into cooled water, neutralized with saturated $NaHCO_3$, and then extracted with DCM. The combined organic phase was dried over Na_2SO_4, filtered, and evaporated under reduced pressure. The crude was purified by column chromatography on silica gel (*n*-hexane) to give title compound as colorless oil (0.49 g, 75%).

^{1}H NMR (300 MHz, $CDCl_3$) δ 7.46 – 7.12 (m, 5H), 4.30 (dt, *J* = 47.2, 6.7 Hz, 2H), 2.07 (dt, *J* = 22.5, 6.7 Hz, 2H), 1.37 (s, 6H).

^{13}C NMR (75 MHz, $CDCl_3$) δ 148.4, 128.4, 126.0, 125.8, 82.2 (d, J = 163.1 Hz), 44.2 (d, J = 18.0 Hz), 36.7 (d, J = 5.9 Hz), 29.4.

^{19}F NMR (282 MHz, $CDCl_3$) δ -218.11 (tt, J = 46.6, 22.4 Hz, 1F).

IR (KBr): 3060, 3026, 2968, 2910, 2878, 1603, 1497, 1474, 1444, 1389, 1368, 1063, 1008, 984, 764, 700 cm^{-1}.

HRMS (EI) [$C_{11}H_{15}F$] $[M]^+$ calculated: 166.1158, found: 166.1164.

(1-(3-Fluoropropyl)cyclopropyl)benzene (1j)

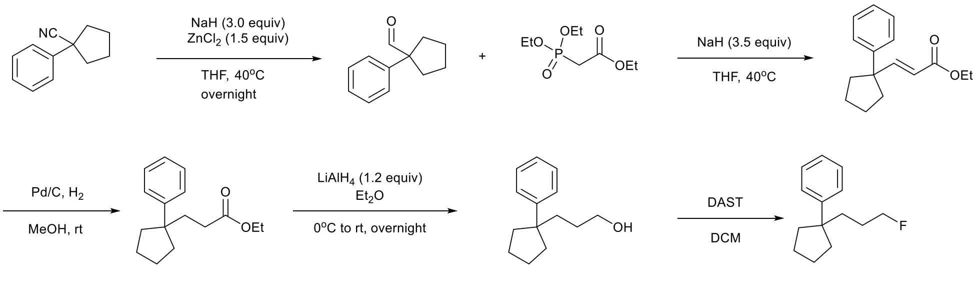

To a flame dried flask were added 1-phenylcyclopentane-1-carbonitrile (2.67 g, 15.6 mmol), NaH (1.87 g, 46.8 mmol, 60% in mineral oil), $ZnCl_2$ (3.20 g, 23.4 mmol) and THF (80 mL). Seal and stir the mixture at 40 °C overnight. After that quench the reaction with saturated ammonium chloride at 0 °C. Extracted with CH_2Cl_2, and dry the combined organic extracts over Na_2SO_4, filtered and evaporated under reduced pressure. The crude was purified by column chromatography on silica gel (*n*-hexane/EtOAc = 20/1) to give 1-phenylcyclopentane-1-carbaldehyde as colorless oil (2.45 g, 91%).[5]

To a flame dried flask, triethylphosphonoacetate (7.88 g, 35.0 mmol) was added to a solution of NaH (1.40 g, 35.0 mmol, 60% in mineral oil) in THF under 0 °C, and the mixture was keep stirring for 30 min. A solution of 1-phenylcyclopentane-1-carbaldehyde (1.74 g, 10.0 mmol) in THF was added dropwise and then stirred at room temperature for 16 h. After the reaction finished ice water was added, and the mixture was extracted with EtOAc. The organic layer was combined and washed with brine, then dried over Na_2SO_4. Filtered and evaporated under reduced pressure. The crude was purified by column chromatography on silica gel (*n*-hexane/EtOAc = 20/1) to give ethyl-3-(1-phenylcyclopentyl)acrylate as colorless oil (1.83 g, 75%).

Ethyl-3-(1-phenylcyclopentyl)acrylate (1.71 g, 7.0 mmol) was dissolved in methanol (50 mL),

then Pd/C (74 mg) was added. The mixture was stirred under hydrogen atmosphere at room temperature for 3 h. After the catalyst was removed, filtrate was evaporated in vacuo to obtain ethyl 3-(1-phenylcyclopentyl)propanoate (1.52 g, 88%) as a colorless oil.

$LiAlH_4$ (230 mg, 6.0 mmol) was added slowly to the solution of ethyl 3-(1-phenylcyclopentyl)propanoate (1.23, 5.0 mmol) in Et_2O (20 mL) at 0 °C and then the solution was stirred for 2 h at room temperature. After that, a solution of NaOH (10% in water) was added carefully until a white solid precipitated. After filtration over Celite® and evaporation of the solvent to obtain crude 3-(1-phenylcyclopentyl)propan-1-ol (0.97 g, 95%) as a colorless oil.

To the solution of DAST (0.75 mL, 5.6 mmol) in DCM (4 mL) was added a solution of 3-(1-phenylcyclopentyl)propan-1-ol (0.97 g, 4.7 mmol) in DCM (5 mL) dropwise at –40 °C. Then the mixture was moved to room temperature and stirred for 6 h. After that, the reaction mixture was poured into cooled water, neutralized with saturated $NaHCO_3$, and then extracted with DCM. The combined organic phase was dried over Na_2SO_4, filtered, and evaporated under reduced pressure. The crude was purified by column chromatography on silica gel (*n*-hexane) to give title compound as colorless oil (0.66 g, 68%).

^{1}H NMR (300 MHz, $CDCl_3$) δ 7.31 – 7.23 (m, 4H), 7.25 – 7.10 (m, 1H), 4.26 (dt, *J* = 47.4, 6.1 Hz, 2H), 2.04 – 1.77 (m, 5H), 1.76 – 1.58 (m, 5H), 1.50 – 1.25 (m, 2H).

^{13}C NMR (75 MHz, $CDCl_3$) δ 148.3, 128.1, 126.9, 125.6, 84.7 (d, *J* = 164.4 Hz), 50.7, 37.7, 37.3 (d, *J* = 4.6 Hz), 26.7 (d, *J* = 19.6 Hz), 23.3.

^{19}F NMR (282 MHz, $CDCl_3$) δ -217.96 (tt, *J* = 47.6, 24.9 Hz, 1F).

IR (KBr): 3059, 3030, 2957, 2915, 2873, 1496, 1478, 1456, 1445, 1336, 1197, 1047, 991, 767, 701 cm^{-1}.

HRMS (EI) [$C_{14}H_{19}F$] $[M]^+$ calculated: 206.1471, found: 206.1480.

(3-Fluorobutyl)benzene (1l)

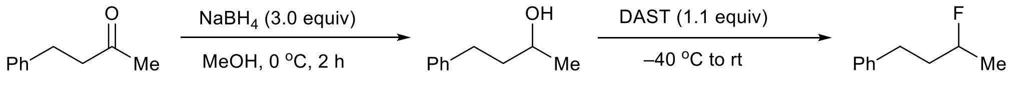

1l: 43%

To the solution of 4-phenylbutan-2-one (1.48 g, 10.0 mmol) in MeOH (30 mL) in 0 °C was added $NaBH_4$ (1.14 g, 30 mmol), and then keep stirring for 2 h at room temperature. After

completion, the mixture was diluted by water (10 mL), extracted by Et_2O, washed by brine and dried over Na_2SO_4. Then the filtrate was concentrated under reduced pressure. The crude was purified by column chromatography on silica gel (*n*-hexane /EtOAc = 4/1) to give 3-methyl-3-phenylbutan-1-ol as a colorless oil (1.36 g, 91%).

To the solution of DAST (1.2 mL, 8.8 mmol) in DCM (10.0 mL) was added a solution of 4-phenylbutan-2-ol (1.20 g, 8.0 mmol) in DCM (5.0 mL) dropwise at –40 °C. Then the mixture was moved to room temperature and stirred for 6 h. After that, the reaction mixture was poured into cooled water, neutralized with saturated $NaHCO_3$, and then extracted with DCM. The combined organic phase was dried over Na_2SO_4, filtered, and evaporated under reduced pressure. The crude was purified by column chromatography on silica gel (*n*-hexane) to give title compound as colorless oil (0.52 g, 43%).

^{1}H NMR (300 MHz, $CDCl_3$) δ 7.42 – 7.06 (m, 5H), 4.97 – 4.38 (m, 1H), 3.02 – 2.51 (m, 2H), 2.17 – 1.65 (m, 2H), 1.47 – 1.20 (m, 3H).

^{19}F NMR (282 MHz, $CDCl_3$) δ -173.55 – -176.04 (m, 1F).

MS(EI): *m/z* 152 $[M]^+$. The chemical shifts were consistent with those reported in the literature.[6]

(2-(Fluoromethyl)cyclopropyl)benzene (1s)

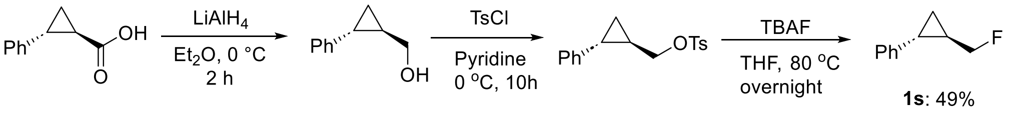

The title product **1s** was synthesized according to the known procedures with modifies.[7]

Add a solution of *trans*-2-phenylcyclopropane-1-carboxylic acid (1.62 g, 10 mmol) in dry diethyl ether (20 mL) to a solution of $LiAlH_4$ (790 mg, 20 mmol) in dry diethyl ether (10 mL) at room temperature, the reaction mixture was stirred for 6 h. Quench the reaction mixture by dropwise addition of H_2O at 0°C, extract the mixture with diethyl ether for 3 times. The combined organic phase was dried over Na_2SO_4, filtered, and evaporated under reduced pressure. The crude was purified by column chromatography on silica gel (*n*-hexane/EtOAc = 3/1) to give the corresponding alcohol as colorless oil (1.35 g, 90%).

trans-(2-Phenylcyclopropyl)methanol (1.35 g, 9.0 mmol) was added to a solution of *p*-toluenesulfonyl chloride (1.72 g, 9.0 mmol), DMAP (108 mg, 0.9 mmol), Et_3N (1.6 mL, 11.7 mmol) in 30 mL of DCM and was stirred for 2 h at room temperature. After check the TLC, the mixture

was added onto a crushed ice and extracted with diethyl ether, dried over Na_2SO_4 and the solvent evaporated in vaccuo to give (2-phenylcyclopropyl)methyl 4-methylbenzenesulfonate and used for the next step without further purification.

In a glovebox, a flame dried flask was charged with (2-phenylcyclopropyl)methyl 4-methylbenzenesulfonate and tetrabutylammonium fluoride (TBAF, 27 mL, 1 M in THF). Then the flask was capped with a rubber septum before moving out from glovebox. The mixture was then heated at 80 °C overnight. After that, the reaction mixture extracted with DCM. The combined organic phase was washed with brine and dried over Na_2SO_4, evaporated under reduced pressure. The crude was purified by column chromatography on silica gel (*n*-hexane) to give title compound as a colorless oil (0.66 g, 49%).

^{1}H NMR (500 MHz, $CDCl_3$) δ **7.29 (t, *J* = 7.6 Hz, 2H), 7.20 (t, *J* = 7.4 Hz, 1H), 7.11 (dd, *J* = 8.1, 1.4 Hz, 2H)**, 4.41 (dd, *J* = 48.5, 7.0 Hz, 2H), **1.95 (dt, *J* = 9.5, 4.9 Hz, 1H), 1.58 (dddd, *J* = 11.2, 8.5, 5.7, 1.3 Hz, 1H)**, 1.08 (dtd, *J* = 8.7, 5.4, 3.5 Hz, 1H), 1.03 (dtd, *J* = 9.0, 5.4, 1.0 Hz, 1H).

^{19}F NMR (282 MHz, $CDCl_3$) δ -210.69 (t, *J* = 48.6 Hz, 1F).

MS(EI): *m/z* 150 $[M]^+$. The chemical shifts were consistent with those reported in the literature.[7]

2.2 Synthesis of arylalkanes 2.

Diphenylmethane (**2b**), 1,1-diphenylethane (**2j)**, Xanthene (**2l)**, 9,10-dihydroanthracene (**2m)**, cumene (**2n)**, and butylbenzene (**2p)**, 4-phenyl-1-butene (**2q)**, allylbenzene (**2r)** were purchased from TCI or Sigma Aldrich. Diarylmethanes **2a**, **2c**, **2f**, **2g**, **2h**, **2k** and **2t** were prepared according to the previous work.[8] A typical known experimental procedure for the preparation of diarylmethanes **2d**, **2e**, **2i**, **2o**, **2s** and **2u** were described below.

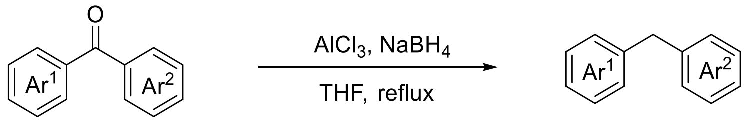

General procedure A: The diarylmethanes was synthesized according to the known procedures with modifies.[9] A dried flask was charged with benzophenones, sodium borohydride, and anhydrous aluminum chloride in anhydrous THF. The mixture was stirred under reflux for 2 h and the reaction progress was monitored by TLC. After the ketones fully consumed, the mixture was cooled to room temperature. To this mixture was slowly added water 10 mL, and then extracted

with EtOAc. The combined organic layer was washed with brine, dried over Na_2SO_4. After filtration, the filtrate was concentrated under reduced pressure to give the crude, which was purified by column chromatography on silica gel (*n*-hexane) to give corresponding diarylmethanes **2**.

1-Benzyl-2-methoxybenzene (2d)

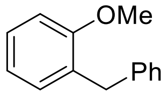

According to general procedure **A**, 2-methoxybenzophenone (0.87 g, 4.11 mmol), sodium borohydride (0.78 g, 20.6 mmol), anhydrous aluminum chloride (1.64 g, 12.3 mmol), and anhydrous THF (20 mL) were used. The title product **2d** was isolated as a white solid after flash chromatography (0.41 g, 50%).

^{1}H NMR (300 MHz, $CDCl_3$) δ 7.35 – 7.12 (m, 6H), 7.11 – 6.99 (m, 1H), 6.95 – 6.78 (m, 2H), 3.98 (s, 2H), 3.82 (s, 3H).

MS(EI): *m/z* 198 $[M]^+$. The chemical shifts were consistent with those reported in the literature.[10]

1-Benzyl-3-chlorobenzene (2e)

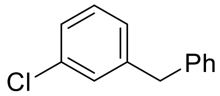

According to general procedure **A**, 3-chlorobenzophenone (2.16 g, 10 mmol), sodium borohydride (1.90 g, 50 mmol), anhydrous aluminum chloride (4.0 g, 30 mmol), and anhydrous THF (50 mL) were used. The title product **2e** was isolated as a white solid after flash chromatography (0.75 g, 37%).

^{1}H NMR (300 MHz, $CDCl_3$) δ 7.50 – 6.95 (m, 7H), 3.95 (s, 1H).

MS(EI): *m/z* 202 $[M]^+$. The chemical shifts were consistent with those reported in the literature.[10]

2-Benzylfuran (2u)

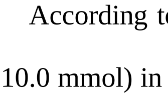

According to a known procedure: Under an argon atmosphere, to a solution of furan (730 µL, 10.0 mmol) in anhydrous THF (10 mL) at 0 ºC was added a solution of *n*-BuLi (4.0 mL, 1.6 M in hexane, 5.0 mmol). Then the mixture was keeping stirring for 20 min at the same temperature followed by the slow addition of benzyl bromide (593 µL, 5.0 mmol) solution in anhydrous THF (2 mL). The reaction mixture was then warmed to rt and stirred for 12 h. The reaction mixture was

then quenched with H_2O slowly and extracted with Et_2O. The combined organic phase was washed with brine, dried over Na_2SO_4, filtered, and evaporated under reduced pressure. The crude was purified by column chromatography on silica gel (*n*-hexane) to give title compound **2u** as a colorless oil (504 mg, 63%).

^{1}H NMR (300 MHz, $CDCl_3$) δ 7.36 – 7.26 (m, 3H), 7.26 – 7.19 (m, 3H), 6.29 (dd, *J* = 3.2, 1.9 Hz, 1H), 6.00 (dd, *J* = 3.2, 0.9 Hz, 1H), 3.97 (s, 2H).

MS(EI): *m/z* 158 $[M]^+$. The chemical shifts were consistent with those reported in the literature.[11]

4-(4-Methylbenzyl)-biphenyl (2i)

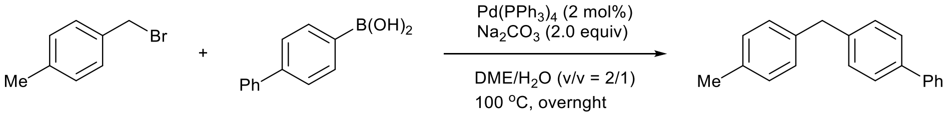

2i: 65%

According to a known procedure: In an oven-dried flask, 4-methylbenzyl bromide (0.925 g, 5.0 mmol) and 4-biphenylboronic acid (1.18 g, 6.0 mmol), $Pd(PPh_3)_4$ (116 mg, 0.1 mmol), Na_2CO_3 (1.06 g, 10.0 mmol) were charged sequentially, then a mixture of DME/H_2O (15 mL, v/v = 2/1) was added. The resulting mixture was allowed to stir at 100 °C overnight. The reaction mixture is then extracted with diethyl ether and water. The combined organic phase was dried over Na_2SO_4, filtered, and evaporated under reduced pressure. The crude was purified by column chromatography on silica gel (*n*-hexane) to give title compound **2i** as a white solid (0.84 g, 65%).

^{1}H NMR (500 MHz, $CDCl_3$) δ 7.58 – 7.54 (m, 2H), 7.52 – 7.48 (m, 2H), 7.41 (t, *J* = 7.3 Hz, 2H), 7.31 (t, *J* = 6.9 Hz, 1H), 7.28 – 7.21 (m, 2H), 7.11 (s, 4H), 3.98 (s, 2H), 2.32 (s, 3H).

MS(EI): *m/z* 258 $[M]^+$. The chemical shifts were consistent with those reported in the literature.[12]

1-Ethyl-3-methoxybenzene (2o)

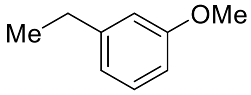

In an oven-dried flask, to a solution of 3-ethyl-phenol (0.61 g, 5.0 mmol) in dry DMF (5 mL) was added NaH (0.3 g, 12.5 mmol), the resulting mixture was allowed to stir for 1 hour at room temperature. Then methyl iodide (1.0 mL, 15.0 mmol) was added. The reaction mixture was stirred at room temperature overnight. The reaction mixture was quenched with ice water slowly, extracted with Et_2O, and the combined organic layer were washed with brine, dried over Na_2SO_4, filtered, and

evaporated under reduced pressure. The crude was purified by column chromatography on silica gel (*n*-hexane/EtOAc: 20/1) to give title compound **2o** as a colorless oil (0.59 g, 86%).

^{1}H NMR (300 MHz, $CDCl_3$) δ 7.19 (t, *J* = 7.8 Hz, 1H), 6.88 – 6.45 (m, 3H), 3.78 (s, 3H), 2.62 (q, *J* = 7.6 Hz, 2H), 1.23 (t, *J* = 7.6 Hz, 3H).

MS(EI): *m/z* 136 $[M]^+$. The chemical shifts were consistent with those reported in the literature.[13]

3. Supplementary details for condition optimization

Table S1 Reaction temperature screening[a]

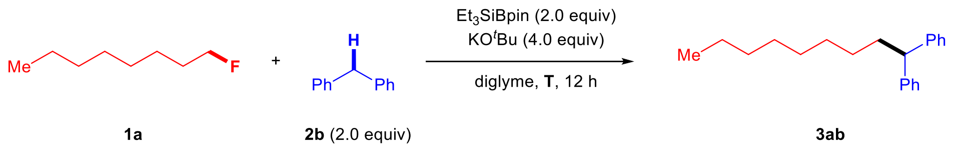

Entry	T(°C)	3ab (%)[b]
1	rt (20 °C)	10
2	30	34
3	40	56
4	50	79
5	60	97
6[c]	60	N.D.
7[d]	60	N.D.

[a]Reactions conditions: unless otherwise noted, the reactions were conducted with **1a** (16.5 µL, 0.1 mmol), **2b** (33.6 mg, 2.0 equiv), $Et_3SiBpin$ (48.4 mg, 2.0 equiv), and KO*t*Bu (44.8 mg, 4.0 equiv) in diglyme (1.0 mL) react for 12 hours under the indicated temperature. [b]Determined by ^{19}F NMR and ^1H NMR spectroscopy using 3-fluoropyridine as an internal standard, [c]Without $Et_3SiBpin$. [d]Without KO*t*Bu.

Table S2 Reaction equivalent screening[a]

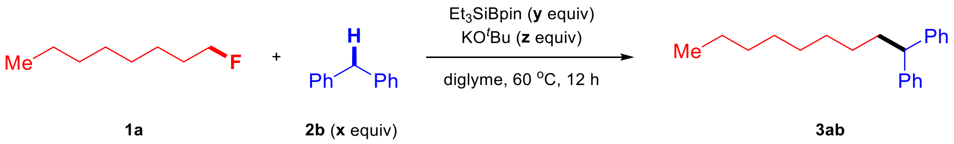

Entry	x	y	z	3ab (%)[b]
1	1.5	2.0	4.0	66
2	1.0	2.0	4.0	28
3	2.0	1.0	2.0	42
4	2.0	0	4.0	N.D.
5	2.0	2.0	0	N.D.

[a]Reactions conditions: **1a** (16.5 μL, 0.1 mmol), **2b**, $Et_3SiBpin$, and KO^{t}Bu in diglyme (1.0 mL) react under 60 °C for 12 hours. [b]Determined by ^{19}F NMR and ^1H NMR spectroscopy using 3-fluoropyridine as an internal standard.

Table S3 Reaction time optimization[a]

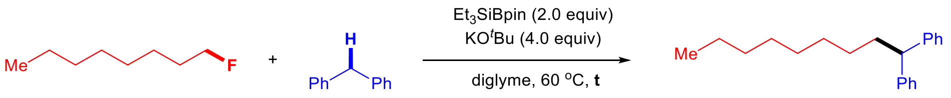

1a **2b** (2.0 equiv) **3ab**

Entry	t(h)	3ab (%)[b]
1	12	97
2	5	98
3	3	97 (90)
4	2	77

[a]Reactions conditions: **1a** (16.5 μL, 0.1 mmol), **2b** (33.6 mg, 2.0 equiv), $Et_3SiBpin$ (48.4 mg, 2.0 equiv), and KO^{t}Bu (44.8 mg, 4.0 equiv) in diglyme (1.0 mL) react under 60 °C for the indicated hours. [b]Determined by ^{19}F NMR and ^1H NMR spectroscopy using 3-fluoropyridine as an internal standard, the number in parentheses refer to isolated yield.

Table S4 Optimization of silylboronate[a]

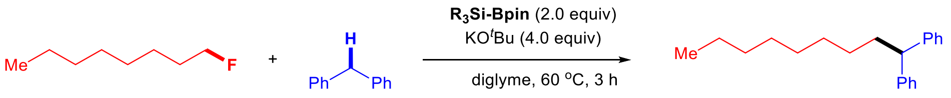

1a **2b** (2.0 equiv) **3ab**

Entry	R3SiBpin	3ab (%)[b]
1	^{t}BuMe$_2$SiBpin	72
2	$PhMe_2SiBpin$	48
3	$TMS_3SiBpin$	16
4	$Et_3SiBpin$	96

[a]Reactions conditions: **1a** (16.5 μL, 0.1 mmol), **2b** (33.6 mg, 2.0 equiv), $R_3SiBpin$ (2.0 equiv), and KO^{t}Bu (44.8 mg, 4.0 equiv) in diglyme (1.0 mL) react under 60 °C for 3 hours. [b]Determined by ^{19}F NMR and ^1H NMR spectroscopy using 3-fluoropyridine as an internal standard, the number in parentheses refer to isolated yield.

4. Supplementary experimental procedures and spectral data of products

4.1. General procedure for the optimization of the cross-coupling of alkyl fluoride with diphenylmethane (General Procedure B)

In a N_2 filled glovebox, to a flame-dried screw-capped test tube was added 1-fluorooctane **1a** (16.5 µL, 0.1 mmol, 1.0 equiv), silylboronates, diphenylmethane **2b**, KO^{t}Bu and diglyme (1.0 mL) sequentially. The tube then was sealed and removed from the glovebox. The solution was stirred at indicated temperature and hours. The reaction tube was quenched with saturated NH_4Cl (5 mL), then extracted with Et_2O, washed with large amount water and brine, dried over Na_2SO_4, concentrated under vacuum, followed by 3-fluoropyridine (8.6 µL, 0.1 mmol) as an internal standard. After NMR analysis, the mixture was then concentrated again to give the crude, which was purified by column chromatography on silica gel to give the product **3ab**.

4.2. General procedure for the optimization of the cross-coupling of alkyl fluoride with arylalkanes (General Procedure C)

In a N_2 filled glovebox, to a flame-dried screw-capped test tube was added alkylfluoride **1** (0.2 mmol, 1.0 equiv), silylboronate $Et_3SiBpin$ (96.8 mg, 0.4 mmol, 2.0 equiv), arylalkanes **2**, KO^{t}Bu (89.6 mg, 0.8 mmol, 4.0 equiv), and diglyme (2.0 mL) sequentially. The tube then was sealed and removed from the glovebox. The solution was stirred at 60 ^{o}C for 3.0 hours. The reaction tube was quenched with saturated NH_4Cl (5 mL), then extracted with Et_2O, washed with large amount water and brine, dried over Na_2SO_4, concentrated under vacuum, followed by 3-fluoropyridine (8.6 µL, 0.1 mmol) as an internal standard. After NMR analysis, the mixture was then concentrated again to give the crude, which was purified by column chromatography on silica gel to give the product **3**.

4.3. Characterization data of products

1-Methoxy-4-(1-phenylnonyl)benzene (3aa)

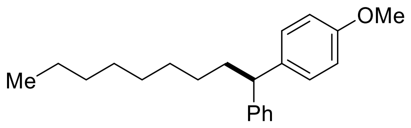

Compound **3aa** was prepared according to the **General Procedure C** start from 1-fluorooctane (33.0 µL, 0.2 mmol), and purified by silica gel column chromatography (*n*-Hexane/DCM = 5/1) as a colorless oil (55.9 mg, Yield: 90%).

^{1}H NMR (300 MHz, $CDCl_3$) δ 7.34 – 7.20 (m, 4H), 7.21 – 7.12 (m, 3H), 6.83 (d, *J* = 8.6 Hz, 2H), 3.85 (t, *J* = 7.8 Hz, 1H), 3.78 (s, 3H), 2.01 (q, *J* = 7.4 Hz, 2H), 1.24 (br-s, 12H), 0.88 (t, *J* = 6.6 Hz, 3H).

MS(EI): *m/z* 310 $[M]^+$. The chemical shifts were consistent with those reported in the literature.[14]

1-Methoxy-4-(1-phenylundecyl)benzene (3ba)

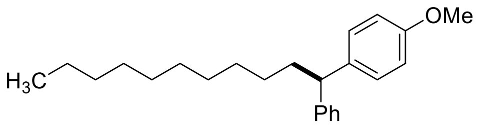

Compound **3ba** was prepared according to the **General Procedure C** start from 1-fluorodecane (32.0 mg, 0.2 mmol), and purified by silica gel column chromatography (*n*-Hexane/DCM = 5/1) as a colorless oil (59.5 mg, Yield: 88%).

^{1}H NMR (300 MHz, $CDCl_3$) δ 7.34 – 7.05 (m, 7H), 6.81 (dd, *J* = 8.6, 1.8 Hz, 2H), 3.83 (t, *J* = 7.8 Hz, 1H), 3.76 (d, *J* = 1.6 Hz, 3H), 1.99 (q, *J* = 7.5 Hz, 2H), 1.22 (br-s, 16H), 0.87 (t, *J* = 6.1 Hz, 3H).

^{13}C NMR (176 MHz, $CDCl_3$) δ 157.9, 145.9, 137.7, 128.9, 128.5, 127.9, 126.0, 113.8, 55.3, 50.6, 36.0, 32.0, 29.8, 29.8, 29.7, 29.6, 29.5, 28.2, 22.8, 14.3.

IR (KBr): 3339, 3276, 2918, 2854, 1611, 1510, 1464, 1302, 1249, 1177, 1039, 826, 698 cm^{-1}.

HRMS (EI) $[C_{24}H_{34}O]$ $[M]^+$ calculated: 338.2610, found: 338. 2600.

1-Methoxy-4-(1-phenylnonadecyl)benzene (3ca)

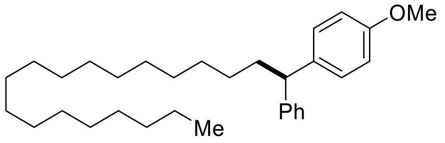

Compound **3ca** was prepared according to the **General Procedure C** start from 1-fluorooctadecane (54.4 mg, 0.2 mmol), and purified by silica gel column chromatography (*n*-Hexane/DCM = 5/1) as a white solid (81.1 mg, Yield: 85%), m.p. = 35.3 –36.2 ^{o}C.

^{1}H NMR (300 MHz, $CDCl_3$) δ 7.32 – 7.19 (m, 4H), 7.19 – 7.09 (m, 3H), 6.81 (d, *J* = 8.6 Hz, 2H), 3.83 (t, *J* = 7.8 Hz, 1H), 3.76 (s, 3H), 1.99 (q, *J* = 7.4 Hz, 2H), 1.25 (br-s, 32H), 0.88 (t, *J* = 6.5 Hz, 3H).

^{13}C NMR (75 MHz, $CDCl_3$) δ 157.9, 145.9, 137.6, 128.9, 128.5, 127.9, 126.0, 113.8, 55.3, 50.6, 36.0, 32.1, 29.9, 29.83, 29.81, 29.7, 29.5, 28.2, 22.9, 14.3.

IR (KBr): 3028, 2954, 2919, 2848, 1611, 1512, 1465, 1302, 1252, 1178, 1035, 827, 789, 723, 700 cm^{-1}.

HRMS (EI) [$C_{32}H_{50}O$] [M]$^{+}$ calculated: 450.3862, found: 450.3866.

1-(4-(4-Methoxyphenyl)-4-phenylbutyl)adamantane (3da)

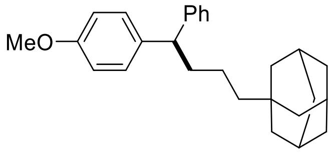

Compound **3da** was prepared according to the **General Procedure C** start from 1-(3-fluoropropyl)adamantane (39.2 mg, 0.2 mmol), and purified by silica gel column chromatography (*n*-Hexane/toluene = 1/1) as a colorless oil (70.3 mg, Yield: 94%).

^{1}H NMR (300 MHz, $CDCl_3$) δ 7.30 – 7.15 (m, 4H), 7.18 – 7.08 (m, 3H), 6.86 – 6.72 (m, 2H), 3.85 (t, *J* = 7.8 Hz, 1H), 3.76 (d, *J* = 1.9 Hz, 3H), 1.94 (q, *J* = 7.5 Hz, 2H), 1.88 (s, 3H), 1.72 – 1.50 (m, 6H), 1.38 (s, 6H), 1.28 – 1.12 (m, 2H), 1.12 – 1.02 (m, 2H).

^{13}C NMR (75 MHz, $CDCl_3$) δ 157.8, 146.0, 137.7, 128.9, 128.5, 127.9, 126.0, 113.8, 55.3, 50.5, 44.8, 42.6, 37.4, 32.4, 28.7, 28.9, 20.9.

IR (KBr): 3060, 3029, 2952, 2915, 2836, 1609, 1512, 1450, 1301, 1250, 1177, 1101, 1038, 825, 784, 699 cm^{-1}.

HRMS (EI) [$C_{27}H_{34}O$] [M]$^{+}$ calculated: 374.2610, found: 374.2603.

1-Methoxy-4-(1-phenylundec-10-en-1-yl)benzene (3ea)

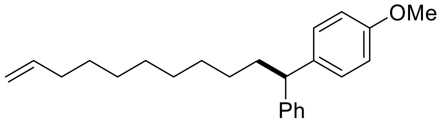

Compound **3ea** was prepared according to the **General Procedure C** start from 10-fluorodec-1-ene (31.6 mg, 0.2 mmol), and purified by silica gel column chromatography (*n*-Hexane/DCM = 5/1) as a colorless oil (53.1 mg, Yield: 79%).

^{1}H NMR (700 MHz, $CDCl_3$) δ 7.32 – 7.23 (m, 2H), 7.27 – 7.18 (m, 2H), 7.23 – 7.13 (m, 1H), 7.20 – 7.12 (m, 2H), 6.83 (d, *J* = 8.7 Hz, 2H), 5.88 – 5.76 (m, 1H), 5.04 – 4.95 (m, 1H), 4.98 – 4.89 (m, 1H), 3.84 (t, *J* = 7.8 Hz, 1H), 3.78 (s, 3H), 2.08 – 1.95 (m, 4H), 1.38 – 1.33 (m, 2H), 1.31 (d, *J* = 6.8 Hz, 2H), 1.28 – 1.21 (m, 8H).

^{13}C NMR (176 MHz, $CDCl_3$) δ 157.9, 145.9, 139.4, 137.7, 128.9, 128.5, 127.9, 126.0, 114.2, 113.8, 55.3, 50.6, 36.0, 34.0, 29.8, 29.59, 29.58, 29.2, 29.0, 28.2.

IR (KBr): 3062, 3030, 2925, 2855, 1610, 1510, 1463, 1301, 1249, 1177, 1038, 909, 825, 699 cm^{-1}.

HRMS (EI) [$C_{24}H_{32}O$] $[M]^+$ calculated: 336.2453, found: 336.2437.

(1-(4-Methoxyphenyl)butane-1,4-diyl)dibenzene (3fa)

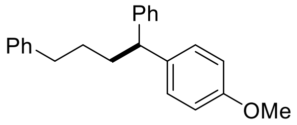

Compound **3fa** was prepared according to the **General Procedure C** start from (3-fluoropropyl)benzene (30.4 mg, 0.2 mmol), and purified by silica gel column chromatography (*n*-Hexane/DCM = 5/1) as a white solid (31.0 mg, Yield: 49%).

^{1}H NMR (300 MHz, $CDCl_3$) δ 7.30 – 7.07 (m, 12H), 6.80 (d, *J* = 8.6 Hz, 2H), 3.86 (t, *J* = 7.8 Hz, 1H), 3.75 (s, 3H), 2.63 (t, *J* = 7.7 Hz, 2H), 2.04 (q, *J* = 7.9 Hz, 2H), 1.60 (q, *J* = 7.9 Hz, 2H).

MS(EI): *m/z* 316 $[M]^+$. The chemical shifts were consistent with those reported in the literature.[15]

(1-(4-Methoxyphenyl)pentane-1,5-diyl)dibenzene (3ga)

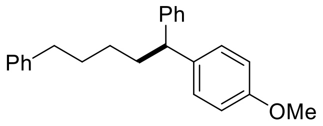

Compound **3ga** was prepared according to the **General Procedure C** start from (4-fluorobutyl)benzene (30.4 mg, 0.2 mmol), and purified by silica gel column chromatography (*n*-Hexane/DCM = 5/1) as a colorless oil (50.2 mg, Yield: 76%).

^{1}H NMR (300 MHz, $CDCl_3$) δ 7.39 – 7.02 (m, 12H), 6.81 (d, *J* = 8.6 Hz, 2H), 3.83 (t, *J* = 8.0 Hz, 1H), 3.75 (s, 3H), 2.55 (t, *J* = 7.9 Hz, 2H), 2.03 (q, *J* = 7.8 Hz, 2H), 1.64 (p, *J* = 7.8 Hz, 2H), 1.31 (p, *J* = 7.9 Hz, 2H).

^{13}C NMR (176 MHz, $CDCl_3$) δ 157.9, 145.7, 142.8, 137.5, 128.9, 128.49, 128.48, 128.4, 127.9, 126.1, 125.7, 113.9, 55.3, 50.5, 35.9, 35.8, 31.6, 27.8.

IR (KBr): 3061, 3028, 2932, 2857, 1609, 1512, 1495, 1454, 1300, 1248, 1177, 1035, 826, 746, 699 cm^{-1}.

HRMS (EI) [$C_{24}H_{26}O$] $[M]^+$ calculated: 330.1984, found: 330.1984.

1-(4-Methoxyphenyl)hexane-1,5-diyl)dibenzene (3ha)

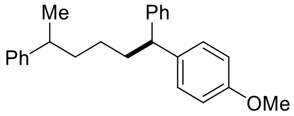

Compound **3ha** was prepared according to the **General Procedure C** start from (5-fluoropentan-2-yl)benzene (33.3 mg, 0.2 mmol), and purified by silica gel column chromatography (*n*-Hexane/DCM = 4/1) as a colorless oil (62.7 mg, Yield: 91%).

^{1}H NMR (700 MHz, $CDCl_3$) δ 7.34 – 7.27 (m, 4H), 7.24 – 7.12 (m, 8H), 6.84 (dd, *J* = 8.7, 4.6 Hz, 2H), 3.84 (t, *J* = 7.8 Hz, 1H), 3.80 (d, *J* = 1.8 Hz, 3H), 2.68 (q, *J* = 7.1 Hz, 1H), 2.09 – 1.94 (m, 2H), 1.72 – 1.57 (m, 2H), 1.34 – 1.17 (m, 2H), 1.24 (d, *J* = 6.9 Hz, 3H).

^{13}C NMR (176 MHz, $CDCl_3$) δ 157.9, 147.8, 145.8, 137.5, 128.8, 128.5, 128.4, 127.8, 127.1, 126.0, 125.9, 113.8, 55.3, 50.4, 39.7, 38.3, 36.0, 26.0, 22.5.

IR (KBr): 3059, 3027, 2956, 2930, 2859, 2834, 1609, 1510, 1493, 1451, 1250, 1177, 1037, 762, 700 cm^{-1}.

HRMS (EI) [$C_{25}H_{28}O$] $[M]^+$ calculated: 344.2140, found: 344.2151.

(1-(4-Methoxyphenyl)-4-methylpentane-1,4-diyl)dibenzene (3ia)

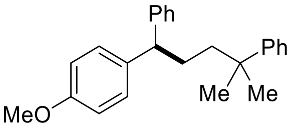

Compound **3ia** was prepared according to the **General Procedure C** start from (4-fluoro-2-methylbutan-2-yl)benzene (33.3 mg, 0.2 mmol), and purified by silica gel column chromatography (*n*-Hexane/DCM = 4/1) as a colorless oil (64.7 mg, Yield: 94%).

^{1}H NMR (700 MHz, $CDCl_3$) δ 7.32 – 7.23 (m, 2H), 7.28 – 7.17 (m, 4H), 7.21 – 7.12 (m, 1H), 7.17 – 7.08 (m, 1H), 7.09 (dd, *J* = 7.7, 1.0 Hz, 2H), 7.02 (d, *J* = 8.8 Hz, 2H), 6.77 (d, *J* = 8.7 Hz, 2H), 3.74 (s, 3H), 3.69 (t, *J* = 7.7 Hz, 1H), 1.81 – 1.71 (m, 2H), 1.63 – 1.53 (m, 2H), 1.26 (s, 6H).

^{13}C NMR (176 MHz, $CDCl_3$) δ 157.9, 149.3, 145.7, 137.4, 128.8, 128.5, 128.2, 127.8, 126.04, 126.00, 125.6, 113.8, 55.3, 51.2, 42.8, 37.8, 31.0, 29.2, 29.1.

IR (KBr): 3057, 3028, 2961, 2937, 2904, 2835, 1610, 1509, 1496, 1463, 1251, 1178, 1038, 765, 699 cm^{-1}.

HRMS (EI) [$C_{25}H_{28}O$] $[M]^+$ calculated: 344.2140, found: 344.2149.

1-Methoxy-4-(1-phenyl-4-(1-phenylcyclopentyl)butyl)benzene (3ja)

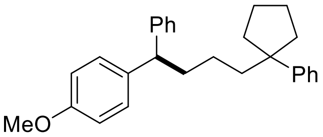

Compound **3ja** was prepared according to the **General Procedure C** start from (2-(fluoromethyl)cyclopropyl)benzene (30.0 mg, 0.2 mmol), and purified by silica gel column chromatography (*n*-Hexane/DCM = 4/1) as a colorless oil (71.4 mg, Yield: 93%).

^{1}H NMR (700 MHz, $CDCl_3$) δ 7.25 – 7.21 (m, 2H), 7.19 (t, *J* = 7.4 Hz, 4H), 7.15 – 7.10 (m, 2H), 7.08 (d, *J* = 8.0 Hz, 2H), 7.01 (d, *J* = 8.6 Hz, 2H), 6.75 (d, *J* = 8.7 Hz, 2H), 3.73 (s, 3H), 3.71 (t, *J* = 7.8 Hz, 1H), 1.82 (q, *J* = 7.4 Hz, 4H), 1.73 (dt, *J* = 11.9, 5.8 Hz, 2H), 1.69 – 1.63 (m, 2H), 1.63 – 1.56 (m, 4H), 1.04 – 0.92 (m, 2H).

^{13}C NMR (75MHz, $CDCl_3$) δ 157.8, 149.0, 145.7, 137.5, 128.8, 128.4, 127.9, 127.8, 126.9, 125.9, 125.3, 113.7, 55.3, 51.1, 49.9, 41.7, 37.74, 37.68, 36.4, 23.3, 23.2.

IR (KBr): 3059, 3026, 2953, 2869, 2834, 1610, 1510, 1495, 1453, 1249, 1177, 1037, 765, 699 cm^{-1}.

HRMS (EI) [$C_{28}H_{32}O$] $[M]^+$ calculated: 384.2453, found: 384.2461.

1-Cyclohexyl-2-(4-methoxyphenyl)ethane-1,2-diyl)dibenzene (3ka)

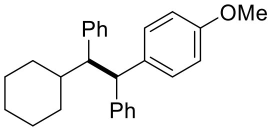

Compound **3ka** was prepared according to the **General Procedure C** start from (cyclohexylfluoromethyl)benzene (38.5 mg, 0.2 mmol), and purified by silica gel column chromatography (*n*-Hexane/toluene = 1/1) as a white solid (51.1 mg, Yield: 69%), m.p. = 123.3 – 124.1 ^{o}C.

^{1}H NMR (700 MHz, $CDCl_3$) δ 7.37 (d, *J* = 8.0 Hz, 2H), 7.29 (t, *J* = 7.2 Hz, 2H), 7.18 – 7.10 (m, 5H), 7.09 – 7.03 (m, 3H), 6.57 (d, *J* = 8.7 Hz, 2H), 4.46 (d, *J* = 12.4 Hz, 1H), 3.62 (s, 3H), 3.45 (dd, *J* = 12.3, 2.6 Hz, 1H), 1.91 (d, *J* = 12.8 Hz, 1H), 1.62 (d, *J* = 11.3 Hz, 1H), 1.50 (d, *J* = 12.7 Hz, 1H), 1.44 (d, *J* = 11.5 Hz, 1H), 1.42 – 1.37 (m, 1H), 1.26 (s, 1H), 1.10 – 1.01 (m, 1H), 0.97 – 0.88 (m, 1H), 0.89 – 0.72 (m, 3H).

^{13}C NMR (176 MHz, $CDCl_3$) δ 157.2, 145.2, 140.6, 136.5, 129.3, 128.8, 127.9, 127.3, 126.2, 125.7, 113.5, 100.1, 55.3, 55.1, 52.4, 39.3, 33.3, 27.0, 26.9, 26.6, 26.5.

IR (KBr): 3061, 2922, 2851, 1612, 1509, 1453, 1252, 1178, 1035, 776, 701 cm^{-1}.

HRMS (EI) [$C_{27}H_{30}O$] $[M]^+$ calculated: 370.2297, found: 370.2292.

Nonane-1,1-diyldibenzene (3ab)

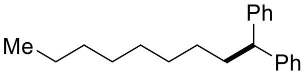

Compound **3ab** was prepared according to the **General Procedure C** start from 1-fluorooctane (33.0 µL, 0.2 mmol), and purified by silica gel column chromatography (*n*-Hexane) as a colorless oil (49.5 mg, Yield: 88%).

^{1}H NMR (300 MHz, $CDCl_3$) δ 7.30 – 7.20 (m, 8H), 7.18 – 7.11 (m, 2H), 3.88 (t, *J* = 7.8 Hz, 1H), 2.15 – 1.92 (m, 2H), 1.31 – 1.14 (m, 12H), 0.86 (t, *J* = 6.7 Hz, 3H).

^{13}C NMR (75 MHz, $CDCl_3$) δ 145.5, 128.5, 128.0, 126.1, 51.5, 35.9, 32.0, 29.8, 29.6, 29.5, 28.2, 22.8, 14.3.

MS(EI): *m/z* 280 $[M]^+$. The chemical shifts were consistent with those reported in the literature.[16]

Undecane-1,1-diyldibenzene (3bb)

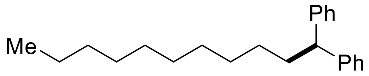

Compound **3bb** was prepared according to the **General Procedure C** start from 1-fluorodecane (32.0 mg, 0.2 mmol), and purified by silica gel column chromatography (*n*-Hexane) as a colorless oil (54.7 mg, Yield: 89%).

^{1}H NMR (300 MHz, $CDCl_3$) δ 7.32 – 7.19 (m, 8H), 7.19 – 7.09 (m, 2H), 3.87 (t, *J* = 7.8 Hz, 1H), 2.02 (q, *J* = 7.5 Hz, 2H), 1.22 (br-s, 16H), 0.87 (t, *J* = 5.8 Hz, 3H).

^{13}C NMR (75 MHz, $CDCl_3$) δ 145.5, 128.5, 128.0, 126.1, 51.5, 35.9, 32.1, 29.8, 29.7, 29.6, 29.5, 28.2, 22.8, 14.3.

IR (KBr): 3027, 2956, 2926, 2854, 1601, 1494, 1463, 1451, 745, 699 cm^{-1}.

MS(EI): *m/z* 308 $[M]^+$. The chemical shifts were consistent with those reported in the literature.[17]

Nonadecane-1,1-diyldibenzene (3cb)

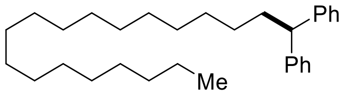

Compound **3cb** was prepared according to the **General Procedure C** start from 1-fluorooctadecane (54.4 mg, 0.2 mmol), and purified by silica gel column chromatography (*n*-Hexane) as a colorless solid (72.3 mg, Yield: 86%). m.p. = 46.5 – 47.4 ^{o}C.

^{1}H NMR (300 MHz, $CDCl_3$) δ 7.38 – 7.14 (m, 10H), 3.92 (t, *J* = 7.8 Hz, 1H), 2.08 (q, *J* = 7.5 Hz, 2H), 1.30 (s, 32H), 0.93 (t, *J* = 6.7 Hz, 3H).

^{13}C NMR (126 MHz, $CDCl_3$) δ 145.5, 128.5, 128.0, 126.1, 51.5, 35.9, 32.1, 29.9, 29.82, 29.79, 29.7, 29.5, 28.2, 22.9, 14.3.

IR (KBr): 3026, 2916, 2850, 1599, 1493, 1472, 1450, 1032, 750, 698 cm^{-1}.

HRMS (ESI) $[C_{31}H_{48}]$ $[M+Na]^+$ calculated: 443.3654, found: 443.3658.

1-(4,4-Diphenylbutyl)adamantane (3db)

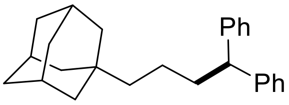

Compound **3db** was prepared according to the **General Procedure C** start from 1-(3-fluoropropyl)adamantane (39.2 mg, 0.2 mmol), and purified by silica gel column chromatography (*n*-Hexane) as a colorless oil (62.6 mg, Yield: 91%).

¹H NMR (300 MHz, $CDCl_3$) δ 7.31 – 7.20 (m, 8H), 7.19 – 7.12 (m, 2H), 3.90 (t, *J* = 7.8 Hz, 1H), 1.98 (q, *J* = 7.6 Hz, 2H), 1.89 (s, 3H), 1.61 (q, *J* = 12.3 Hz, 6H), 1.39 (s, 6H), 1.28 – 1.14 (m, 2H), 1.12 – 1.03 (m, 2H).

¹³C NMR (75 MHz, $CDCl_3$) δ 145.5, 128.5, 128.0, 126.1, 51.4, 44.8, 42.6, 37.4, 36.9, 32.4, 28.8, 20.9.

IR (KBr): 3058, 3023, 2898, 2845, 2671, 1600, 1492, 1448, 739, 697 cm^{-1}.

HRMS (ESI) [$C_{26}H_{32}Na$] $[M+Na]^+$ calculated: 367.2402, found: 367.2404.

Undec-10-ene-1,1-diyldibenzene (3eb)

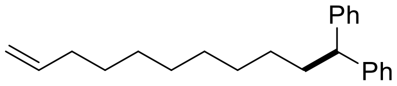

Compound **3eb** was prepared according to the **General Procedure C** start from 10-fluorodec-1-ene (31.6 mg, 0.2 mmol), and purified by silica gel column chromatography (*n*-Hexane) as a colorless oil (47.2 mg, Yield: 77%).

¹H NMR (300 MHz, $CDCl_3$) δ 7.32 – 7.19 (m, 8H), 7.19 – 7.10 (m, 2H), 5.80 (ddt, *J* = 16.9, 10.1, 6.7 Hz, 1H), 5.07 – 4.84 (m, 2H), 3.87 (t, *J* = 7.8 Hz, 1H), 2.17 – 1.90 (m, 4H), 1.40 – 1.15 (m, 12H).

¹³C NMR (75 MHz, $CDCl_3$) δ 145.5, 139.4, 128.5, 128.0, 126.1, 114.2, 51.5, 35.9, 34.0, 29.8, 29.6, 29.5, 29.2, 29.0, 28.2.

IR (KBr): 3027, 2926, 2854, 1640, 1600, 1494, 1451, 746, 699 cm^{-1}.

HRMS (ESI) [$C_{23}H_{30}Na$] $[M+Na]^+$ calculated: 329.2245, found: 329.2248.

Butane-1,1,4-triyltribenzene (3fb)

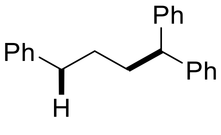

Compound **3fb** was prepared according to the **General Procedure C** start from (3-fluoropropyl)benzene (27.6 mg, 0.2 mmol), and purified by silica gel column chromatography (*n*-Hexane) as a colorless oil (29.8 mg, Yield: 52%).

¹H NMR (300 MHz, $CDCl_3$) δ 7.39 – 6.98 (m, 15H), 3.90 (t, *J* = 7.8 Hz, 1H), 2.63 (t, *J* = 7.7 Hz, 2H), 2.15 – 2.00 (m, 2H), 1.67 – 1.54 (m, 2H).

MS(EI): *m/z* 286 $[M]^+$. The chemical shifts were consistent with those reported in the literature.[18]

Pentane-1,1,5-triyltribenzene (3gb)

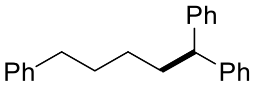

Compound **3gb** was prepared according to the **General Procedure C** start from (4-fluorobutyl)benzene (30.4 mg, 0.2 mmol), and purified by silica gel column chromatography (*n*-Hexane) as a colorless oil (43.9 mg, Yield: 73%).

^{1}H NMR (300 MHz, $CDCl_3$) δ 7.41 – 6.99 (m, 15H), 3.88 (t, *J* = 7.8 Hz, 1H), 2.55 (m, *J* = 7.8 Hz, 2H), 2.07 (q, *J* = 7.8 Hz, 2H), 1.64 (p, *J* = 7.6 Hz, 2H), 1.32 (tt, *J* = 10.3, 6.3 Hz, 2H).

MS(EI): *m/z* 300 $[M]^+$. The chemical shifts were consistent with those reported in the literature.[19]

Hexane-1,1,5-triyltribenzene (3hb)

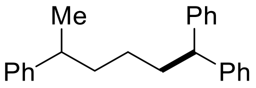

Compound **3hb** was prepared according to the **General Procedure C** start from (5-fluoropentan-2-yl)benzene (33.2 mg, 0.2 mmol), and purified by silica gel column chromatography (*n*-Hexane) as a colorless oil (56.6 mg, Yield: 90%).

^{1}H NMR (300 MHz, $CDCl_3$) δ 7.43 – 7.05 (m, 15H), 3.87 (t, *J* = 7.8 Hz, 1H), 2.67 (q, *J* = 7.1 Hz, 1H), 2.14 – 1.90 (m, 2H), 1.64 (tt, *J* = 8.6, 6.4 Hz, 2H), 1.34 – 1.25 (m, 2H), 1.21 (d, *J* = 7.0 Hz, 3H).

^{13}C NMR (75 MHz, $CDCl_3$) δ 147.8, 145.4, 145.2, 128.5, 128.4, 128.0, 127.9, 127.1, 126.1, 125.9, 51.3, 39.7, 38.3, 35.8, 26.1, 22.5.

IR (KBr): 3060, 3026, 2928, 2861, 1601, 1493, 1451, 1030, 759, 699 cm^{-1}.

HRMS (ESI) $[C_{24}H_{26}Na]$ $[M+Na]^+$ calculated: 337.1932, found: 337.1949.

4-(2,2-Diphenylethyl)-biphenyl (3mb)

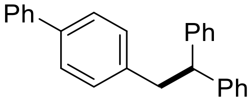

Compound **3mb** was prepared according to the **General Procedure C** start from 4-(fluoromethyl)-biphenyl (37.2 mg, 0.2 mmol), and purified by silica gel column chromatography (*n*-Hexane) as a white solid (54.7 mg, Yield: 82%).

^{1}H NMR (300 MHz, $CDCl_3$) δ 7.57 – 7.49 (m, 2H), 7.43 – 7.34 (m, 4H), 7.32 – 7.26 (m, 2H), 7.25

– 7.20 (m, 7H), 7.19 – 7.11 (m, 2H), 7.06 (d, J = 8.2 Hz, 2H), 4.26 (t, J = 7.8 Hz, 1H), 3.39 (d, J = 7.8 Hz, 2H).

^{13}C NMR (75 MHz, $CDCl_3$) δ 144.6, 141.1, 139.5, 138.8, 129.6, 128.8, 128.5, 128.2, 127.1, 127.0, 126.9, 126.4, 53.1, 41.9.

MS(EI): *m/z* 334 $[M]^+$. The chemical shifts were consistent with those reported in the literature.[20]

(2-(4-(Trifluoromethoxy)phenyl)ethane-1,1-diyl)dibenzene (3nb)

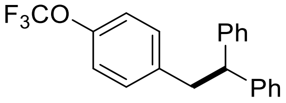

Compound **3nb** was prepared according to the **General Procedure C** start from 1-(fluoromethyl)-4-(trifluoromethoxy)benzene (38.8 mg, 0.2 mmol), and purified by silica gel column chromatography (*n*-Hexane/DCM = 15/1) as a colorless oil (59.6 mg, Yield: 87%).

^{1}H NMR (300 MHz, $CDCl_3$) δ 7.43 – 7.08 (m, 10H), 6.99 (s, 4H), 4.18 (t, J = 7.8 Hz, 1H), 3.35 (d, J = 7.8 Hz, 2H).

^{13}C NMR (126 MHz, $CDCl_3$) δ 147.6, 144.2, 139.1, 130.4, 128.6, 128.1, 126.5, 120.7, 120.6 (q, J = 256.7 Hz), 53.2, 41.5.

^{19}F NMR (282 MHz, $CDCl_3$) δ -58.38 (s, 3F).

IR (KBr): 3062, 3029, 2937, 1600, 1509, 1451, 1262, 1224, 747, 700 cm^{-1}.

HRMS (ESI) [$C_{21}H_{17}F_3NaO$] $[M+Na]^+$ calculated: 365.1129, found: 365.1131.

1-(2,2-Diphenylethyl)naphthalene (3ob)

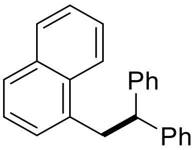

Compound **3ob** was prepared according to the **General Procedure C** start from 1-(fluoromethyl)naphthalene (32.0 mg, 0.2 mmol), and purified by silica gel column chromatography (*n*-Hexane) as a white solid (52.2 mg, Yield: 85%).

^{1}H-NMR (300 MHz, $CDCl_3$) δ 8.05 – 8.02 (m, 1H), 7.86 – 7.83 (m, 1H), 7.66 – 7.64 (m, 1H), 7.49 – 7.46 (m, 2H), 7.26 – 7.15 (m, 11H), 6.89 – 6.87 (m, 1H), 4.44 (t, J = 7.5 Hz, 1H), 3.80 (d, J = 7.5 Hz, 2H).

MS(EI): *m/z* 308 $[M]^+$. The chemical shifts were consistent with those reported in the literature.[20]

2-(2,2-Diphenylethyl)naphthalene (3pb)

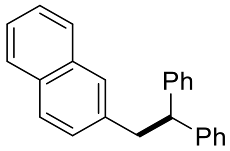

Compound **3pb** was prepared according to the **General Procedure C** start from 2-(fluoromethyl)naphthalene (32.0 mg, 0.2 mmol), and purified by silica gel column chromatography (*n*-Hexane) as a white solid (44.6 mg, Yield: 72%).

^{1}H NMR (300 MHz, $CDCl_3$) δ 8.05 – 8.02 (m, 1H), 7.85 – 7.82 (m, 1H), 7.66 – 7.63 (m, 1H), 7.48 – 7.46 (m, 2H), 7.26 – 7.14 (m, 11H), 6.89 – 6.86 (m, 1H), 4.44 (t, J = 7.5 Hz, 1H), 3.80 (d, J = 7.5 Hz, 2H).

MS(EI): *m/z* 308 $[M]^+$. The chemical shifts were consistent with those reported in the literature.[20]

1-Methyl-2-(1-phenylnonyl)benzene (3ac)

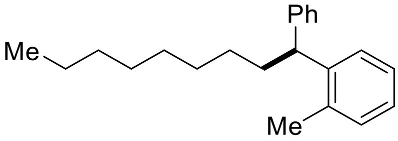

Compound **3ac** was prepared according to the **General Procedure C** start from 1-fluorooctane (33.0 µL, 0.2 mmol), and purified by silica gel column chromatography (*n*-Hexane) as a colorless oil (34.3 mg, Yield: 58%).

^{1}H NMR (300 MHz, $CDCl_3$) δ 7.32 (d, J = 7.2 Hz, 1H), 7.30 – 6.98 (m, 8H), 4.07 (t, J = 7.6 Hz, 1H), 2.26 (s, 3H), 1.99 (q, J = 7.7, 7.3 Hz, 2H), 1.40 – 1.11 (m, 13H), 0.86 (t, J = 6.7 Hz, 3H).

^{13}C NMR (75 MHz, $CDCl_3$) δ 145.1, 143.1, 136.4, 130.6, 128.35, 128.34, 126.8, 126.1, 126.0, 125.9, 47.0, 36.4, 32.0, 29.9, 29.6, 29.5, 28.2, 22.8, 20.1, 14.3.

IR (KBr): 3025, 2926, 2855, 1601, 1492, 1460, 1378, 1031, 748, 699 cm^{-1}.

HRMS (ESI) $[C_{22}H_{30}Na]$ $[M+Na]^+$ calculated: 317.2245, found: 317.2246.

1-Methoxy-2-(1-phenylnonyl)benzene (3ad)

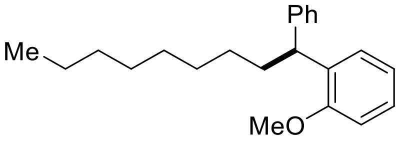

Compound **3ad** was prepared according to the **General Procedure C** start from 1-fluorooctane (33.0 µL, 0.2 mmol), and purified by silica gel column chromatography (*n*-Hexane/DCM = 5/1) as a colorless oil (47.5 mg, Yield: 76%).

^{1}H NMR (300 MHz, $CDCl_3$) δ 7.31 – 7.08 (m, 7H), 6.90 (t, *J* = 7.5 Hz, 1H), 6.81 (d, *J* = 8.2 Hz, 1H), 4.37 (t, *J* = 7.8 Hz, 1H), 3.75 (s, 3H), 1.98 (q, *J* = 7.6 Hz, 2H), 1.25 (s, 12H), 0.86 (t, *J* = 6.5 Hz, 3H).

^{13}C NMR (126 MHz, $CDCl_3$) δ 157.2, 145.5, 134.0, 128.3, 128.2, 127.7, 127.0, 125.8, 120.6, 110.8, 55.6, 43.2, 35.2, 32.0, 29.8, 29.6, 29.5, 28.1, 22.8, 14.3.

IR (KBr): 3062, 3028, 2926, 2854, 1600, 1492, 1461, 1242, 1031, 751, 698 cm^{-1}.

HRMS (ESI) [$C_{22}H_{30}ONa$] [M+Na]$^+$ calculated: 333.2194, found: 333.2192.

1-Chloro-3-(1-phenylnonyl)benzene (3ae)

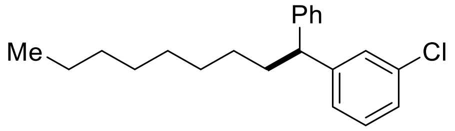

Compound **3ae** was prepared according to the **General Procedure C** start from 1-fluorooctane (33.0 µL, 0.2 mmol), and purified by silica gel column chromatography (*n*-Hexane) as a colorless oil (41.3 mg, Yield: 65%).

^{1}H NMR (300 MHz, $CDCl_3$) δ 7.38 – 7.01 (m, 9H), 3.87 (t, *J* = 7.8 Hz, 1H), 2.03 (q, *J* = 7.6 Hz, 2H), 1.28 (d, *J* = 13.6 Hz, 12H), 0.99 – 0.77 (m, 3H).

^{13}C NMR (126 MHz, $CDCl_3$) δ 147.6, 144.6, 134.3, 129.8, 128.6, 128.1, 127.9, 126.4, 126.3, 126.2, 51.3, 35.7, 32.0, 29.7, 29.6, 29.4, 28.1, 22.8, 14.3.

IR (KBr): 3062, 3027, 2926, 2855, 1594, 1572, 1469, 779, 702 cm^{-1}.

HRMS (ESI) [$C_{21}H_{27}ClNa$] [M+Na]$^+$ calculated: 337.1699, found: 337.1685.

1-Chloro-4-(1-phenylnonyl)benzene (3af)

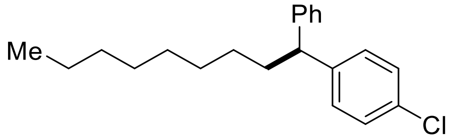

Compound **3af** was prepared according to the **General Procedure C** start from 1-fluorooctane (33.0 µL, 0.2 mmol), and purified by silica gel column chromatography (*n*-Hexane) as a colorless oil (30.9 mg, Yield: 49%).

^{1}H NMR (300 MHz, $CDCl_3$) δ 7.34 – 7.03 (m, 9H), 3.85 (t, *J* = 7.8 Hz, 1H), 2.10 – 1.86 (m, 2H), 1.33 – 1.10 (m, 12H), 0.86 (t, *J* = 6.7 Hz, 3H).

^{13}C NMR (75 MHz, $CDCl_3$) δ 145.0, 144.0, 131.8, 129.3, 128.6, 127.9, 126.3, 50.9, 35.8, 32.0, 29.8, 29.6, 29.4, 28.1, 22.8, 14.3.

IR (KBr): 3028, 2926, 2855, 1601, 1491, 1454, 1092, 1014, 752, 699 cm^{-1}.

HRMS (ESI) [$C_{21}H_{27}ClNa$] $[M+Na]^+$ calculated: 337.1699, found: 337.1704.

4,4'-(Nonane-1,1-diyl)bis(methoxybenzene) (3ag)

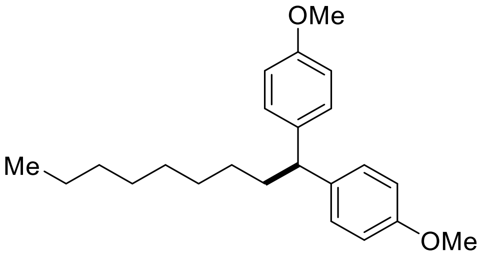

Compound **3ag** was prepared according to the **General Procedure C** start from 1-fluorooctane (33.0 µL, 0.2 mmol), and purified by silica gel column chromatography (*n*-Hexane/EtOAc: 10/1) as a colorless oil (66.7 mg, Yield: 98%).

^{1}H NMR (300 MHz, $CDCl_3$) δ 7.15 (d, *J* = 8.1 Hz, 4H), 6.83 (d, *J* = 8.1 Hz, 4H), 3.89 – 3.65 (m, 7H), 1.98 (q, *J* = 7.3, 6.9 Hz, 2H), 1.25 (s, 12H), 0.88 (t, *J* = 6.5 Hz, 3H).

^{13}C NMR (75 MHz, $CDCl_3$) δ 157.8, 138.1, 128.7, 113.8, 55.3, 49.7, 36.2, 32.0, 29.8, 29.6, 29.5, 28.2, 22.8, 14.3.

IR (KBr): 2998, 2926, 2854, 1610, 1511, 1463, 1247, 1177, 1038, 825 cm^{-1}.

HRMS (ESI) [$C_{23}H_{32}O_2Na$] $[M+Na]^+$ calculated: 363.2300, found: 363.2302.

4,4'-(Nonane-1,1-diyl)bis(methylbenzene) (3ah)

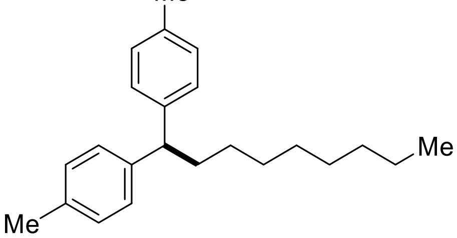

Compound **3ah** was prepared according to the **General Procedure C** start from 1-fluorooctane (33.0 µL, 0.2 mmol), and purified by silica gel column chromatography (*n*-Hexane) as a colorless oil (54.1 mg, Yield: 88%).

^{1}H NMR (300 MHz, $CDCl_3$) δ 7.23 – 7.03 (m, 8H), 3.84 (t, *J* = 7.8 Hz, 1H), 2.32 (s, 6H), 2.02 (q, *J* = 7.7 Hz, 2H), 1.28 (s, 12H), 0.89 (t, *J* = 6.5 Hz, 3H).

^{13}C NMR (75 MHz, $CDCl_3$) δ 142.8, 135.4, 129.2, 127.8, 50.7, 36.0, 32.0, 29.8, 29.6, 29.5, 28.2, 22.8, 21.1, 14.3.

IR (KBr): 3019, 2925, 2855, 1896, 1512, 1461, 808 cm^{-1}.

HRMS (ESI) [$C_{23}H_{33}$] $[M+H]^+$ calculated: 309.2582, found: 309.2596.

4-(1-(*p*-Tolyl)nonyl)-1,1'-biphenyl (3ai)

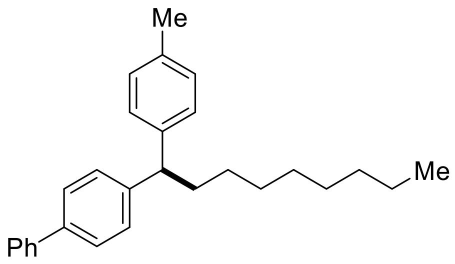

Compound **3ai** was prepared according to the **General Procedure C** start from 1-fluorooctane (33.0 µL, 0.2 mmol), and purified by silica gel column chromatography (*n*-Hexane) as a white solid (38.2 mg, Yield: 51%).

^{1}H NMR (300 MHz, $CDCl_3$) δ 7.62 – 7.55 (m, 2H), 7.54 – 7.47 (m, 2H), 7.47 – 7.38 (m, 2H), 7.37 – 7.28 (m, 3H), 7.22 – 7.15 (m, 2H), 7.15 – 7.08 (m, 2H), 3.91 (t, *J* = 7.8 Hz, 1H), 2.32 (s, 3H), 2.07 (q, *J* = 6.6, 6.0 Hz, 2H), 1.42 – 1.14 (m, 12H), 0.89 (t, *J* = 6.6 Hz, 3H).

^{13}C NMR (75 MHz, $CDCl_3$) δ 145.0, 142.4, 141.2, 138.9, 135.7, 129.3, 128.8, 128.3, 127.9, 127.2, 127.1, 50.8, 35.9, 32.0, 29.8, 29.6, 29.5, 28.2, 22.8, 21.2, 14.3.

IR (KBr): 3026, 2921, 2851, 1604, 1511, 1484, 1459, 754, 694 cm^{-1}.

HRMS (ESI) [$C_{28}H_{34}Na$] $[M+Na]^+$ calculated: 393.2558, found: 393.2563.

9-Octyl-9*H*-xanthene (3aj)

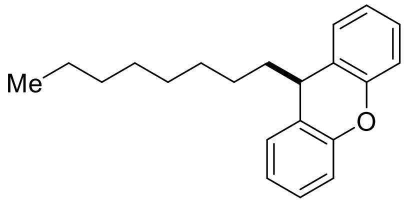

Compound **3aj** was prepared according to the **General Procedure C** start from 1-fluorooctane (33.0 µL, 0.2 mmol), and purified by silica gel column chromatography (*n*-Hexane/DCM: 15/1) as a colorless oil (50.0 mg, Yield: 85%).

^{1}H NMR (300 MHz, $CDCl_3$) δ 7.29 – 7.15 (m, 4H), 7.12 – 7.00 (m, 4H), 3.98 (t, *J* = 6.1 Hz, 1H), 1.72 (q, *J* = 6.8 Hz, 2H), 1.18 (s, 12H), 0.86 (t, *J* = 6.8 Hz, 3H).

^{13}C NMR (126 MHz, $CDCl_3$) δ 152.4, 128.7, 127.5, 126.0, 123.2, 116.4, 40.9, 39.2, 32.0, 29.7, 29.6, 29.4, 25.7, 22.8, 14.2.

IR (KBr): 3064, 3037, 2923, 2848, 1903, 1600, 1575, 1478, 1458, 1254, 752, 717 cm^{-1}.

HRMS (ESI) [$C_{21}H_{26}NaO$] $[M+Na]^+$ calculated: 317.1881, found: 317.1885.

9-Octyl-9,10-dihydroanthracene (3ak)

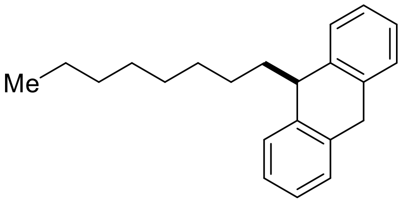

Compound **3ak** was prepared according to the **General Procedure C** start from 1-fluorooctane (33.0 µL, 0.2 mmol), and purified by silica gel column chromatography (*n*-Hexane) as a colorless oil (48.1 mg, Yield: 82%).

^{1}H NMR (300 MHz, $CDCl_3$) δ 7.31 – 7.11 (m, 8H), 4.11 (d, *J* = 18.2 Hz, 1H), 3.83 (d, *J* = 18.4 Hz, 1H), 3.87 (t, *J* = 7.0 Hz, 1H), 1.60 (q, *J* = 7.4 Hz, 2H), 1.22 (d, *J* = 9.2 Hz, 12H), 0.85 (t, *J* = 6.7 Hz, 3H).

^{13}C NMR (75 MHz, $CDCl_3$) δ 141.0, 136.2, 128.1, 127.8, 126.1, 126.0, 47.6, 37.7, 35.5, 32.0, 29.8, 29.7, 29.5, 27.8, 22.8, 14.3.

MS(EI): *m/z* 292 $[M]^+$. The chemical shifts were consistent with those reported in the literature.[21]

Decane-2,2-diyldibenzene (3al)

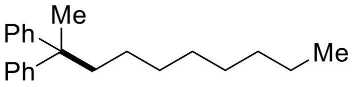

Compound **3al** was prepared according to the **General Procedure C** start from 1-fluorooctane (33.0 µL, 0.2 mmol), and purified by silica gel column chromatography (*n*-Hexane) as a colorless oil (38.2 mg, Yield: 65%).

^{1}H NMR (300 MHz, $CDCl_3$) δ 7.43 – 7.02 (m, 10H), 2.17 – 1.96 (m, 2H), 1.62 (s, 3H), 1.35 – 1.14 (m, 10H), 1.13 – 1.01 (m, 2H), 0.85 (t, *J* = 6.7 Hz, 3H).

^{13}C NMR (75 MHz, $CDCl_3$) δ 150.1, 128.0, 127.5, 125.6, 46.3, 41.9, 32.0, 30.6, 29.6, 29.5, 27.7,

24.8, 22.8, 14.3.

IR (KBr): 3058, 3026, 2928, 2855, 1599, 1494, 1465, 1444, 1029, 757, 699 cm^{-1}.

HRMS (ESI) [$C_{22}H_{30}Na$] $[M+Na]^+$ calculated: 317.2245, found: 317.2245.

Undecane-3,3-diyldibenzene (3am)

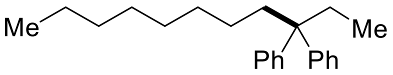

Compound **3am** was prepared according to the **General Procedure C** start from 1-fluorooctane (33.0 µL, 0.2 mmol), and purified by silica gel column chromatography (*n*-Hexane) as a colorless oil (31.5 mg, Yield: 51%).

^{1}H NMR (300 MHz, $CDCl_3$) δ 7.37 – 7.00 (m, 10H), 2.12 (q, *J* = 7.3 Hz, 2H), 2.08 – 2.01 (m, 2H), 1.31 – 1.13 (m, 10H), 0.95 (t, *J* = 7.9 Hz, 2H), 0.85 (t, *J* = 6.8 Hz, 3H), 0.62 (t, *J* = 7.3 Hz, 3H).

^{13}C NMR (75 MHz, $CDCl_3$) δ 149.1, 128.2, 127.8, 125.5, 49.7, 36.9, 32.0, 30.5, 30.1, 29.6, 29.5, 24.0, 22.8, 14.3, 8.6.

IR (KBr): 3087, 3059, 3026, 2929, 2855, 1600, 1495, 1463, 1446, 1031, 753, 700 cm^{-1}.

HRMS (ESI) [$C_{23}H_{32}Na$] $[M+Na]^+$ calculated: 331.2402, found: 331.2415.

(2-Methyldecan-2-yl)benzene (3an)

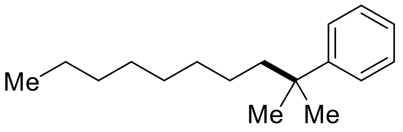

Compound **3an** was prepared according to the **General Procedure C** start from 1-fluorooctane (33.0 µL, 0.2 mmol), and purified by silica gel column chromatography (*n*-Hexane) as a colorless oil (25.2 mg, Yield: 54%).

^{1}H NMR (300 MHz, $CDCl_3$) δ 7.39 – 7.27 (m, 4H), 7.21 – 7.14 (m, 1H), 1.67 – 1.56 (m, 2H), 1.30 (s, 6H), 1.28 – 1.17 (m, 10H), 1.13 – 1.00 (m, 2H), 0.87 (t, *J* = 6.8 Hz, 3H).

^{13}C NMR (75 MHz, $CDCl_3$) δ 149.9, 128.1, 125.9, 125.4, 44.8, 37.8, 32.0, 30.5, 29.7, 29.5, 29.1, 24.9, 22.8, 14.3.

MS(EI): *m/z* 232 $[M]^+$. The chemical shifts were consistent with those reported in the literature.[22]

1-(Decan-2-yl)-3-methoxybenzene (3ao)

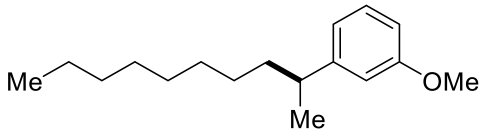

Compound **3ao** was prepared according to the **General Procedure C** start from 1-fluorooctane (33.0 µL, 0.2 mmol), and purified by silica gel column chromatography (*n*-Hexane/DCM: 5/1) as a colorless oil (27.7 mg, Yield: 55%).

^{1}H NMR (300 MHz, $CDCl_3$) δ 7.25 – 7.16 (m, 1H), 6.82 – 6.76 (m, 1H), 6.76 – 6.69 (m, 2H), 3.81 (s, 3H), 2.65 (q, *J* = 7.1 Hz, 1H), 1.62 – 1.47 (m, 2H), 1.34 – 1.14 (m, 15H), 0.87 (t, *J* = 6.7 Hz, 3H).

^{13}C NMR (75 MHz, $CDCl_3$) δ 159.7, 149.9, 129.3, 119.6, 113.1, 110.8, 55.2, 40.2, 38.5, 32.0, 29.9, 29.7, 29.5, 27.9, 22.8, 22.5, 14.3.

IR (KBr): 2957, 2926, 2854, 1606, 1487, 1461, 1261, 1049, 778, 700 cm^{-1}.

HRMS (ESI) [$C_{17}H_{29}O$] $[M+H]^+$ calculated: 249.2218, found: 249.2220.

Tridecan-5-ylbenzene (3ap)

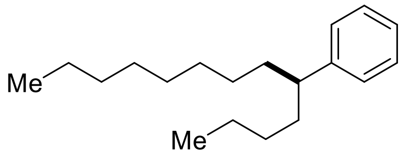

Compound **3ap** was prepared according to the **General Procedure C** start from 1-fluorooctane (33.0 µL, 0.2 mmol), and purified by silica gel column chromatography (*n*-Hexane) as a colorless oil (11.1 mg, Yield: 22%).

^{1}H NMR (300 MHz, $CDCl_3$) δ 7.32 – 7.23 (m, 2H), 7.20 – 7.10 (m, 3H), 2.48 (tt, *J* = 9.0, 5.6 Hz, 1H), 1.65 – 1.44 (m, 4H), 1.34 – 1.03 (m, 14H), 0.85 (q, *J* = 7.1 Hz, 6H).

MS(EI): *m/z* 260 $[M]^+$. The chemical shifts were consistent with those reported in the literature.[23]

Dodec-1-en-4-ylbenzene (3aq)

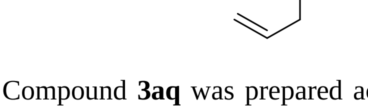

Compound **3aq** was prepared according to the **General Procedure C** start from 1-fluorooctane (33.0 µL, 0.2 mmol), and **failed** to get pure product through column chromatography (*n*-Hexane). As a result, impure colorless oil (12.1 mg, Yield: 24%) were characterized to be the desired product through ^{1}H NMR analysis.

(2-((1*S*,2*R*)-2-phenylcyclopropyl)ethane-1,1-diyl)dibenzene (3sb)

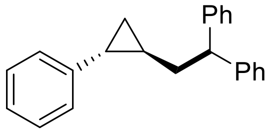

Compound **3sb** was prepared according to the **General Procedure C** start from (2-(fluoromethyl)cyclopropyl)benzene (30.0 mg, 0.2 mmol), and purified by silica gel column chromatography (*n*-hexane/DCM = 10/1) as a colorless oil (48.9 mg, Yield: 82%).

^{1}H NMR (500 MHz, $CDCl_3$) δ 7.27 – 7.19 (m, 8H), 7.19 – 7.12 (m, 4H), 7.11 – 7.06 (m, 1H), 6.90 – 6.86 (m, 2H), 4.07 (t, *J* = 7.7 Hz, 1H), 2.19 (ddd, *J* = 13.7, 8.3, 6.8 Hz, 1H), 2.06 (dt, *J* = 13.8, 7.0 Hz, 1H), 1.61 (dt, *J* = 9.1, 4.8 Hz, 2H), **0.97 (td, *J* = 12.6, 6.8 Hz, 1H), 0.84 (dt, *J* = 8.5, 5.0 Hz, 1H)**, 0.76 (dt, *J* = 8.6, 5.2 Hz, 1H).

^{13}C NMR (75 MHz, $CDCl_3$) δ 145.3, 144.9, 143.5, 128.6, 128.5, 128.2, 128.1, 128.0, 126.3, 126.2, 125.8, 125.3, 51.6, 40.8, 23.8, 22.4, 16.2.

IR (KBr): 3060, 3025, 2913, 1945, 1602, 1494, 1448, 1030, 749, 698 cm^{-1}.

HRMS (ESI) [$C_{23}H_{22}Na$] [M+Na]$^+$ calculated: 307.1463, found: 307.1469.

9-(((1*R*,2*S*)-2-phenylcyclopropyl)methyl)-9*H*-xanthene (3sk)

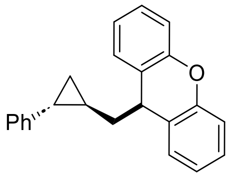

Compound **3sk** was prepared according to the **General Procedure C** start from (2-(fluoromethyl)cyclopropyl)benzene (15.0 mg, 0.1 mmol), and purified by silica gel column chromatography (*n*-Hexane/DCM = 10/1) as a colorless oil (27.7 mg, Yield: 88%).

^{1}H NMR (700 MHz, $CDCl_3$) δ 7.25 – 7.18 (m, 5H), 7.16 (d, *J* = 7.4 Hz, 1H), 7.12 (t, *J* = 7.3 Hz, 1H), 7.08 (d, *J* = 7.7 Hz, 1H), 7.05 – 7.01 (m, 3H), 6.88 (d, *J* = 7.2 Hz, 2H), 4.09 (t, *J* = 6.3 Hz, 1H), 1.88 – 1.75 (m, 2H), **1.43 (dt, *J* = 9.0, 4.8 Hz, 1H), 0.97 (dt, *J* = 12.9, 6.3 Hz, 1H)**, 0.79 (dt, *J* = 8.6, 5.1 Hz, 1H), 0.57 (dt, *J* = 8.6, 5.3 Hz, 1H).

^{13}C NMR (176 MHz, $CDCl_3$) δ 152.4, 152.3, 143.4, 128.9, 128.2, 127.63, 127.62, 125.60, 125.59, 125.5, 125.3, 123.2, 123.1, 116.6, 116.4, 45.8, 39.6, 23.3, 20.7, 16.2.

IR (KBr): 3065, 3040, 2908, 1603, 1576, 1479, 1325, 1259, 1219, 1093, 1034, 756, 697 cm^{-1}.

HRMS (EI) [$C_{23}H_{30}NaO$] [M]$^+$ calculated: 321.1255, found: 321.1262.

5. Mechanistic control experiments

5.1. Cross-coupling reactions using alkyl halides

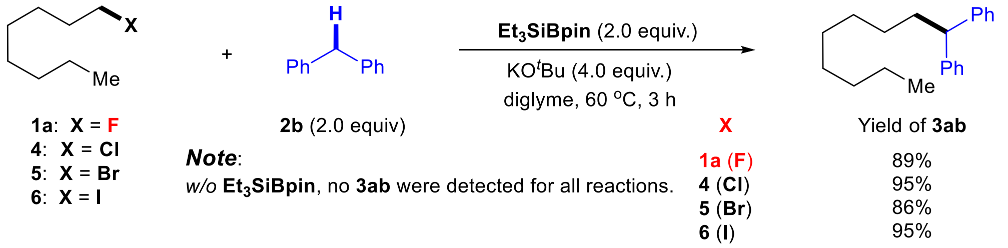

Following the **General Procedure C**, charging KO*t*Bu (45 mg, 0.4 mmol), anhydrous diglyme (0.5 mL), silylboronate $Et_3SiBpin$ (48.4 mg, 0.2 mmol), TEMPO, **2b** (33.6 mg, 0.2 mmol), alkyl halide **1a**, **4**, **5** or **6** (0.1 mmol), and then anhydrous diglyme (0.5 mL) sequentially. The reactions were move out of glovebox and stirred at 60 °C for 3 h. The reaction mixture was then quenched by adding *n*-hexane (5.0 mL) and saturated NH_4Cl (2.0 mL) while stirring for 5 min, then the reaction mixture was extracted with Et_2O, washed by water, dried over Na_2SO_4, and concentrated under vacuum, followed by 3-fluoropyridine (8.6 µL, 0.1 mmol) as an internal standard. Then the ^1H NMR analysis of the crude were conducted to show the details of the control reactions.

Using of **1-fluorooctane** (**without Et₃SiBpin**)

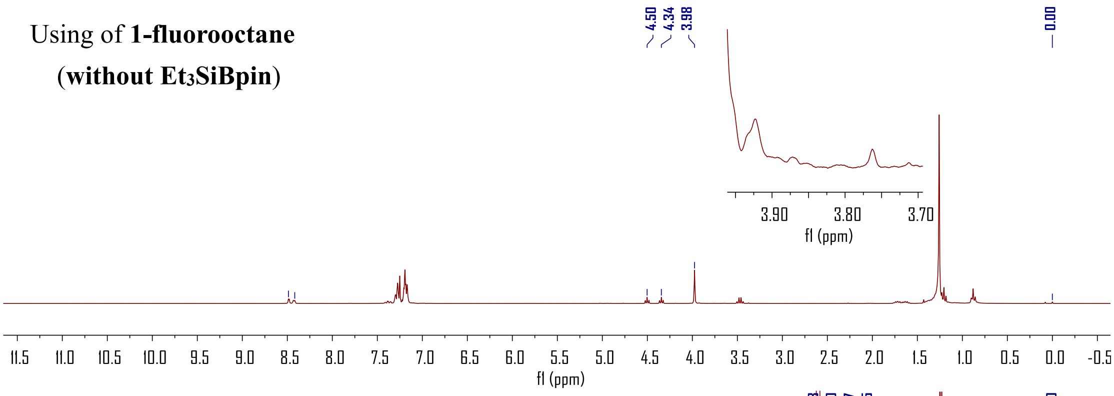

Using of **1-fluorooctane** (**with Et₃SiBpin**)

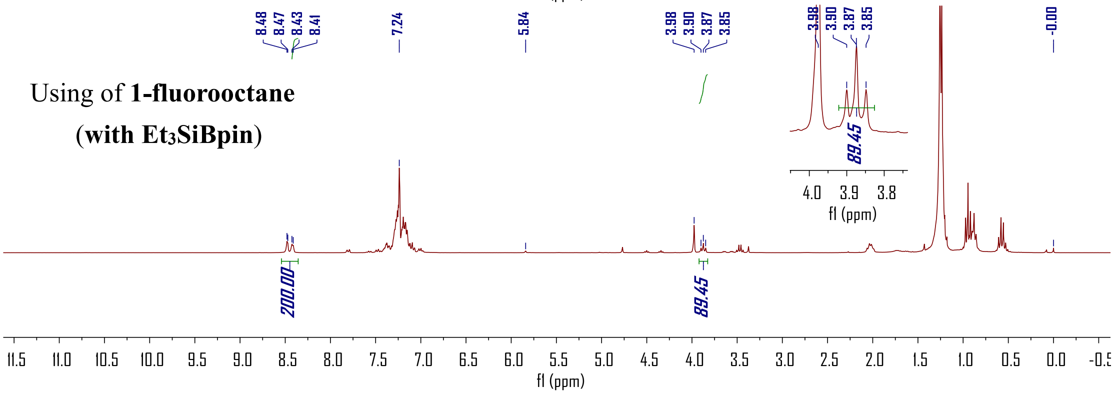

Using of **1-chlorooctane**
(**without $Et_3SiBpin$**)

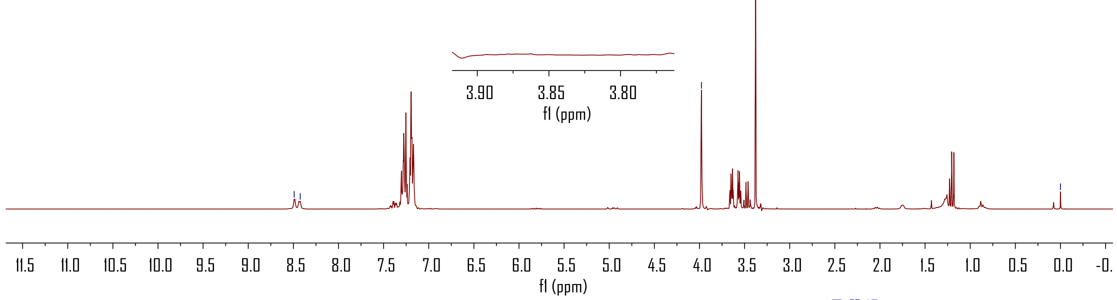

Using of **1-chlorooctane**
(**with $Et_3SiBpin$**)

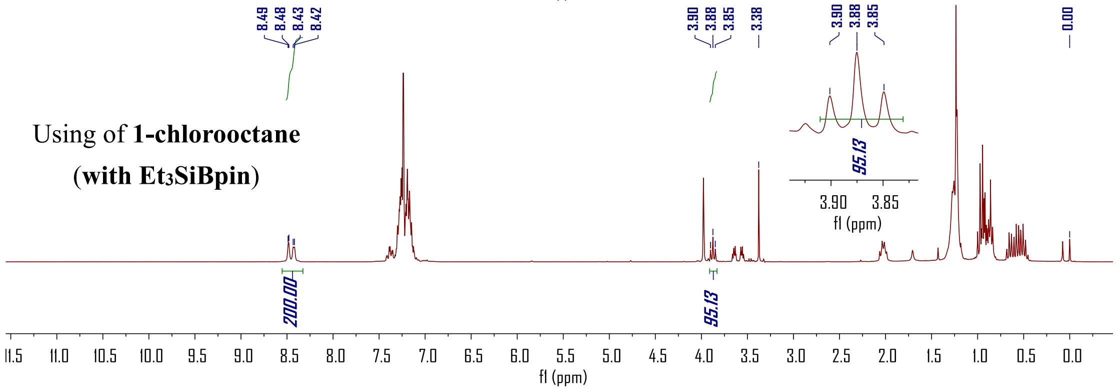

Using of **1-iodooctane**
(**without $Et_3SiBpin$**)

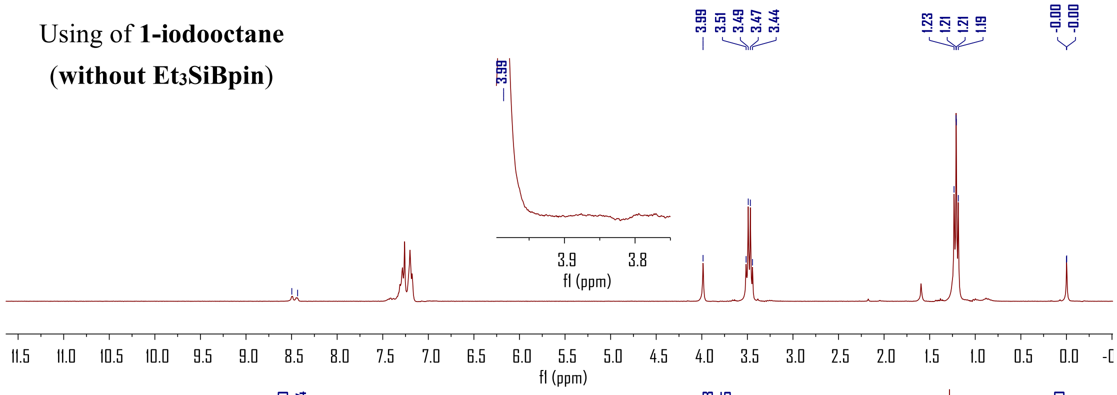

Using of **1-iodooctane**
(**with $Et_3SiBpin$**)

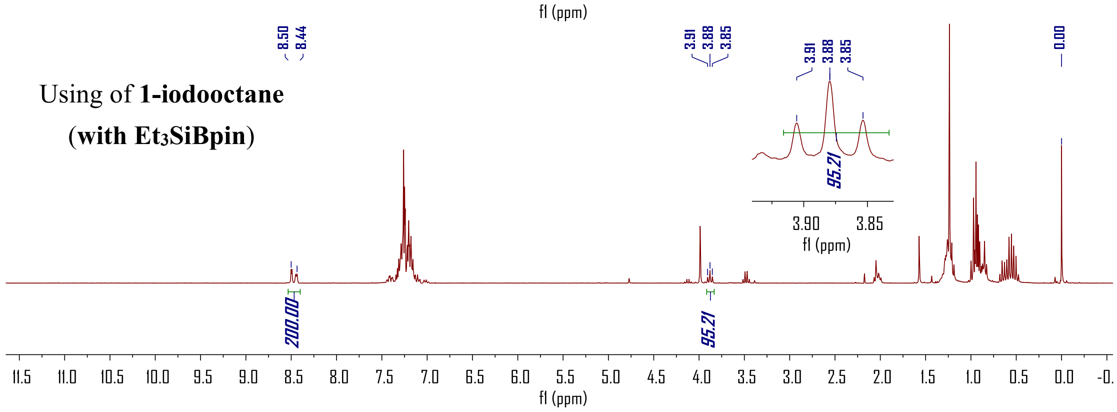

Using of **1-bromooctane** (**with $Et_3SiBpin$**)

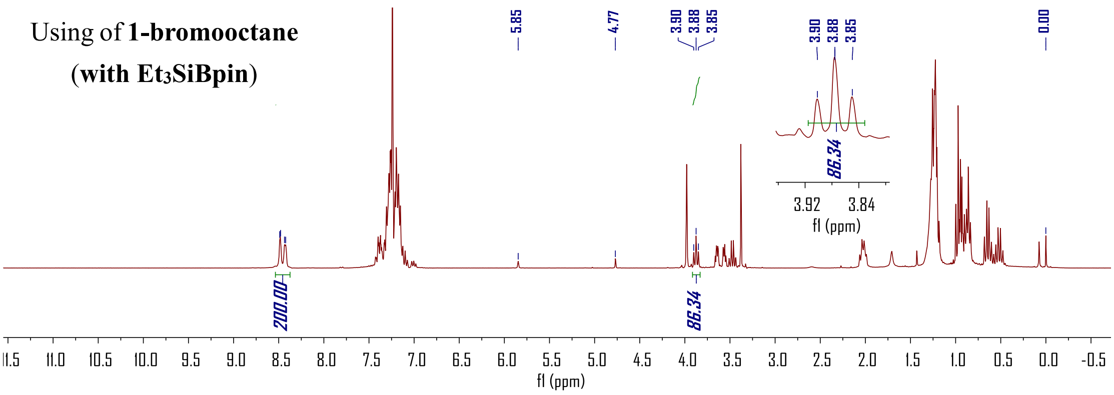

5.2. Reaction with radical scavenger TEMPO

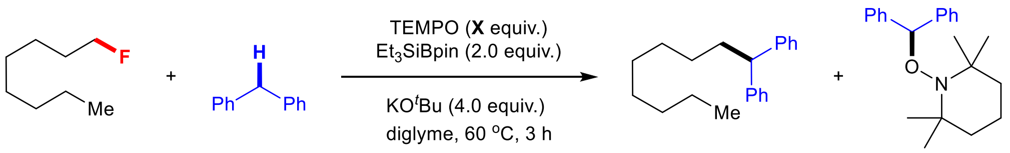

	1a	2b (2.0 equiv)	3ab	Int-TEMPO
**X** = 1.0			26%	14%
**X** = 2.0			8%	63%
**X** = 4.0			0%	134%

Following the **General Procedure C**, charging KO*t*Bu (45 mg, 0.4 mmol), anhydrous diglyme (0.5 mL), silylboronate $Et_3SiBpin$ (48.4 mg, 0.2 mmol), TEMPO, **2b** (33.6 mg, 0.2 mmol), **1a** (16.5 µL, 0.1 mmol), and then anhydrous diglyme (0.5 mL) sequentially. The reactions were move out of glovebox and stirred at 60 °C for 3 h. The reaction mixture was then quenched by adding *n*-hexane (5.0 mL) and saturated NH_4Cl (2.0 mL) while stirring for 5 min, then the reaction mixture was extracted with Et_2O, washed by water, dried over Na_2SO_4, and concentrated under vacuum, followed by 3-fluoropyridine (8.6 µL, 0.1 mmol) as an internal standard. Then the ^1H NMR analysis and ^{19}F NMR analysis of the crude were conducted to show the details of the control reactions.

Using of 1.0 equivalent of **TEMPO**

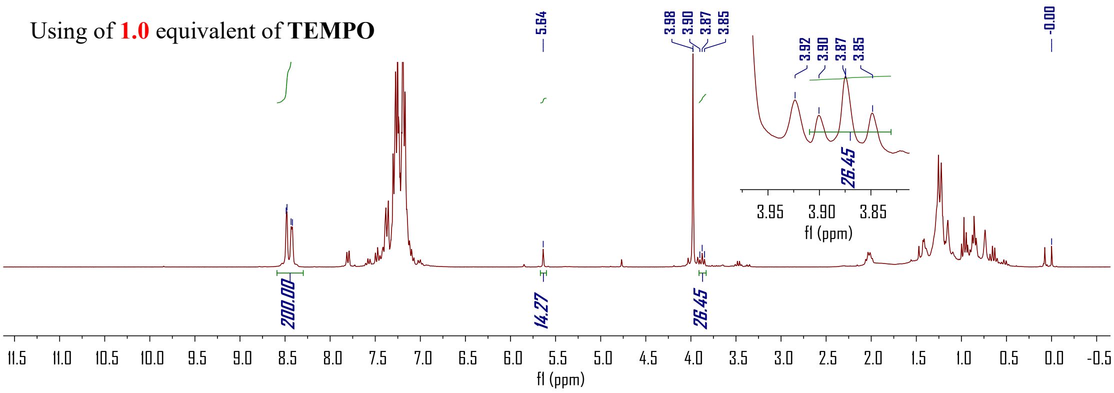

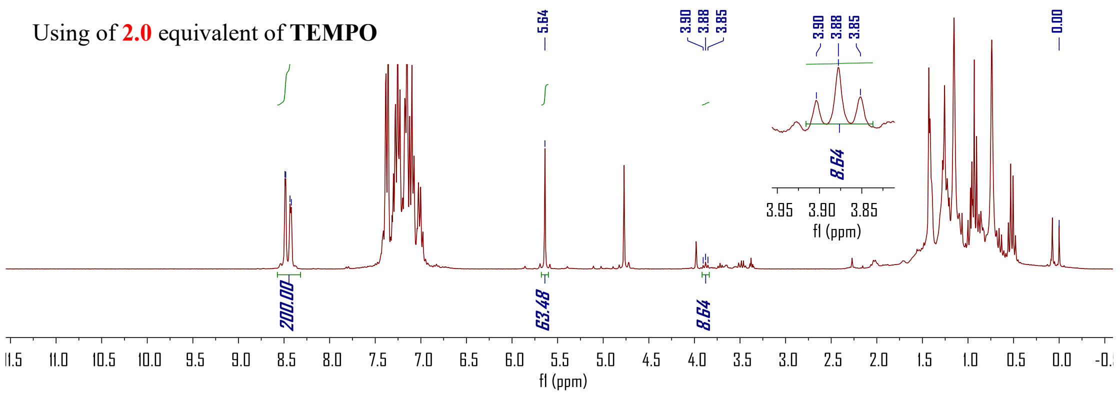

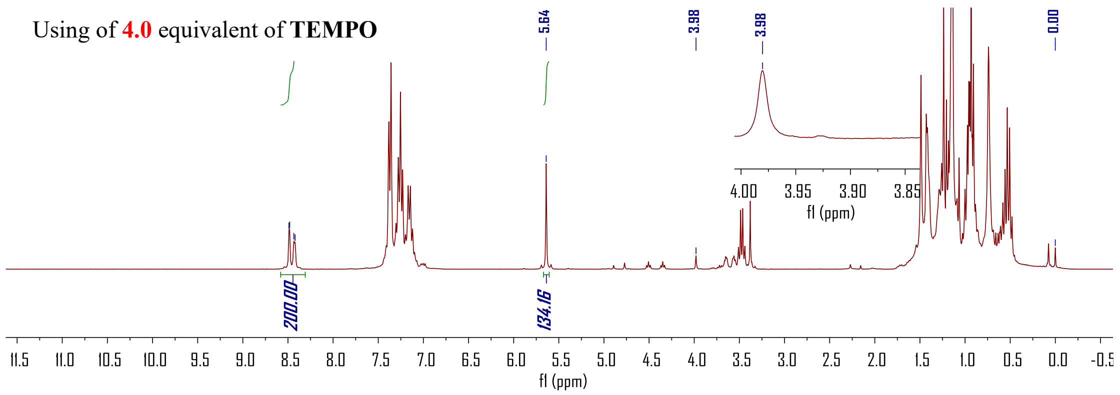

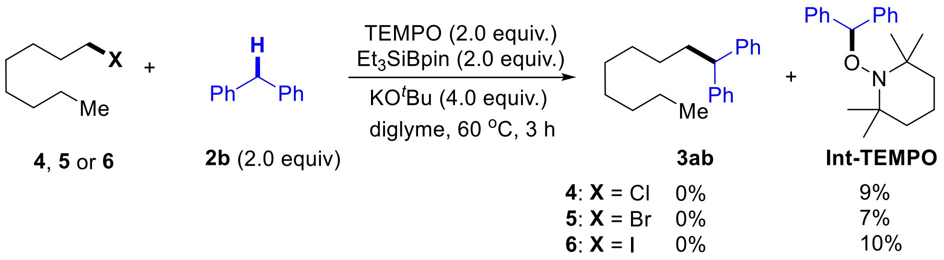

Following the **General Procedure C**, charging KO*t*Bu (22.5 mg, 0.2 mmol), anhydrous diglyme (0.5 mL), silylboronate $Et_3SiBpin$ (48.4 mg, 0.2 mmol), TEMPO (32.0 mg, 0.2 mmol), **2b** (33.6 mg, 0.2 mmol), alkyl halide **4**, **5** or **6** (0.1 mmol), and then anhydrous diglyme (0.5 mL) sequentially. The reactions were move out of glovebox and stirred at 60 °C for 3 h. The reaction mixture was then quenched by adding *n*-hexane (5.0 mL) and saturated NH_4Cl (2.0 mL) while stirring for 5 min, then the reaction mixture was extracted with Et_2O, washed by water, dried over Na_2SO_4, and concentrated under vacuum, followed by 3-fluoropyridine (8.6 μL, 0.1 mmol) as an internal standard. Then the ^1H NMR analysis of the crude were conducted to show the details of the control reactions.

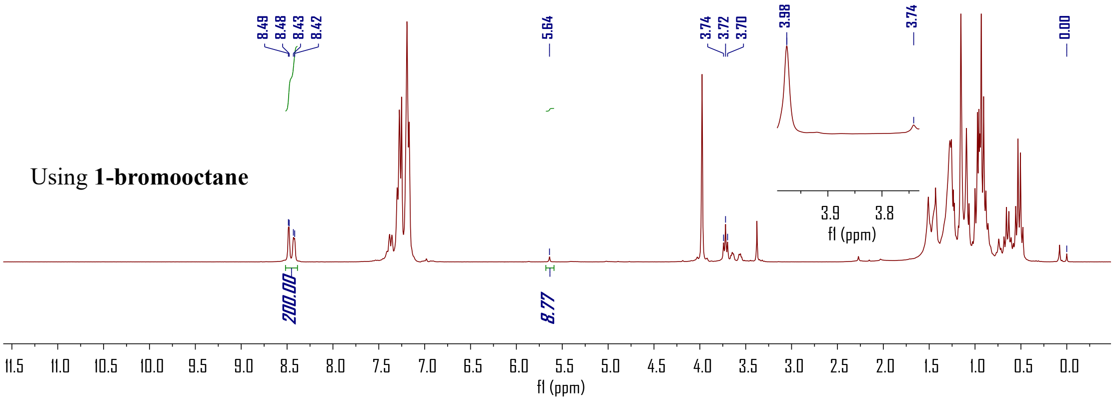

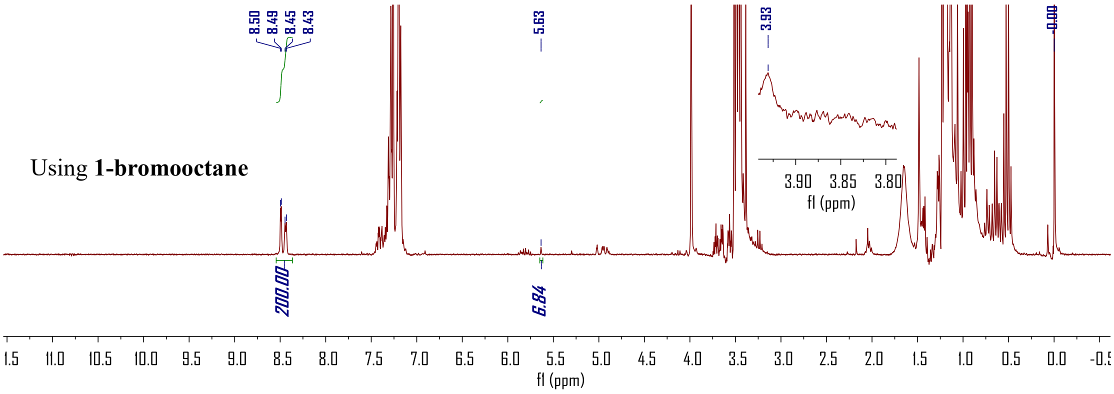

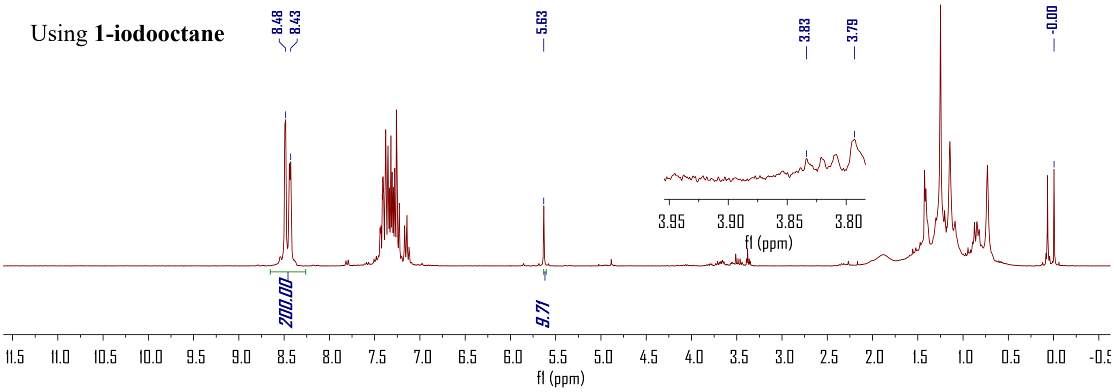

5.3. S_N2 comparative experiments

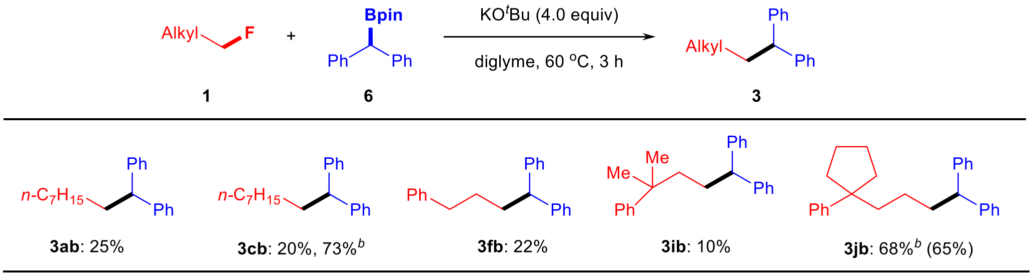

[a]Unless otherwise noted, reactions were conducted with **1** (0.2 mmol, 1.0 equiv.), **6** (2.0 equiv.), KO^{t}Bu (89.6 mg, 4.0 equiv.), and diglyme (2.0 mL) under 60 °C for 3 h, with ^{1}H NMR yields shown that determined by ^{19}F and ^{1}H NMR spectroscopy using 3-fluoropyridine (8.6 µL, 0.1 mmol) as an internal standard. [b]Reaction was performed for 12 hours. Number in parentheses refer to isolated yield.

In an oven-dried tube, charging KO*t*Bu (22.5 mg, 0.2 mmol), anhydrous diglyme (0.5 mL), **6** (58.8 mg, 0.2 mmol), alkyl fluorides **1** (0.1 mmol), and then anhydrous diglyme (0.5 mL) sequentially. The tubes were sealed and move out of glovebox and stirred at 60 ^{o}C for indicted hours. The reaction mixture was then quenched by adding *n*-hexane (5.0 mL) and saturated NH_4Cl (2.0 mL) while stirring for 5 min, then the reaction mixture was extracted with Et_2O, washed by water, dried over Na_2SO_4, and concentrated under vacuum, followed by 3-fluoropyridine (8.6 μL, 0.1 mmol) as an internal standard. Then the ^{1}H NMR analysis of the crude were conducted to show the details of the comparative reactions.

6. Computational details

All density functional theory (DFT) calculations were carried out using the Gaussian16 package.[24] All the structures were optimized using the long-range corrected hybrid ωB97xD density functional[25] in combination with the Def2TZVP basis set.[26] The effect of the solvent was mimicked by applying the SMD model using ether as solvent.[27] The nature of the stationary points was confirmed by frequency calculations analysis at the same level of theory (minima were characterized by no imaginary frequencies, whereas transition states had one imaginary frequency). Transition states were further verified by relaxing the imaginary frequency towards the reactant and the product and by means of IRC calculations.[28] 3D structures of optimized stationary points were represented using the CYLview 1.0 program.[29]

Energies and cartesian coordinates of stationary points

$Me_3SiBpin$

E(electronic) = -820.602948136
Zero-point correction= 0.293748 (Hartree/Particle)
Thermal correction to Energy= 0.311242
Thermal correction to Enthalpy= 0.312186
Thermal correction to Gibbs Free Energy= 0.248716
Sum of electronic and zero-point Energies= -820.309200
Sum of electronic and thermal Energies= -820.291707
Sum of electronic and thermal Enthalpies= -820.290762
Sum of electronic and thermal Free Energies= -820.354232

8	-0.434966000	1.075699000	0.375515000
8	-0.430357000	-1.053796000	-0.422132000
6	-1.804206000	-0.780594000	-0.054202000
6	-1.815110000	0.780115000	0.050553000
6	-2.113064000	1.470365000	-1.275213000
1	-3.157321000	1.342101000	-1.563601000
1	-1.915522000	2.538060000	-1.170654000
1	-1.480727000	1.085244000	-2.076866000
6	-2.709855000	1.337618000	1.140346000
1	-2.651765000	2.427177000	1.141612000
1	-3.749320000	1.054724000	0.961200000
1	-2.416766000	0.980166000	2.126274000
6	-2.723809000	-1.353100000	-1.115106000
1	-2.467137000	-0.992070000	-2.109835000
1	-2.648689000	-2.441613000	-1.117760000
1	-3.761486000	-1.086347000	-0.903797000
6	-2.048518000	-1.474938000	1.280410000
1	-3.084822000	-1.363163000	1.602443000
1	-1.837629000	-2.539427000	1.169259000
1	-1.396700000	-1.079669000	2.061341000
5	0.332787000	0.015535000	-0.030522000
14	2.360647000	0.005876000	-0.008634000
6	2.946448000	-0.617517000	1.666221000
1	2.581820000	-1.629528000	1.861180000
1	4.039259000	-0.640560000	1.710983000
1	2.591805000	0.027137000	2.474789000
6	3.014097000	-1.136477000	-1.347949000
1	4.107133000	-1.174692000	-1.329548000
1	2.639771000	-2.154476000	-1.212662000
1	2.707408000	-0.799830000	-2.341778000
6	3.024136000	1.740806000	-0.281851000
1	2.655978000	2.427023000	0.485212000
1	4.117357000	1.752197000	-0.246542000
1	2.716219000	2.134722000	-1.254078000

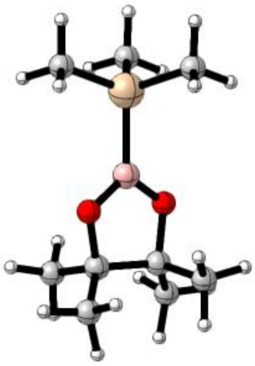

KO^{t}Bu

E(electronic) = -833.058815221

Zero-point correction=	0.123412 (Hartree/Particle)
Thermal correction to Energy=	0.131736
Thermal correction to Enthalpy=	0.132680
Thermal correction to Gibbs Free Energy=	0.089354
Sum of electronic and zero-point Energies=	-832.935403
Sum of electronic and thermal Energies=	-832.927079
Sum of electronic and thermal Enthalpies=	-832.926135
Sum of electronic and thermal Free Energies=	-832.969462

8	-0.282241000	-0.000182000	0.000115000
6	1.083310000	-0.000092000	0.000020000
6	1.626898000	-1.435751000	0.146294000
1	1.261518000	-1.873362000	1.079877000
1	1.261644000	-2.052523000	-0.680048000
1	2.721569000	-1.481086000	0.151002000
6	1.627389000	0.591077000	-1.316232000
1	2.722058000	0.609674000	-1.357286000
1	1.262535000	0.001488000	-2.162324000
1	1.262384000	1.615128000	-1.437448000
6	1.627439000	0.844509000	1.169912000
1	2.722122000	0.870653000	1.206211000
1	1.262839000	1.872108000	1.081717000
1	1.262632000	0.438264000	2.117781000
19	-2.593241000	0.000140000	-0.000019000

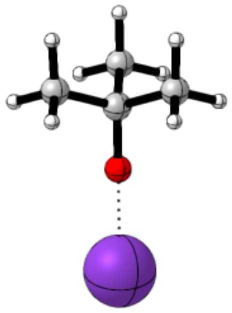

TSI (with K)

E(electronic) = -1653.67989616

Zero-point correction=	0.419289 (Hartree/Particle)
Thermal correction to Energy=	0.445347
Thermal correction to Enthalpy=	0.446291
Thermal correction to Gibbs Free Energy=	0.364801
Sum of electronic and zero-point Energies=	-1653.260607
Sum of electronic and thermal Energies=	-1653.234549
Sum of electronic and thermal Enthalpies=	-1653.233605
Sum of electronic and thermal Free Energies=	-1653.315095

Frequency 30.7092

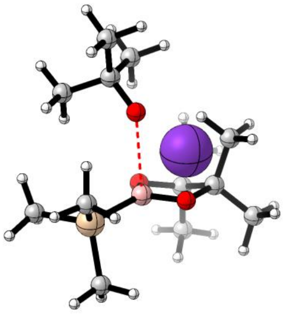

8	-1.129103000	0.077526000	-1.295153000
8	-1.240776000	0.250863000	0.974261000
6	-2.130494000	-0.841724000	0.631267000
6	-2.314986000	-0.647135000	-0.914138000
6	-2.385979000	-1.937232000	-1.708619000
1	-3.256736000	-2.524401000	-1.408519000
1	-2.483951000	-1.705712000	-2.770396000
1	-1.492596000	-2.543620000	-1.573388000
6	-3.499720000	0.245536000	-1.270762000
1	-3.462163000	0.467768000	-2.338104000
1	-4.451209000	-0.243367000	-1.056252000
1	-3.462169000	1.191801000	-0.728220000
6	-1.424482000	-2.141594000	0.997254000
1	-0.435455000	-2.181910000	0.536457000
1	-1.311954000	-2.195034000	2.084496000
1	-2.011212000	-3.011583000	0.701022000
6	-3.405320000	-0.696890000	1.441971000
1	-4.126022000	-1.466578000	1.157631000
1	-3.182263000	-0.823300000	2.503080000
1	-3.867300000	0.279643000	1.306747000
5	-0.598595000	0.674709000	-0.182703000
14	0.483982000	2.387226000	-0.226818000
6	1.158465000	2.791789000	-1.930679000
1	0.392362000	2.652710000	-2.697839000
1	1.478812000	3.837615000	-1.964085000
1	2.014397000	2.170763000	-2.196050000
6	-0.732665000	3.758780000	0.225447000
1	-0.237976000	4.734585000	0.244068000
1	-1.549387000	3.815014000	-0.499982000
1	-1.177285000	3.589441000	1.210106000
6	1.890960000	2.441843000	1.023520000
1	2.524740000	1.558304000	0.913005000
1	2.508297000	3.329935000	0.860575000
1	1.523169000	2.507703000	2.053230000
8	1.599497000	-0.927676000	0.337109000
6	2.376888000	-1.517648000	-0.622455000
6	2.776241000	-0.501559000	-1.706851000
1	1.875551000	-0.112040000	-2.186179000
1	3.309252000	0.336874000	-1.249706000
1	3.418641000	-0.935846000	-2.480704000
6	3.663282000	-2.088719000	0.006639000
1	4.318480000	-2.575235000	-0.724427000

1	4.226908000	-1.283094000	0.486932000
1	3.402711000	-2.824555000	0.773617000
6	1.620526000	-2.669380000	-1.313027000
1	2.217208000	-3.165365000	-2.086226000
1	1.327238000	-3.421563000	-0.574474000
1	0.714296000	-2.275360000	-1.778640000
19	0.951559000	-0.334759000	2.541266000

TSI (without K)

E(electronic) = -1053.76772460
Zero-point correction= 0.415611 (Hartree/Particle)
Thermal correction to Energy= 0.439493
Thermal correction to Enthalpy= 0.440437
Thermal correction to Gibbs Free Energy= 0.364670
Sum of electronic and zero-point Energies= -1053.352113
Sum of electronic and thermal Energies= -1053.328232
Sum of electronic and thermal Enthalpies= -1053.327287
Sum of electronic and thermal Free Energies= -1053.40305

Frequency -128.8247

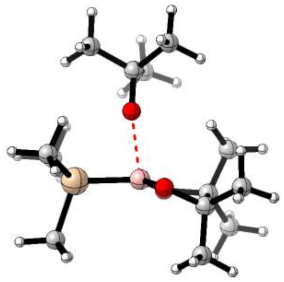

8	1.038534000	0.050544000	1.061785000
8	1.167598000	0.011880000	-1.219557000
6	2.323997000	-0.675470000	-0.742184000
6	1.952085000	-1.001488000	0.744222000
6	1.200627000	-2.319837000	0.894182000
1	1.827372000	-3.183785000	0.664037000
1	0.856653000	-2.408346000	1.926648000
1	0.323032000	-2.310027000	0.250001000
6	3.118100000	-0.959076000	1.717203000
1	2.760698000	-1.180200000	2.725038000
1	3.869366000	-1.708394000	1.454255000
1	3.595643000	0.019948000	1.736540000
6	2.575327000	-1.897748000	-1.610163000
1	1.679742000	-2.512299000	-1.691764000
1	2.861720000	-1.582926000	-2.615908000
1	3.386721000	-2.507443000	-1.203775000
6	3.502604000	0.290545000	-0.860293000
1	4.447896000	-0.178131000	-0.577496000
1	3.581239000	0.621749000	-1.897448000
1	3.348158000	1.172962000	-0.236703000
5	0.429799000	0.473493000	-0.125245000
14	-0.698994000	2.134754000	-0.189424000
6	0.413380000	3.681543000	-0.089004000
1	1.130178000	3.706654000	-0.916425000

1	-0.168128000	4.609281000	-0.127864000
1	0.989319000	3.691872000	0.842327000
6	-1.686571000	2.299939000	-1.781214000
1	-2.383152000	3.143829000	-1.759647000
1	-1.037919000	2.413256000	-2.654545000
1	-2.224086000	1.360386000	-1.873153000
6	-1.855124000	2.298286000	1.289512000
1	-1.294199000	2.454696000	2.215853000
1	-2.543718000	3.140991000	1.169994000
1	-2.434224000	1.386515000	1.397762000
8	-1.400029000	-0.547133000	-0.490828000
6	-2.433195000	-1.347630000	-0.081377000
6	-2.403736000	-1.632516000	1.437304000
1	-1.544147000	-2.258502000	1.691232000
1	-2.313109000	-0.691228000	1.985163000
1	-3.305623000	-2.148901000	1.787005000
6	-3.796255000	-0.688126000	-0.407362000
1	-4.653957000	-1.347272000	-0.227097000
1	-3.934310000	0.204855000	0.208193000
1	-3.805838000	-0.400253000	-1.462975000
6	-2.404279000	-2.700331000	-0.832706000
1	-3.215345000	-3.375989000	-0.534489000
1	-2.481739000	-2.520337000	-1.908952000
1	-1.453927000	-3.208241000	-0.649307000

A (with K^+)

E(electronic) = -1653.70867050

Zero-point correction= 0.419939 (Hartree/Particle)
Thermal correction to Energy= 0.445990
Thermal correction to Enthalpy= 0.446935
Thermal correction to Gibbs Free Energy= 0.366588
Sum of electronic and zero-point Energies= -1653.288731
Sum of electronic and thermal Energies= -1653.262680
Sum of electronic and thermal Enthalpies= -1653.261736
Sum of electronic and thermal Free Energies= -1653.342082

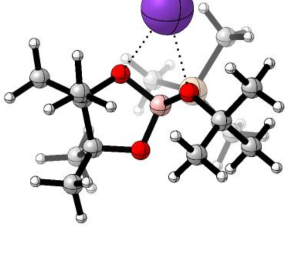

8	-0.856495000	-0.066996000	-1.309566000
8	-0.887481000	0.262738000	1.003162000
6	-2.145723000	-0.281314000	0.637225000
6	-2.206876000	0.001996000	-0.903750000
6	-3.016712000	-1.020008000	-1.690967000
1	-4.053625000	-1.053965000	-1.346073000
1	-3.022809000	-0.744611000	-2.747812000
1	-2.588358000	-2.018293000	-1.606964000
6	-2.740917000	1.402470000	-1.212473000

1	-2.559842000	1.617814000	-2.267340000
1	-3.813975000	1.486272000	-1.025608000
1	-2.224175000	2.156629000	-0.619802000
6	-2.162686000	-1.778955000	0.958592000
1	-1.392682000	-2.307059000	0.398617000
1	-1.972013000	-1.916911000	2.027475000
1	-3.132260000	-2.233160000	0.744481000
6	-3.237189000	0.405846000	1.444737000
1	-4.229171000	0.088209000	1.113544000
1	-3.139000000	0.143444000	2.501219000
1	-3.172938000	1.489786000	1.360109000
5	0.032699000	0.167507000	-0.176852000
14	1.106582000	1.957293000	-0.184592000
6	1.983348000	2.500046000	-1.775913000
1	1.300641000	2.449648000	-2.629497000
1	2.322969000	3.537279000	-1.686702000
1	2.854875000	1.888320000	-2.016651000
6	0.033166000	3.453812000	0.270587000
1	0.656148000	4.319927000	0.518111000
1	-0.613957000	3.748515000	-0.560097000
1	-0.606032000	3.240758000	1.131581000
6	2.453933000	1.920576000	1.167608000
1	3.079331000	1.024743000	1.094755000
1	3.119139000	2.786781000	1.095702000
1	2.013973000	1.959837000	2.171730000
8	0.926265000	-1.015467000	0.114699000
6	1.623920000	-1.809203000	-0.819811000
6	2.318846000	-0.960305000	-1.882046000
1	1.592142000	-0.376633000	-2.448423000
1	3.029355000	-0.274703000	-1.417307000
1	2.867965000	-1.596127000	-2.580620000
6	2.675481000	-2.577000000	-0.018926000
1	3.263178000	-3.237456000	-0.660472000
1	3.362371000	-1.882050000	0.472194000
1	2.194278000	-3.195270000	0.744922000
6	0.675415000	-2.803347000	-1.495388000
1	1.218250000	-3.442942000	-2.196582000
1	0.202722000	-3.445499000	-0.748055000
1	-0.104173000	-2.265335000	-2.032810000
19	0.889984000	-0.755657000	2.639037000

A’ (ionic par) (with K^+)

E(electronic) = -1653.67749675

Zero-point correction= 0.420238 (Hartree/Particle)

Thermal correction to Energy= 0.447480

Thermal correction to Enthalpy= 0.448424

Thermal correction to Gibbs Free Energy= 0.362083

Sum of electronic and zero-point Energies= -1653.257259

Sum of electronic and thermal Energies= -1653.230017

Sum of electronic and thermal Enthalpies= -1653.229073

Sum of electronic and thermal Free Energies= -1653.315414

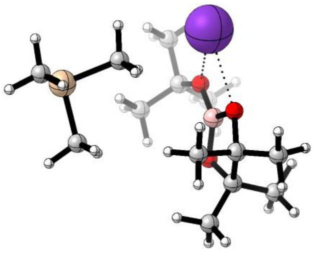

8	1.737118000	0.072321000	-1.076902000
8	1.372034000	-0.355996000	1.146479000
6	1.840342000	-1.605668000	0.575034000
6	2.496483000	-1.123474000	-0.767550000
6	3.948567000	-0.695400000	-0.605267000
1	4.596363000	-1.555034000	-0.430295000
1	4.275504000	-0.201553000	-1.521049000
1	4.071088000	0.006125000	0.222031000
6	2.356744000	-2.099238000	-1.917728000
1	2.840838000	-1.690650000	-2.805747000
1	2.842239000	-3.045783000	-1.672447000
1	1.312281000	-2.292600000	-2.156894000
6	2.799618000	-2.263222000	1.545036000
1	3.603230000	-1.590093000	1.839926000
1	2.262724000	-2.574028000	2.442855000
1	3.239099000	-3.154138000	1.092776000
6	0.612406000	-2.478216000	0.360752000
1	0.885905000	-3.457791000	-0.032144000
1	0.107422000	-2.627738000	1.316826000
1	-0.095244000	-2.014862000	-0.327657000
5	1.197338000	0.517574000	0.089068000
14	-3.798285000	-0.516118000	-0.776917000
6	-2.581340000	-1.615380000	-1.810334000
1	-1.708334000	-1.041263000	-2.144032000
1	-2.215017000	-2.482549000	-1.243312000
1	-3.065793000	-2.000332000	-2.714730000
6	-2.620500000	-0.215903000	0.750284000
1	-2.213693000	-1.154708000	1.154501000
1	-1.793210000	0.413514000	0.405963000
1	-3.160941000	0.311796000	1.546172000
6	-4.883726000	-1.925154000	-0.011215000
1	-5.527850000	-2.385156000	-0.769249000
1	-4.276802000	-2.727305000	0.432786000
1	-5.548121000	-1.541577000	0.771816000
8	0.514577000	1.659464000	0.338280000

6	0.223579000	2.720009000	-0.612926000
6	-0.504748000	2.150142000	-1.821193000
1	0.122488000	1.446686000	-2.368109000
1	-1.425116000	1.645185000	-1.521068000
1	-0.776000000	2.962784000	-2.496966000
6	-0.676005000	3.679855000	0.148725000
1	-0.930503000	4.535954000	-0.477163000
1	-1.608002000	3.185442000	0.432668000
1	-0.174244000	4.050093000	1.045867000
6	1.532787000	3.387144000	-1.007288000
1	1.334831000	4.231974000	-1.668793000
1	2.056629000	3.757883000	-0.123799000
1	2.183017000	2.687565000	-1.534787000
19	-0.541083000	0.760035000	2.711301000

A' (ionic par) (without K^+)

E(electronic) = -1053.78848685

Zero-point correction= 0.417776 (Hartree/Particle)
Thermal correction to Energy= 0.441587
Thermal correction to Enthalpy= 0.442531
Thermal correction to Gibbs Free Energy= 0.363823
Sum of electronic and zero-point Energies= -1053.370711
Sum of electronic and thermal Energies= -1053.346900
Sum of electronic and thermal Enthalpies= -1053.345956
Sum of electronic and thermal Free Energies= -1053.424664

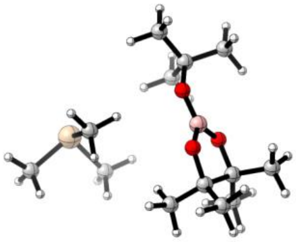

8	-1.642139000	0.218539000	0.781555000
8	-1.355460000	-0.434174000	-1.398341000
6	-1.776958000	-1.624260000	-0.681698000
6	-2.389668000	-1.014435000	0.629127000
6	-3.854969000	-0.627775000	0.482432000
1	-4.492319000	-1.510730000	0.424115000
1	-4.155587000	-0.045960000	1.354526000
1	-4.023076000	-0.018485000	-0.407555000
6	-2.186514000	-1.862429000	1.867749000
1	-2.643570000	-1.370685000	2.727492000
1	-2.662766000	-2.836690000	1.741989000
1	-1.130006000	-2.013848000	2.083049000
6	-2.760280000	-2.394076000	-1.538408000
1	-3.587611000	-1.767736000	-1.868677000
1	-2.252747000	-2.788807000	-2.420186000
1	-3.164547000	-3.239428000	-0.978621000
6	-0.524975000	-2.450880000	-0.428078000
1	-0.764007000	-3.388077000	0.075173000
1	-0.054705000	-2.691801000	-1.383194000

1	0.199779000	-1.907427000	0.179180000
5	-1.156763000	0.547663000	-0.445306000
14	3.887785000	-0.313851000	0.319561000
6	2.733388000	-1.317468000	1.510107000
1	1.863056000	-0.724955000	1.817606000
1	2.362165000	-2.244607000	1.051528000
1	3.259955000	-1.598400000	2.429139000
6	2.645990000	-0.192799000	-1.181419000
1	2.242031000	-1.174776000	-1.469127000
1	1.820675000	0.456510000	-0.870962000
1	3.144804000	0.256993000	-2.049090000
6	4.969727000	-1.778531000	-0.339211000
1	5.651583000	-2.146961000	0.435673000
1	4.361650000	-2.631816000	-0.672507000
1	5.595665000	-1.468828000	-1.184181000
8	-0.506415000	1.667501000	-0.839251000
6	-0.199267000	2.825937000	-0.015895000
6	0.586323000	2.396584000	1.214398000
1	-0.005472000	1.744682000	1.856421000
1	1.503938000	1.877072000	0.931158000
1	0.867843000	3.279420000	1.790575000
6	0.651320000	3.714494000	-0.908999000
1	0.913323000	4.635078000	-0.386227000
1	1.580977000	3.207335000	-1.177725000
1	0.108027000	3.981289000	-1.818591000
6	-1.504834000	3.510660000	0.359961000
1	-1.297718000	4.422900000	0.921597000
1	-2.069666000	3.779044000	-0.535217000
1	-2.120431000	2.860235000	0.983221000

B (with K^+)

E(electronic) = -1653.62681499

Zero-point correction= 0.421330 (Hartree/Particle)

Thermal correction to Energy= 0.448976

Thermal correction to Enthalpy= 0.449921

Thermal correction to Gibbs Free Energy= 0.360645

Sum of electronic and zero-point Energies= -1653.205485

Sum of electronic and thermal Energies= -1653.177839

Sum of electronic and thermal Enthalpies= -1653.176894

Sum of electronic and thermal Free Energies= -1653.266170

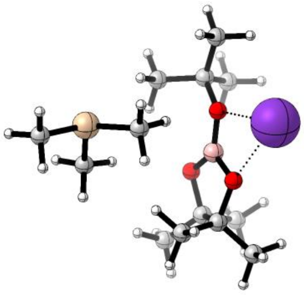

8	1.241167000	-0.294277000	-1.223577000
8	1.385972000	-0.218839000	1.062134000
6	1.720538000	-1.578911000	0.694106000
6	2.048776000	-1.433567000	-0.835320000
6	3.499759000	-1.056209000	-1.101597000
1	4.170767000	-1.887851000	-0.884299000
1	3.609666000	-0.793532000	-2.154480000
1	3.807236000	-0.197058000	-0.502488000
6	1.647982000	-2.625225000	-1.680425000
1	1.913369000	-2.441697000	-2.722566000
1	2.177810000	-3.520290000	-1.348964000
1	0.576556000	-2.813426000	-1.629784000
6	2.881092000	-2.050147000	1.546246000
1	3.723495000	-1.362237000	1.498872000
1	2.564775000	-2.132826000	2.586968000
1	3.215088000	-3.035626000	1.215727000
6	0.487228000	-2.428347000	0.964895000
1	0.678447000	-3.481450000	0.756147000
1	0.215402000	-2.334735000	2.017361000
1	-0.359432000	-2.102812000	0.360453000
5	1.006473000	0.430699000	-0.092000000
14	-3.481078000	-0.701019000	-0.358837000
6	-2.358801000	-1.665534000	-1.515771000
1	-1.435570000	-1.120597000	-1.725046000
1	-2.096239000	-2.629083000	-1.064838000
1	-2.852052000	-1.873326000	-2.468176000
6	-2.510958000	-0.038229000	1.109734000
1	-2.096756000	-0.864202000	1.697816000
1	-1.690372000	0.591084000	0.759825000
1	-3.149552000	0.557586000	1.765681000
6	-4.900324000	-1.778452000	0.226960000
1	-5.480698000	-2.168470000	-0.612125000
1	-4.508583000	-2.632931000	0.790829000
1	-5.578771000	-1.227840000	0.882170000
8	0.447978000	1.657655000	0.027115000
6	0.141988000	2.573309000	-1.054661000
6	-0.858105000	1.933986000	-2.007492000
1	-0.440248000	1.047802000	-2.484650000
1	-1.771379000	1.654278000	-1.477767000
1	-1.128222000	2.647338000	-2.787869000
6	-0.468419000	3.787232000	-0.373671000
1	-0.732228000	4.543237000	-1.114669000
1	-1.375703000	3.508246000	0.166397000
1	0.237366000	4.230643000	0.331345000

6	1.435472000	2.941109000	-1.767996000
1	1.237186000	3.690694000	-2.535790000
1	2.155643000	3.356922000	-1.060786000
1	1.878734000	2.068677000	-2.250395000
19	0.265194000	1.565360000	2.911757000

B (without K^+)

E(electronic) = - -1053.68994778

Zero-point correction= 0.416812 (Hartree/Particle)

Thermal correction to Energy= 0.441917

Thermal correction to Enthalpy= 0.442862

Thermal correction to Gibbs Free Energy= 0.361797

Sum of electronic and zero-point Energies= -1053.273136

Sum of electronic and thermal Energies= -1053.248030

Sum of electronic and thermal Enthalpies= -1053.247086

Sum of electronic and thermal Free Energies= -1053.328150

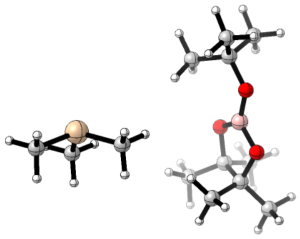

8	-1.298518000	0.236649000	0.983816000
8	-1.825132000	0.385493000	-1.276935000
6	-2.240643000	-0.882804000	-0.816426000
6	-2.339600000	-0.678372000	0.733100000
6	-3.676004000	-0.054624000	1.151537000
1	-4.509781000	-0.753023000	1.041416000
1	-3.611292000	0.237969000	2.201921000
1	-3.880816000	0.842975000	0.566582000
6	-2.113326000	-1.942983000	1.550406000
1	-2.203425000	-1.713573000	2.615020000
1	-2.856640000	-2.707613000	1.306167000
1	-1.119933000	-2.355872000	1.381877000
6	-3.552854000	-1.265384000	-1.487655000
1	-4.299405000	-0.480544000	-1.367940000
1	-3.391461000	-1.414344000	-2.558276000
1	-3.953766000	-2.195896000	-1.075142000
6	-1.165595000	-1.907718000	-1.189392000
1	-1.463666000	-2.932603000	-0.950211000
1	-0.980294000	-1.845763000	-2.263785000
1	-0.232366000	-1.682542000	-0.674496000
5	-1.179478000	1.111825000	-0.190206000
14	3.081083000	-1.204131000	0.145995000
6	1.709198000	-1.526485000	1.381051000
1	0.899772000	-0.798448000	1.271013000
1	1.291943000	-2.526179000	1.209521000
1	2.071365000	-1.495351000	2.411812000
6	2.389282000	-0.923780000	-1.572961000
1	1.975999000	-1.855897000	-1.974803000

1	1.589920000	-0.178834000	-1.528953000
1	3.158487000	-0.574224000	-2.266483000
6	4.299593000	-2.641524000	0.139287000
1	4.725136000	-2.815046000	1.130652000
1	3.786899000	-3.560285000	-0.170056000
1	5.123629000	-2.469390000	-0.557345000
8	0.170438000	1.505053000	-0.515169000
6	0.814639000	2.631398000	0.031572000
6	0.812023000	2.581101000	1.561172000
1	-0.212167000	2.599665000	1.937796000
1	1.282963000	1.657717000	1.907305000
1	1.359605000	3.430460000	1.980926000
6	2.255270000	2.590565000	-0.474316000
1	2.815878000	3.466475000	-0.136399000
1	2.765968000	1.695992000	-0.106562000
1	2.271539000	2.567186000	-1.566437000
6	0.149139000	3.924090000	-0.451124000
1	0.668936000	4.804089000	-0.060206000
1	0.161479000	3.966667000	-1.543341000
1	-0.893324000	3.946511000	-0.125669000

2

E(electronic) = -502.630678843

Zero-point correction= 0.211230 (Hartree/Particle)

Thermal correction to Energy= 0.220567

Thermal correction to Enthalpy= 0.221512

Thermal correction to Gibbs Free Energy= 0.175418

Sum of electronic and zero-point Energies= -502.419449

Sum of electronic and thermal Energies= -502.410111

Sum of electronic and thermal Enthalpies= -502.409167

Sum of electronic and thermal Free Energies= -502.455261

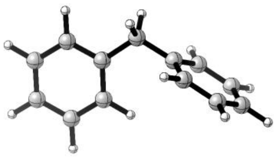

6	0.056900000	-1.421790000	-0.001701000
1	0.108551000	-2.077533000	0.871530000
1	0.108185000	-2.075276000	-0.876658000
6	-1.261274000	-0.696369000	-0.000542000
6	-1.875484000	-0.338856000	1.196026000
6	-1.878060000	-0.339567000	-1.195970000
6	-3.075050000	0.357685000	1.199990000
6	-3.077654000	0.356977000	-1.197762000
6	-3.680112000	0.708571000	0.001656000
1	-1.405879000	-0.607781000	2.135854000
1	-1.410497000	-0.609074000	-2.136648000
1	-3.539456000	0.625281000	2.141482000
1	-3.544094000	0.624013000	-2.138407000

1	-4.617492000	1.251188000	0.002508000
6	1.289460000	-0.535574000	-0.000813000
6	1.224224000	0.852466000	-0.002468000
6	2.546892000	-1.139054000	0.001421000
6	2.384109000	1.618425000	-0.001937000
6	3.704317000	-0.379329000	0.001945000
6	3.627156000	1.007830000	0.000247000
1	0.261260000	1.347846000	-0.004158000
1	2.615755000	-2.221786000	0.002778000
1	2.310288000	2.699364000	-0.003235000
1	4.670278000	-0.870008000	0.003722000
1	4.530559000	1.605276000	0.000680000

Me_3SiH

E(electronic) = -409.885207135

Zero-point correction= 0.118947 (Hartree/Particle)

Thermal correction to Energy= 0.126362

Thermal correction to Enthalpy= 0.127306

Thermal correction to Gibbs Free Energy= 0.088826

Sum of electronic and zero-point Energies= -409.766260

Sum of electronic and thermal Energies= -409.758845

Sum of electronic and thermal Enthalpies= -409.757901

Sum of electronic and thermal Free Energies= -409.796381

14	0.000141000	-0.000109000	0.374741000
6	1.737707000	-0.356069000	-0.220684000
1	2.087208000	-1.329330000	0.133162000
1	1.776077000	-0.364189000	-1.313719000
1	2.441388000	0.401875000	0.132829000
6	-1.177419000	-1.326499000	-0.220621000
1	-1.202954000	-1.356030000	-1.313657000
1	-0.874224000	-2.315103000	0.133416000
1	-2.195053000	-1.141142000	0.132522000
6	-0.560460000	1.682682000	-0.220537000
1	0.107494000	2.472035000	0.133530000
1	-0.572332000	1.719931000	-1.313591000
1	-1.568850000	1.913030000	0.132631000
1	0.000306000	-0.000229000	1.867562000

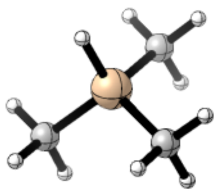

C (with K^+)

E(electronic) = -1746.39348310

Zero-point correction= 0.504330 (Hartree/Particle)

Thermal correction to Energy= 0.535010

Thermal correction to Enthalpy= 0.535954

Thermal correction to Gibbs Free Energy= 0.440557

Sum of electronic and zero-point Energies= -1745.889153
Sum of electronic and thermal Energies= -1745.858473
Sum of electronic and thermal Enthalpies= -1745.857529
Sum of electronic and thermal Free Energies= -1745.952926

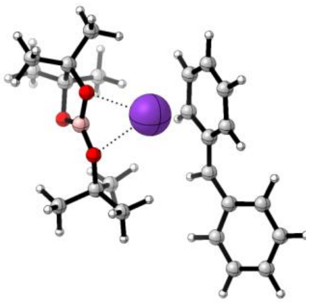

8	-3.021203000	1.501763000	0.547495000
8	-2.656286000	-0.261622000	-0.870248000
6	-3.963560000	-0.483320000	-0.290424000
6	-3.914942000	0.441613000	0.974532000
6	-5.246530000	1.046156000	1.367005000
1	-5.964838000	0.257574000	1.599486000
1	-5.122127000	1.663308000	2.257899000
1	-5.656594000	1.669314000	0.573695000
6	-3.256811000	-0.230122000	2.173129000
1	-3.075459000	0.520150000	2.943695000
1	-3.897433000	-1.005571000	2.594269000
1	-2.298766000	-0.680498000	1.904078000
6	-4.992305000	-0.035455000	-1.319414000
1	-4.897293000	1.028205000	-1.545067000
1	-4.838466000	-0.595257000	-2.242967000
1	-6.007531000	-0.226563000	-0.970109000
6	-4.120981000	-1.959328000	0.012489000
1	-5.048853000	-2.137694000	0.559345000
1	-4.166512000	-2.525328000	-0.919440000
1	-3.287025000	-2.338356000	0.601618000
5	-2.208374000	0.956455000	-0.403249000
8	-1.045519000	1.428272000	-0.908233000
6	-0.299008000	2.575806000	-0.434399000
6	-0.117189000	2.493896000	1.075588000
1	-1.056477000	2.661722000	1.601044000
1	0.266745000	1.511020000	1.354422000
1	0.599687000	3.249385000	1.401518000
6	1.045374000	2.476870000	-1.138896000
1	1.672227000	3.332441000	-0.884026000
1	1.574232000	1.570185000	-0.834212000
1	0.910566000	2.462613000	-2.222220000
6	-1.042295000	3.839167000	-0.840751000
1	-0.479231000	4.721223000	-0.530688000
1	-1.171619000	3.876422000	-1.924041000
1	-2.025002000	3.878929000	-0.367782000
19	-0.191622000	-1.160976000	-1.605393000
6	2.688694000	-0.341710000	0.920721000
1	2.172145000	0.386376000	1.538255000
6	1.911150000	-1.493791000	0.581661000
6	0.671133000	-1.639250000	1.275877000

6	2.251386000	-2.420587000	-0.416439000
6	-0.151605000	-2.801383000	1.035017000
6	1.382176000	-3.540996000	-0.706665000
6	0.227495000	-3.716347000	0.073790000
1	0.427292000	-0.947910000	2.073168000
1	3.141784000	-2.279427000	-1.011465000
1	-1.046596000	-2.963851000	1.623367000
1	1.684201000	-4.294127000	-1.422720000
1	-0.393674000	-4.592128000	-0.093770000
6	4.025288000	0.037765000	0.568211000
6	5.023037000	-0.842446000	0.103643000
6	4.392240000	1.391251000	0.731990000
6	6.290264000	-0.383576000	-0.208018000
6	5.656155000	1.844293000	0.412037000
6	6.616236000	0.959973000	-0.069270000
1	4.811627000	-1.899476000	0.030861000
1	3.652625000	2.087824000	1.111395000
1	7.038673000	-1.086938000	-0.553696000
1	5.899965000	2.892148000	0.541406000
1	7.609866000	1.311416000	-0.318016000

C (without K^+)

E(electronic) = -1146.48680842

Zero-point correction=	0.502057 (Hartree/Particle)
Thermal correction to Energy=	0.527348
Thermal correction to Enthalpy=	0.528292
Thermal correction to Gibbs Free Energy=	0.448219
Sum of electronic and zero-point Energies=	-1145.984752
Sum of electronic and thermal Energies=	-1145.959460
Sum of electronic and thermal Enthalpies=	-1145.958516
Sum of electronic and thermal Free Energies=	-1146.038589

8	0.739683000	-1.871465000	-0.789048000
8	0.530426000	-2.202834000	1.467514000
6	-0.775542000	-2.428827000	0.907946000
6	-0.455059000	-2.648544000	-0.611370000
6	-0.096520000	-4.094542000	-0.944862000
1	-0.971298000	-4.744709000	-0.890398000
1	0.299992000	-4.131951000	-1.960523000
1	0.666896000	-4.477508000	-0.265199000
6	-1.524123000	-2.136611000	-1.557304000
1	-1.224221000	-2.330134000	-2.589035000
1	-2.473204000	-2.645146000	-1.373446000
1	-1.687806000	-1.067549000	-1.438023000
6	-1.407066000	-3.623870000	1.598937000

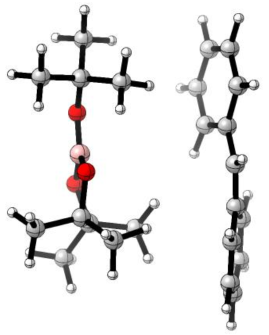

1	-0.758779000	-4.498872000	1.567216000
1	-1.598223000	-3.376594000	2.643861000
1	-2.361525000	-3.871200000	1.129050000
6	-1.603974000	-1.177058000	1.153019000
1	-2.633885000	-1.281718000	0.808895000
1	-1.618360000	-0.963470000	2.222218000
1	-1.177815000	-0.312770000	0.644067000
5	1.313601000	-1.704464000	0.452913000
6	-1.189854000	2.381502000	-1.036266000
8	2.517962000	-1.158082000	0.711738000
6	3.424134000	-0.619873000	-0.262587000
6	2.724132000	0.385279000	-1.169534000
1	1.984012000	-0.099526000	-1.803811000
1	2.225353000	1.148426000	-0.573718000
1	3.460818000	0.881786000	-1.804620000
6	4.499176000	0.084421000	0.555314000
1	5.275495000	0.483454000	-0.100743000
1	4.054562000	0.913257000	1.108195000
1	4.955861000	-0.611812000	1.261603000
6	4.018870000	-1.774267000	-1.065170000
1	4.756710000	-1.399085000	-1.777587000
1	4.509276000	-2.487268000	-0.398726000
1	3.235327000	-2.291956000	-1.620627000
6	-2.405543000	1.824915000	-0.574690000
6	-2.889956000	1.937655000	0.781131000
6	-3.265880000	1.130798000	-1.489149000
6	-4.050019000	1.323852000	1.172100000
6	-4.414836000	0.512206000	-1.084628000
6	-4.824598000	0.561794000	0.274551000
1	-2.343296000	2.549009000	1.486990000
1	-2.968450000	1.077370000	-2.533164000
1	-4.383992000	1.436022000	2.199160000
1	-5.024234000	-0.013789000	-1.812447000
1	-5.737206000	0.078360000	0.598757000
6	-0.032646000	2.773777000	-0.334519000
6	0.948550000	3.599193000	-0.977004000
6	0.295902000	2.328870000	0.998621000
6	2.103160000	3.982157000	-0.354958000
6	1.465592000	2.712478000	1.606194000
6	2.389130000	3.554933000	0.966899000
1	0.754052000	3.927950000	-1.993928000
1	-0.363102000	1.639989000	1.508635000
1	2.815034000	4.606531000	-0.884667000
1	1.688927000	2.332153000	2.597816000

1	3.305508000	3.851464000	1.460985000
1	-1.133166000	2.519229000	-2.119011000

C' (ionic par) (with K^{+})

E(electronic) = -1746.46679296

Zero-point correction= 0.509059 (Hartree/Particle)

Thermal correction to Energy= 0.539113

Thermal correction to Enthalpy= 0.540057

Thermal correction to Gibbs Free Energy= 0.447255

Sum of electronic and zero-point Energies= -1745.957734

Sum of electronic and thermal Energies= -1745.927680

Sum of electronic and thermal Enthalpies= -1745.926736

Sum of electronic and thermal Free Energies= -1746.019538

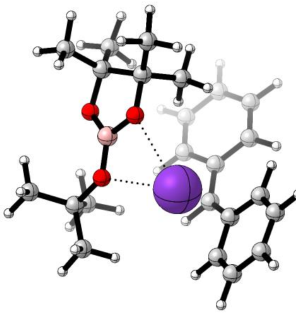

8	-2.557432000	0.755351000	0.350846000
8	-1.220277000	0.799459000	-1.512619000
6	-2.128895000	-0.314965000	-1.708746000
6	-3.261293000	-0.003687000	-0.664413000
6	-4.353115000	0.904541000	-1.213762000
1	-4.967600000	0.383907000	-1.949062000
1	-4.997104000	1.219789000	-0.391801000
1	-3.935307000	1.798518000	-1.680667000
6	-3.868440000	-1.232172000	-0.018664000
1	-4.643128000	-0.929982000	0.687718000
1	-4.330032000	-1.868611000	-0.776202000
1	-3.121001000	-1.814378000	0.518346000
6	-2.587704000	-0.324768000	-3.152572000
1	-2.998917000	0.636965000	-3.455697000
1	-1.744778000	-0.559498000	-3.804679000
1	-3.349104000	-1.092855000	-3.300366000
6	-1.351154000	-1.581788000	-1.387369000
1	-1.953329000	-2.473012000	-1.565138000
1	-0.472196000	-1.635869000	-2.032490000
1	-1.012996000	-1.597108000	-0.350945000
5	-1.445671000	1.274738000	-0.237324000
6	1.976702000	-1.035955000	1.301522000
8	-0.549659000	2.160361000	0.261324000
6	-0.594034000	2.798747000	1.561439000
6	-0.610102000	1.732681000	2.646508000
1	-1.537415000	1.160404000	2.626988000
1	0.225886000	1.048206000	2.496785000
1	-0.510414000	2.198185000	3.628339000
6	0.688892000	3.612477000	1.631963000
1	0.740905000	4.157862000	2.575156000
1	1.561897000	2.957295000	1.573421000

1	0.732061000	4.336111000	0.815172000
6	-1.818413000	3.699005000	1.630263000
1	-1.833863000	4.233354000	2.581653000
1	-1.799799000	4.434572000	0.823574000
1	-2.736350000	3.114717000	1.552916000
19	1.491298000	1.382465000	-1.385270000
6	1.084930000	-2.157843000	1.280624000
6	1.192647000	-3.299552000	0.453514000
6	-0.019298000	-2.159918000	2.171224000
6	0.245637000	-4.312161000	0.476500000
6	-0.953925000	-3.173395000	2.192471000
6	-0.847415000	-4.262465000	1.329119000
1	2.049806000	-3.423001000	-0.191489000
1	-0.138322000	-1.318845000	2.845861000
1	0.376248000	-5.165056000	-0.181306000
1	-1.785174000	-3.112790000	2.886850000
1	-1.584411000	-5.055683000	1.338848000
6	2.919677000	-0.584981000	0.364393000
6	3.675467000	0.601250000	0.661894000
6	3.162939000	-1.116420000	-0.944487000
6	4.514480000	1.203357000	-0.246841000
6	4.013916000	-0.495057000	-1.844727000
6	4.692003000	0.680628000	-1.535026000
1	3.561728000	1.041279000	1.647377000
1	2.647238000	-2.005175000	-1.273633000
1	5.047483000	2.101930000	0.046520000
1	4.143380000	-0.943294000	-2.824797000
1	5.363605000	1.147070000	-2.243412000
1	1.872612000	-0.402257000	2.177520000

C' (ionic par) (without K^+)

E(electronic) = -1146.56518201
Zero-point correction= 0.510846 (Hartree/Particle)
Thermal correction to Energy= 0.536612
Thermal correction to Enthalpy= 0.537556
Thermal correction to Gibbs Free Energy= 0.458304
Sum of electronic and zero-point Energies= -1146.054336
Sum of electronic and thermal Energies= -1146.028570
Sum of electronic and thermal Enthalpies= -1146.027626
Sum of electronic and thermal Free Energies= -1146.106878

8	2.032387000	-0.758352000	-0.222091000
8	2.030321000	0.181080000	1.867795000
6	1.330320000	-1.072367000	1.981200000
6	1.814268000	-1.837670000	0.705464000
6	3.161117000	-2.529681000	0.903060000
1	3.066019000	-3.404710000	1.548222000
1	3.531539000	-2.856979000	-0.069283000
1	3.895583000	-1.850578000	1.340241000
6	0.802799000	-2.805541000	0.127728000
1	1.216410000	-3.292128000	-0.756648000
1	0.558145000	-3.579260000	0.859241000
1	-0.116628000	-2.306406000	-0.167351000
6	1.707457000	-1.730653000	3.295248000
1	2.787863000	-1.812909000	3.408504000
1	1.320179000	-1.135538000	4.123229000
1	1.268102000	-2.728743000	3.357454000
6	-0.161745000	-0.766661000	1.942909000
1	-0.759715000	-1.668344000	2.081911000
1	-0.400795000	-0.057375000	2.735642000
1	-0.456562000	-0.316332000	0.992801000
5	2.230766000	0.381432000	0.523311000
6	-2.067082000	0.488077000	-1.145637000
8	2.610493000	1.588742000	0.048909000
6	2.622691000	1.957916000	-1.336011000
6	1.239079000	1.740045000	-1.939060000
1	0.972408000	0.683715000	-1.951865000
1	0.484098000	2.265908000	-1.352637000
1	1.211109000	2.118357000	-2.963548000
6	2.974931000	3.440466000	-1.339855000
1	3.014511000	3.820885000	-2.362532000
1	2.222574000	4.002511000	-0.785450000
1	3.945818000	3.600764000	-0.867036000
6	3.688053000	1.155761000	-2.079333000
1	3.753893000	1.491582000	-3.116516000
1	4.663011000	1.296108000	-1.607192000
1	3.442389000	0.094431000	-2.072185000
6	-2.211943000	-0.916482000	-1.200776000
6	-2.856915000	-1.762635000	-0.253883000
6	-1.667770000	-1.610009000	-2.326005000
6	-2.910242000	-3.137262000	-0.410586000
6	-1.730440000	-2.975189000	-2.469699000
6	-2.345661000	-3.777625000	-1.505498000
1	-3.347267000	-1.335618000	0.606364000
1	-1.171079000	-1.020612000	-3.090305000

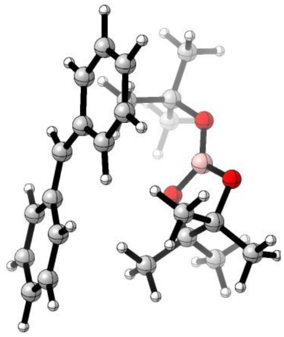

1	-3.415391000	-3.724707000	0.350673000
1	-1.283332000	-3.433211000	-3.346837000
1	-2.389218000	-4.854123000	-1.613061000
6	-2.412285000	1.456342000	-0.168978000
6	-2.228085000	2.834115000	-0.490357000
6	-2.902941000	1.225667000	1.145359000
6	-2.503802000	3.854852000	0.389687000
6	-3.176104000	2.261490000	2.022084000
6	-2.989996000	3.591246000	1.670822000
1	-1.859868000	3.077856000	-1.482192000
1	-3.038255000	0.217760000	1.503998000
1	-2.339496000	4.881037000	0.075086000
1	-3.540838000	2.016054000	3.015166000
1	-3.207811000	4.392747000	2.365580000
1	-1.621050000	0.913726000	-2.040216000

1 (CH_3F)

E(electronic) = -139.764845872

Zero-point correction= 0.039356 (Hartree/Particle)

Thermal correction to Energy= 0.042269

Thermal correction to Enthalpy= 0.043213

Thermal correction to Gibbs Free Energy= 0.017929

Sum of electronic and zero-point Energies= -139.725490

Sum of electronic and thermal Energies= -139.722577

Sum of electronic and thermal Enthalpies= -139.721633

Sum of electronic and thermal Free Energies= -139.746917

6	0.000000000	0.000000000	-0.636971000
1	0.000000000	1.033499000	-0.985776000
1	0.895036000	-0.516749000	-0.985776000
1	-0.895036000	-0.516749000	-0.985776000
9	0.000000000	0.000000000	0.753239000

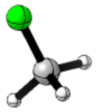

TS-I (with K^+)

E(electronic) = -1886.20806760

Zero-point correction= 0.549782 (Hartree/Particle)

Thermal correction to Energy= 0.583031

Thermal correction to Enthalpy= 0.583975

Thermal correction to Gibbs Free Energy= 0.482423

Sum of electronic and zero-point Energies= -1885.658286

Sum of electronic and thermal Energies= -1885.625037

Sum of electronic and thermal Enthalpies= -1885.624093

Sum of electronic and thermal Free Energies= -1885.725644

Frequency -698.0812

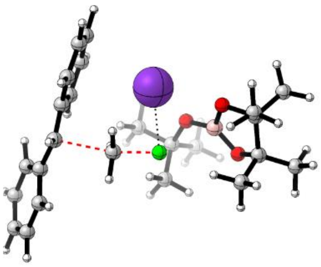

8	-3.678360000	-1.413656000	0.256077000
8	-3.027889000	0.773100000	0.041035000
6	-4.370996000	0.705196000	-0.493452000
6	-4.540002000	-0.832737000	-0.753921000
6	-5.948858000	-1.353486000	-0.558860000
1	-6.633955000	-0.859178000	-1.250387000
1	-5.975715000	-2.424396000	-0.764813000
1	-6.302601000	-1.192111000	0.458247000
6	-3.991714000	-1.272075000	-2.105540000
1	-3.973996000	-2.362030000	-2.141038000
1	-4.619498000	-0.914317000	-2.922461000
1	-2.973139000	-0.912214000	-2.263649000
6	-5.308292000	1.237727000	0.581723000
1	-5.271298000	0.626240000	1.484939000
1	-5.007743000	2.252869000	0.844941000
1	-6.338727000	1.268742000	0.226196000
6	-4.447602000	1.566550000	-1.737840000
1	-5.419460000	1.448023000	-2.220579000
1	-4.334448000	2.617965000	-1.467809000
1	-3.669985000	1.307499000	-2.454940000
5	-2.727026000	-0.477817000	0.542395000
8	-1.565476000	-0.623540000	1.219304000
6	-0.967516000	-1.888823000	1.603810000
6	-0.767978000	-2.750432000	0.364295000
1	-1.723671000	-3.059149000	-0.060078000
1	-0.211815000	-2.198716000	-0.395968000
1	-0.202968000	-3.646791000	0.625455000
6	0.373813000	-1.517380000	2.213650000
1	0.886565000	-2.410835000	2.572476000
1	1.013700000	-1.040478000	1.469370000
1	0.240445000	-0.833465000	3.053940000
6	-1.859684000	-2.571139000	2.630476000
1	-1.389813000	-3.494333000	2.973939000
1	-2.013413000	-1.922236000	3.495077000
1	-2.830264000	-2.821347000	2.201105000
19	-0.464749000	1.663079000	-0.058476000
6	1.672072000	0.216547000	-1.645567000
1	1.594949000	-0.124247000	-0.627044000
1	2.083545000	-0.466275000	-2.372443000
9	-0.018953000	-0.080715000	-1.993106000
1	1.592615000	1.257011000	-1.907759000
6	3.703508000	0.896184000	-0.929042000
1	3.988879000	1.365487000	-1.866496000

6	3.113817000	1.804247000	0.010389000
6	2.615044000	1.458873000	1.291024000
6	2.790367000	3.116503000	-0.423352000
6	1.898398000	2.356340000	2.070358000
6	2.065283000	4.001151000	0.351631000
6	1.608668000	3.637761000	1.618553000
1	2.769585000	0.465886000	1.683827000
1	3.135000000	3.434630000	-1.401720000
1	1.553045000	2.038536000	3.048303000
1	1.860189000	4.994434000	-0.032220000
1	1.058708000	4.336884000	2.235524000
6	4.410493000	-0.348675000	-0.683734000
6	4.843816000	-1.090580000	-1.804496000
6	4.704507000	-0.909214000	0.571900000
6	5.493657000	-2.302315000	-1.682648000
6	5.355281000	-2.130036000	0.691351000
6	5.749704000	-2.845942000	-0.427306000
1	4.656650000	-0.688164000	-2.794817000
1	4.468212000	-0.371732000	1.478334000
1	5.804228000	-2.831917000	-2.576390000
1	5.565883000	-2.517710000	1.682045000
1	6.255323000	-3.798246000	-0.327814000

TS-I (without K^+)

E(electronic) = -1286.30310131

Zero-point correction=	0.548406 (Hartree/Particle)
Thermal correction to Energy=	0.578606
Thermal correction to Enthalpy=	0.579550
Thermal correction to Gibbs Free Energy=	0.487075
Sum of electronic and zero-point Energies=	-1285.754696
Sum of electronic and thermal Energies=	-1285.724495
Sum of electronic and thermal Enthalpies=	-1285.723551
Sum of electronic and thermal Free Energies=	-1285.816026

Frequency -745.5882

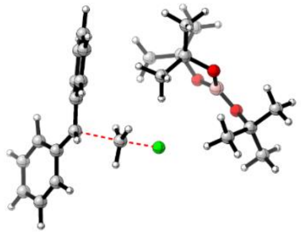

8	-3.364093000	0.546599000	-0.427298000
8	-2.742474000	0.159926000	1.743724000
6	-2.301599000	1.494463000	1.434389000
6	-2.320581000	1.501471000	-0.133735000
6	-2.675255000	2.835817000	-0.758635000
1	-1.935611000	3.589902000	-0.480802000
1	-2.667321000	2.744477000	-1.846194000
1	-3.661885000	3.181408000	-0.451633000

6	-1.037201000	0.954663000	-0.739682000
1	-1.176403000	0.834206000	-1.815825000
1	-0.197005000	1.631368000	-0.580888000
1	-0.787998000	-0.020583000	-0.318654000
6	-3.311062000	2.458814000	2.047914000
1	-4.304168000	2.326185000	1.614154000
1	-3.380257000	2.263456000	3.119229000
1	-3.006859000	3.497429000	1.908225000
6	-0.929701000	1.715504000	2.041104000
1	-0.531288000	2.685205000	1.736411000
1	-1.001734000	1.701538000	3.130321000
1	-0.228400000	0.941425000	1.733596000
5	-3.428051000	-0.307793000	0.650109000
8	-4.098446000	-1.479675000	0.710236000
6	-4.576101000	-2.219738000	-0.426115000
6	-3.425227000	-2.478542000	-1.393427000
1	-3.078334000	-1.550301000	-1.848318000
1	-2.583517000	-2.934887000	-0.868765000
1	-3.749060000	-3.152812000	-2.189133000
6	-5.089075000	-3.533594000	0.146860000
1	-5.487999000	-4.167360000	-0.647474000
1	-4.280416000	-4.070095000	0.646025000
1	-5.882024000	-3.348998000	0.874525000
6	-5.710511000	-1.455974000	-1.100444000
1	-6.125557000	-2.045721000	-1.920624000
1	-6.510125000	-1.254239000	-0.384059000
1	-5.355265000	-0.507530000	-1.503681000
9	0.042128000	-2.196020000	0.093958000
6	3.349211000	-0.281791000	-1.045121000
1	3.344132000	-0.646953000	-2.069627000
6	2.869882000	1.077886000	-0.933981000
6	2.661038000	1.758276000	0.284572000
6	2.443822000	1.761170000	-2.094075000
6	2.123118000	3.034887000	0.326832000
6	1.896212000	3.029095000	-2.047248000
6	1.739818000	3.692071000	-0.833934000
1	2.887496000	1.267921000	1.221213000
1	2.559596000	1.270105000	-3.054795000
1	1.986247000	3.515319000	1.289549000
1	1.589989000	3.509906000	-2.969990000
1	1.318995000	4.689307000	-0.795729000
6	4.381202000	-0.913245000	-0.249827000
6	4.808242000	-2.209650000	-0.621203000
6	5.007203000	-0.371173000	0.890015000

6	5.765008000	-2.907068000	0.089081000
6	5.969852000	-1.074453000	1.599159000
6	6.360542000	-2.349098000	1.216516000
1	4.367150000	-2.665608000	-1.501962000
1	4.768077000	0.631705000	1.213257000
1	6.053348000	-3.899215000	-0.241269000
1	6.427437000	-0.608100000	2.465250000
1	7.109247000	-2.894293000	1.777763000
6	1.533003000	-1.357600000	-0.411282000
1	1.573233000	-0.773812000	0.492078000
1	1.064964000	-0.948903000	-1.290235000
1	2.091475000	-2.276258000	-0.477791000

3

E(electronic) = -541.947484493

Zero-point correction= 0.239746 (Hartree/Particle)

Thermal correction to Energy= 0.251298

Thermal correction to Enthalpy= 0.252242

Thermal correction to Gibbs Free Energy= 0.200446

Sum of electronic and zero-point Energies= -541.707738

Sum of electronic and thermal Energies= -541.696187

Sum of electronic and thermal Enthalpies= -541.695242

Sum of electronic and thermal Free Energies= -541.747038

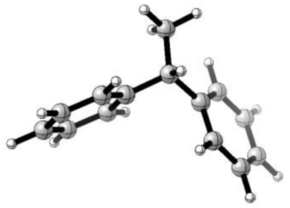

6	0.019143000	1.153714000	-0.626092000
1	0.063545000	1.265104000	-1.712788000
6	-1.258325000	0.388047000	-0.322220000
6	-1.567135000	-0.733537000	-1.091528000
6	-2.126617000	0.739100000	0.704607000
6	-2.704957000	-1.481498000	-0.843407000
6	-3.269601000	-0.009981000	0.959312000
6	-3.563342000	-1.121963000	0.188150000
1	-0.899236000	-1.024239000	-1.895359000
1	-1.920220000	1.606400000	1.318471000
1	-2.924859000	-2.347536000	-1.456058000
1	-3.931924000	0.282252000	1.765431000
1	-4.454728000	-1.704597000	0.385711000
6	1.246292000	0.356015000	-0.216659000
6	1.383852000	-0.128058000	1.082252000
6	2.271999000	0.118891000	-1.124375000
6	2.517571000	-0.827336000	1.462698000
6	3.409902000	-0.582825000	-0.748131000
6	3.536632000	-1.057916000	0.547523000
1	0.589542000	0.036424000	1.801841000
1	2.179885000	0.487483000	-2.140275000

1	2.605537000	-1.198339000	2.476809000
1	4.196921000	-0.759117000	-1.471544000
1	4.421543000	-1.607766000	0.843617000
6	0.070650000	2.556920000	-0.021902000
1	0.106719000	2.526768000	1.068985000
1	0.968915000	3.072921000	-0.364757000
1	-0.798018000	3.146801000	-0.321459000

D (with K^+)

E(electronic) = -1344.36819031

Zero-point correction= 0.311314 (Hartree/Particle)

Thermal correction to Energy= 0.330411

Thermal correction to Enthalpy= 0.331355

Thermal correction to Gibbs Free Energy= 0.265487

Sum of electronic and zero-point Energies= -1344.056877

Sum of electronic and thermal Energies= -1344.037779

Sum of electronic and thermal Enthalpies= -1344.036835

Sum of electronic and thermal Free Energies= -1344.102703

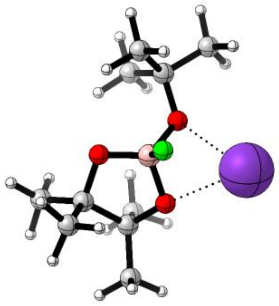

8	0.623169000	-1.068129000	0.693026000
8	0.776497000	1.096200000	-0.151470000
6	1.884456000	0.364573000	-0.646781000
6	1.982828000	-0.814921000	0.380013000
6	2.607867000	-2.082342000	-0.180822000
1	3.625503000	-1.895601000	-0.533062000
1	2.656412000	-2.845038000	0.599296000
1	2.020690000	-2.482307000	-1.006839000
6	2.717599000	-0.407859000	1.658403000
1	2.571659000	-1.187441000	2.408420000
1	3.790704000	-0.289833000	1.492737000
1	2.318932000	0.524632000	2.059677000
6	1.561312000	-0.124545000	-2.059586000
1	0.705318000	-0.799806000	-2.051193000
1	1.308069000	0.736909000	-2.681544000
1	2.410395000	-0.635468000	-2.518118000
6	3.107291000	1.266205000	-0.688759000
1	4.002136000	0.698167000	-0.954449000
1	2.969626000	2.044378000	-1.443345000
1	3.275829000	1.751364000	0.272082000
5	-0.126176000	0.144377000	0.520001000
9	-0.494836000	0.776876000	1.789797000
8	-1.360970000	0.052242000	-0.258220000
6	-2.288287000	-1.016500000	-0.191038000
6	-2.496299000	-1.505146000	1.242627000
1	-1.568182000	-1.901481000	1.655740000

1	-2.835189000	-0.688948000	1.883108000
1	-3.249140000	-2.296609000	1.265046000
6	-3.602462000	-0.467421000	-0.740466000
1	-4.369818000	-1.243650000	-0.780738000
1	-3.973614000	0.341821000	-0.105232000
1	-3.456289000	-0.078493000	-1.751135000
6	-1.800451000	-2.167943000	-1.071846000
1	-2.518754000	-2.991763000	-1.073104000
1	-1.670469000	-1.824253000	-2.100805000
1	-0.842298000	-2.535769000	-0.705023000
19	-1.286306000	2.683069000	-0.058782000

D (without K^+)

E(electronic) = -744.468445175

Zero-point correction= 0.309200 (Hartree/Particle)

Thermal correction to Energy= 0.326323

Thermal correction to Enthalpy= 0.327267

Thermal correction to Gibbs Free Energy= 0.266525

Sum of electronic and zero-point Energies= -744.159246

Sum of electronic and thermal Energies= -744.142122

Sum of electronic and thermal Enthalpies= -744.141178

Sum of electronic and thermal Free Energies= -744.201920

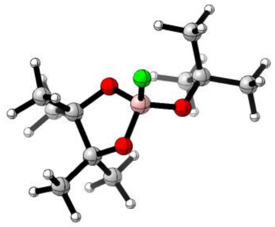

8	0.275476000	0.510699000	0.807589000
8	0.853247000	0.129322000	-1.416642000
6	1.790795000	-0.531018000	-0.605289000
6	1.652199000	0.226214000	0.758440000
6	2.042259000	-0.594900000	1.980473000
1	3.083221000	-0.925314000	1.921417000
1	1.932953000	0.011395000	2.883337000
1	1.404275000	-1.472204000	2.086418000
6	2.442670000	1.539751000	0.766422000
1	2.132446000	2.129493000	1.631951000
1	3.520982000	1.372943000	0.835034000
1	2.233962000	2.122722000	-0.131142000
6	1.399012000	-2.008244000	-0.479320000
1	0.435446000	-2.107919000	0.020946000
1	1.300945000	-2.428658000	-1.482616000
1	2.146078000	-2.591017000	0.066152000
6	3.171870000	-0.436441000	-1.240371000
1	3.942053000	-0.841598000	-0.577741000
1	3.191432000	-1.011497000	-2.169551000
1	3.425994000	0.595684000	-1.480843000
5	-0.236750000	0.619578000	-0.570187000
9	-0.480718000	2.010064000	-0.900892000

8	-1.456392000	-0.133868000	-0.812592000
6	-2.533167000	-0.229930000	0.082326000
6	-2.840159000	1.096911000	0.785850000
1	-1.991889000	1.415123000	1.392755000
1	-3.048546000	1.878575000	0.053334000
1	-3.713054000	0.989731000	1.436317000
6	-3.745841000	-0.646764000	-0.751050000
1	-4.631361000	-0.792497000	-0.125966000
1	-3.969700000	0.118293000	-1.498173000
1	-3.535228000	-1.581670000	-1.275980000
6	-2.248086000	-1.311297000	1.133115000
1	-3.096362000	-1.440810000	1.812203000
1	-2.055475000	-2.267402000	0.639320000
1	-1.365909000	-1.036744000	1.711372000

E (with K^+)

E(electronic) = -1477.57481949

Zero-point correction= 0.435700 (Hartree/Particle)

Thermal correction to Energy= 0.460180

Thermal correction to Enthalpy= 0.461124

Thermal correction to Gibbs Free Energy= 0.384500

Sum of electronic and zero-point Energies= -1477.139119

Sum of electronic and thermal Energies= -1477.114640

Sum of electronic and thermal Enthalpies= -1477.113696

Sum of electronic and thermal Free Energies= -1477.190319

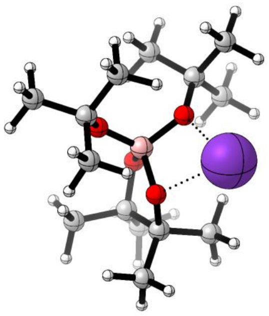

8	-0.861461000	-0.442637000	-1.342101000
8	-0.845397000	0.309187000	0.870603000
6	-2.139715000	-0.188753000	0.581063000
6	-2.190196000	-0.153375000	-0.985092000
6	-3.110737000	-1.195957000	-1.605053000
1	-4.139275000	-1.076757000	-1.254174000
1	-3.110934000	-1.081384000	-2.691135000
1	-2.777706000	-2.208087000	-1.376204000
6	-2.578528000	1.229101000	-1.519086000
1	-2.398151000	1.246315000	-2.595610000
1	-3.633262000	1.454575000	-1.344454000
1	-1.966618000	2.008369000	-1.067243000
6	-2.252107000	-1.619171000	1.120106000
1	-1.500200000	-2.264163000	0.660531000
1	-2.109407000	-1.610803000	2.206031000
1	-3.236707000	-2.054284000	0.941815000
6	-3.180293000	0.684844000	1.265425000
1	-4.193233000	0.381327000	0.988770000
1	-3.088867000	0.595044000	2.351017000

1	-3.048136000	1.733730000	1.003095000
5	0.034681000	0.104153000	-0.328799000
8	1.068209000	-0.888487000	0.094229000
6	1.845976000	-1.671437000	-0.788042000
6	2.247699000	-0.911066000	-2.051617000
1	1.366865000	-0.607107000	-2.616610000
1	2.808116000	-0.010772000	-1.798754000
1	2.870204000	-1.547305000	-2.685939000
6	3.104633000	-2.063798000	-0.014145000
1	3.767150000	-2.685807000	-0.620245000
1	3.656280000	-1.171435000	0.291280000
1	2.844743000	-2.637792000	0.881355000
6	1.065417000	-2.932677000	-1.168433000
1	1.662207000	-3.590835000	-1.805608000
1	0.788956000	-3.489030000	-0.267932000
1	0.153221000	-2.655397000	-1.695713000
19	0.688315000	-1.012434000	2.580515000
8	0.635184000	1.336233000	-0.811094000
6	1.184209000	2.380112000	-0.053646000
6	2.273284000	3.015103000	-0.919212000
1	3.085817000	2.304797000	-1.089572000
1	1.858149000	3.292870000	-1.890033000
1	2.688961000	3.910305000	-0.449252000
6	0.098004000	3.418428000	0.247748000
1	-0.317834000	3.806630000	-0.684624000
1	-0.706793000	2.951217000	0.817729000
1	0.496174000	4.259345000	0.822979000
6	1.810732000	1.923086000	1.270858000
1	1.025021000	1.641286000	1.974821000
1	2.483780000	1.080142000	1.105557000
1	2.377540000	2.739472000	1.723987000

E (without K^+)

E(electronic) = -877.673198076

Zero-point correction= 0.433055 (Hartree/Particle)

Thermal correction to Energy= 0.455908

Thermal correction to Enthalpy= 0.456852

Thermal correction to Gibbs Free Energy= 0.384584

Sum of electronic and zero-point Energies= -877.240143

Sum of electronic and thermal Energies= -877.217290

Sum of electronic and thermal Enthalpies= -877.216346

Sum of electronic and thermal Free Energies= -877.288614

8	0.803477000	0.586882000	-1.011049000
8	0.803475000	-0.586890000	1.011050000
6	2.103749000	-0.117902000	0.774006000
6	2.103750000	0.117888000	-0.774006000
6	3.112020000	1.154532000	-1.254094000
1	4.133970000	0.871568000	-0.985151000
1	3.062548000	1.240901000	-2.342676000
1	2.899454000	2.135984000	-0.830399000
6	2.322049000	-1.187982000	-1.547617000
1	2.103921000	-1.008377000	-2.602801000
1	3.349504000	-1.553741000	-1.467326000
1	1.637680000	-1.958022000	-1.191439000
6	2.322055000	1.187967000	1.547618000
1	1.637690000	1.958011000	1.191439000
1	2.103926000	1.008363000	2.602801000
1	3.349512000	1.553722000	1.467327000
6	3.112017000	-1.154549000	1.254094000
1	4.133968000	-0.871589000	0.985148000
1	3.062547000	-1.240917000	2.342675000
1	2.899445000	-2.136001000	0.830400000
5	-0.107027000	-0.000001000	-0.000001000
8	-0.926945000	1.025874000	0.668223000
6	-1.617362000	2.052728000	0.015778000
6	-2.249589000	1.609629000	-1.308548000
1	-1.482467000	1.283303000	-2.010844000
1	-2.927687000	0.770806000	-1.146762000
1	-2.810909000	2.435938000	-1.755527000
6	-2.726971000	2.498320000	0.971003000
1	-3.295984000	3.340184000	0.565643000
1	-3.416586000	1.671288000	1.157477000
1	-2.295596000	2.800385000	1.928481000
6	-0.675523000	3.237162000	-0.246838000
1	-1.204268000	4.074293000	-0.713730000
1	-0.245927000	3.586067000	0.696005000
1	0.137799000	2.917540000	-0.898128000
8	-0.926945000	-1.025877000	-0.668225000
6	-1.617381000	-2.052717000	-0.015777000
6	-2.727016000	-2.498274000	-0.970988000
1	-3.416618000	-1.671225000	-1.157436000
1	-2.295665000	-2.800332000	-1.928480000
1	-3.296038000	-3.340133000	-0.565630000
6	-0.675563000	-3.237175000	0.246809000
1	-0.245992000	-3.586078000	-0.696045000
1	0.137777000	-2.917578000	0.898089000

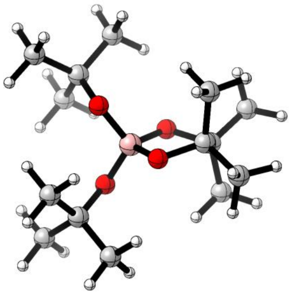

1	-1.204316000	-4.074300000	0.713703000
6	-2.249576000	-1.609619000	1.308564000
1	-1.482434000	-1.283330000	2.010855000
1	-2.927647000	-0.770770000	1.146800000
1	-2.810916000	-2.435918000	1.755535000

KF

E(electronic) = -699.862085841
Zero-point correction= 0.000778 (Hartree/Particle)
Thermal correction to Energy= 0.003509
Thermal correction to Enthalpy= 0.004453
Thermal correction to Gibbs Free Energy= -0.021513
Sum of electronic and zero-point Energies= -699.861308
Sum of electronic and thermal Energies= -699.858577
Sum of electronic and thermal Enthalpies= -699.857633
Sum of electronic and thermal Free Energies= -699.883599

19	0.000000000	0.000000000	0.740548000
9	0.000000000	0.000000000	-1.563379000

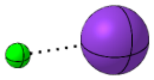

diglyme

E(electronic) = -462.736870984
Zero-point correction= 0.203576 (Hartree/Particle)
Thermal correction to Energy= 0.215353
Thermal correction to Enthalpy= 0.216297
Thermal correction to Gibbs Free Energy= 0.164563
Sum of electronic and zero-point Energies= -462.533295
Sum of electronic and thermal Energies= -462.521518
Sum of electronic and thermal Enthalpies= -462.520574
Sum of electronic and thermal Free Energies= -462.572308

1	-5.543373000	-0.296425000	-0.000130000
6	-4.688251000	0.379650000	0.000095000
1	-4.742242000	1.020127000	-0.890242000
1	-4.742299000	1.019612000	0.890799000
8	-3.524742000	-0.404565000	-0.000094000
6	-2.354704000	0.369741000	0.000022000
1	-2.315170000	1.017082000	-0.887395000
1	-2.315235000	1.016915000	0.887562000
6	-1.170286000	-0.570717000	-0.000029000
1	-1.208474000	-1.216966000	-0.887564000
1	-1.208425000	-1.217011000	0.887475000
8	-0.000001000	0.205285000	-0.000041000
6	1.170283000	-0.570718000	0.000065000
1	1.208455000	-1.216860000	0.887678000

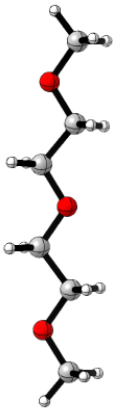

1	1.208445000	-1.217121000	-0.887359000
6	2.354697000	0.369743000	-0.000081000
1	2.315183000	1.016875000	-0.887649000
1	2.315208000	1.017120000	0.887310000
8	3.524741000	-0.404554000	0.000008000
6	4.688260000	0.379641000	0.000011000
1	4.742305000	1.019860000	-0.890510000
1	4.742295000	1.019865000	0.890530000
1	5.543352000	-0.296439000	0.000016000

KOtBu in diglyme

E(electronic) = -2221.34084245

Zero-point correction= 0.741908 (Hartree/Particle)

Thermal correction to Energy= 0.787382

Thermal correction to Enthalpy= 0.788326

Thermal correction to Gibbs Free Energy= 0.659577

Sum of electronic and zero-point Energies= -2220.598935

Sum of electronic and thermal Energies= -2220.553461

Sum of electronic and thermal Enthalpies= -2220.552517

Sum of electronic and thermal Free Energies= -2220.681265

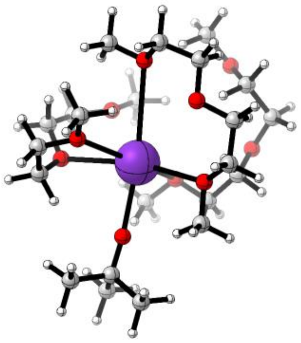

6	4.567455000	0.566286000	-1.471554000
1	3.733179000	1.107488000	-1.008421000
1	5.437741000	0.637728000	-0.803488000
8	4.236222000	-0.773954000	-1.733109000
6	3.858961000	-1.460865000	-0.568026000
1	4.687192000	-1.456936000	0.158034000
1	3.005749000	-0.956727000	-0.092935000
6	3.518851000	-2.899243000	-0.895889000
1	4.299082000	-3.306489000	-1.542729000
1	3.519979000	-3.482307000	0.035894000
8	2.299033000	-3.060564000	-1.576818000
6	1.177868000	-3.044870000	-0.723779000
1	1.104081000	-3.995720000	-0.175930000
1	1.258925000	-2.249678000	0.024037000
6	-0.081166000	-2.843152000	-1.521638000
1	-0.948677000	-2.983695000	-0.861533000
1	-0.140329000	-3.589110000	-2.326485000
8	-0.085405000	-1.543572000	-2.060394000
6	-1.282463000	-1.248967000	-2.751231000
1	-2.129213000	-1.231729000	-2.055371000
1	-1.464510000	-1.978140000	-3.551677000
1	-1.163081000	-0.261118000	-3.195367000
19	-0.640956000	0.141858000	0.148855000
1	-1.922830000	-2.198878000	1.455270000

6	-1.261738000	-2.552946000	2.251666000
1	-1.739642000	-2.358028000	3.220218000
1	-1.083878000	-3.633239000	2.162819000
8	-0.050984000	-1.833901000	2.136725000
6	0.797293000	-1.998539000	3.241071000
1	0.335903000	-1.566275000	4.139661000
1	0.977944000	-3.065195000	3.436347000
6	2.120431000	-1.338157000	2.969809000
1	2.591038000	-1.793641000	2.087050000
1	2.782208000	-1.515536000	3.829679000
8	1.931021000	0.038010000	2.760012000
6	3.139924000	0.722628000	2.549408000
1	3.719145000	0.779881000	3.482150000
1	3.753383000	0.197099000	1.806374000
6	2.859349000	2.115870000	2.060929000
1	2.219018000	2.650909000	2.775520000
1	3.813637000	2.658631000	1.998166000
8	2.245538000	2.052801000	0.799564000
6	2.077706000	3.318029000	0.215183000
1	4.818939000	1.036075000	-2.422531000
1	3.038258000	3.842357000	0.117473000
1	1.400528000	3.948095000	0.806882000
1	1.653877000	3.159175000	-0.773858000
6	-0.780552000	2.898450000	2.725087000
1	-0.268093000	3.858300000	2.579502000
1	-1.557334000	3.032154000	3.488544000
8	-1.325170000	2.418997000	1.524054000
6	-2.276614000	3.279472000	0.948149000
1	-3.095877000	3.480612000	1.652506000
1	-1.812122000	4.244985000	0.703925000
6	-2.875143000	2.610239000	-0.270941000
1	-3.364443000	1.683013000	0.031939000
1	-3.620665000	3.287845000	-0.709251000
8	-1.936553000	2.205061000	-1.242251000
6	-1.177390000	3.221463000	-1.841436000
1	-1.826284000	3.963380000	-2.327573000
1	-0.560104000	3.744243000	-1.101555000
6	-0.288802000	2.588574000	-2.880996000
1	0.296366000	3.375352000	-3.378353000
1	-0.901081000	2.090684000	-3.645486000
8	0.559106000	1.656781000	-2.256252000
6	1.460873000	1.049544000	-3.149185000
1	2.173747000	1.783326000	-3.548390000
1	0.935328000	0.582769000	-3.991095000

1	1.999166000	0.273293000	-2.609823000
1	-0.062224000	2.158602000	3.076276000
8	-2.973900000	-0.920593000	0.097580000
6	-4.262193000	-1.366739000	0.055610000
6	-4.467314000	-2.613828000	0.948586000
1	-5.491745000	-2.999781000	0.905667000
1	-3.790587000	-3.413767000	0.633268000
1	-4.240439000	-2.371156000	1.990784000
6	-5.250267000	-0.283666000	0.546615000
1	-5.227556000	0.577699000	-0.126585000
1	-6.285003000	-0.641493000	0.598188000
1	-4.953249000	0.055575000	1.543415000
6	-4.677210000	-1.755980000	-1.381924000
1	-5.721511000	-2.081398000	-1.450557000
1	-4.540615000	-0.898882000	-2.047826000
1	-4.043572000	-2.569801000	-1.746989000

KF in diglyme

E(electronic) = -2088.15146932

Zero-point correction= 0.619363 (Hartree/Particle)

Thermal correction to Energy= 0.659834

Thermal correction to Enthalpy= 0.660778

Thermal correction to Gibbs Free Energy= 0.543484

Sum of electronic and zero-point Energies= -2087.532106

Sum of electronic and thermal Energies= -2087.491635

Sum of electronic and thermal Enthalpies= -2087.490691

Sum of electronic and thermal Free Energies= -2087.607985

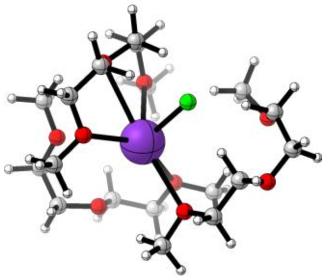

6	2.700751000	1.677253000	-2.235725000
1	1.795011000	1.695777000	-1.620205000
1	2.968636000	2.713913000	-2.486270000
8	3.753646000	1.014251000	-1.586799000
6	4.053020000	1.572998000	-0.322841000
1	4.625030000	2.505134000	-0.451571000
1	3.127137000	1.808535000	0.219686000
6	4.897115000	0.595144000	0.463142000
1	5.718035000	0.238676000	-0.164524000
1	5.333988000	1.123838000	1.322560000
8	4.204153000	-0.552633000	0.897335000
6	3.387894000	-0.319536000	2.029795000
1	4.019572000	-0.020812000	2.880012000
1	2.655579000	0.477720000	1.843118000
6	2.668502000	-1.585594000	2.404360000
1	2.293982000	-1.490805000	3.433323000
1	3.364970000	-2.435165000	2.375256000

8	1.590135000	-1.819342000	1.525347000
6	0.987735000	-3.070388000	1.726895000
1	0.612070000	-3.175144000	2.754499000
1	1.694273000	-3.889708000	1.537361000
1	0.151371000	-3.152033000	1.033207000
19	-0.245963000	0.062218000	0.349538000
1	-2.232854000	-1.342655000	2.991726000
6	-2.793320000	-0.407753000	3.022370000
1	-3.708819000	-0.518433000	2.428435000
1	-3.070683000	-0.202708000	4.064932000
8	-1.969207000	0.601405000	2.504054000
6	-2.570726000	1.876047000	2.548109000
1	-3.574450000	1.833682000	2.102647000
1	-2.675928000	2.204548000	3.591193000
6	-1.722104000	2.869103000	1.799940000
1	-0.670645000	2.764174000	2.091771000
1	-2.064353000	3.884709000	2.044189000
8	-1.854880000	2.629274000	0.415244000
6	-0.943283000	3.361611000	-0.376211000
1	-1.157929000	4.438171000	-0.315586000
1	0.076712000	3.173862000	-0.018113000
6	-1.086149000	2.922857000	-1.809089000
1	-2.121750000	3.052952000	-2.154129000
1	-0.439341000	3.552084000	-2.435964000
8	-0.707528000	1.570771000	-1.923413000
6	-0.755152000	1.084704000	-3.237593000
1	2.494872000	1.137979000	-3.161909000
1	-0.135193000	1.690561000	-3.911199000
1	-1.782103000	1.077897000	-3.626522000
1	-0.367054000	0.066432000	-3.222466000
6	-4.016400000	0.653326000	-0.968219000
1	-3.995993000	0.576292000	-2.063859000
1	-5.066267000	0.670935000	-0.645067000
8	-3.319242000	-0.411193000	-0.371695000
6	-3.863197000	-1.661117000	-0.693220000
1	-4.882392000	-1.765811000	-0.292555000
1	-3.934908000	-1.777532000	-1.784049000
6	-3.004493000	-2.748950000	-0.088748000
1	-3.030828000	-2.676965000	0.999452000
1	-3.424000000	-3.722470000	-0.378895000
8	-1.643208000	-2.672273000	-0.440973000
6	-1.380101000	-2.774646000	-1.821417000
1	-1.897655000	-3.643861000	-2.249283000
1	-1.722463000	-1.877561000	-2.351433000

6	0.100993000	-2.940974000	-2.022733000
1	0.296856000	-3.143020000	-3.085624000
1	0.458765000	-3.804810000	-1.445646000
8	0.762032000	-1.773857000	-1.610367000
6	2.166186000	-1.905858000	-1.636654000
1	2.524118000	-2.049751000	-2.664997000
1	2.490181000	-2.756916000	-1.027214000
1	2.605710000	-1.000308000	-1.226503000
1	-3.525264000	1.572327000	-0.659006000
9	1.261829000	1.902617000	1.236128000

TS-III (with K^+)

E(electronic) = -1577.49532023

Zero-point correction= 0.435428 (Hartree/Particle)
Thermal correction to Energy= 0.461059
Thermal correction to Enthalpy= 0.462003
Thermal correction to Gibbs Free Energy= 0.382604
Sum of electronic and zero-point Energies= -1577.059892
Sum of electronic and thermal Energies= -1577.034262
Sum of electronic and thermal Enthalpies= -1577.033317
Sum of electronic and thermal Free Energies= -1577.112717

Frequency -277.7735

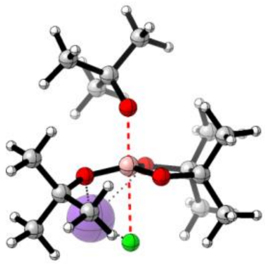

8	0.770498000	-0.747743000	0.899551000
8	0.659245000	0.172806000	-1.228867000
6	2.026751000	0.223988000	-0.883722000
6	2.079880000	-0.317804000	0.618005000
6	3.011414000	-1.512610000	0.801268000
1	4.051478000	-1.258880000	0.577791000
1	2.961651000	-1.843275000	1.841760000
1	2.685909000	-2.334206000	0.166716000
6	2.445731000	0.761357000	1.641040000
1	2.421401000	0.313869000	2.637848000
1	3.446707000	1.170476000	1.479021000
1	1.700612000	1.555752000	1.611005000
6	2.791207000	-0.638539000	-1.889744000
1	2.396207000	-1.652846000	-1.875074000
1	2.648241000	-0.215885000	-2.889946000
1	3.865587000	-0.652097000	-1.691224000
6	2.512146000	1.665094000	-1.033755000
1	3.575629000	1.757905000	-0.798350000
1	2.369682000	1.983495000	-2.069769000
1	1.946700000	2.336842000	-0.392770000

5	-0.101390000	-0.296478000	-0.104405000
8	-1.377528000	-0.818339000	-0.430707000
6	-2.170155000	-1.745483000	0.282932000
6	-3.361319000	-1.004959000	0.889034000
1	-3.009418000	-0.257979000	1.599587000
1	-3.933754000	-0.496229000	0.109347000
1	-4.027969000	-1.700141000	1.407660000
6	-2.676650000	-2.769656000	-0.737160000
1	-1.832607000	-3.340911000	-1.127884000
1	-3.384438000	-3.466445000	-0.281086000
1	-3.194130000	-2.261821000	-1.558172000
6	-1.414819000	-2.478276000	1.388117000
1	-2.066521000	-3.237409000	1.831026000
1	-0.524647000	-2.941181000	0.966216000
1	-1.102971000	-1.785859000	2.168970000
8	-0.557893000	1.326034000	0.720474000
6	-1.208090000	2.419741000	0.206487000
6	-2.667831000	2.494977000	0.705438000
1	-3.241860000	1.654023000	0.316192000
1	-2.685272000	2.440029000	1.797409000
1	-3.166835000	3.422150000	0.398918000
6	-0.494710000	3.700156000	0.691829000
1	-0.461276000	3.703845000	1.785327000
1	0.535121000	3.718658000	0.326442000
1	-0.992222000	4.618483000	0.357746000
6	-1.250322000	2.451675000	-1.335359000
1	-0.245660000	2.457235000	-1.751357000
1	-1.755742000	1.554718000	-1.703053000
1	-1.792040000	3.333918000	-1.695602000
9	0.505087000	-2.474049000	-1.053102000
19	-0.524362000	-1.412744000	-2.887792000

TS-III (without K^+)

E(electronic) = -977.836508591

Zero-point correction= 0.436324 (Hartree/Particle)

Thermal correction to Energy= 0.458830

Thermal correction to Enthalpy= 0.459774

Thermal correction to Gibbs Free Energy= 0.389973

Sum of electronic and zero-point Energies= -977.172761

Sum of electronic and thermal Energies= -977.150255

Sum of electronic and thermal Enthalpies= -977.149311

Sum of electronic and thermal Free Energies= -977.219112

Frequency -299.1071

8	0.721358000	-0.738409000	0.954312000
8	0.874287000	0.301131000	-1.131454000
6	2.174290000	0.351334000	-0.602263000
6	2.040428000	-0.267540000	0.868882000
6	2.977007000	-1.447367000	1.120690000
1	4.031859000	-1.166878000	1.037906000
1	2.807110000	-1.825684000	2.133503000
1	2.729847000	-2.234412000	0.408831000
6	2.222832000	0.767901000	1.984881000
1	2.076315000	0.267304000	2.945910000
1	3.219159000	1.220441000	1.988036000
1	1.459109000	1.539920000	1.889569000
6	3.079556000	-0.442517000	-1.548499000
1	2.625024000	-1.425757000	-1.675640000
1	3.096050000	0.069403000	-2.516439000
1	4.111138000	-0.518350000	-1.190856000
6	2.670335000	1.800507000	-0.602414000
1	3.712938000	1.861240000	-0.274372000
1	2.619333000	2.187736000	-1.623628000
1	2.064131000	2.443211000	0.030103000
5	0.002282000	-0.325884000	-0.194612000
8	-1.162952000	-0.952962000	-0.679535000
6	-2.020349000	-1.778609000	0.056333000
6	-2.625197000	-1.065747000	1.272950000
1	-1.834245000	-0.656148000	1.898637000
1	-3.255322000	-0.230724000	0.962413000
1	-3.235632000	-1.766542000	1.853258000
6	-3.149747000	-2.163606000	-0.903813000
1	-2.747159000	-2.722441000	-1.752216000
1	-3.899828000	-2.784379000	-0.404324000
1	-3.644362000	-1.267737000	-1.288506000
6	-1.314027000	-3.056500000	0.517018000
1	-2.040018000	-3.778016000	0.909542000
1	-0.762449000	-3.464427000	-0.329199000
1	-0.576042000	-2.823957000	1.284867000
8	-0.747415000	1.198035000	0.603506000
6	-1.433576000	2.210247000	-0.007233000
6	-2.450488000	2.803313000	0.993365000
1	-3.160771000	2.032163000	1.302353000
1	-1.921532000	3.145977000	1.887638000
1	-3.016207000	3.648575000	0.580816000
6	-0.490967000	3.358004000	-0.440725000
1	0.085202000	3.707162000	0.421809000
1	0.209655000	2.987829000	-1.189346000

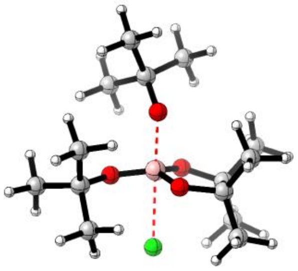

1	-1.033533000	4.214787000	-0.860632000
6	-2.212058000	1.773017000	-1.268094000
1	-1.527605000	1.335560000	-1.995353000
1	-2.949582000	1.009460000	-1.018972000
1	-2.730260000	2.622338000	-1.730701000
9	1.020646000	-2.390579000	-1.037006000

TS-A'C'

E(electronic) = -911.925246789
Zero-point correction= 0.313861 (Hartree/Particle)
Thermal correction to Energy= 0.332880
Thermal correction to Enthalpy= 0.333824
Thermal correction to Gibbs Free Energy= 0.264116
Sum of electronic and zero-point Energies= -911.611386
Sum of electronic and thermal Energies= -911.592367
Sum of electronic and thermal Enthalpies= -911.591422
Sum of electronic and thermal Free Energies= -911.661131

Frequency -1382.4747

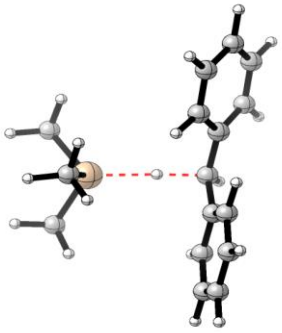

14	0.018753000	-0.393327000	-3.718875000
6	1.527122000	-0.508828000	-4.895489000
1	1.834629000	-1.548031000	-5.052523000
1	1.311871000	-0.075574000	-5.881123000
1	2.390542000	0.021679000	-4.480913000
6	-1.378867000	-1.198541000	-4.754877000
1	-1.448046000	-0.760082000	-5.759194000
1	-1.222899000	-2.275707000	-4.876039000
1	-2.350605000	-1.067325000	-4.267588000
6	-0.431672000	1.467335000	-3.802565000
1	0.388928000	2.087258000	-3.426684000
1	-0.650203000	1.795850000	-4.827178000
1	-1.310806000	1.688587000	-3.188492000
6	0.155147000	-1.000056000	-0.475195000
1	0.231955000	-2.081146000	-0.353384000
1	0.162596000	-0.874455000	-1.866114000
6	-1.189421000	-0.517942000	-0.165023000
6	-1.496451000	0.711744000	0.439591000
6	-2.285689000	-1.301595000	-0.576990000
6	-2.807160000	1.121058000	0.629447000
6	-3.593307000	-0.891791000	-0.385938000
6	-3.871561000	0.328364000	0.218993000
1	-0.696214000	1.351201000	0.789594000
1	-2.090414000	-2.255551000	-1.055652000

1	-2.998609000	2.074124000	1.110823000
1	-4.405345000	-1.529509000	-0.718251000
1	-4.894203000	0.653299000	0.366953000
6	1.378040000	-0.307114000	-0.086226000
6	1.594767000	1.049430000	-0.391650000
6	2.468696000	-1.008242000	0.455887000
6	2.805004000	1.670118000	-0.122806000
6	3.679702000	-0.390243000	0.715994000
6	3.857737000	0.961146000	0.439952000
1	0.804427000	1.617090000	-0.867053000
1	2.349695000	-2.063363000	0.680151000
1	2.931084000	2.718295000	-0.371479000
1	4.492889000	-0.967190000	1.142896000
1	4.802945000	1.447580000	0.648465000

7. References

1. J. Zhou, B. Jiang, Y. Fujihira, Z. Zhao, T. Imai and N. Shibata, *Nat. Commun.*, 2021, **12**, 3749.
2. L. Li, C. Ni, F. Wang and J. Hu, *Nat. Commun.* 2016, **7**, 13320.
3. S. Chiba and D. Y. Ong, *Synthesis* 2020, **52**, 1369–1378.
4. C. Rösner and U. Hennecke, *Org. Lett.* 2015, **17**, 3226–3229.
5. M. Shibuya, M. Abe, S. Fujita and Y. Yamamoto, *Org. Biomol. Chem.* 2016, **14**, 5322–5328.
6. M. Dryzhakov and J. Moran, *ACS Catal.* 2016, **6**, 3670–3673.
7. M. Kirihara, Y. Kikkawa, R. Nakamura, K. Nakakura, Y. Suzuki and Y. Muramatsu, *Tetrahedron Lett.* 2021, **64**, 152655.
8. J. Zhou, Z. Zhao, B. Jiang, K. Yamamoto, Y. Sumii and N. Shibata, *Chem. Sci.*, 2023, **14**, 4248–4256.
9. A. Ono, N. Suzuki, and J. Kamimura, *Synthesis* 1987, **8**, 736–738.
10. Y. Cheng, W. Dong, L. Wang, K. Parthasarathy and C. Bolm, *Org. Lett.* 2014, **16**, 2000–2002.
11. D. Kalaitzakis, T. Montagnon, E. Antonatou, N. Bardají and G. Vassilikogiannakis, *Chem. Eur. J.* 2013, **19**, 10119–10123.
12. G. Toupalas, G. Thomann, L. Schlemper, M. A. Rivero-Crespo, H. L. Schmitt and B. Morandi, *ACS Catal.* 2022, **12**, 8147–8154.
13. Y. Liang, U. K. Das, J. Luo, Y. Diskin-Posner, L. Avram and D. Milstein, *J. Am. Chem. Soc.* 2022, **144**, 19115–19126.
14. Y. He, Y. Cai and S. Zhu, *J. Am. Chem. Soc.* 2017, **139**, 1061–1064.
15. Y. He, C. Liu, L. Yu and S. Zhu, *Angew. Chem., Int. Ed.* 2020, **59**, 21530–21534.
16. W. Xu, P. Zheng, J. Zhou, Z. Hu and T. Xu, *Angew. Chem. Int. Ed.* 2022, **61**, e202214213.
17. K. B. Urkalan and M. S. Sigman, *Angew. Chem. Int. Ed.* 2009, **4**, 3146–3149.
18. T. Suga, Y. Takahashi, C. Miki and Y. Ukaji, *Angew. Chem. Int. Ed.* 2022, **61**, e202112533.
19. E. Grovenstein, J. A. Beres, Y. M. Cheng and J. A. Pegolotti, *J. Org. Chem.* 1972, **37**, 1281–1292.

20. K. Nozawa-Kumada, S. Onuma, K. Ono, T. Kumagai, Y. Iwakawa, K. Sato, M. Shigeno and Y. Kondo, *Chem. Eur. J.* 2023, **29**, e202203143.
21. C. Melero, R. P. Herrera, A. Guijarro and M. Yus, *Chem. Eur. J.* 2007, **13**, 10096–10107.
22. P. J. Boratyński, M. Pink, S. Rajca and A. Rajca, *Angew. Chem. Int. Ed.* 2010, **49**, 5459–5462.
23. S. Sivasanker and A. Thangaraj, *J. Catal.* 1992, **138**, 386–390.
24. Gaussian 16, Revision B.01, M. J. Frisch, G. W. Trucks, H. B. Schlegel, G. E. Scuseria, M. A. Robb, J. R. Cheeseman, G. Scalmani, V. Barone, G. A. Petersson, H. Nakatsuji, X. Li, M. Caricato, A. V. Marenich, J. Bloino, B. G. Janesko, R. Gomperts, B. Mennucci, H. P. Hratchian, J. V. Ortiz, A. F. Izmaylov, J. L. Sonnenberg, D. Williams-Young, F. Ding, F. Lipparini, F. Egidi, J. Goings, B. Peng, A. Petrone, T. Henderson, D. Ranasinghe, V. G. Zakrzewski, J. Gao, N. Rega, G. Zheng, W. Liang, M. Hada, M. Ehara, K. Toyota, R. Fukuda, J. Hasegawa, M. Ishida, T. Nakajima, Y. Honda, O. Kitao, H. Nakai, T. Vreven, K. Throssell, J. A. Montgomery, Jr., J. E. Peralta, F. Ogliaro, M. J. Bearpark, J. J. Heyd, E. N. Brothers, K. N. Kudin, V. N. Staroverov, T. A. Keith, R. Kobayashi, J. Normand, K. Raghavachari, A. P. Rendell, J. C. Burant, S. S. Iyengar, J. Tomasi, M. Cossi, J. M. Millam, M. Klene, C. Adamo, R. Cammi, J. W. Ochterski, R. L. Martin, K. Morokuma, O. Farkas, J. B. Foresman, D. J. Fox, Gaussian, Inc., Wallingford CT, 2016.
25. J.-D. Chai and M. Head-Gordon, *Phys. Chem. Chem. Phys.*, 2008, **10**, 6615–6620.
26. (a) F. Weigend and R. Ahlrichs, *Phys. Chem. Chem. Phys.*, 2005, **7**, 3297-3305; (b) F. Weigend, *Phys. Chem. Chem. Phys.*, 2006, **8**, 1057-65.
27. A. V. Marenich, C. J. Cramer and Do. G. Truhlar, *J. Phys. Chem. B*, 2009, **113**, 6378–6396.
28. (a) C. Gonzalez and H. B. Schlegel, J. Phys. Chem., 1990, **94**, 5523–5527. (b) K. Fukui, *Acc. Chem. Res.*, 1981, **14**, 363–368.
29. CYLview20; Legault, C. Y., Université de Sherbrooke, 2020 (http://www.cylview.org).

8. Supplementary NMR spectra (^{1}H NMR, ^{13}C NMR and ^{19}F NMR)

1-Fluorooctadecane (1c)

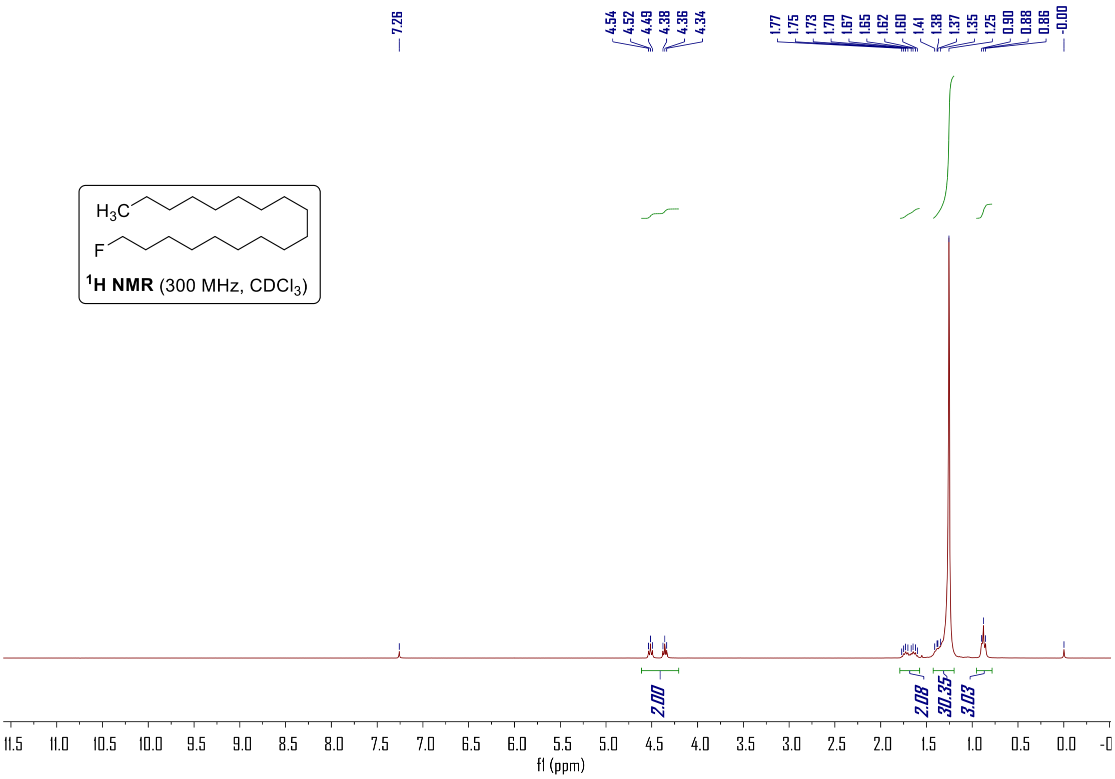

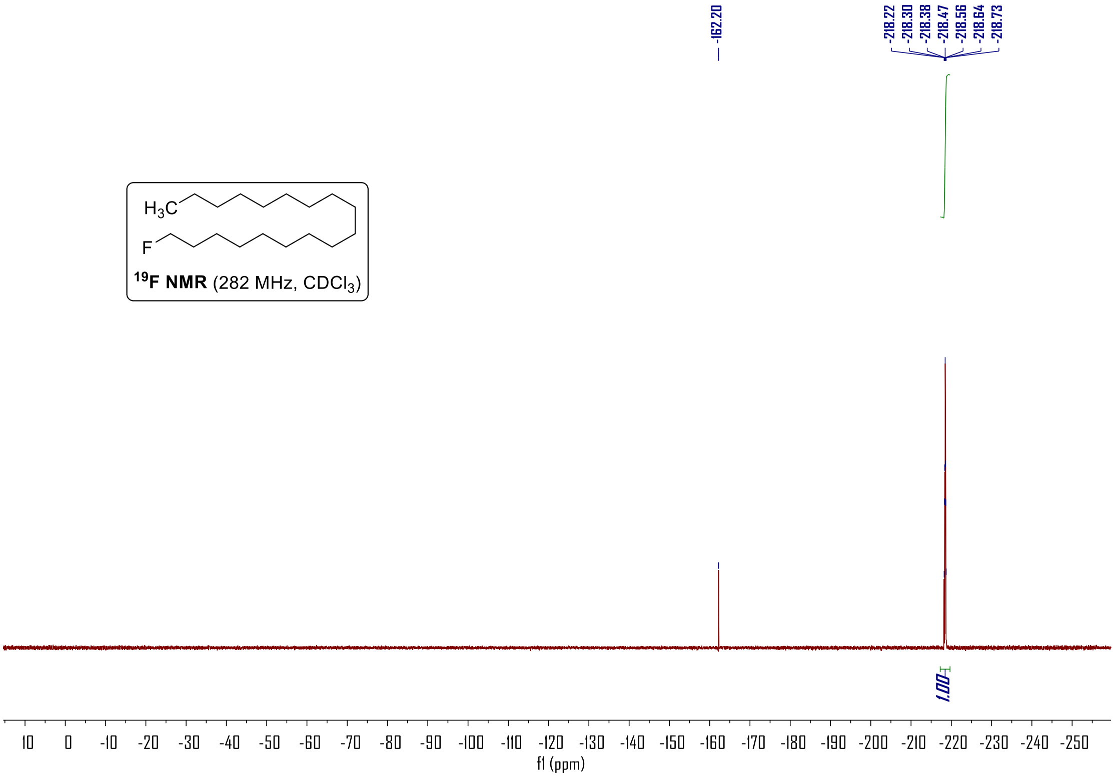

(5-Fluoropentan-2-yl)benzene (1h)

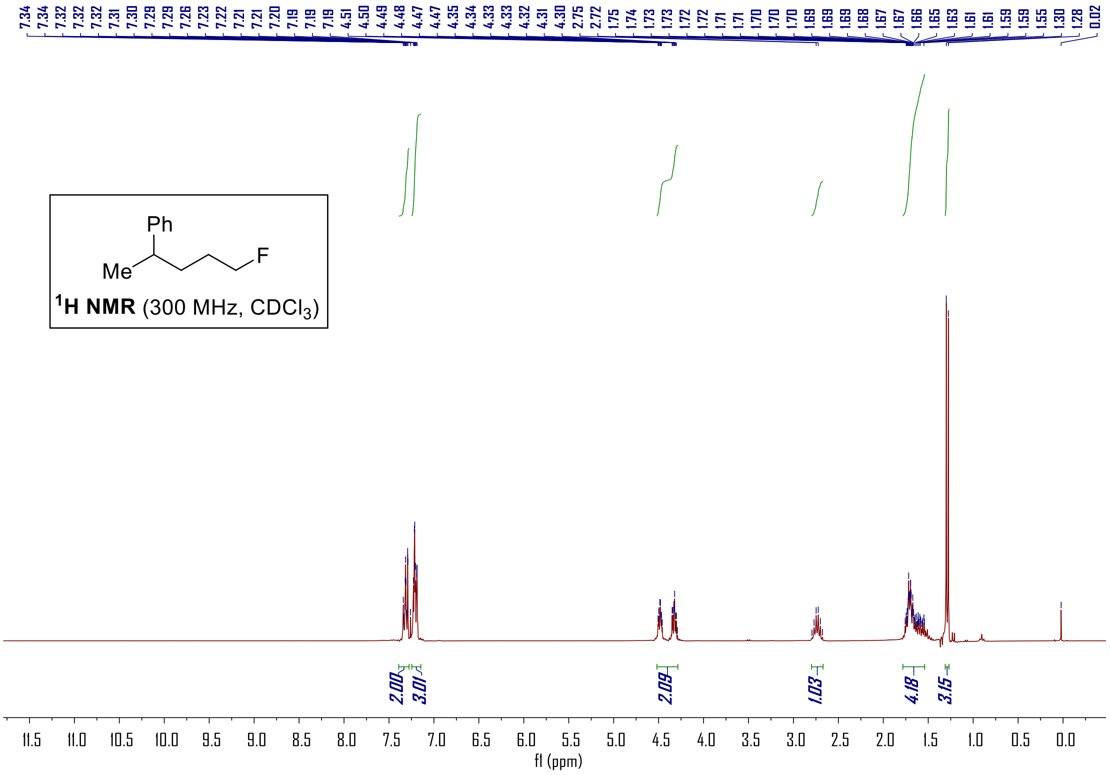

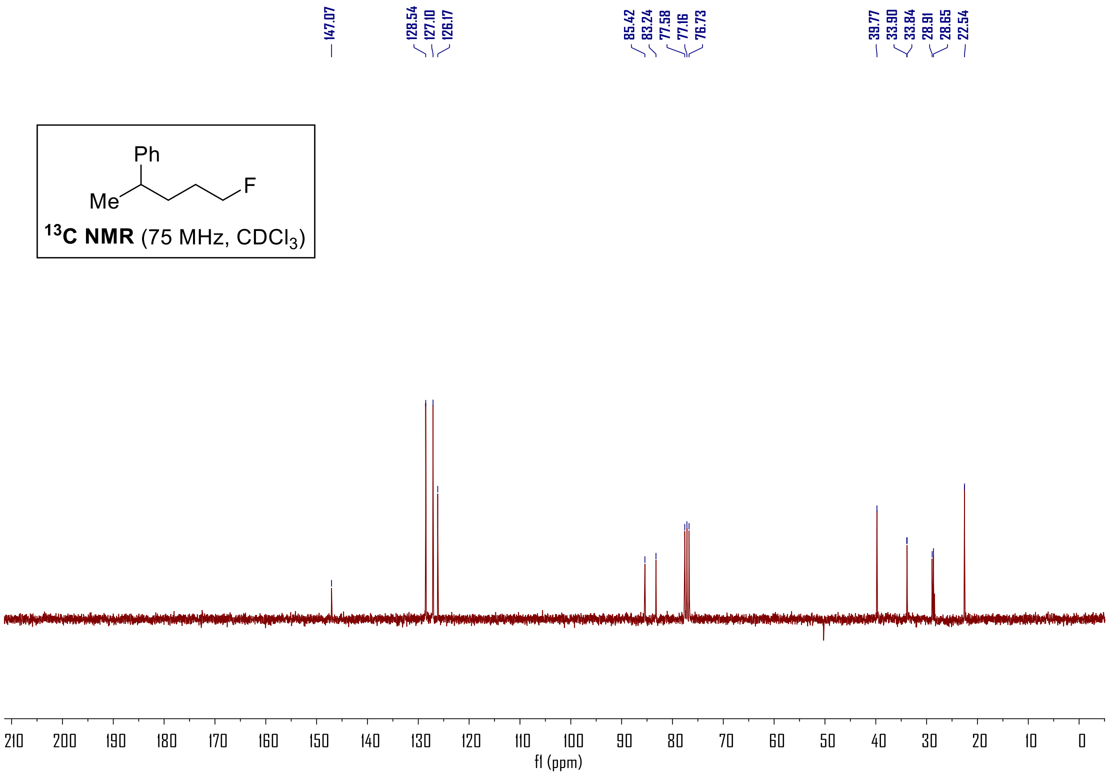

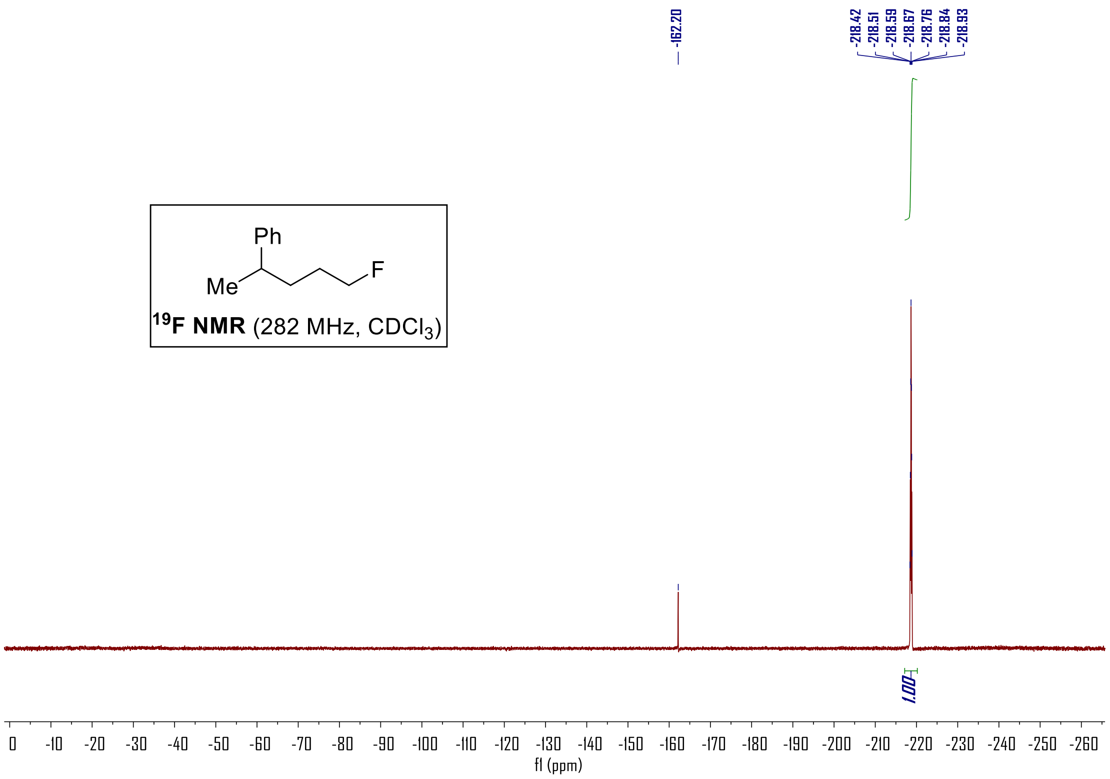

(4-Fluoro-2-methylbutan-2-yl)benzene (1i)

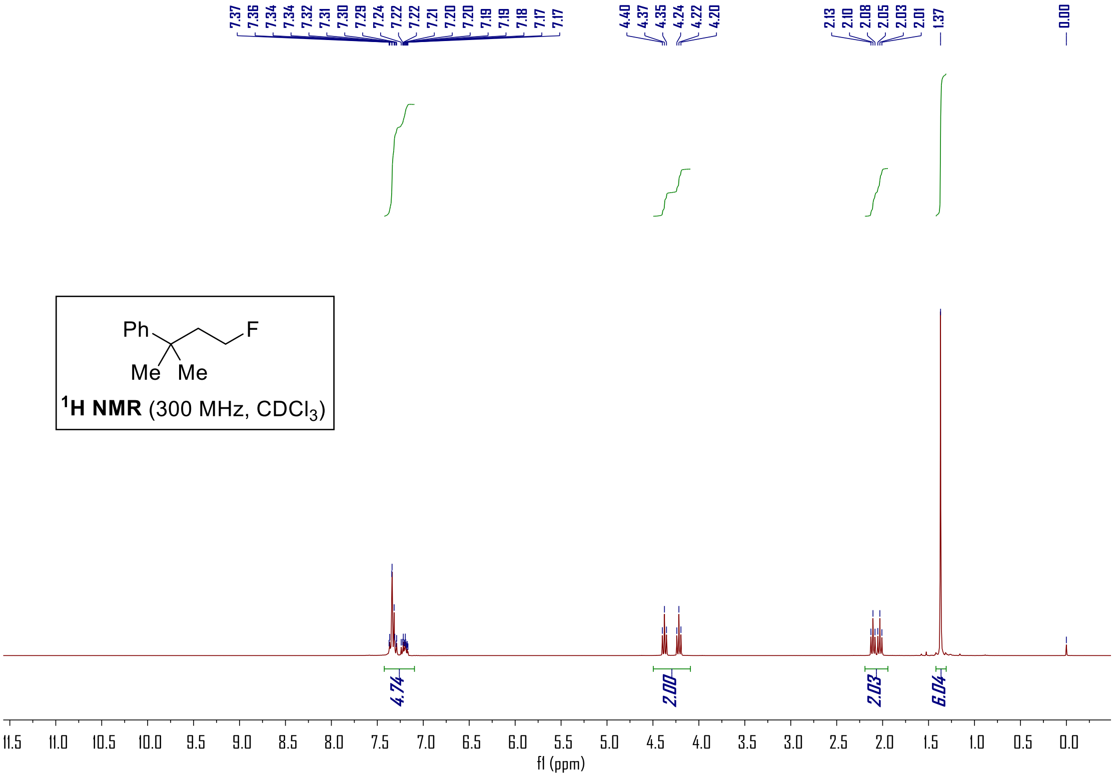

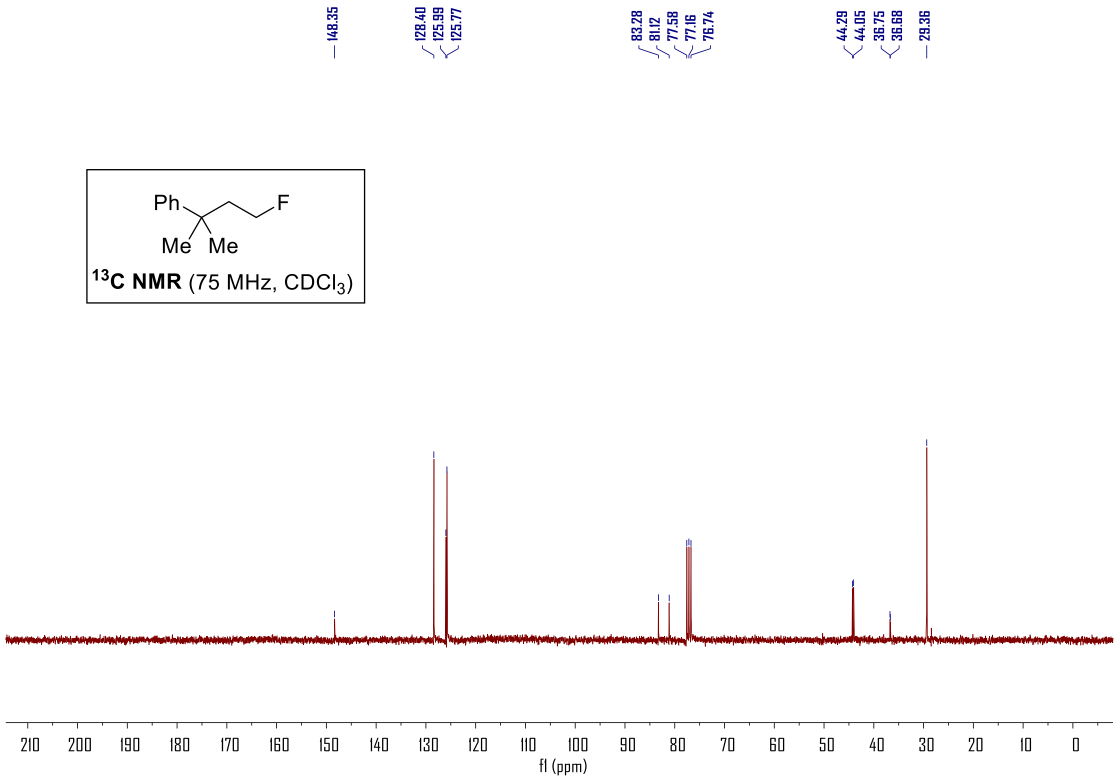
148.35
128.40
125.99
125.77
83.28
81.12
77.58
77.16
76.74
44.29
44.05
36.75
36.68
29.36
Ph
F
Me
Me
13C NMR (75 MHz, CDCl3)
fl (ppm)

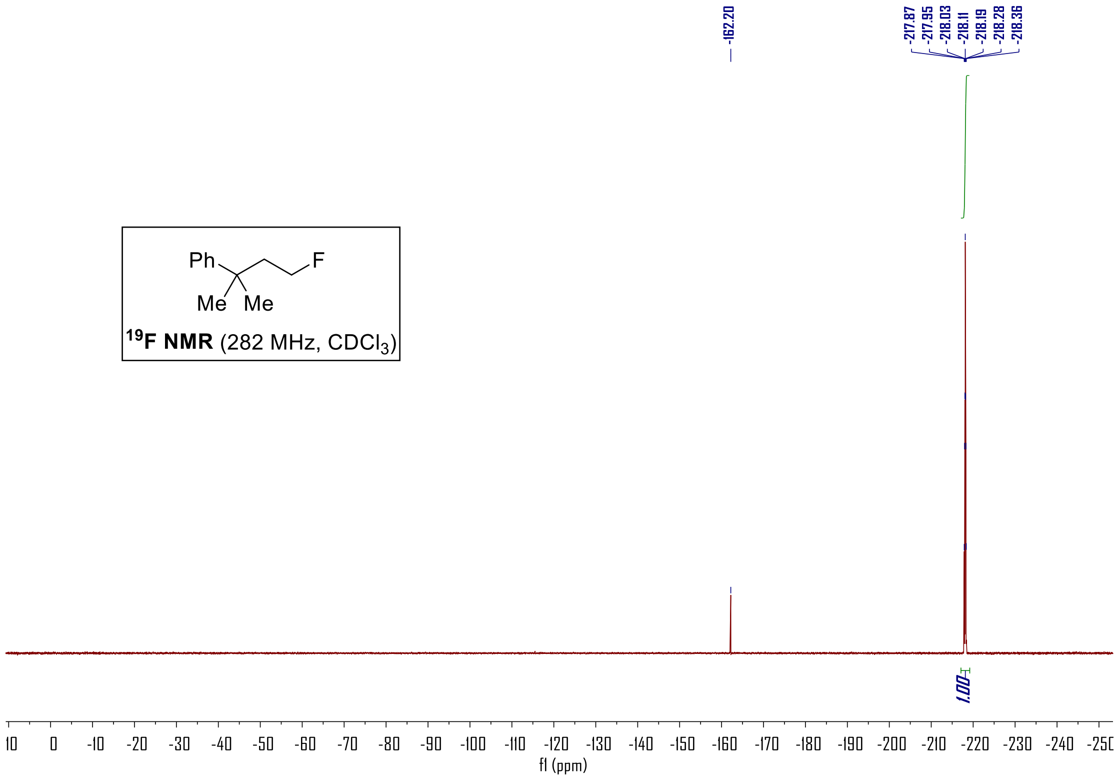
-162.20
-217.87
-217.95
-218.03
-218.11
-218.19
-218.28
-218.36
Ph
F
Me
Me
19F NMR (282 MHz, CDCl3)
1.00
fl (ppm)

(1-(3-Fluoropropyl)cyclopropyl)benzene (1j)

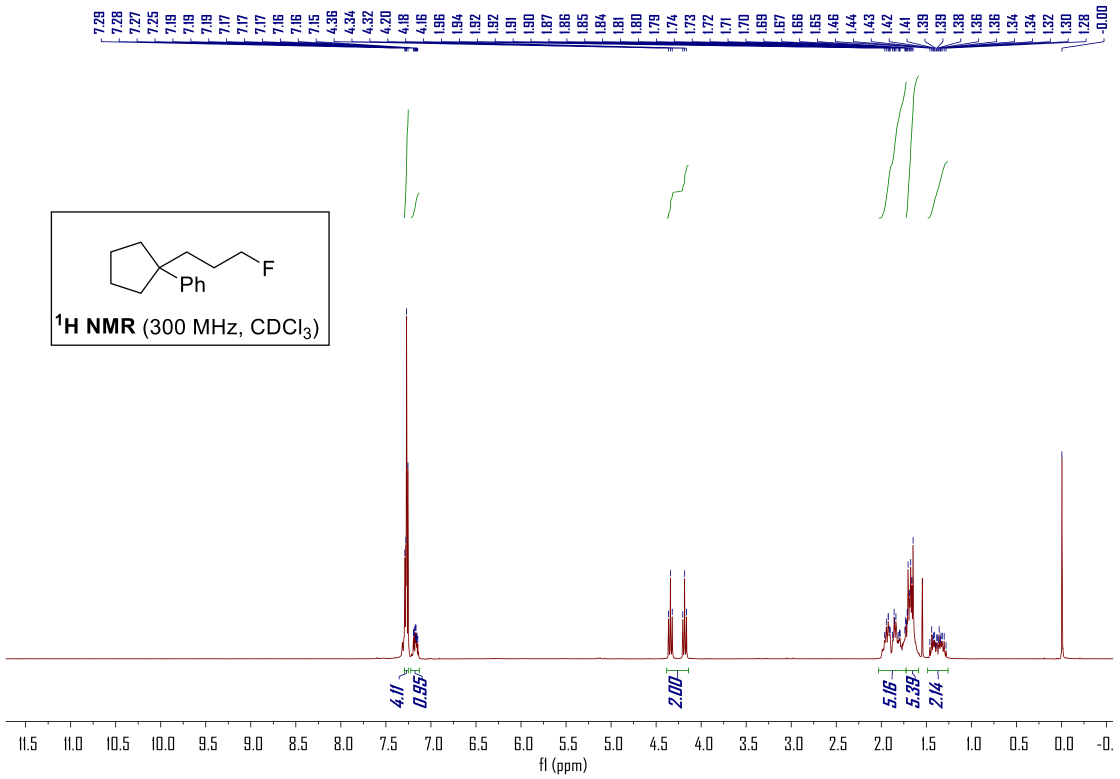

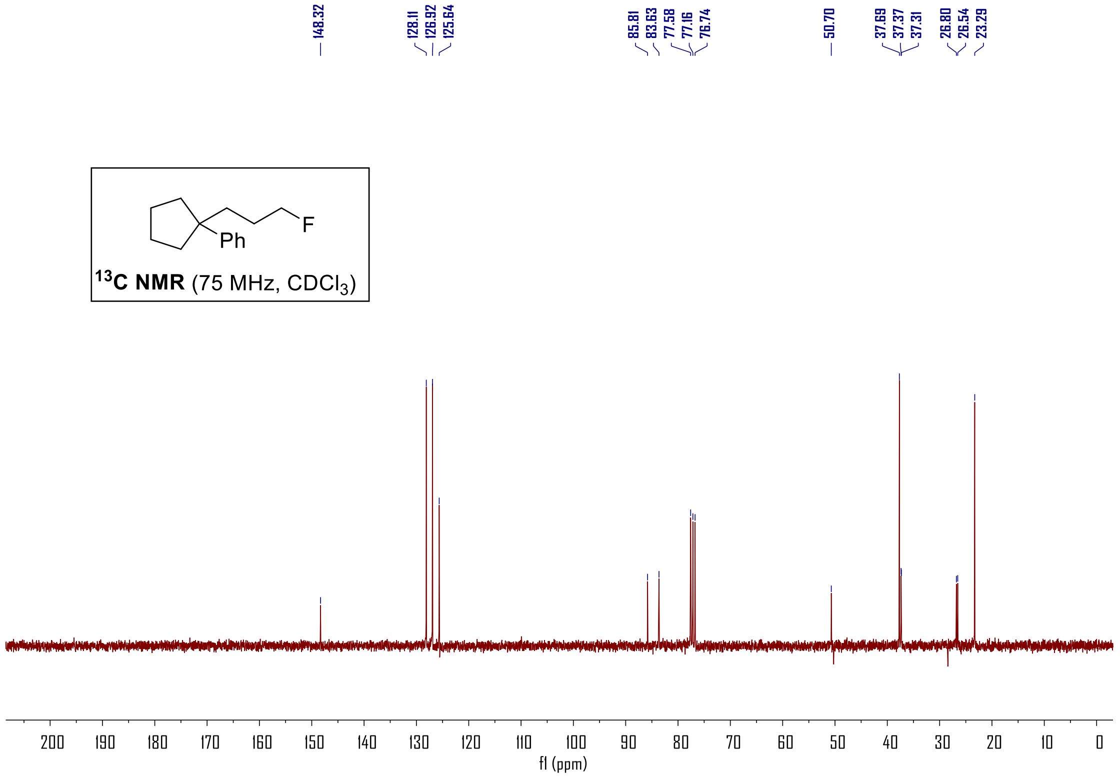

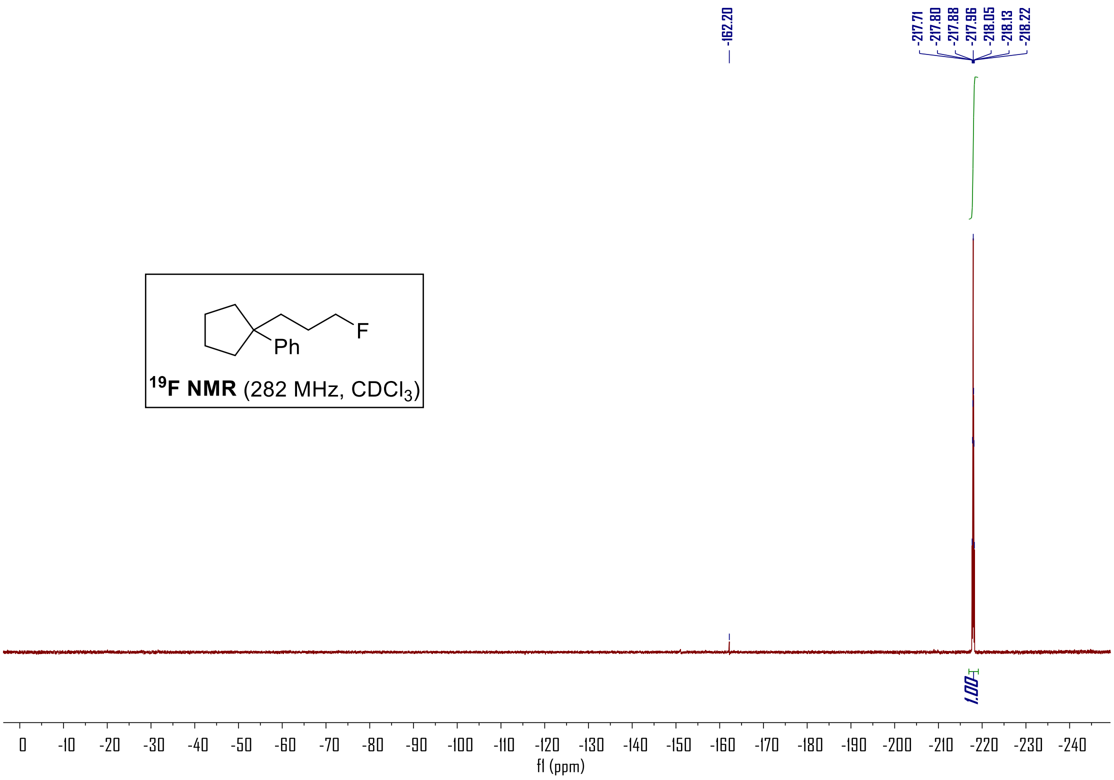

(3-Fluorobutyl)benzene (1l)

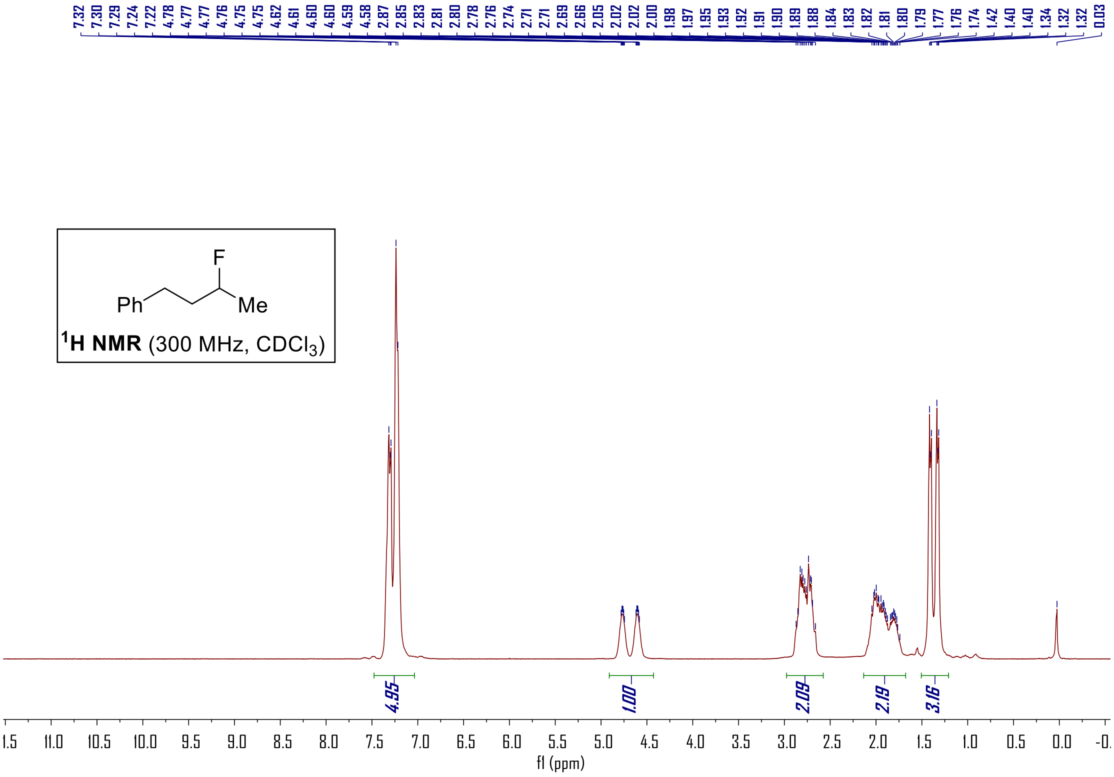

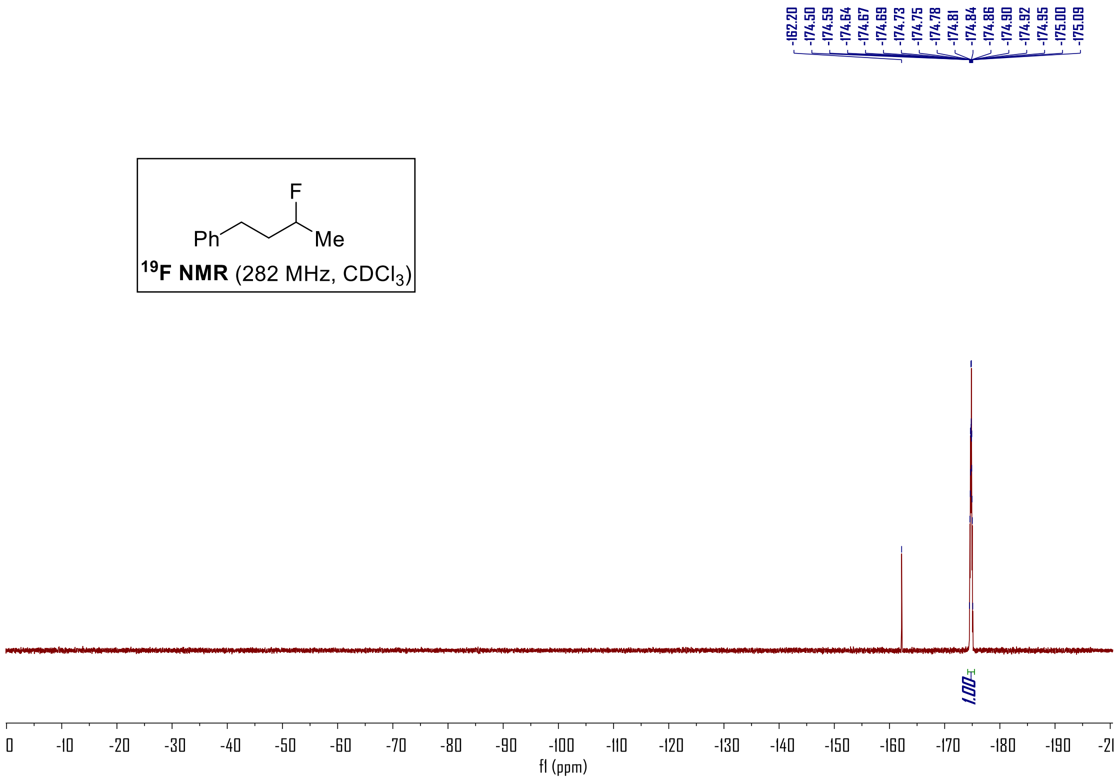

(2-(Fluoromethyl)cyclopropyl)benzene (1s)

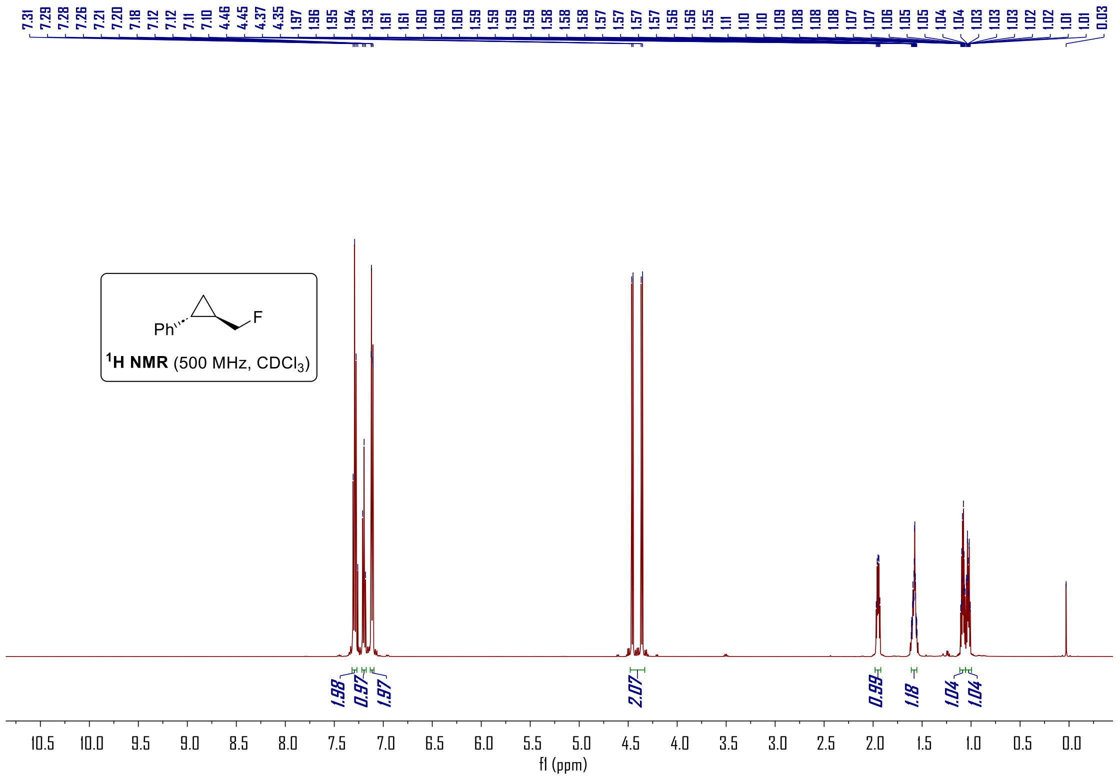

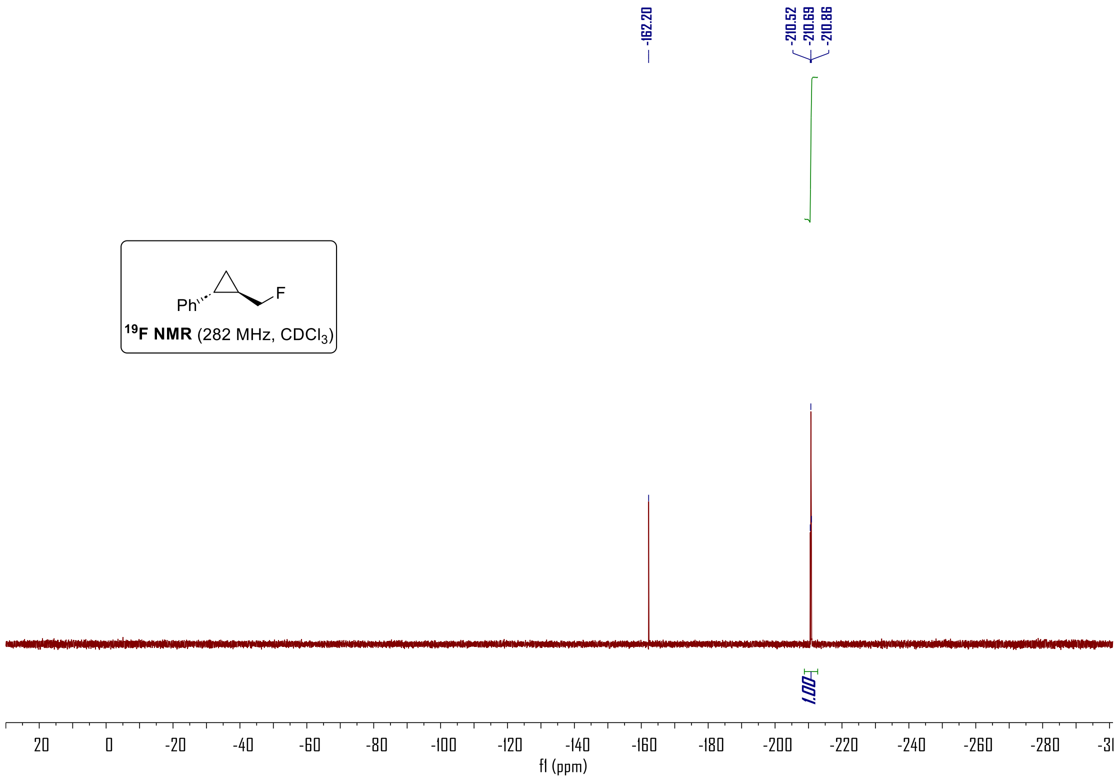

1-Benzyl-2-methoxybenzene (2d)

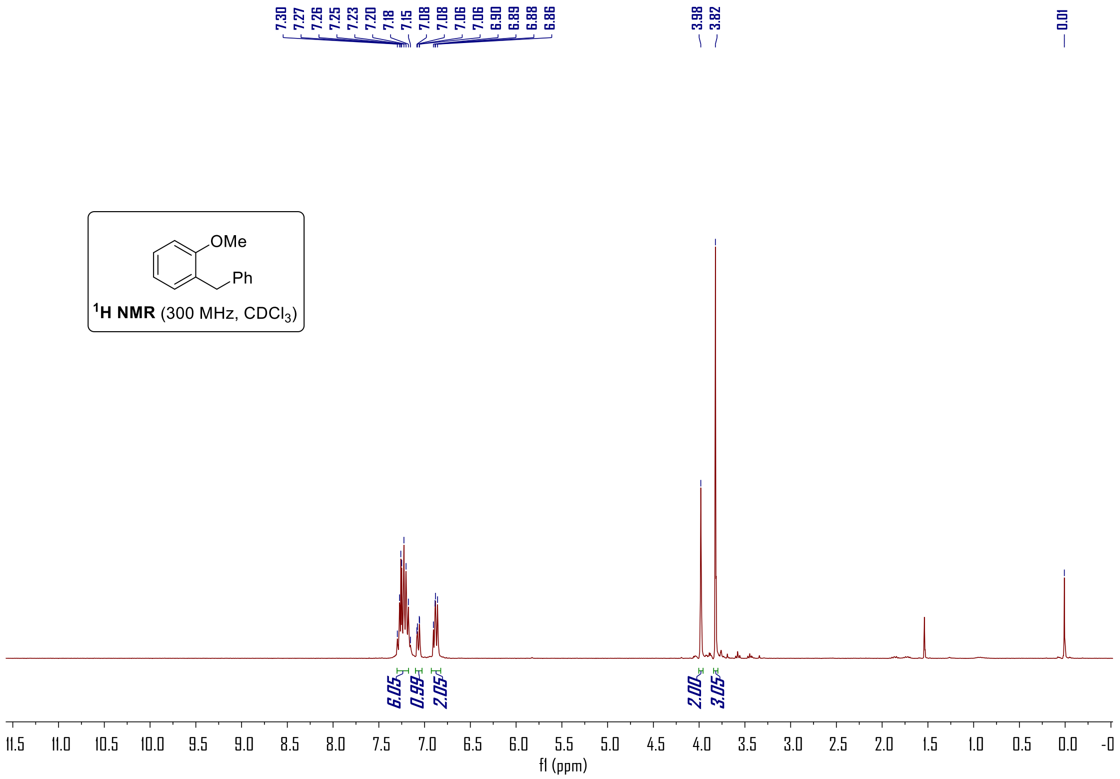

1-Benzyl-3-chlorobenzene (2e)

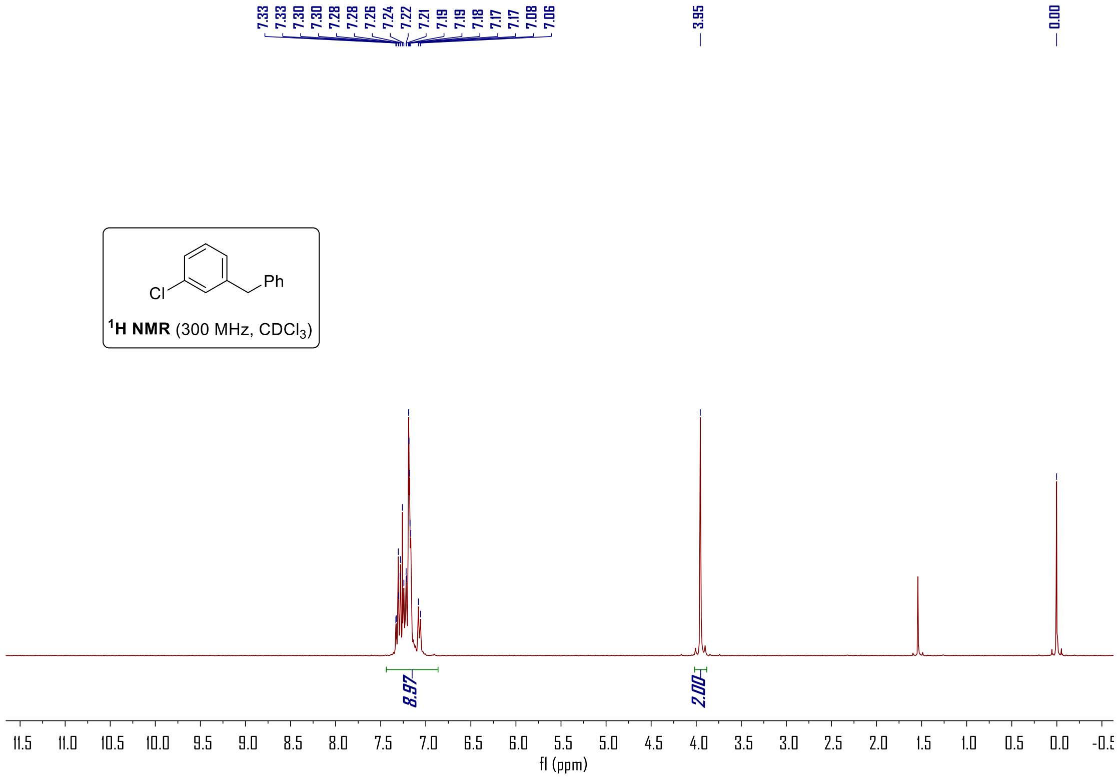

4-(4-Methylbenzyl)-biphenyl (2i)

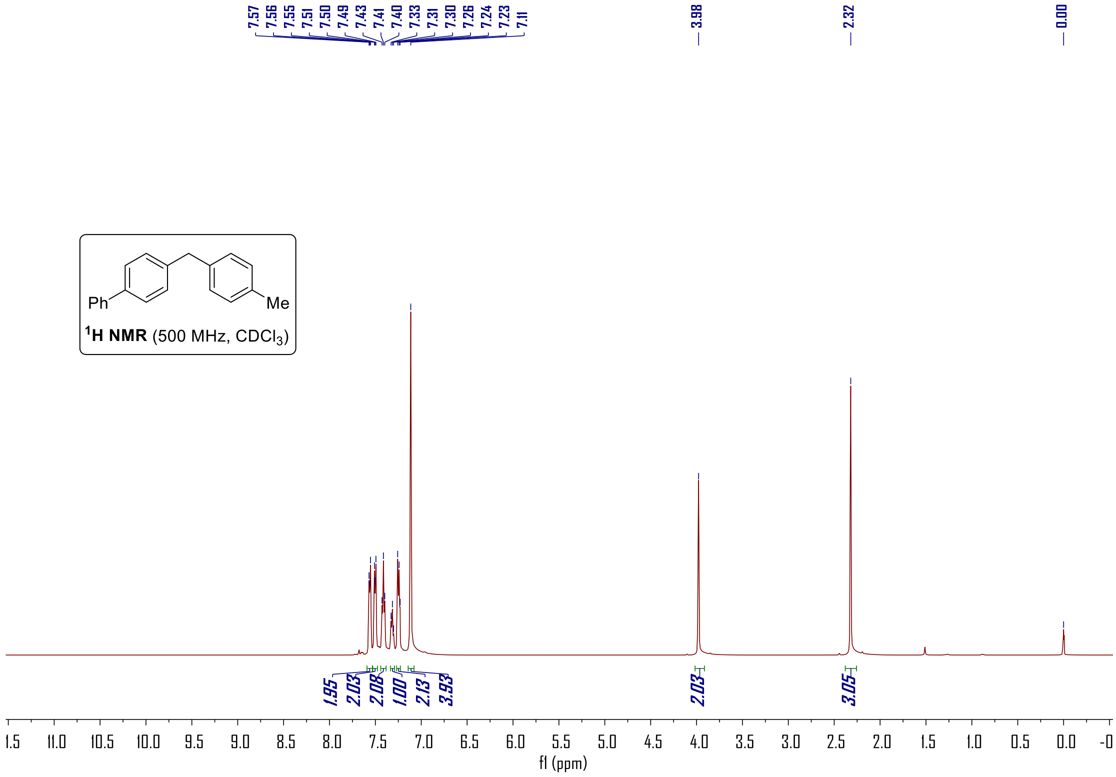

1-Ethyl-3-methoxybenzene (2o)

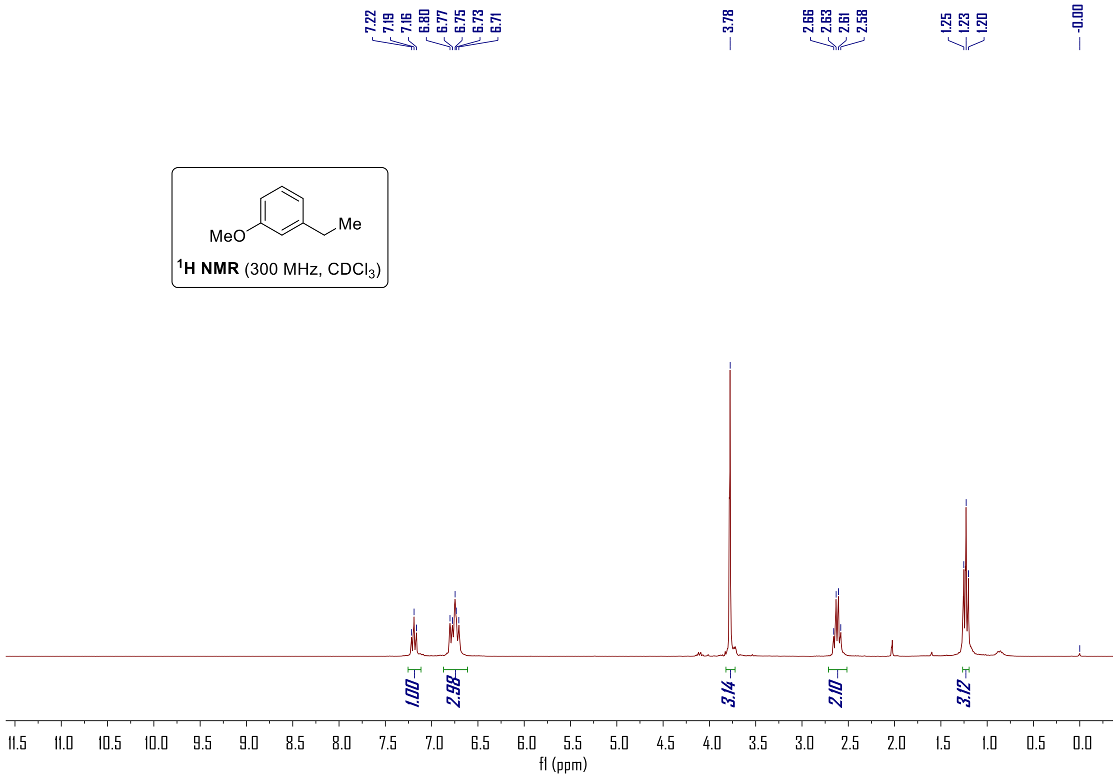

2-Benzylfuran (2u)

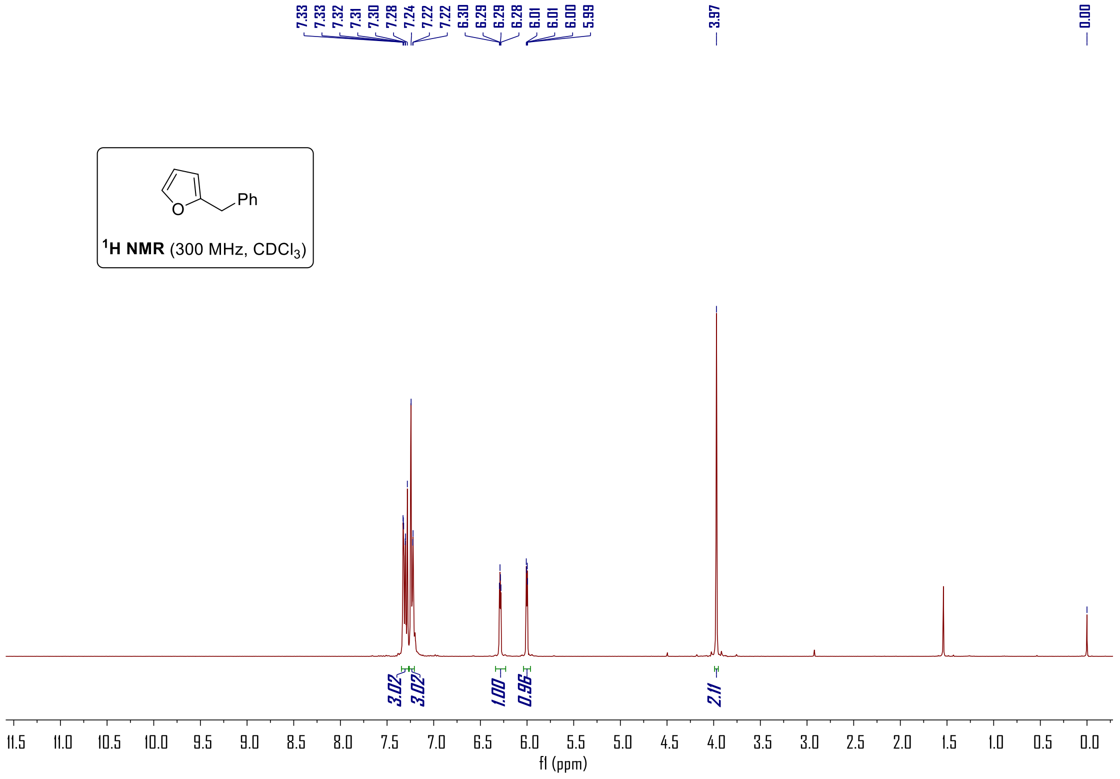

1-Methoxy-4-(1-phenylnonyl)benzene (3aa)

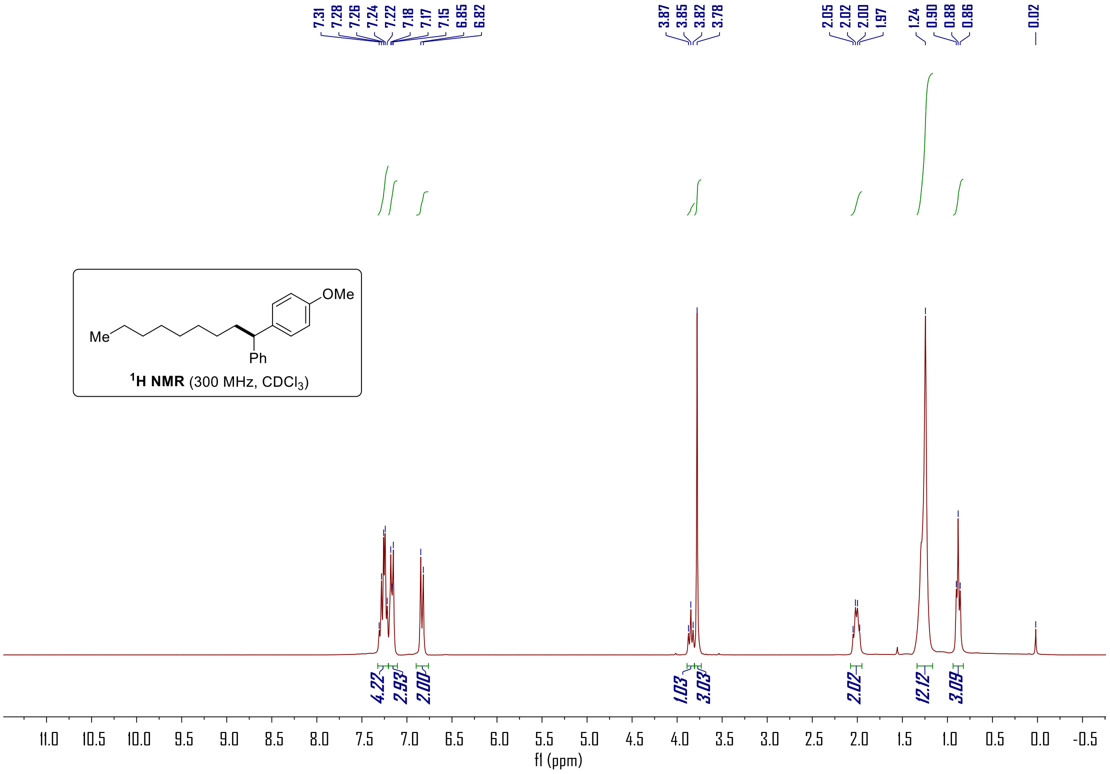

1-Methoxy-4-(1-phenylundecyl)benzene (3ba)

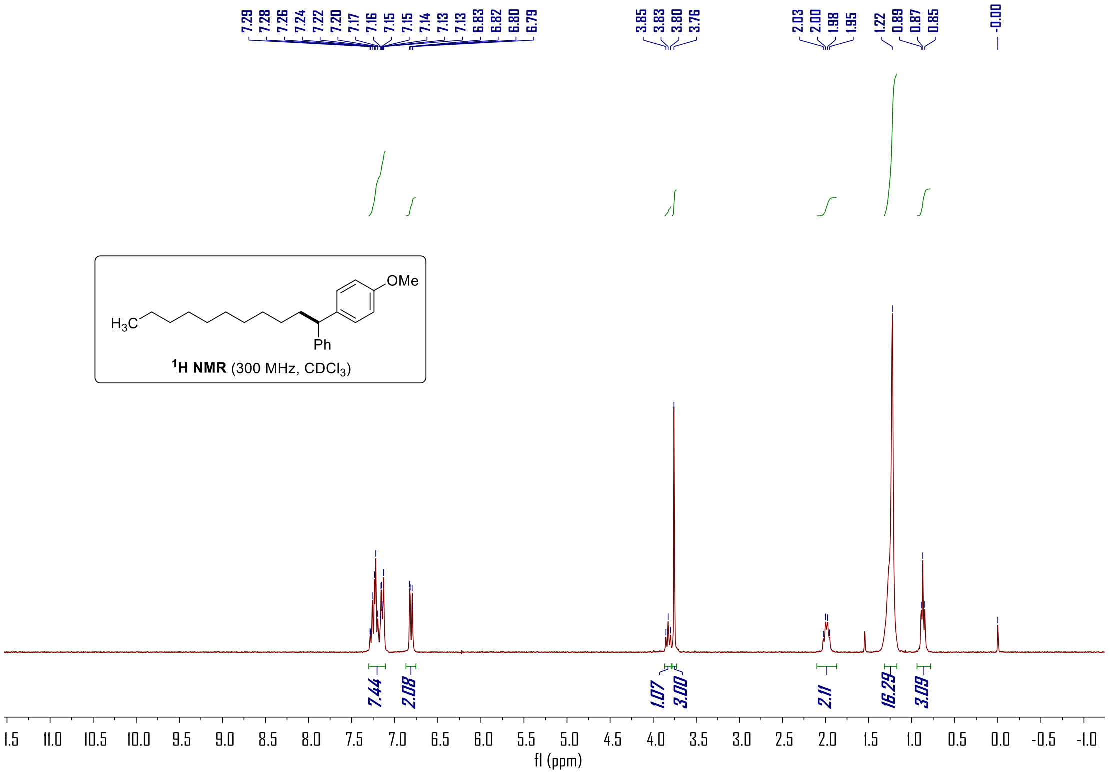

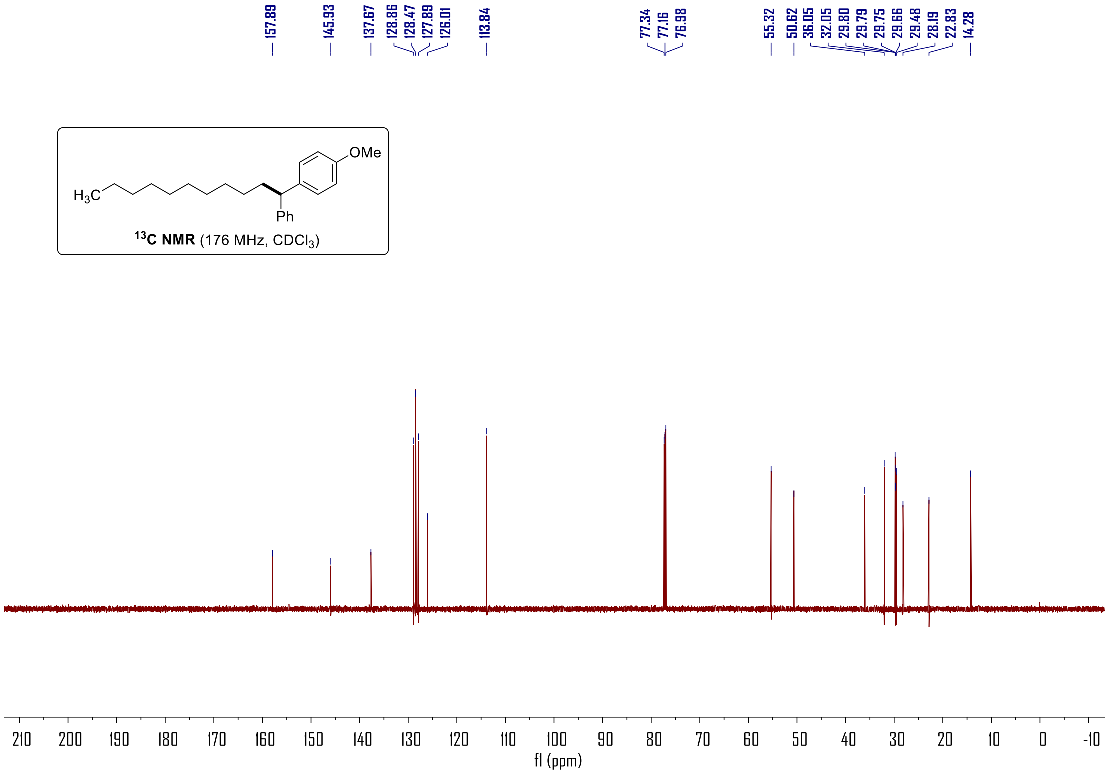

1-Methoxy-4-(1-phenylnonadecyl)benzene (3ca)

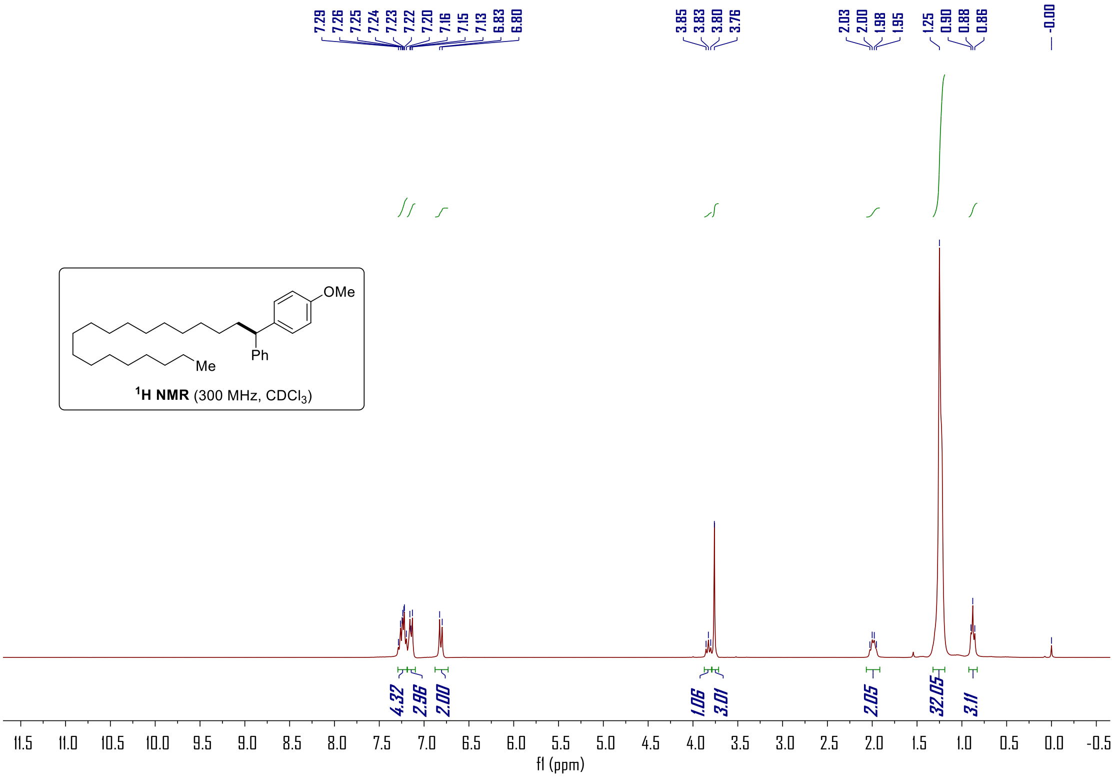

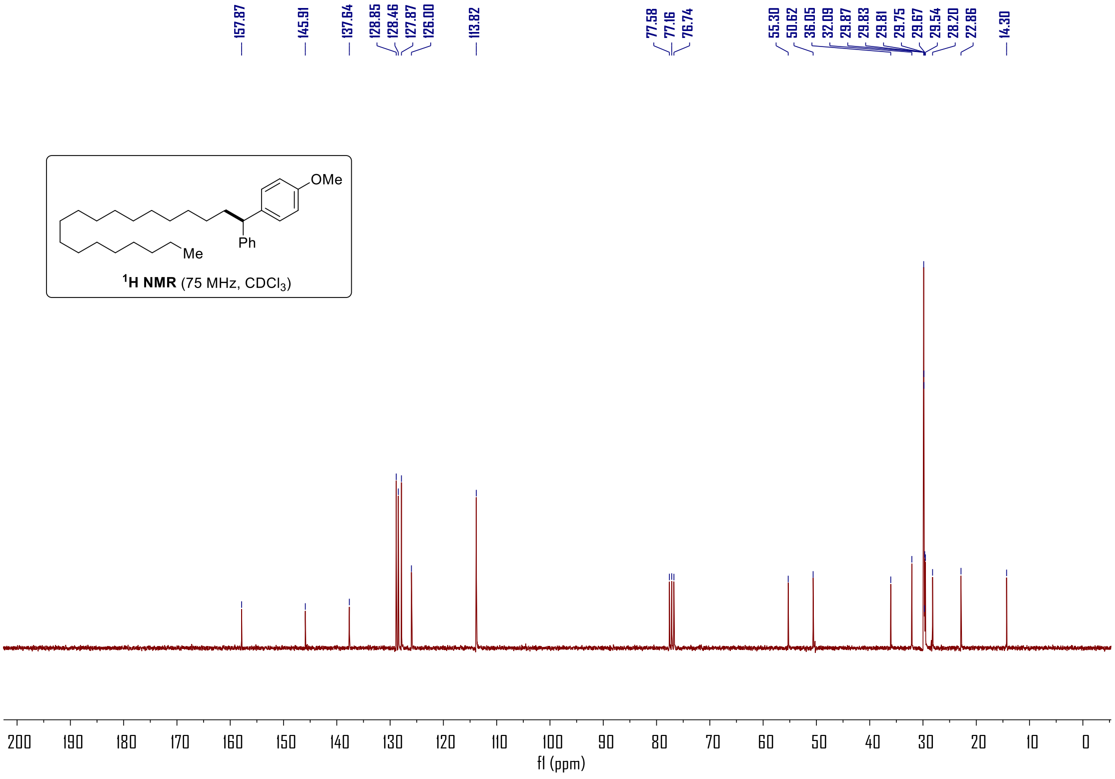

1-(4-(4-Methoxyphenyl)-4-phenylbutyl)adamantane (3da)

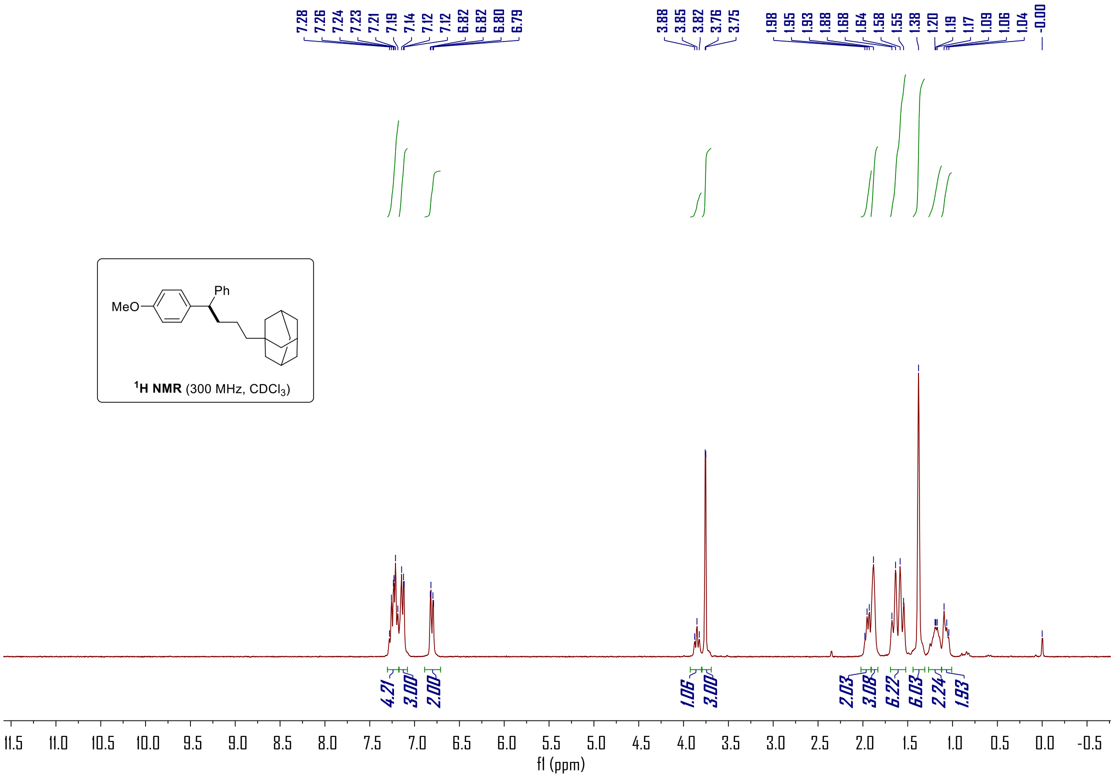

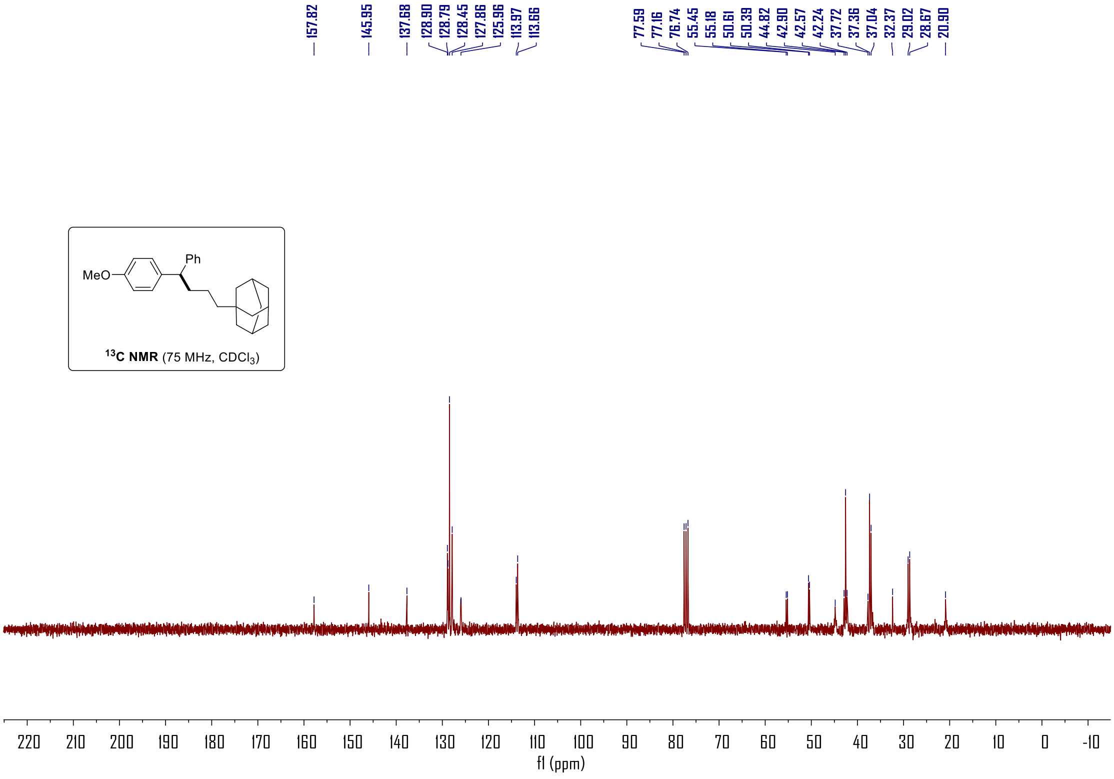

1-Methoxy-4-(1-phenylundec-10-en-1-yl)benzene (3ea)

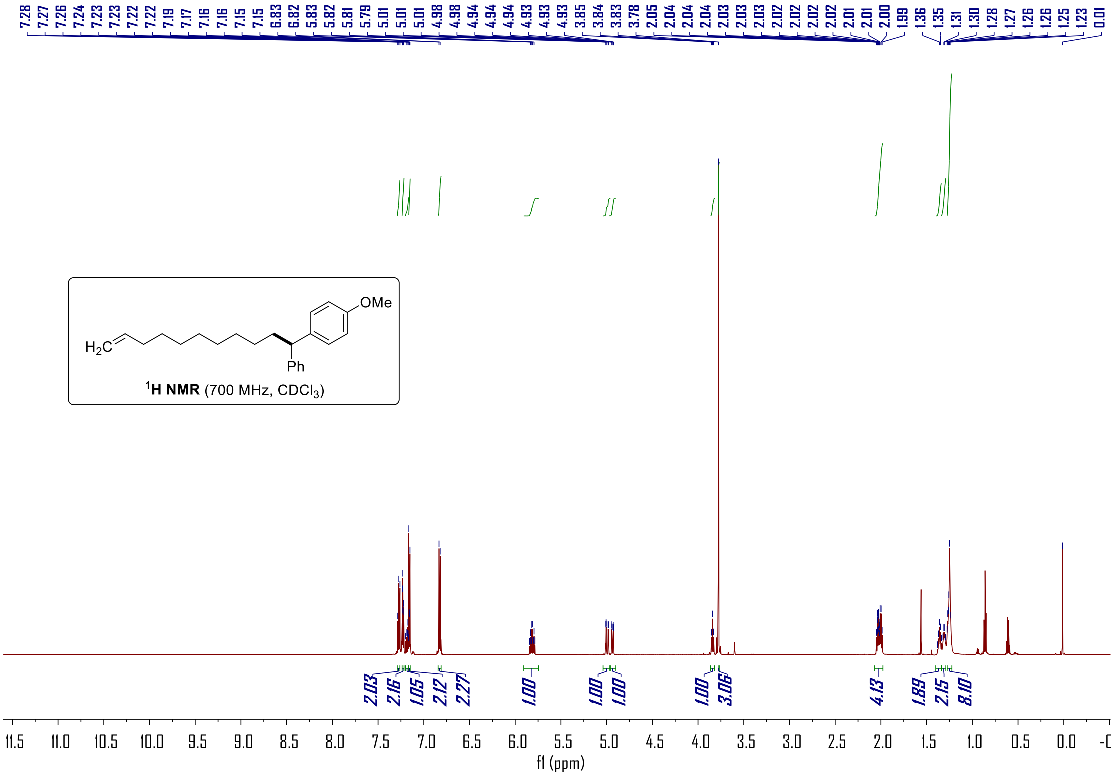

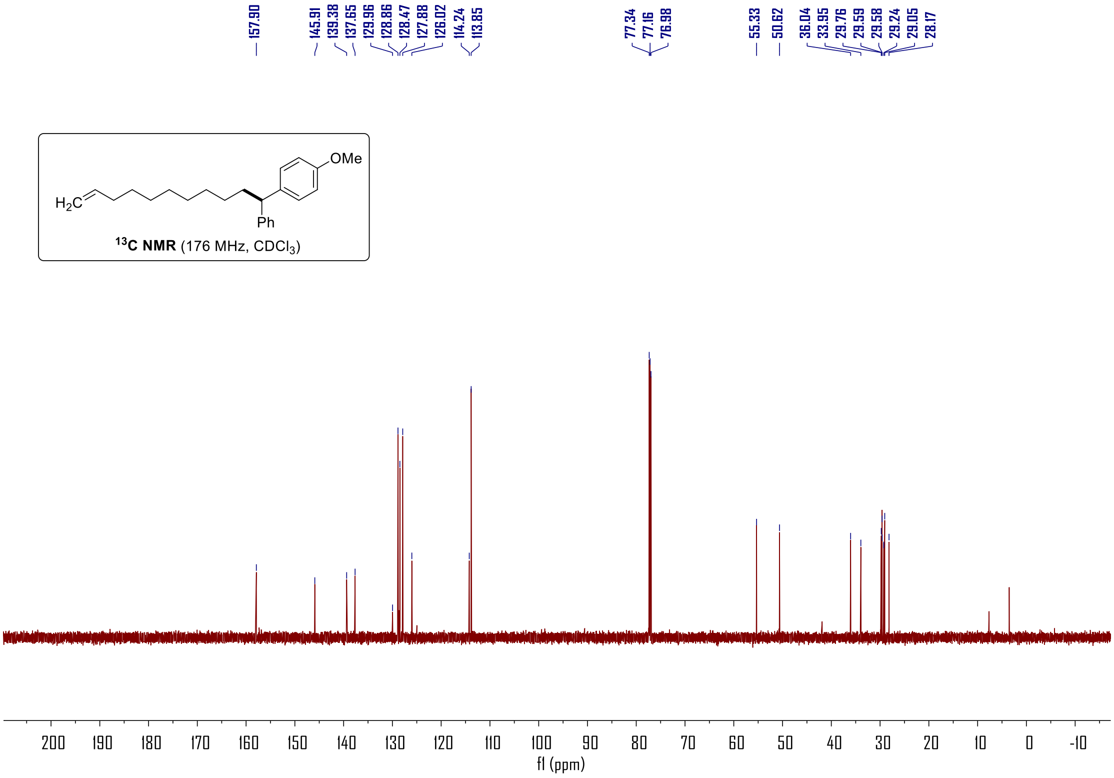

(1-(4-Methoxyphenyl)butane-1,4-diyl)dibenzene (3fa)

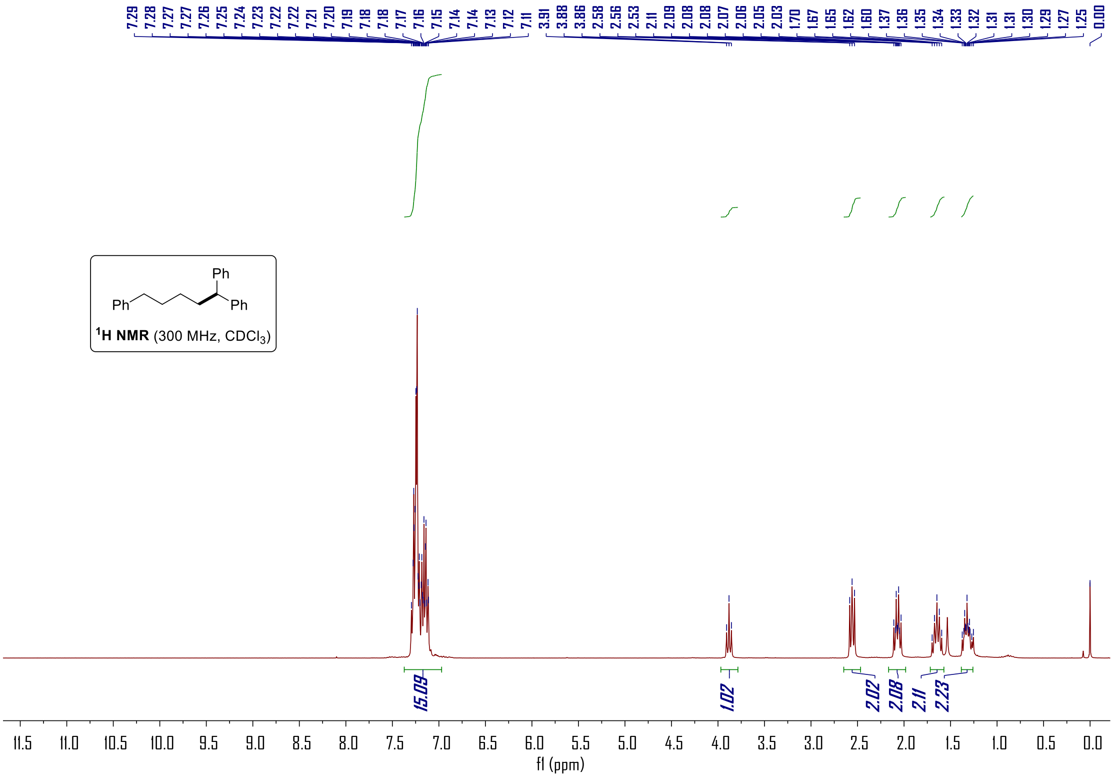

(1-(4-Methoxyphenyl)pentane-1,5-diyl)dibenzene (3ga)

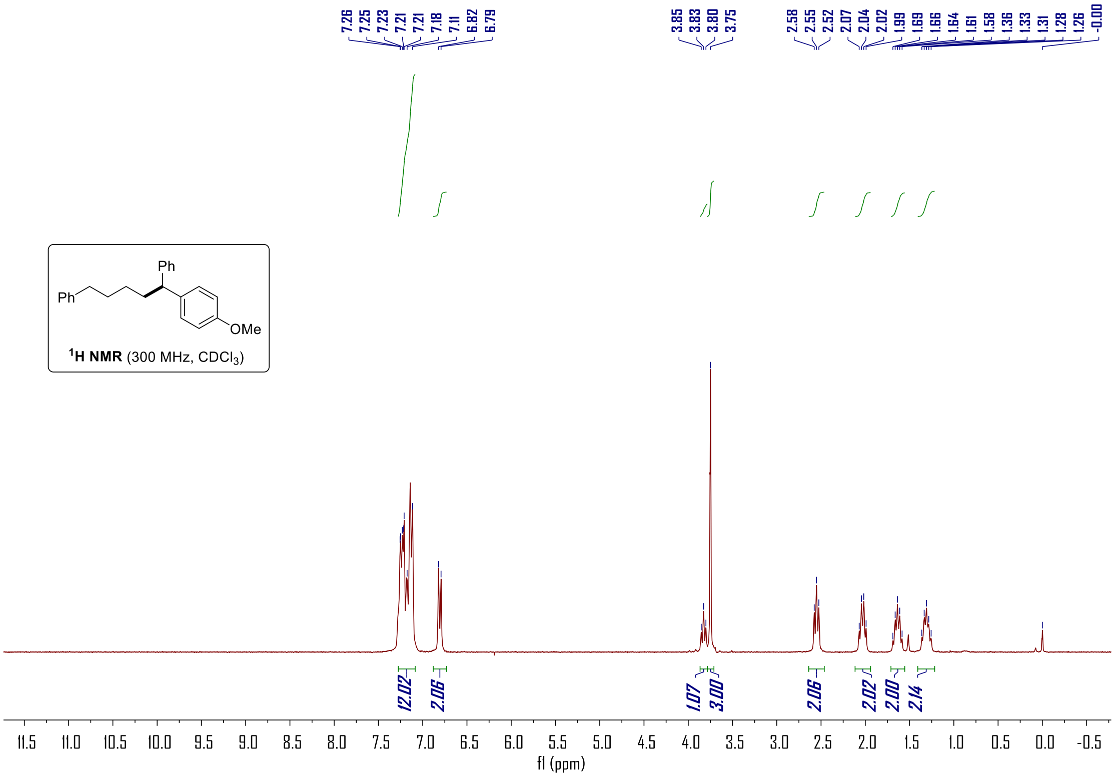

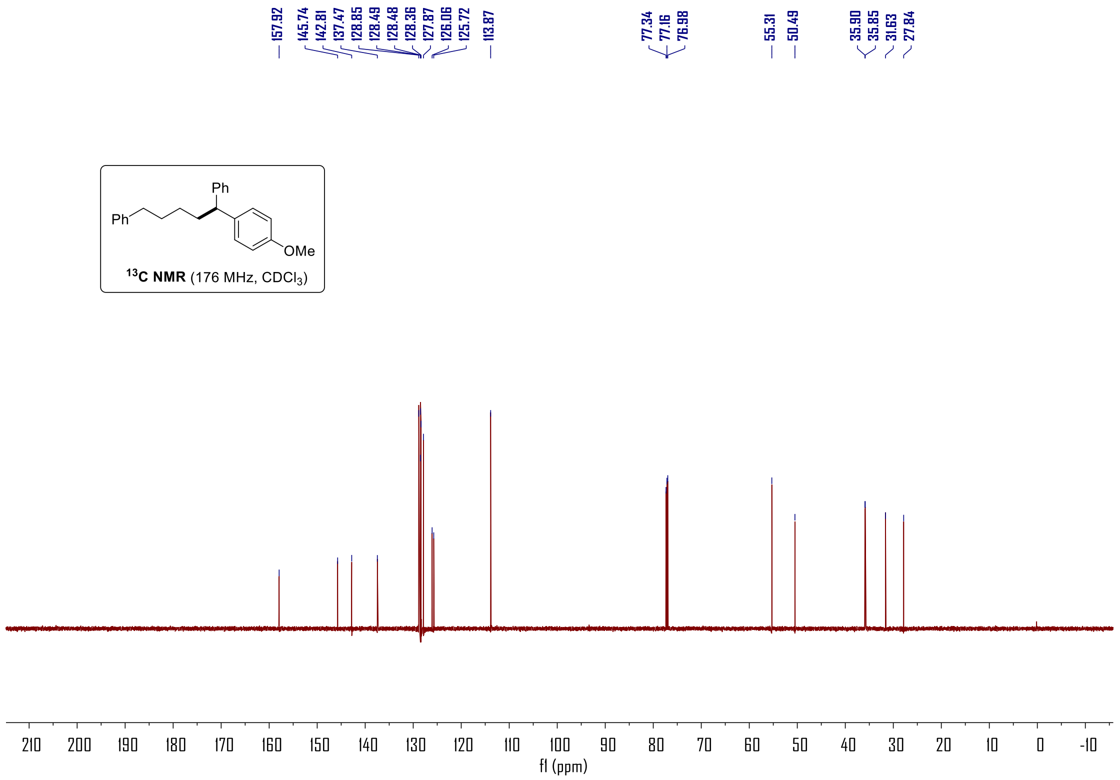

1-(4-Methoxyphenyl)hexane-1,5-diyl)dibenzene (3ha)

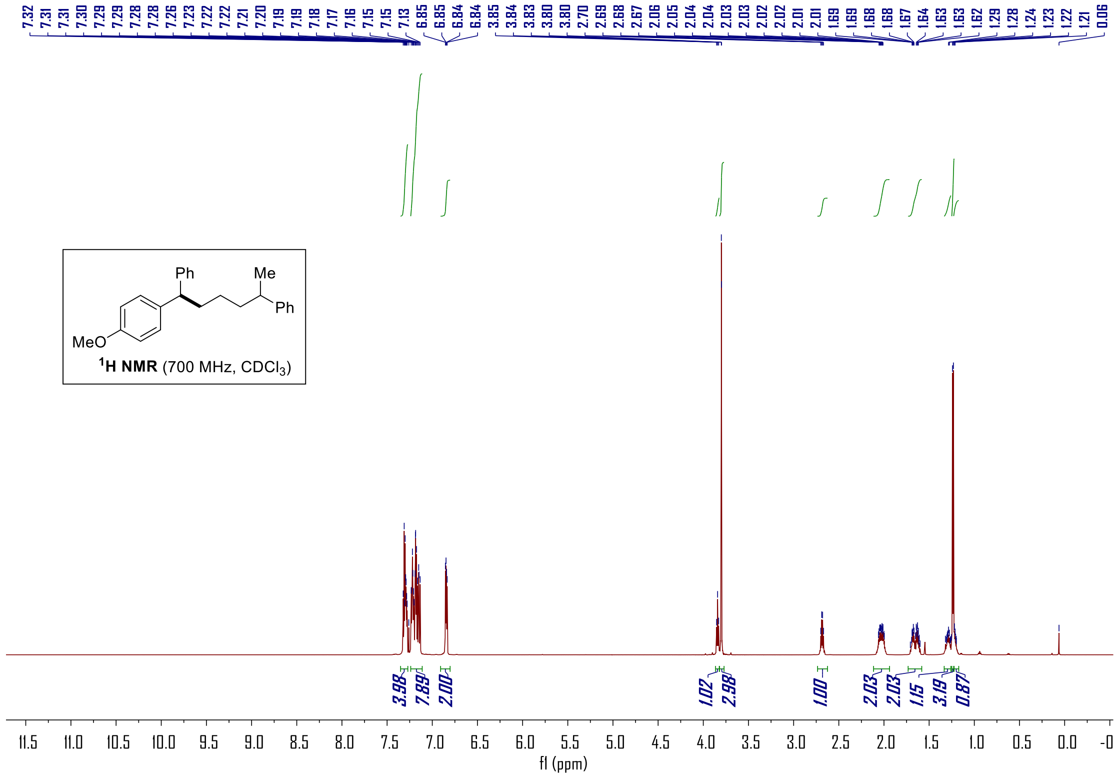

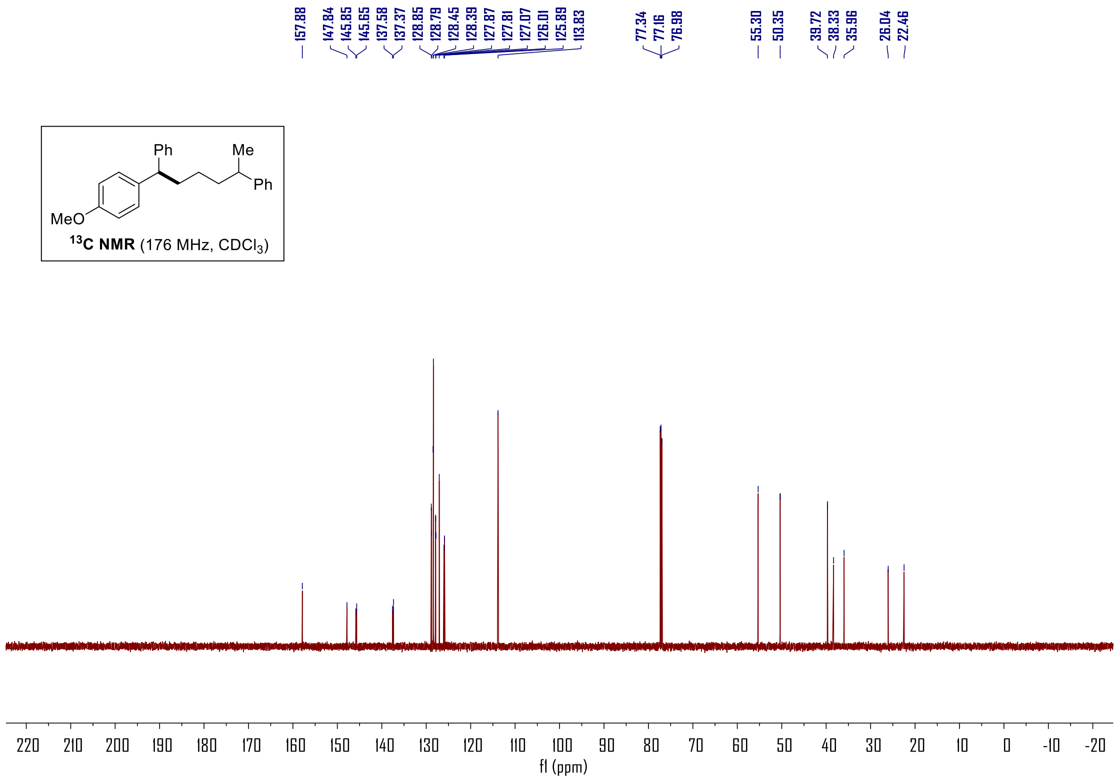

(1-(4-Methoxyphenyl)-4-methylpentane-1,4-diyl)dibenzene (3ia)

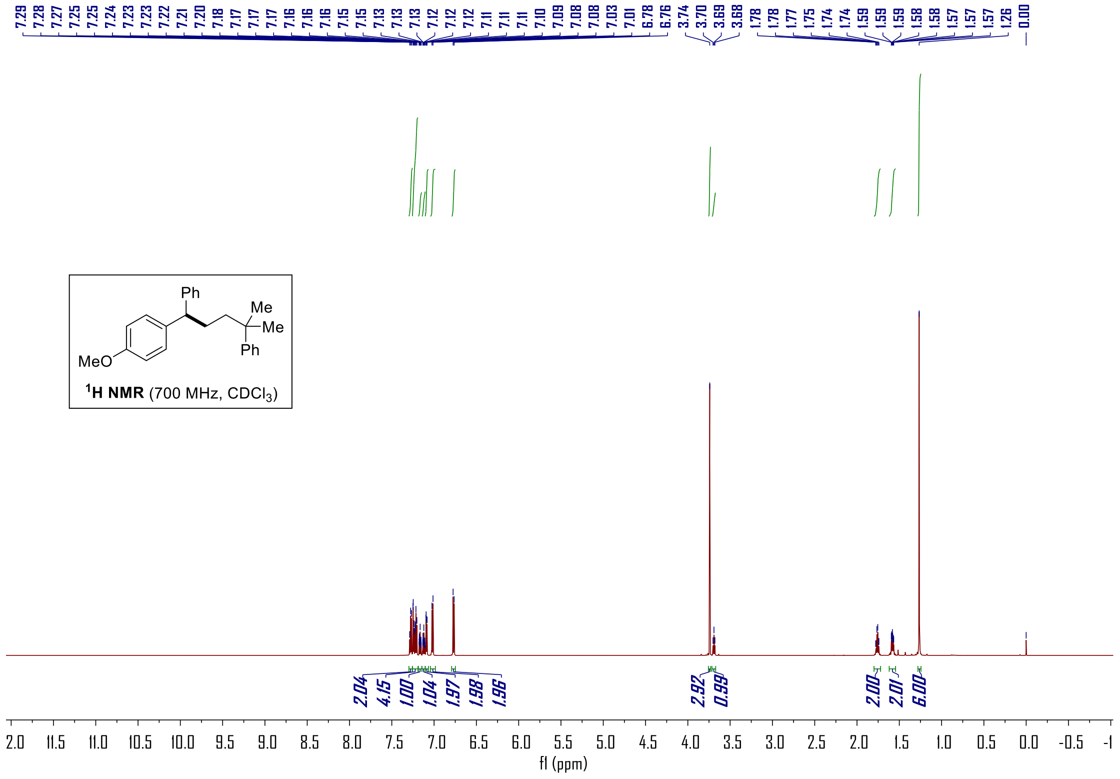

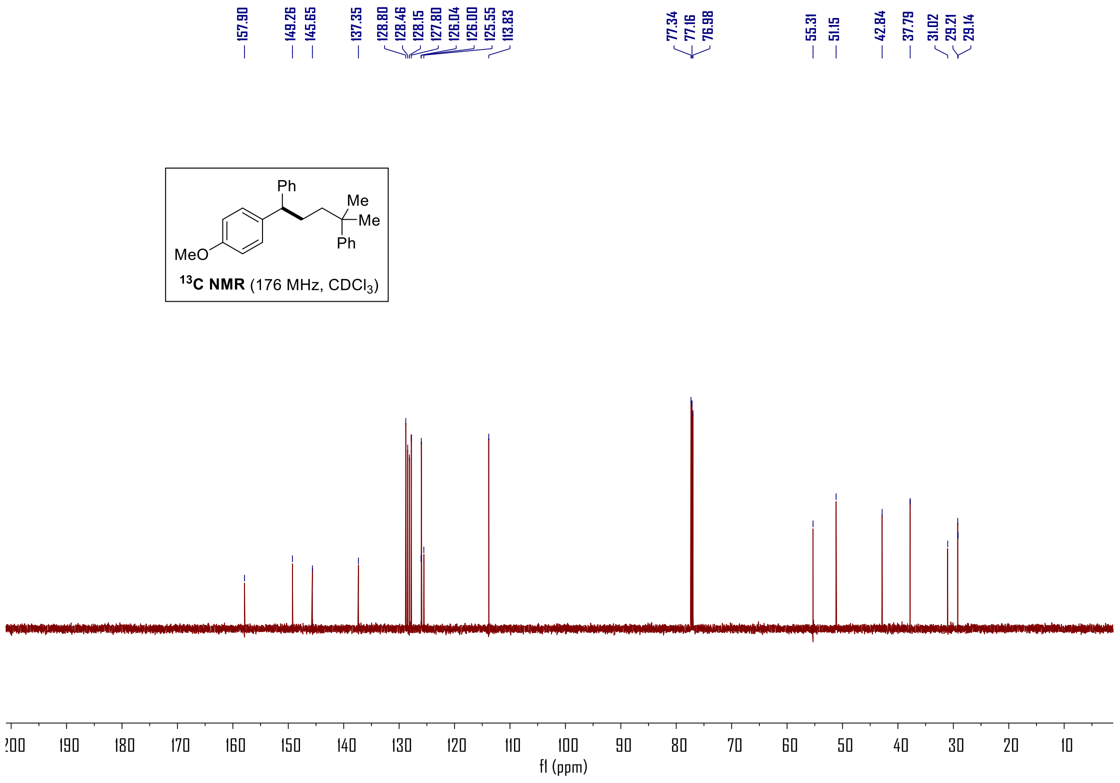

1-Methoxy-4-(1-phenyl-4-(1-phenylcyclopentyl)butyl)benzene (3ja)

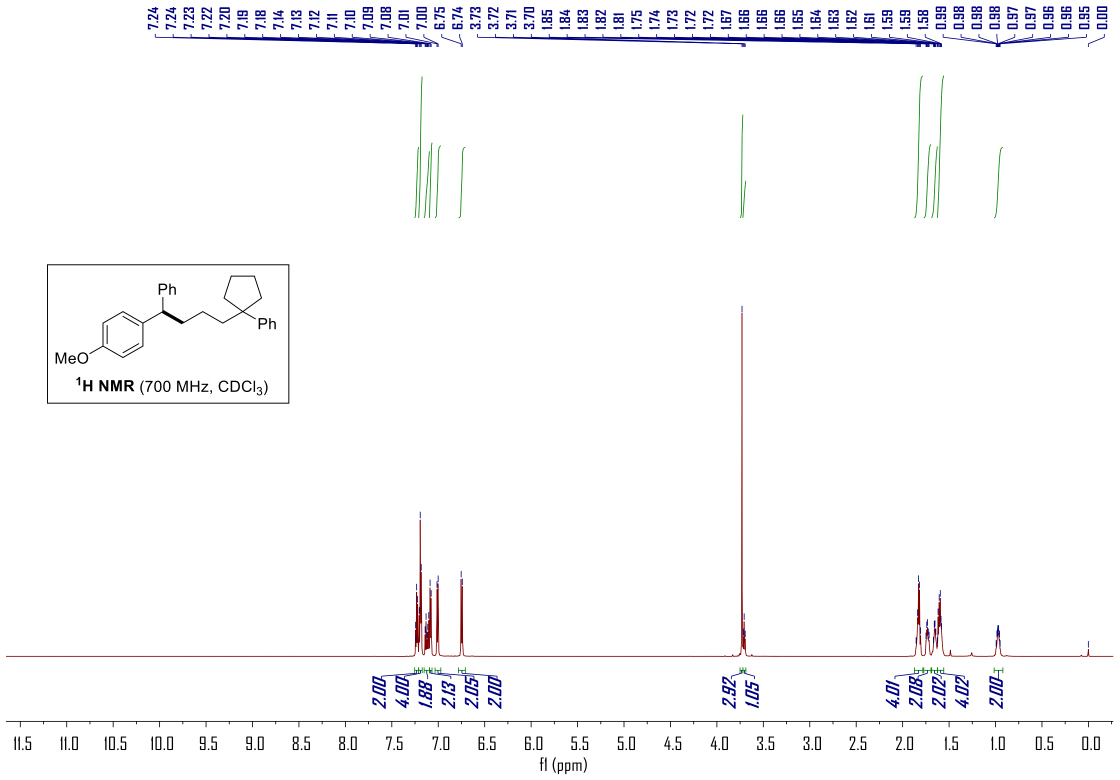

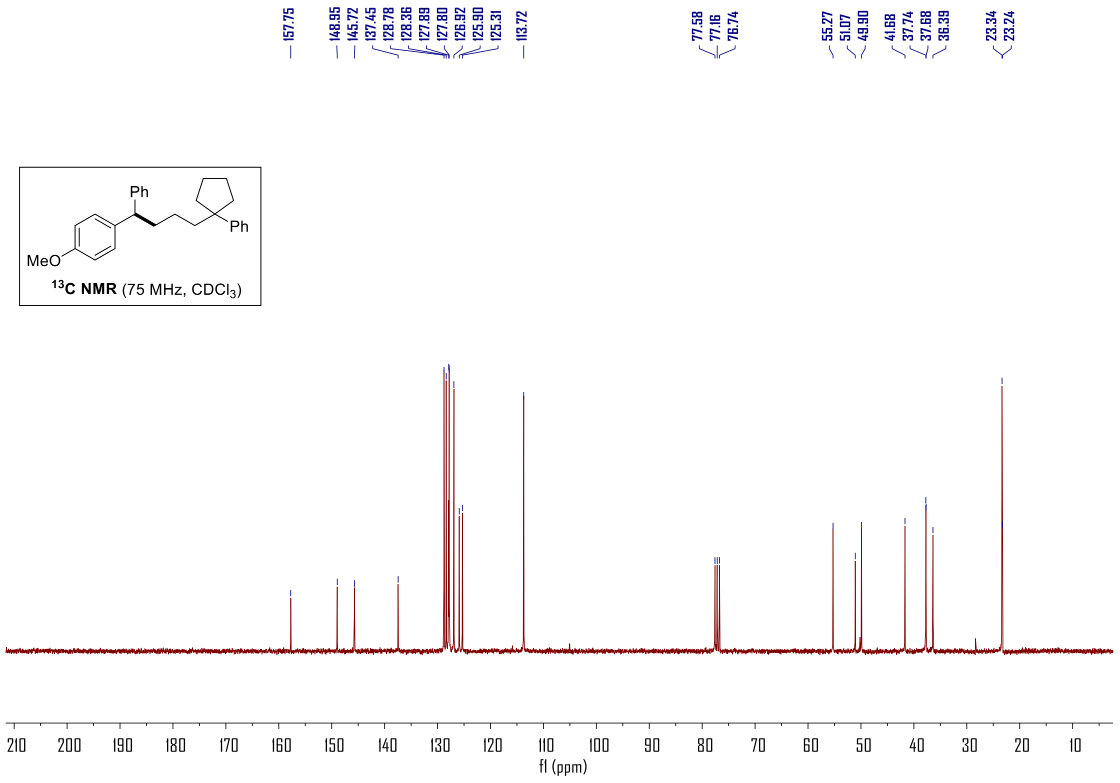

1-Cyclohexyl-2-(4-methoxyphenyl)ethane-1,2-diyl)dibenzene (3ka)

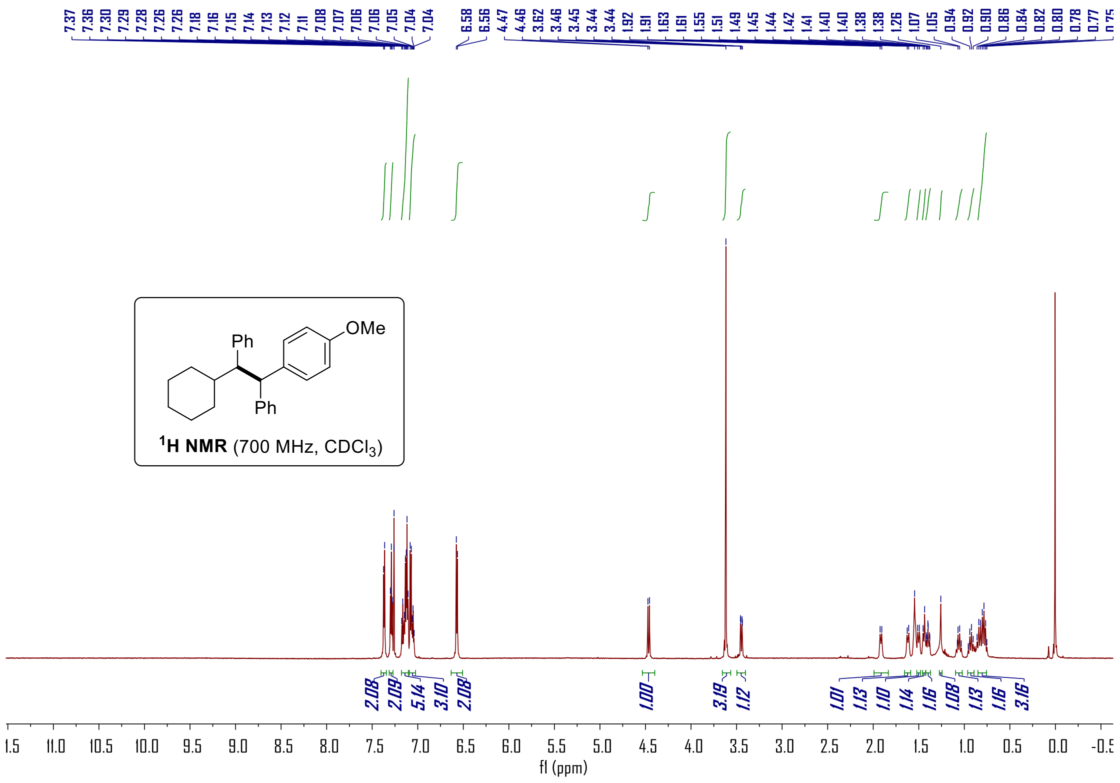

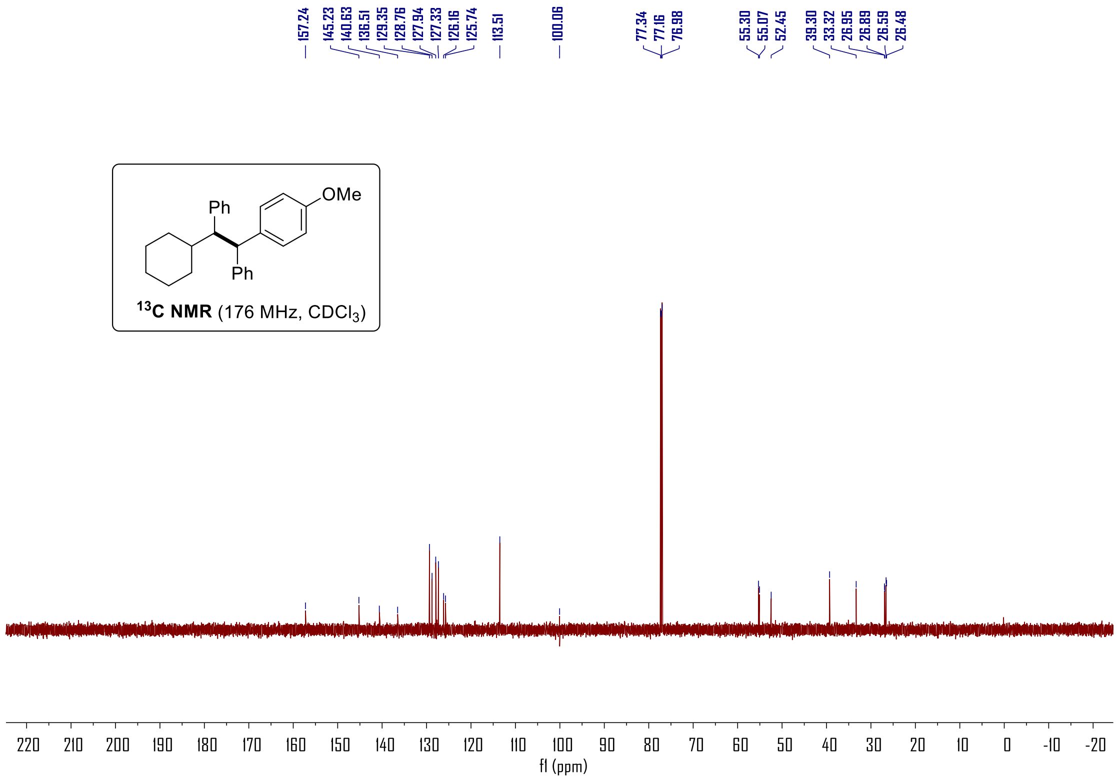

Nonane-1,1-diyldibenzene (3ab)

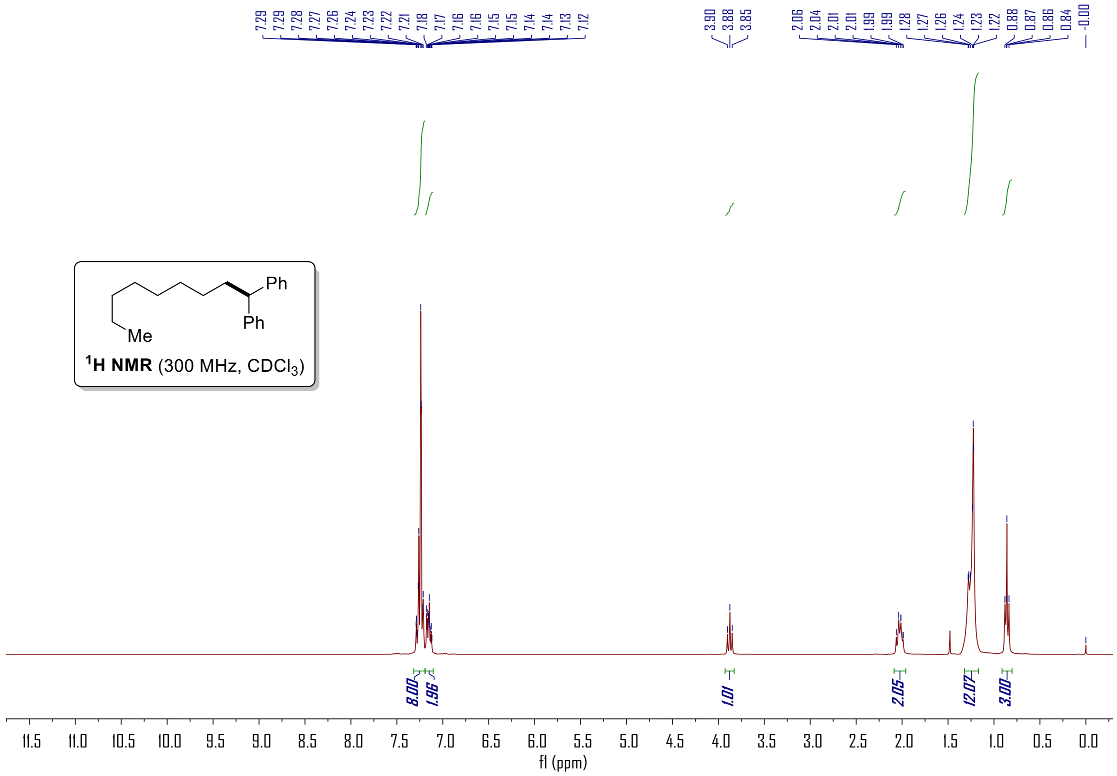

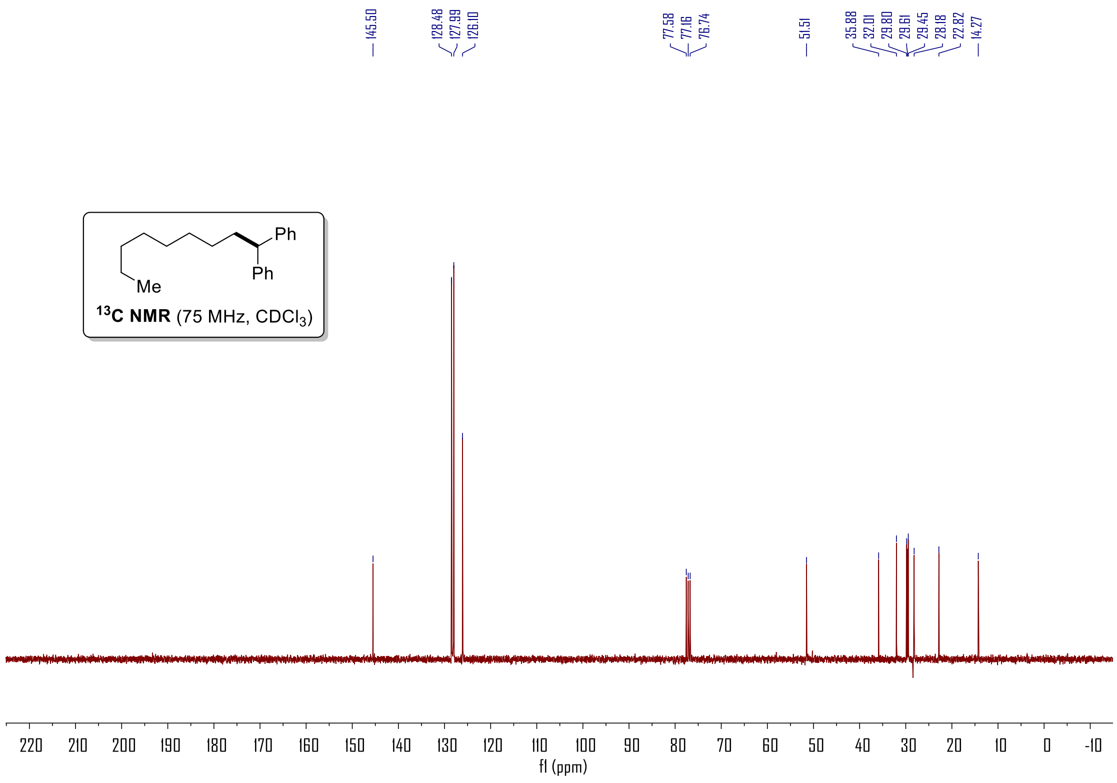

Undecane-1,1-diyldibenzene (3bb)

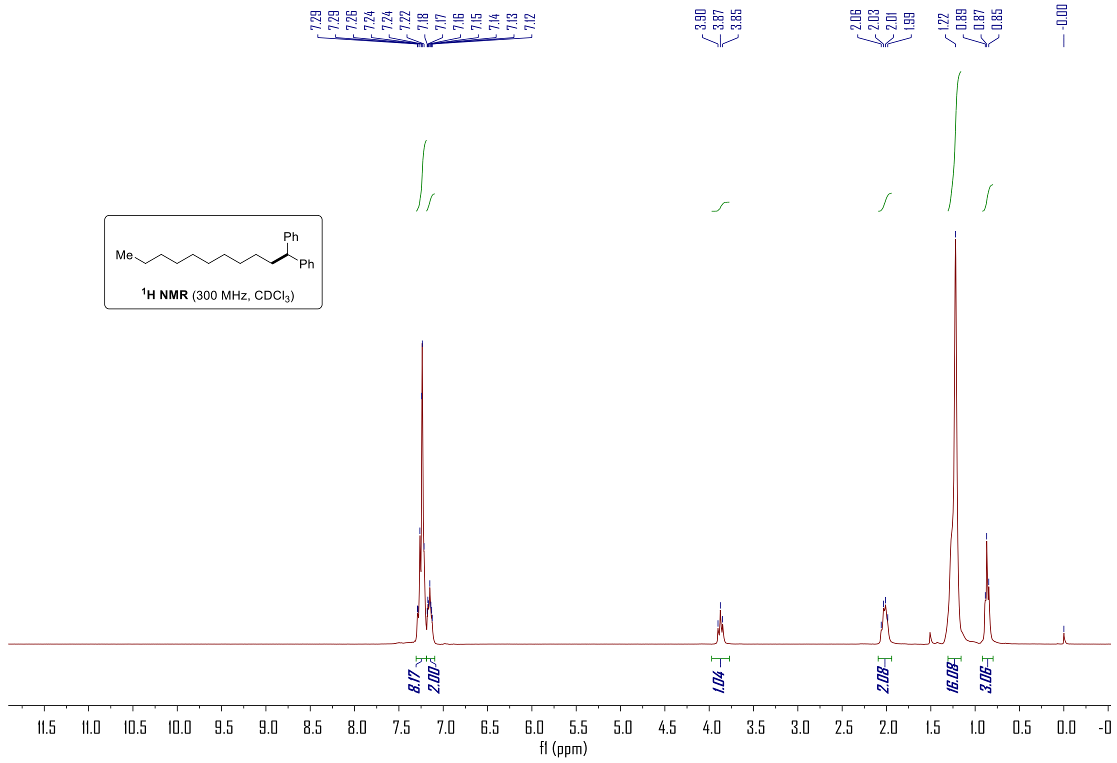

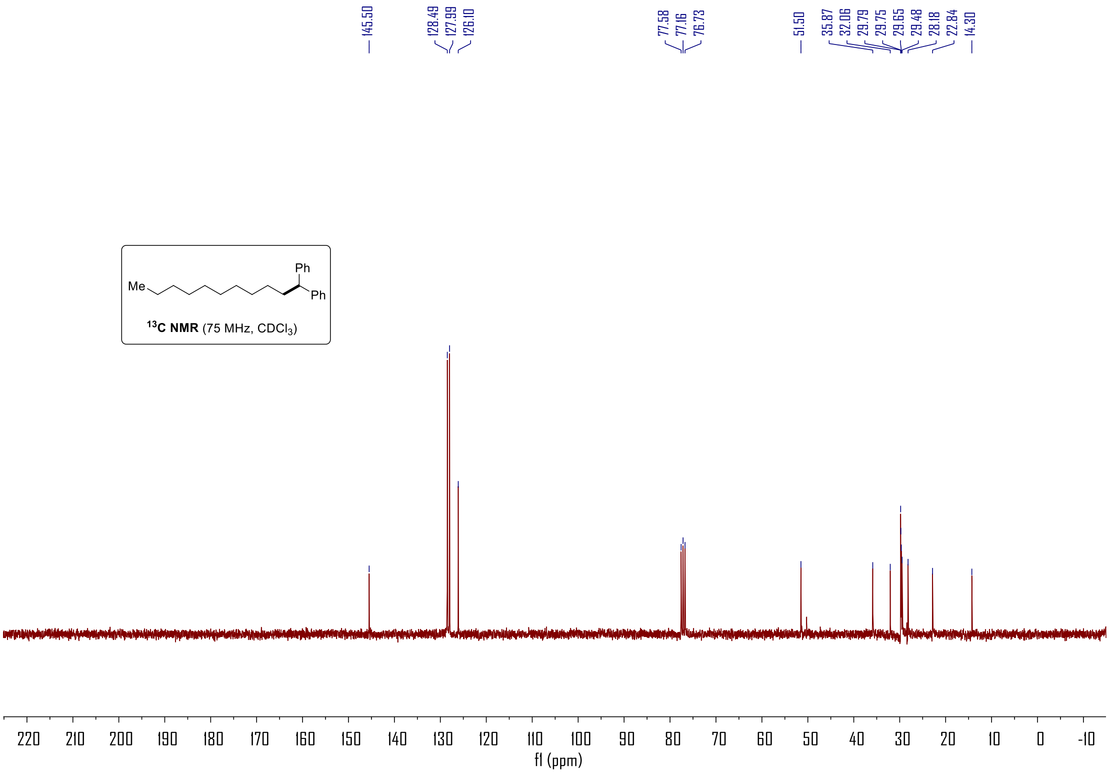

Nonadecane-1,1-diyldibenzene (3cb)

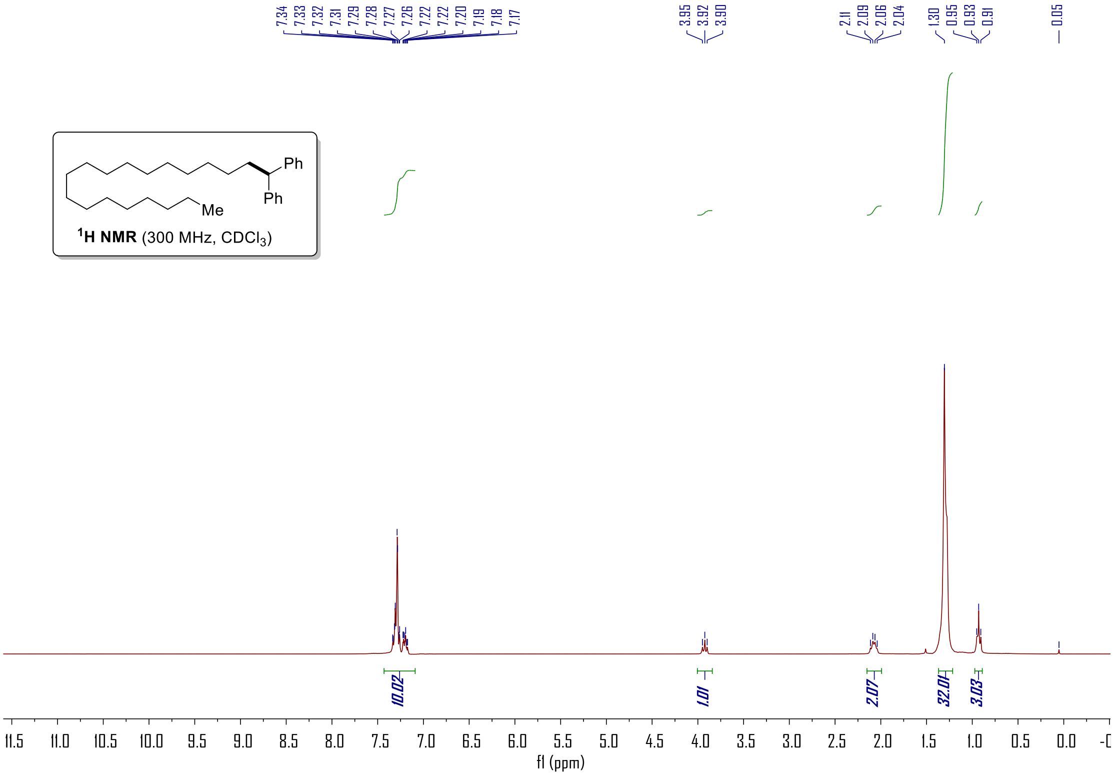

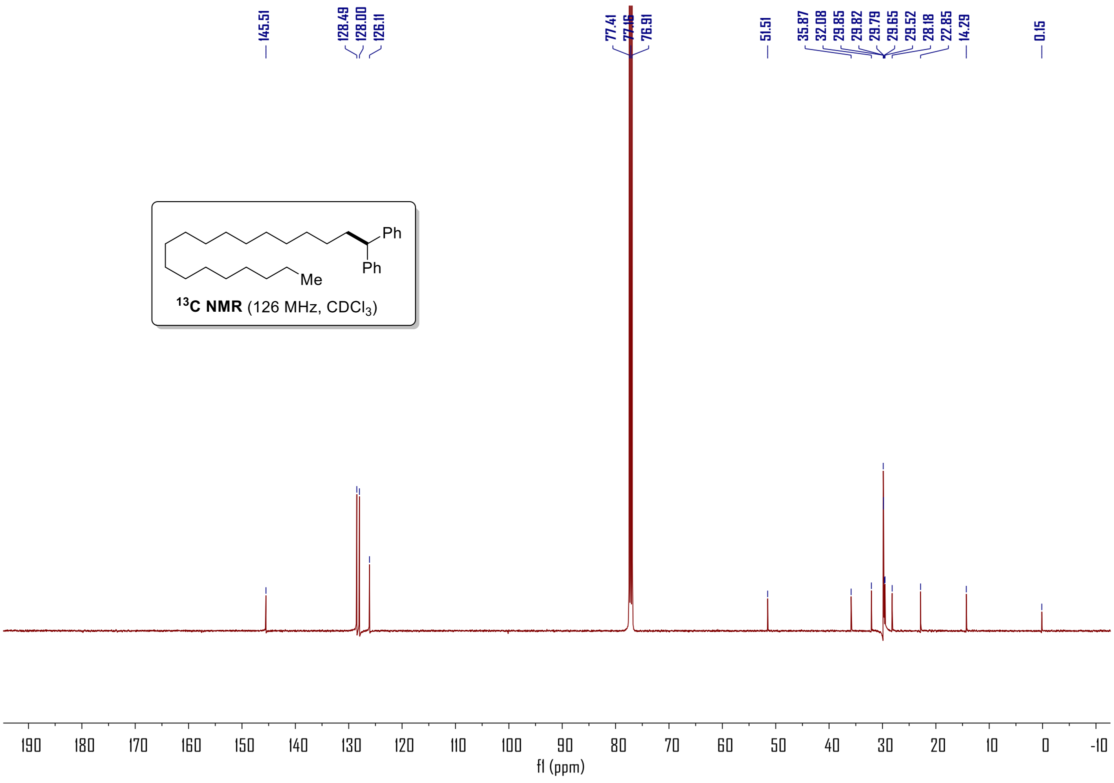

1-(4,4-Diphenylbutyl)adamantane (3db)

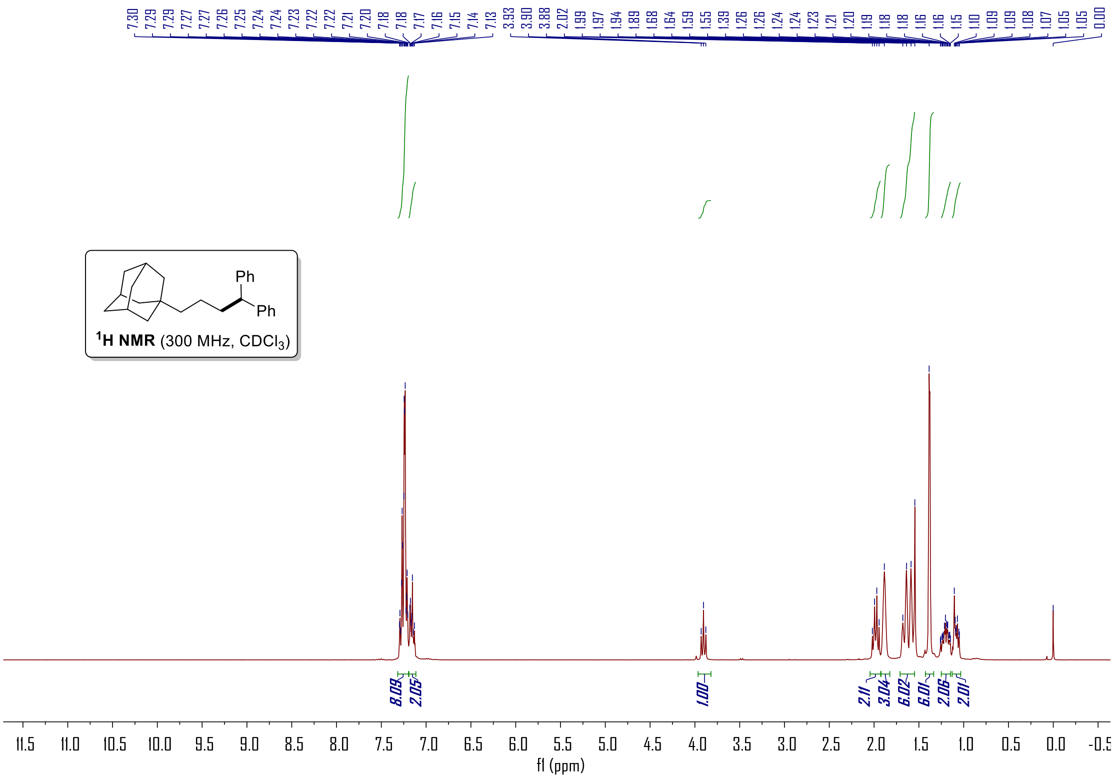

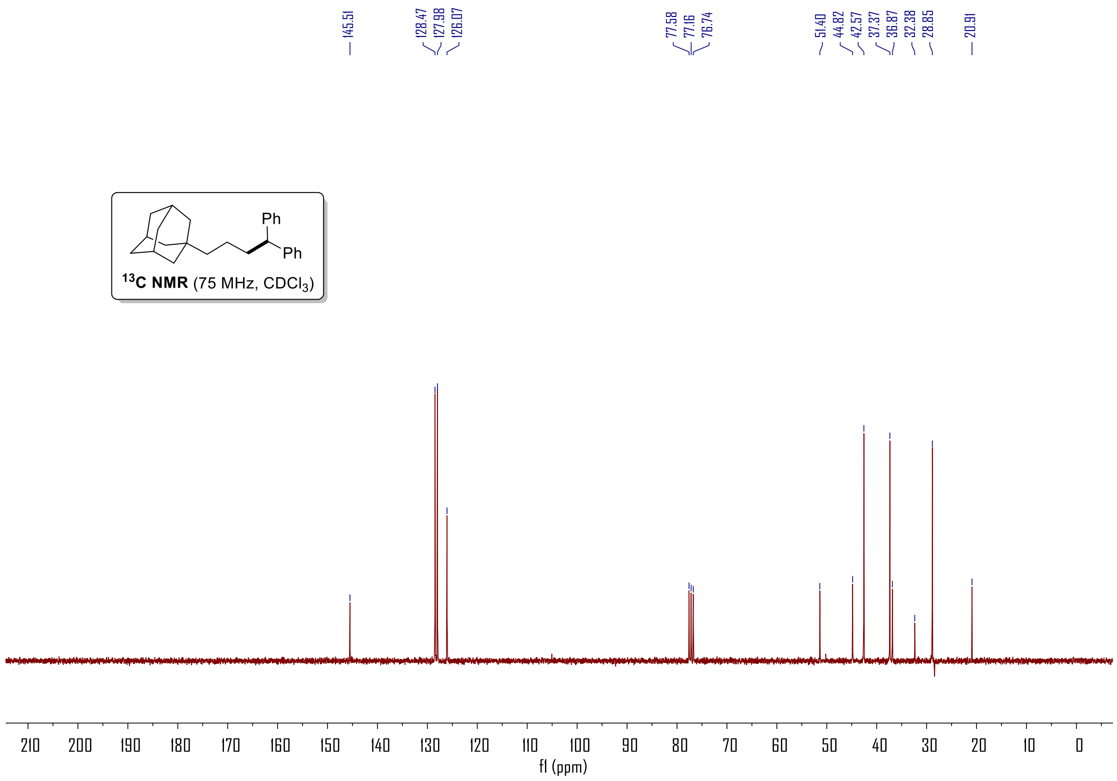

Undec-10-ene-1,1-diyldibenzene (3eb)

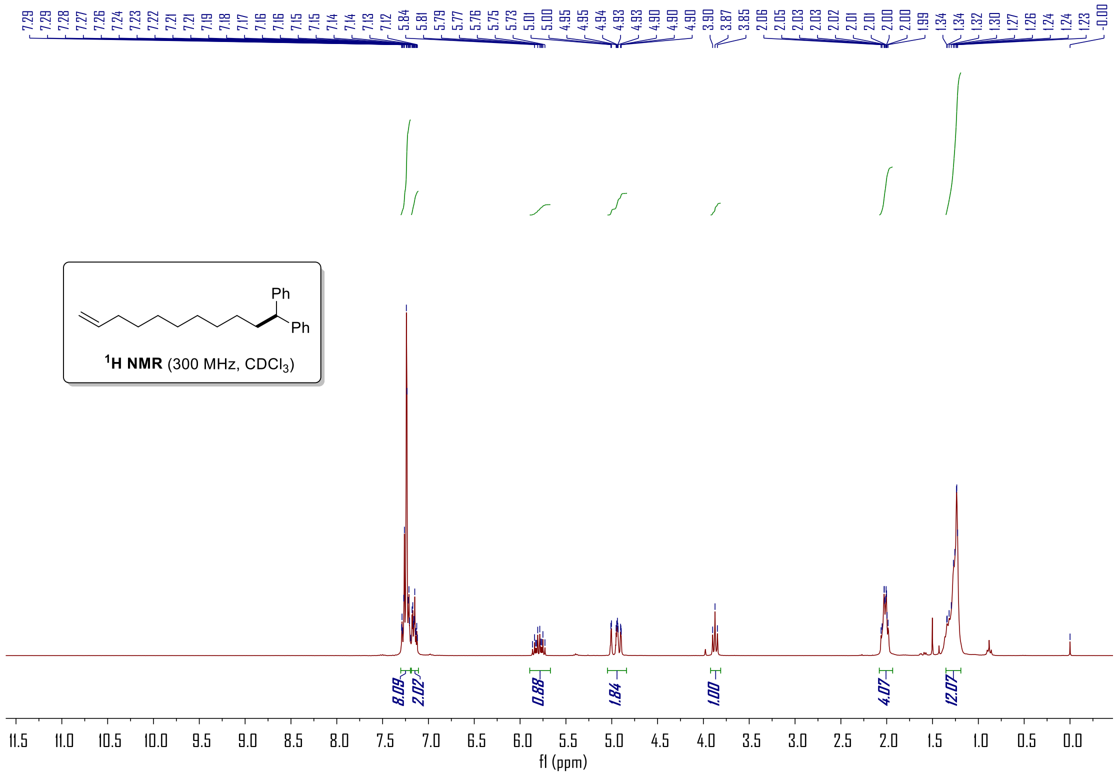

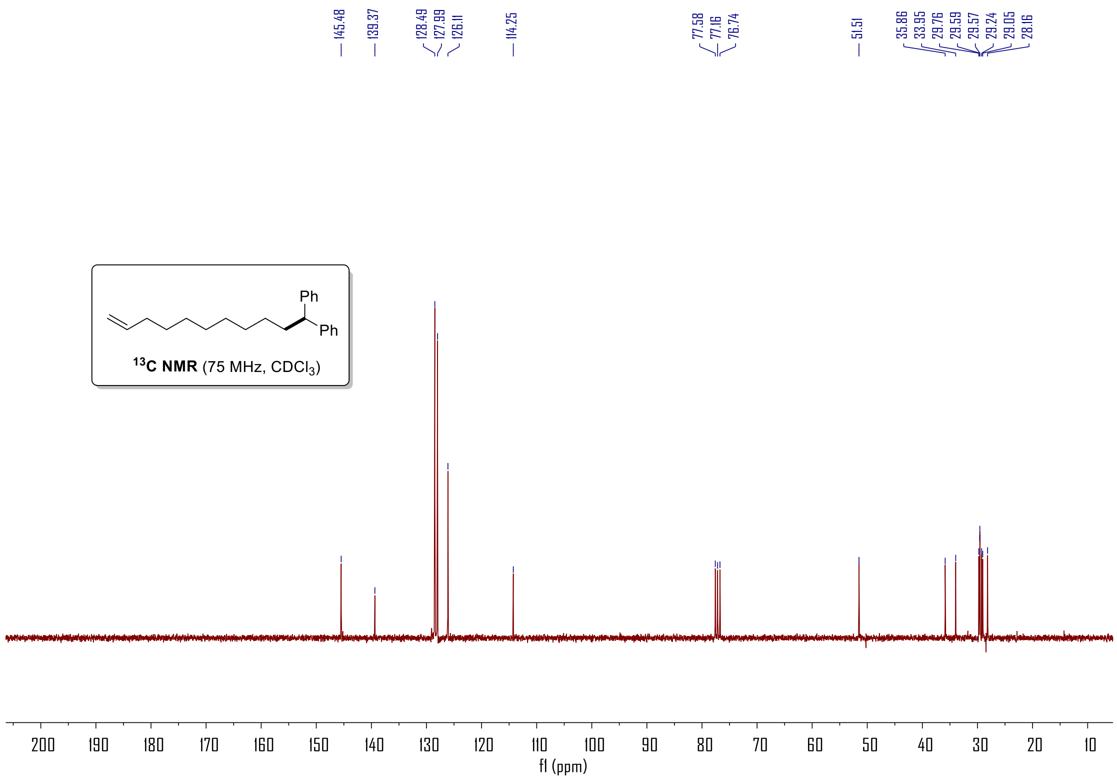

Butane-1,1,4-triyltribenzene (3fb)

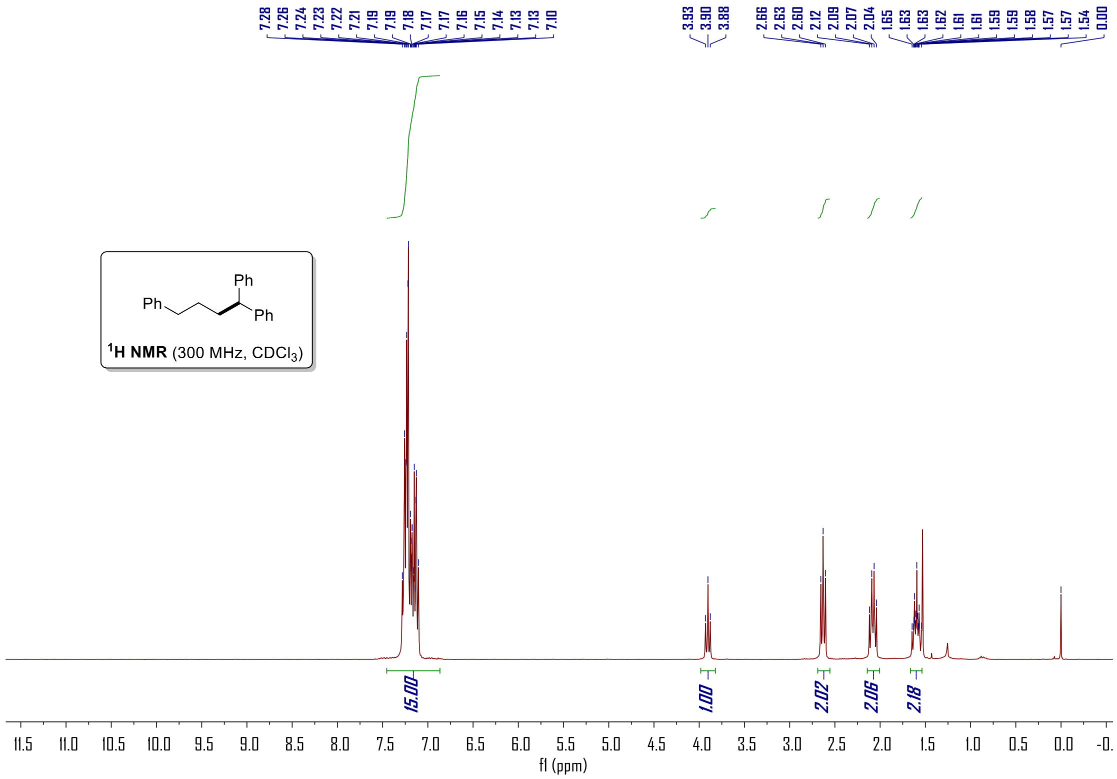

Pentane-1,1,5-triyltribenzene (3gb)

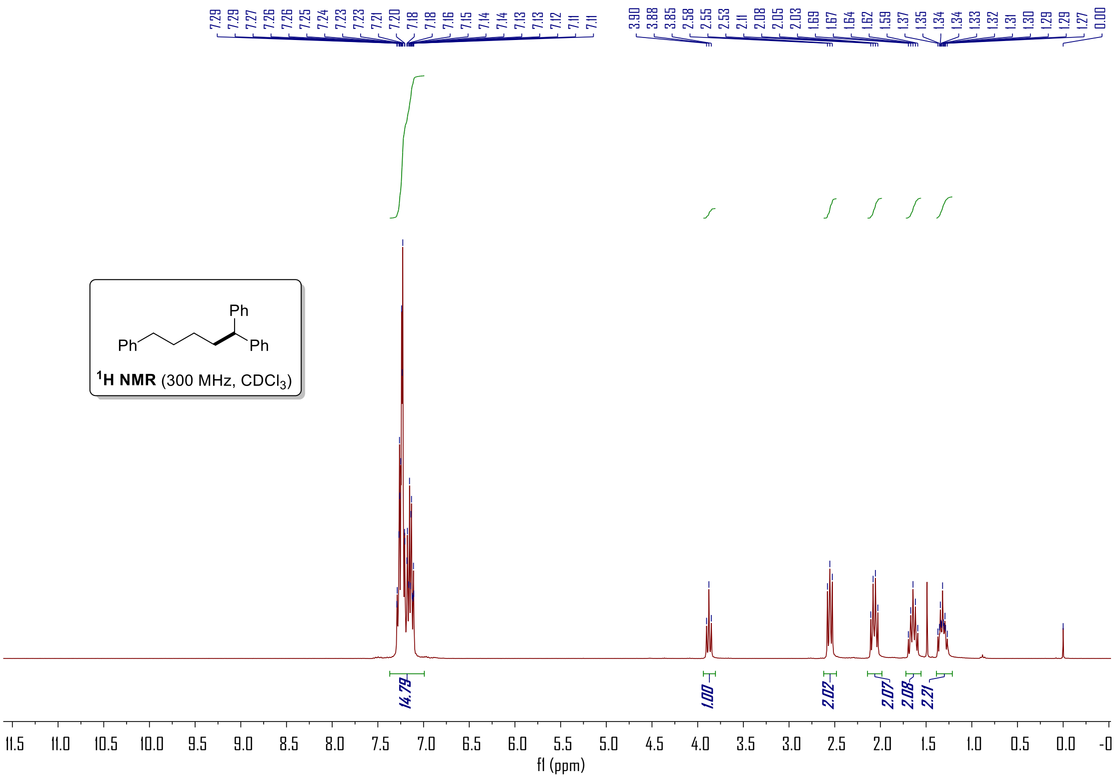

Hexane-1,1,5-triyltribenzene (3hb)

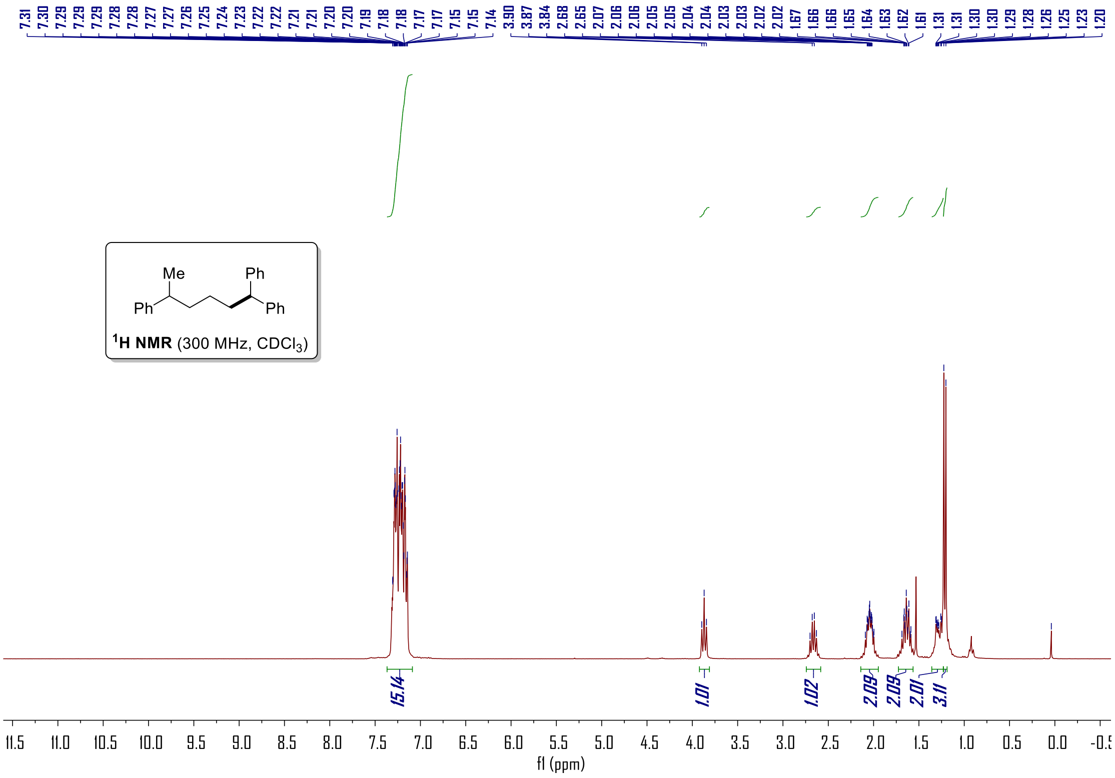

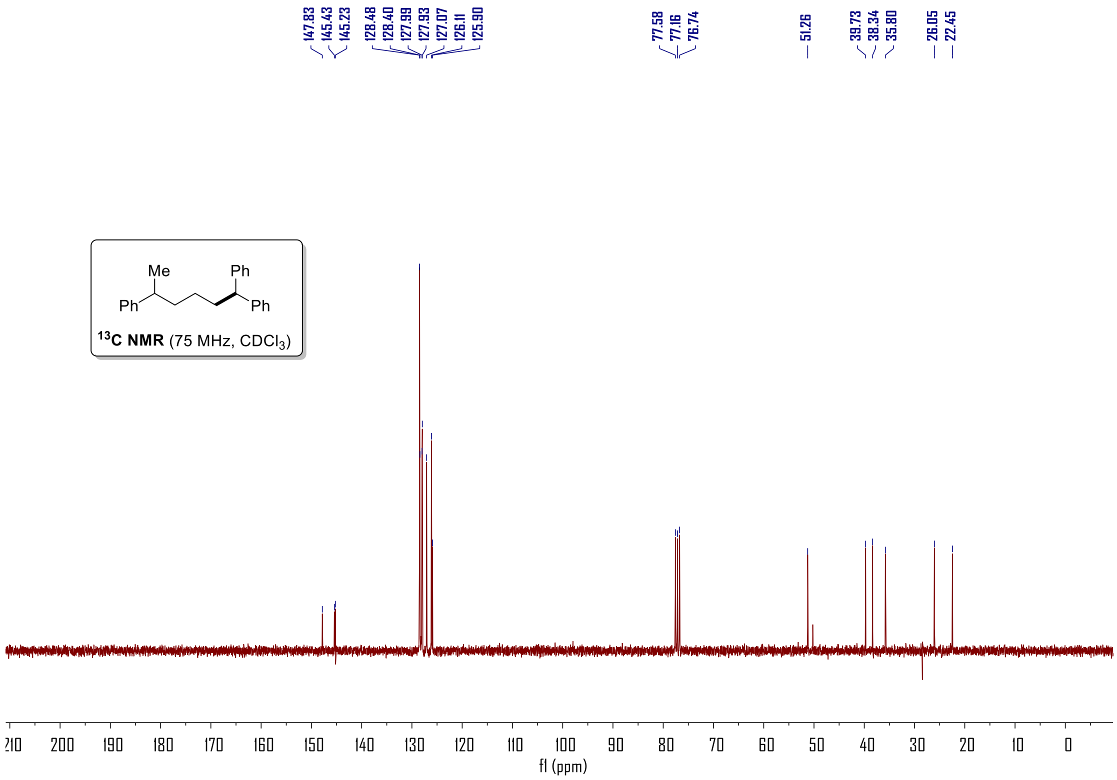

4-(2,2-Diphenylethyl)-biphenyl (3mb)

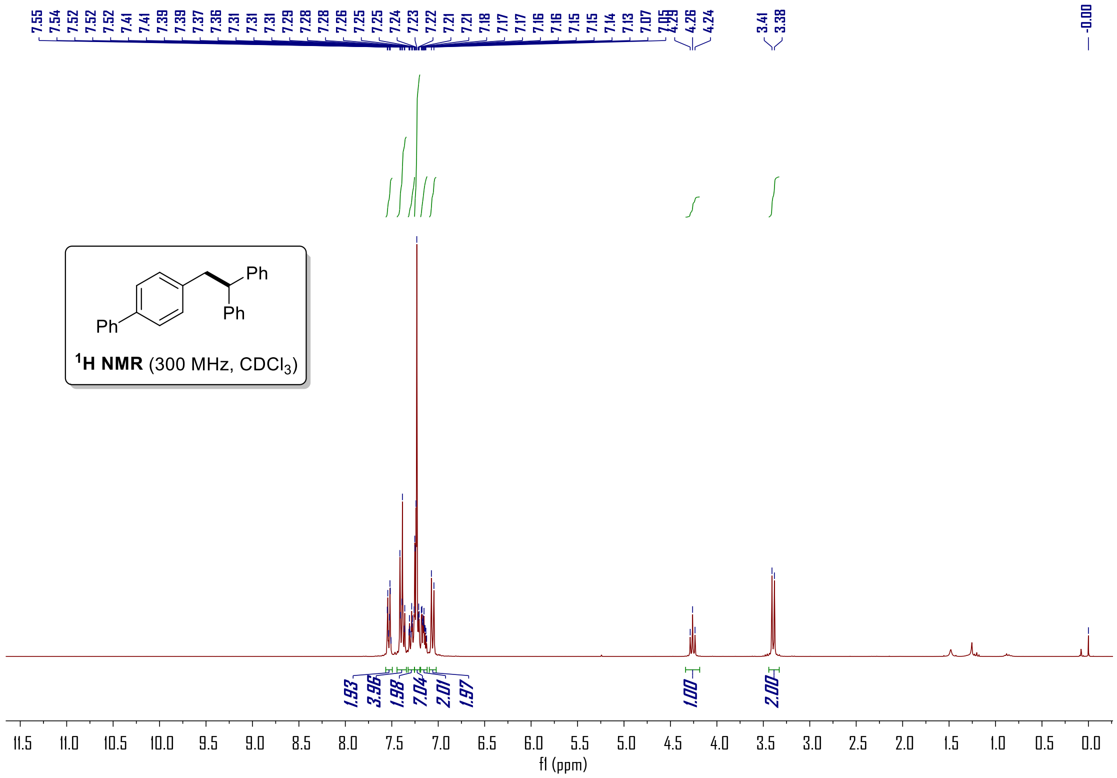

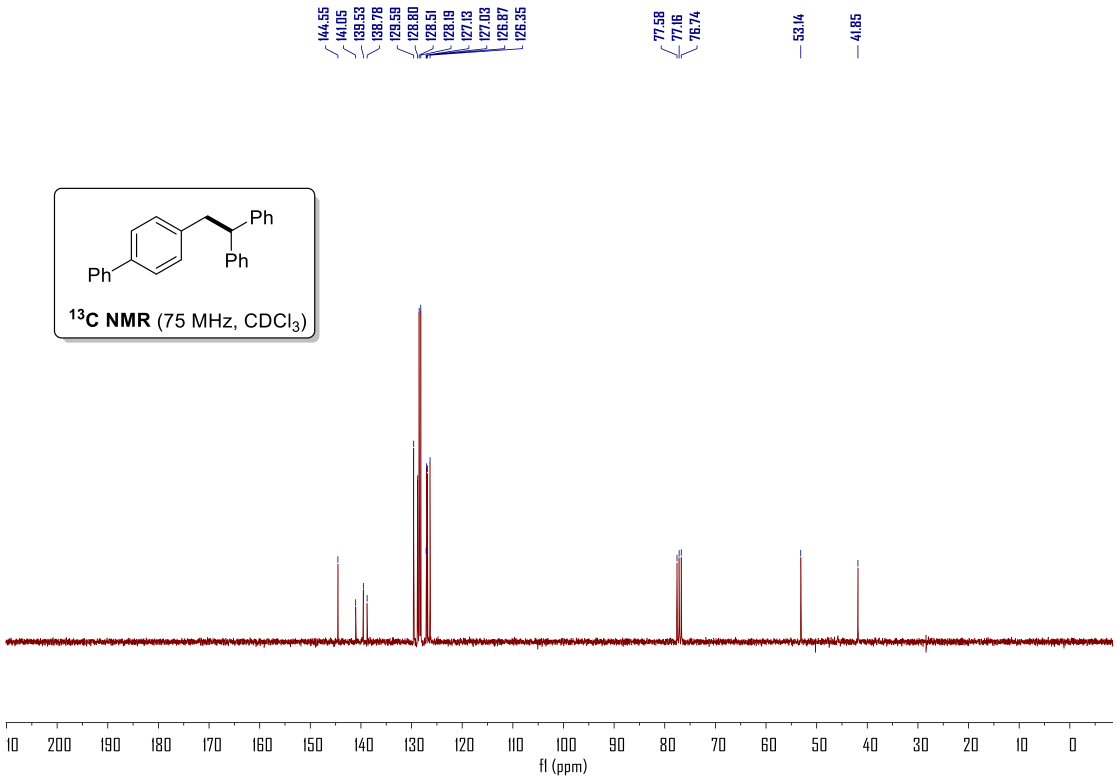

(2-(4-(Trifluoromethoxy)phenyl)ethane-1,1-diyl)dibenzene (3nb)

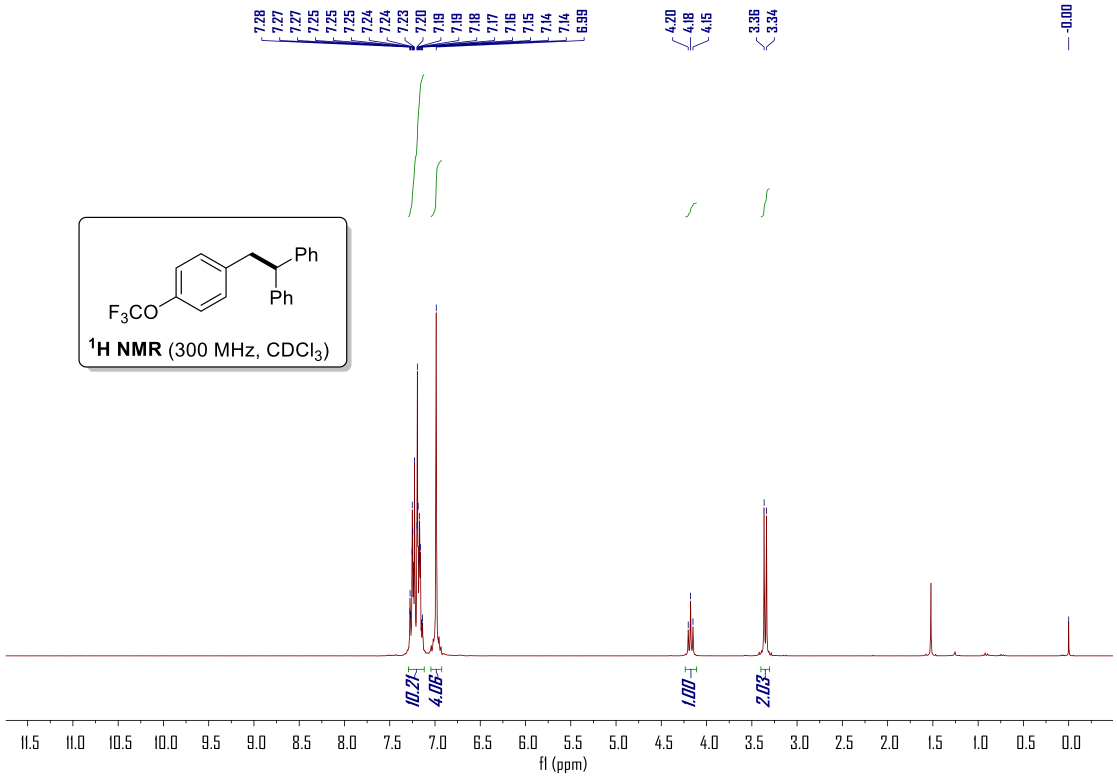

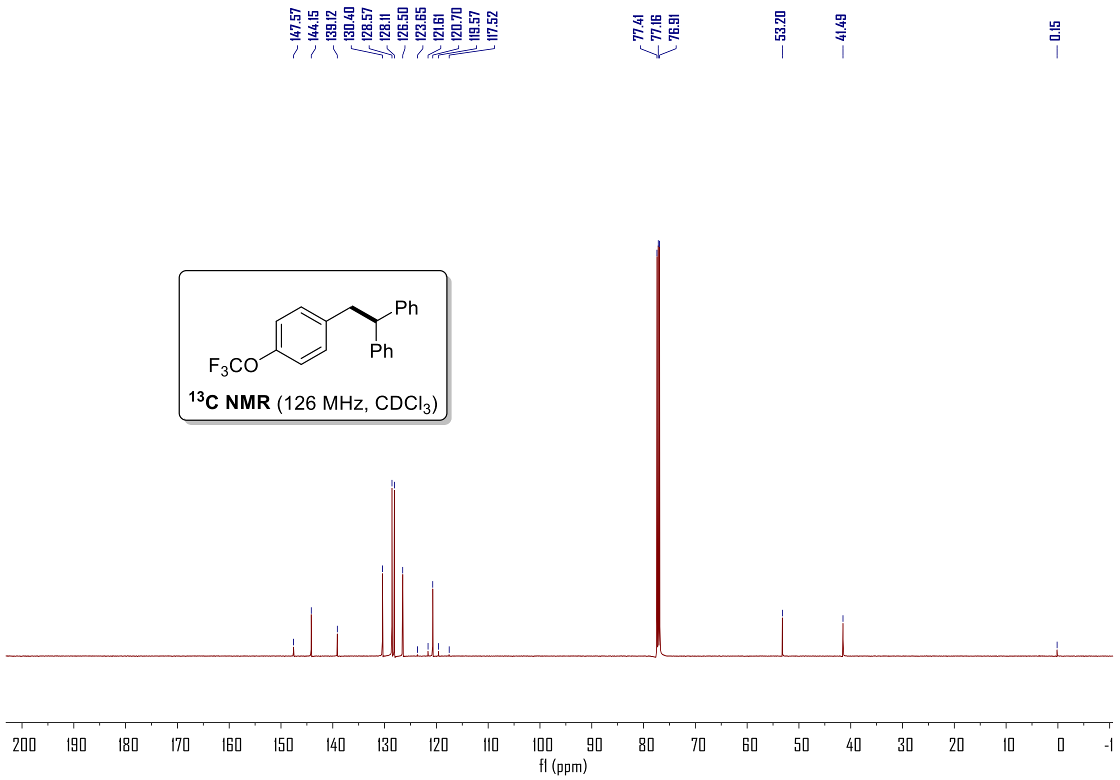
147.57
144.15
139.12
130.40
128.57
128.11
126.50
123.65
121.61
120.70
119.57
117.52
77.41
77.16
76.91
53.20
41.49
0.15
F3CO
Ph
Ph
13C NMR (126 MHz, CDCl3)
fl (ppm)

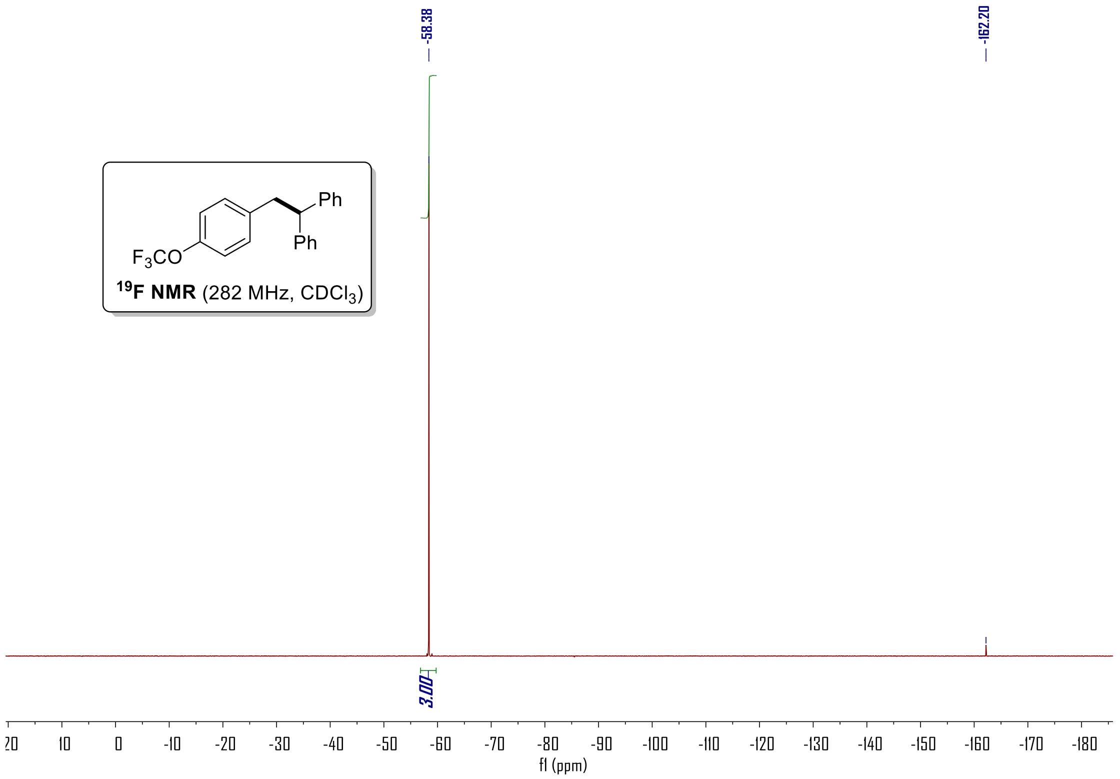
-58.38
-162.20
F3CO
Ph
Ph
19F NMR (282 MHz, CDCl3)
3.00
fl (ppm)

1-(2,2-Diphenylethyl)naphthalene (3ob)

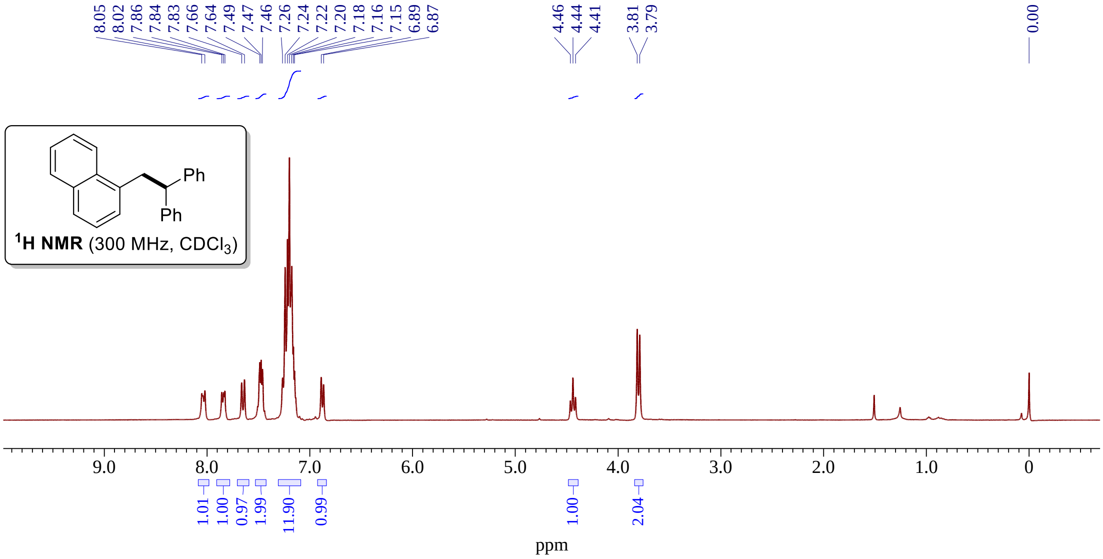

2-(2,2-Diphenylethyl)naphthalene (3pb)

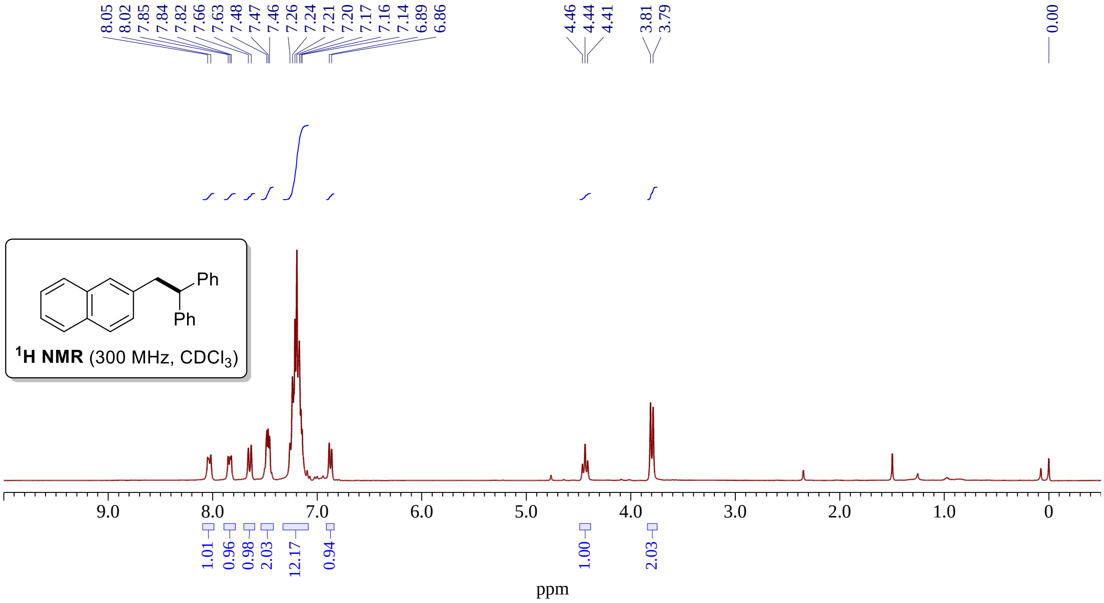

1-Methyl-2-(1-phenylnonyl)benzene (3ac)

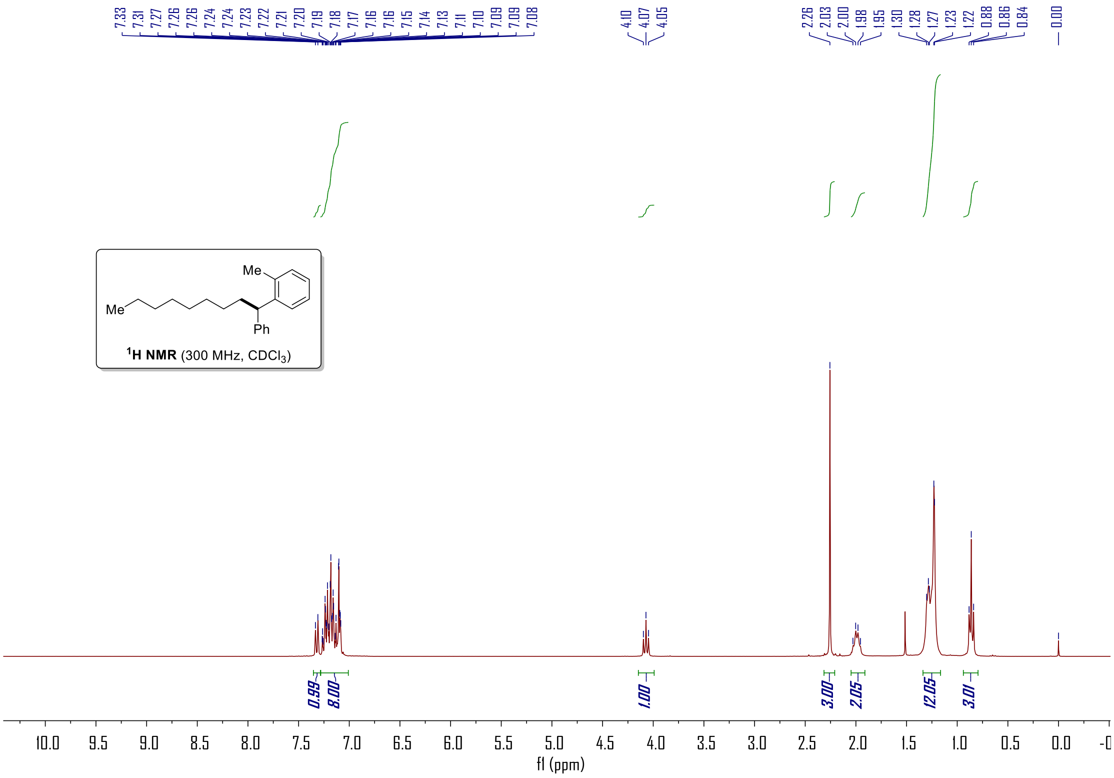

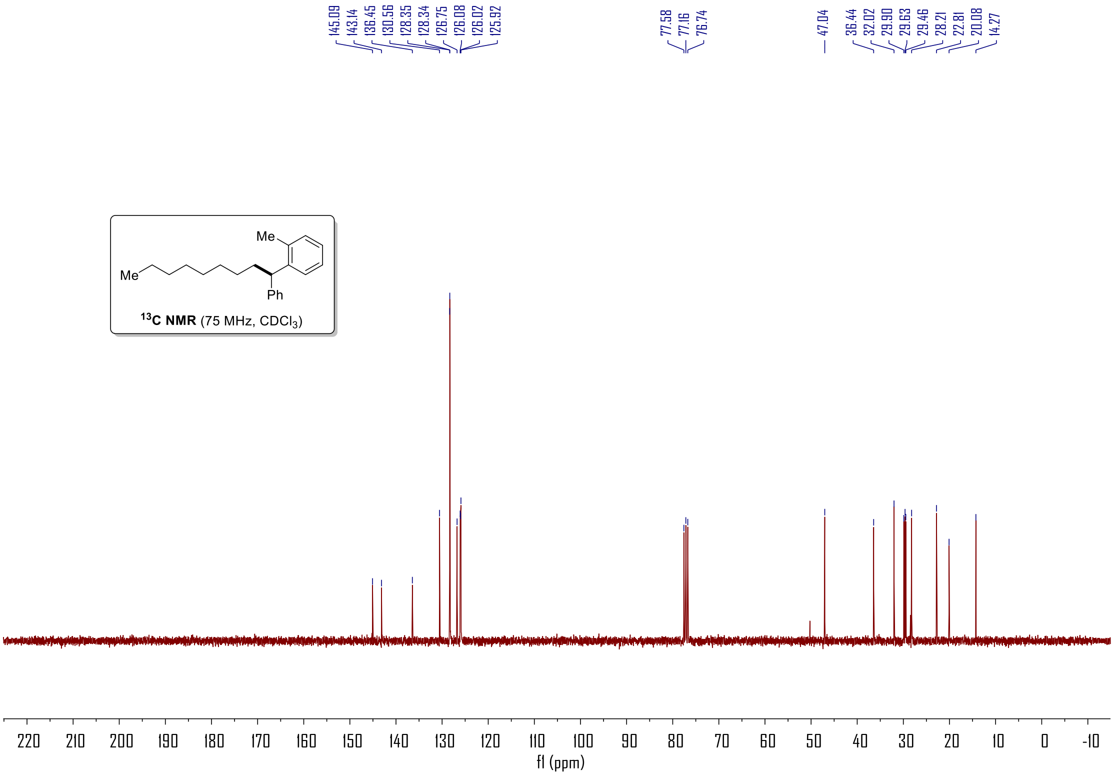

1-Methoxy-2-(1-phenylnonyl)benzene (3ad)

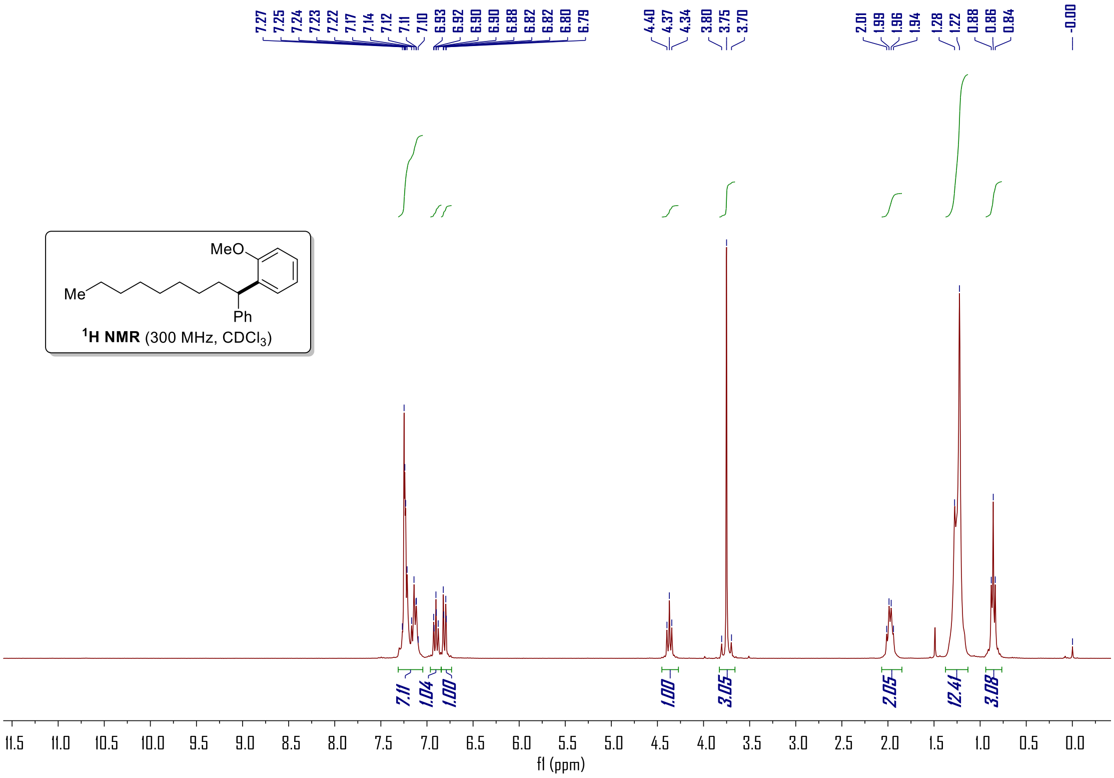

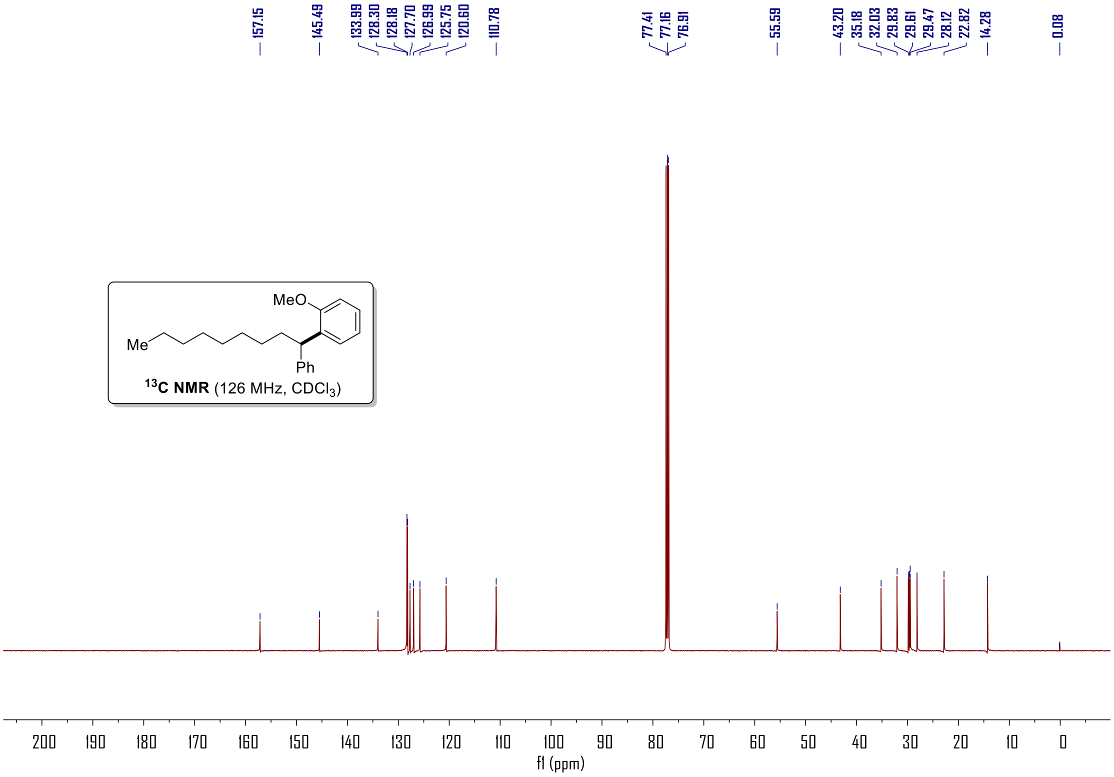

1-Chloro-3-(1-phenylnonyl)benzene (3ae)

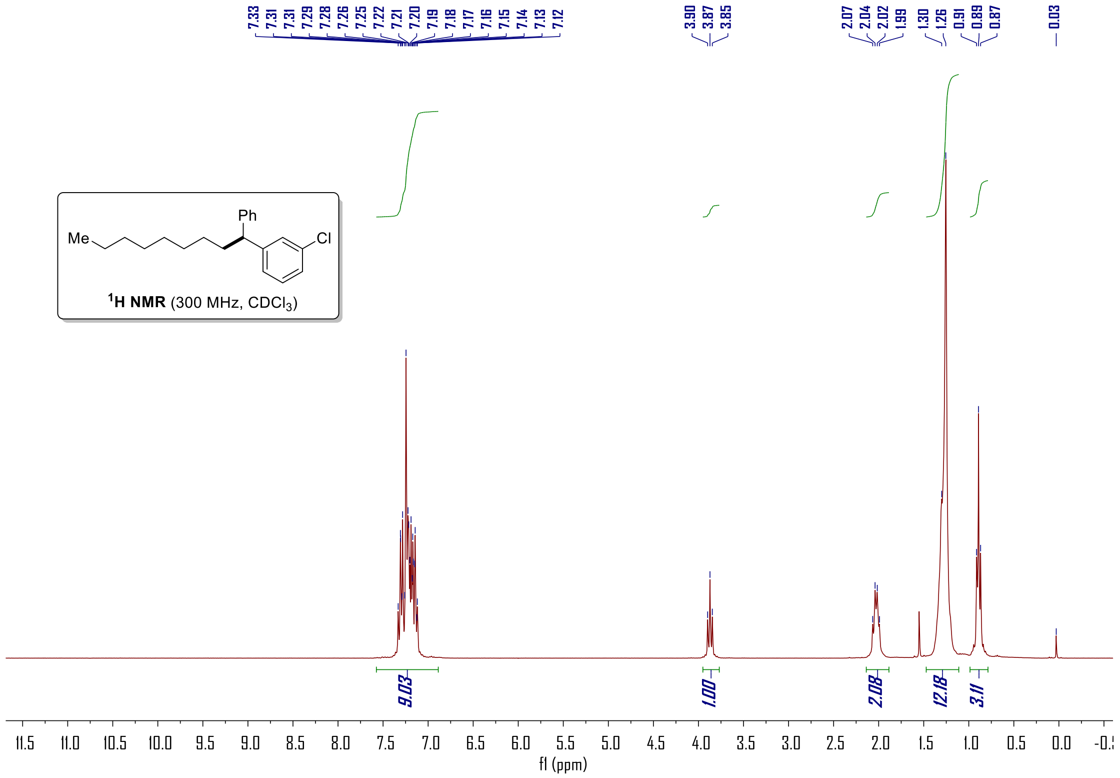

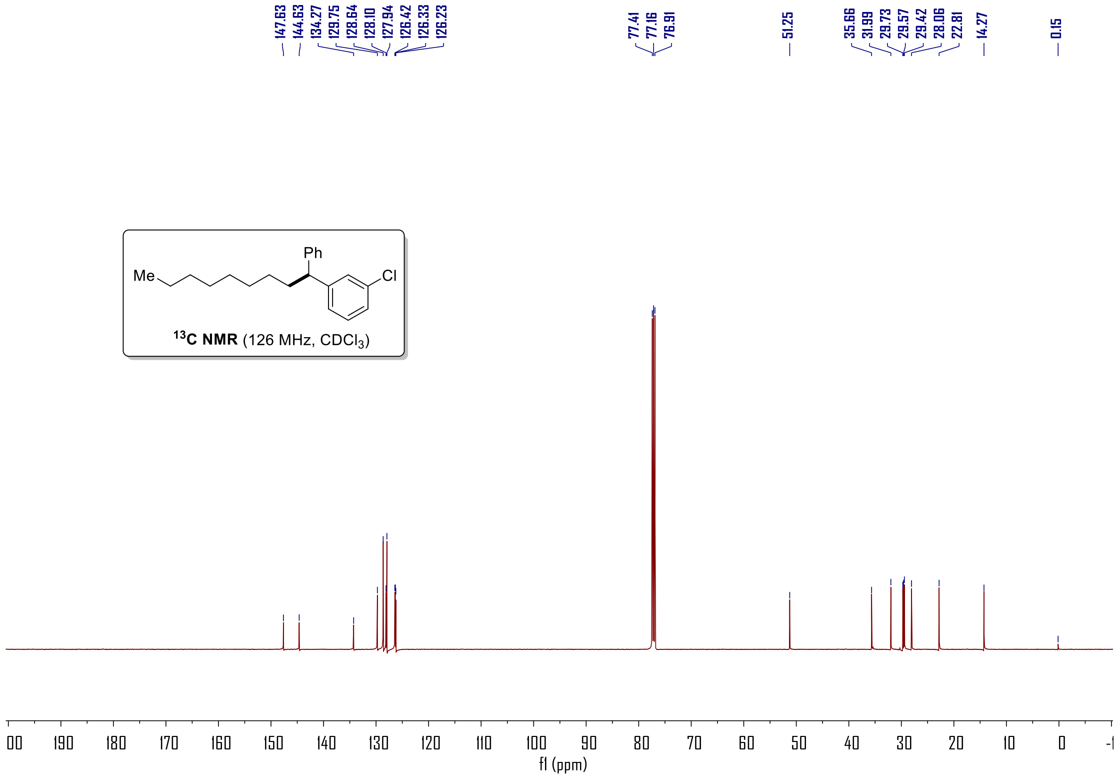

1-Chloro-4-(1-phenylnonyl)benzene (3af)

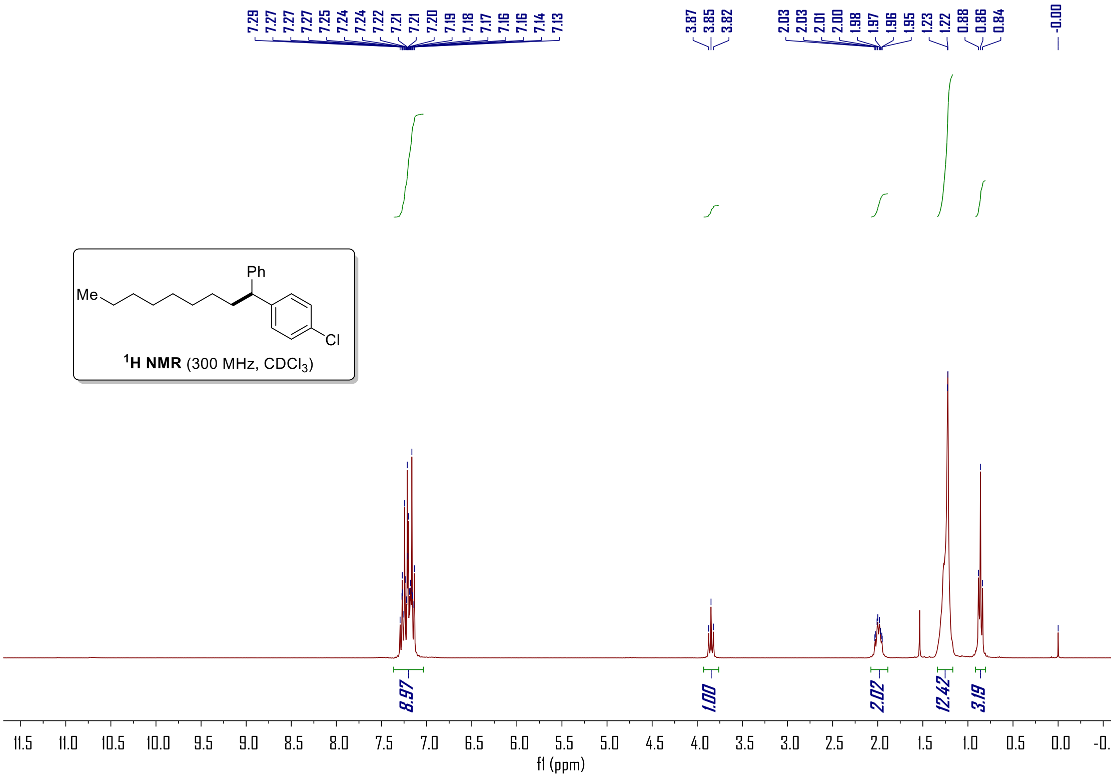

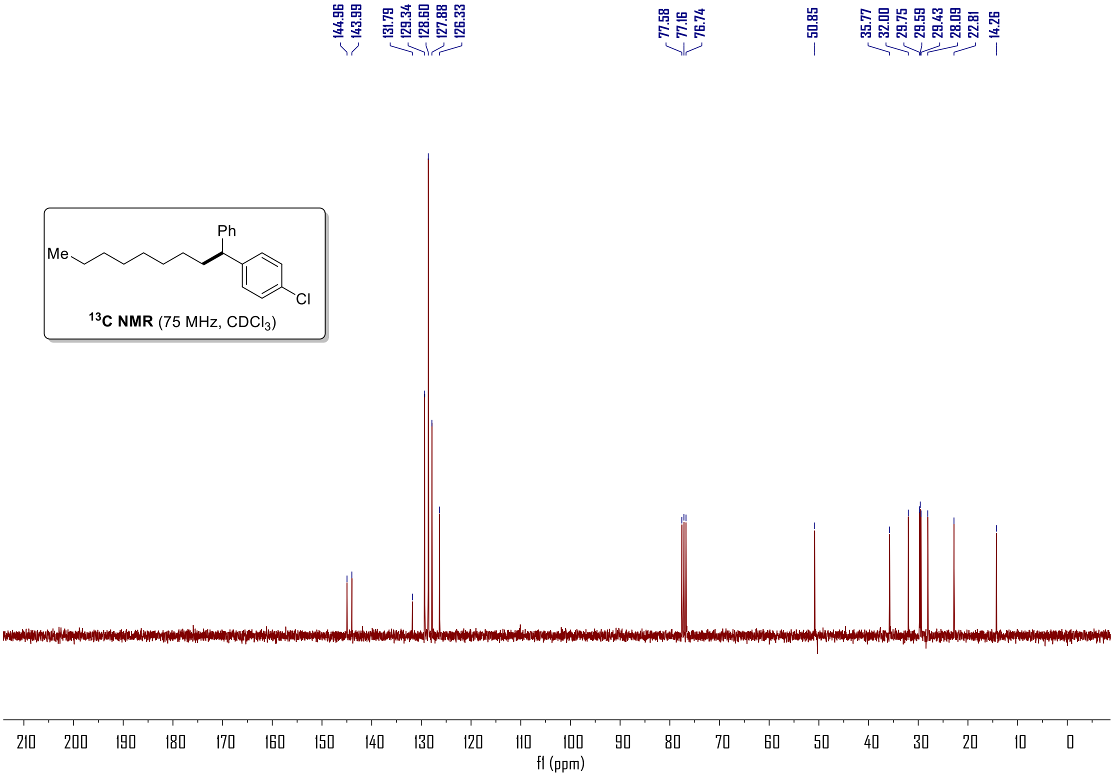

4,4'-(Nonane-1,1-diyl)bis(methoxybenzene) (3ag)

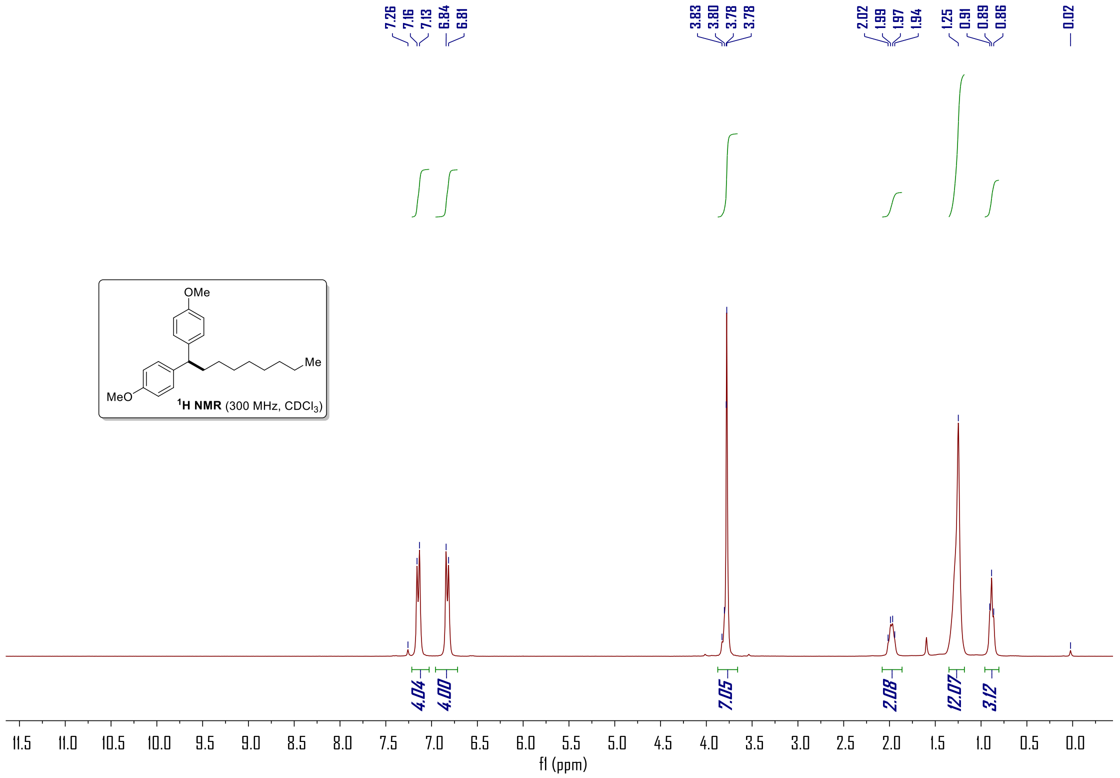

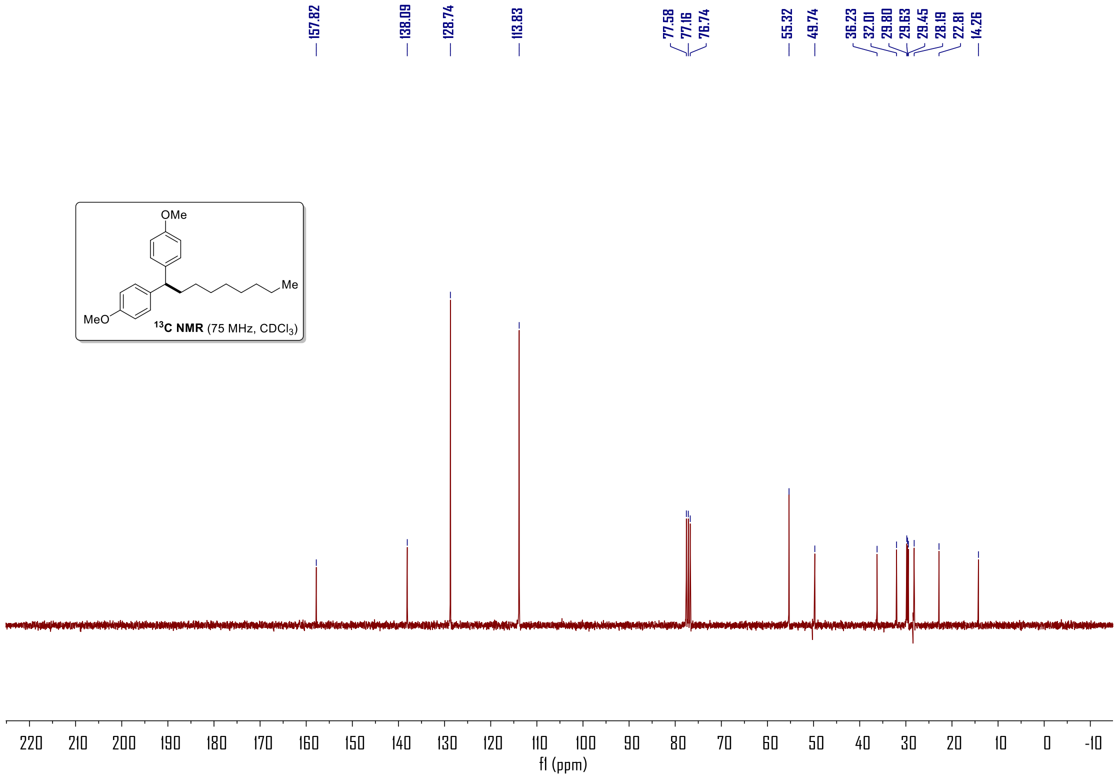

4,4'-(Nonane-1,1-diyl)bis(methylbenzene) (3ah)

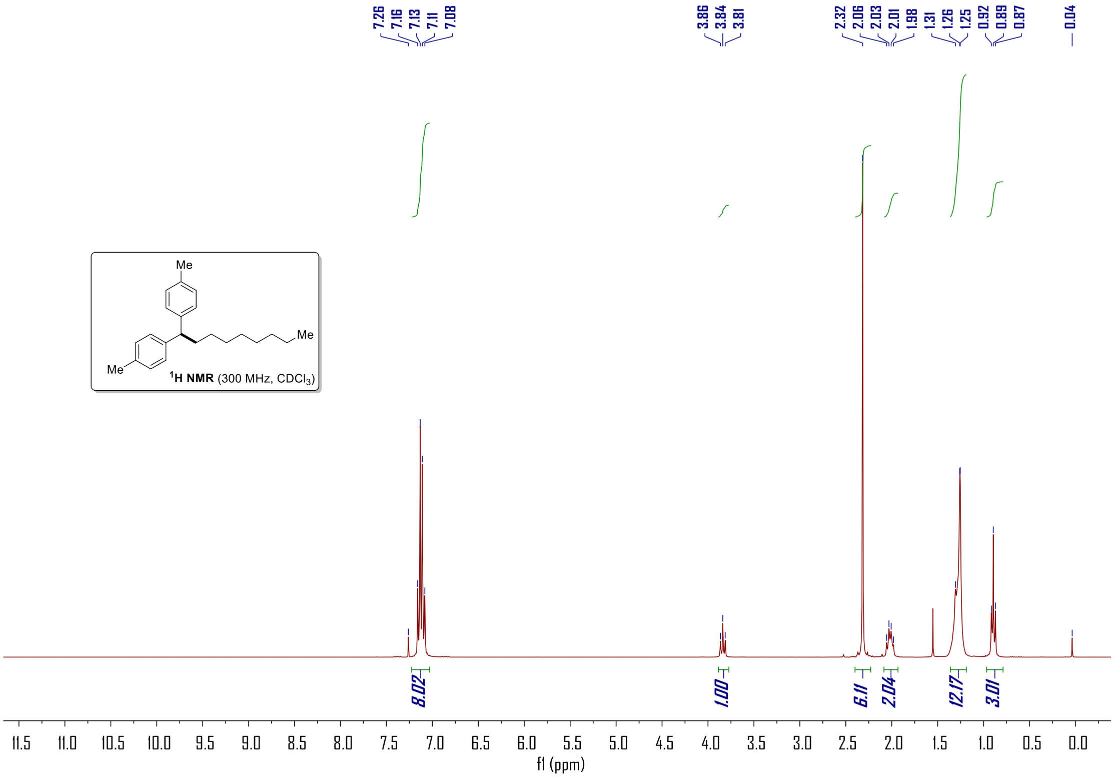

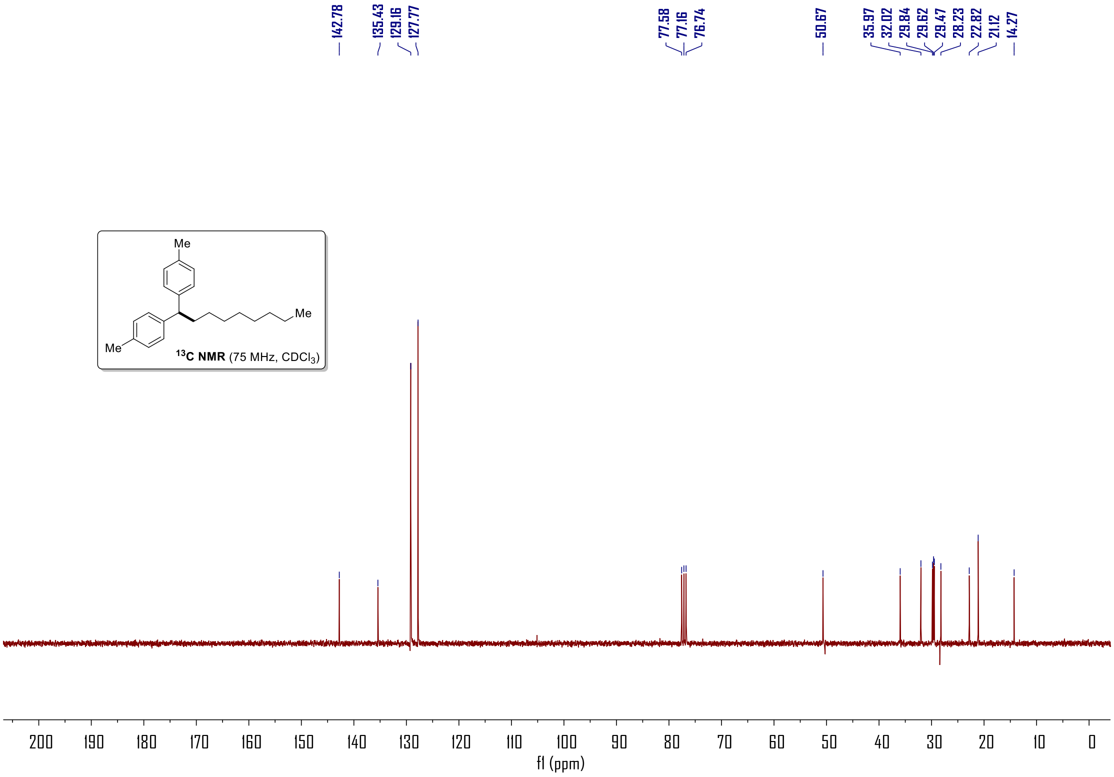

4-(1-(*p*-Tolyl)nonyl)-1,1'-biphenyl (3ai)

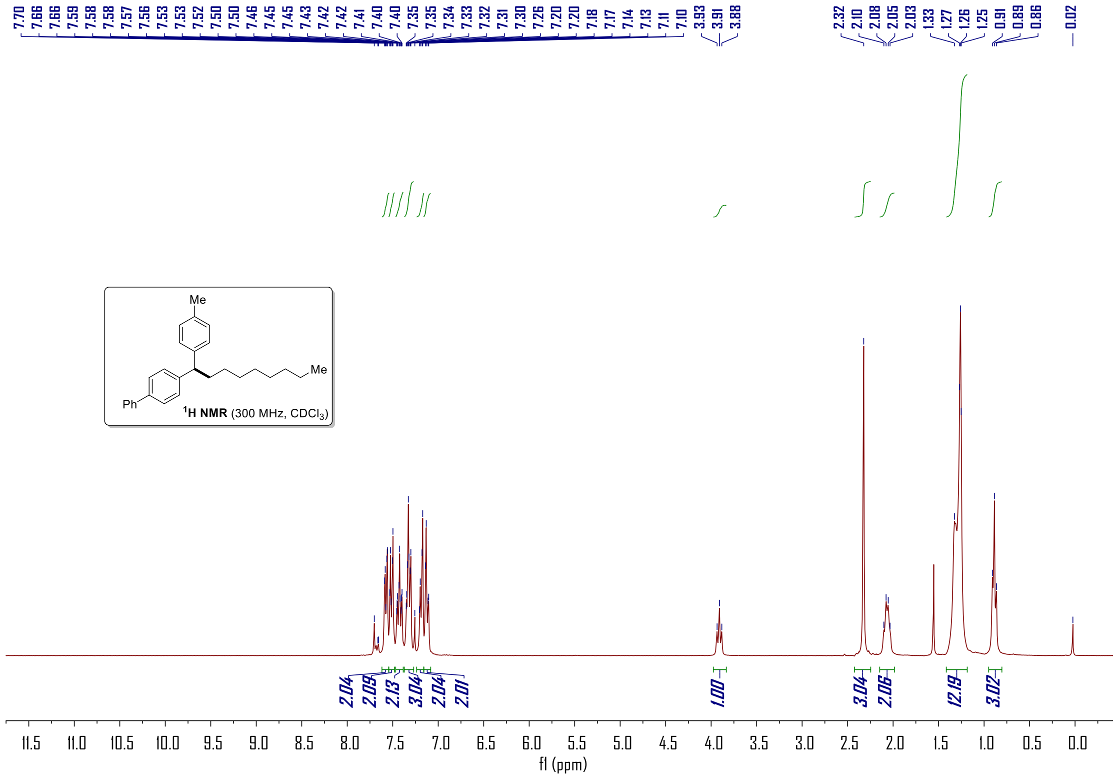

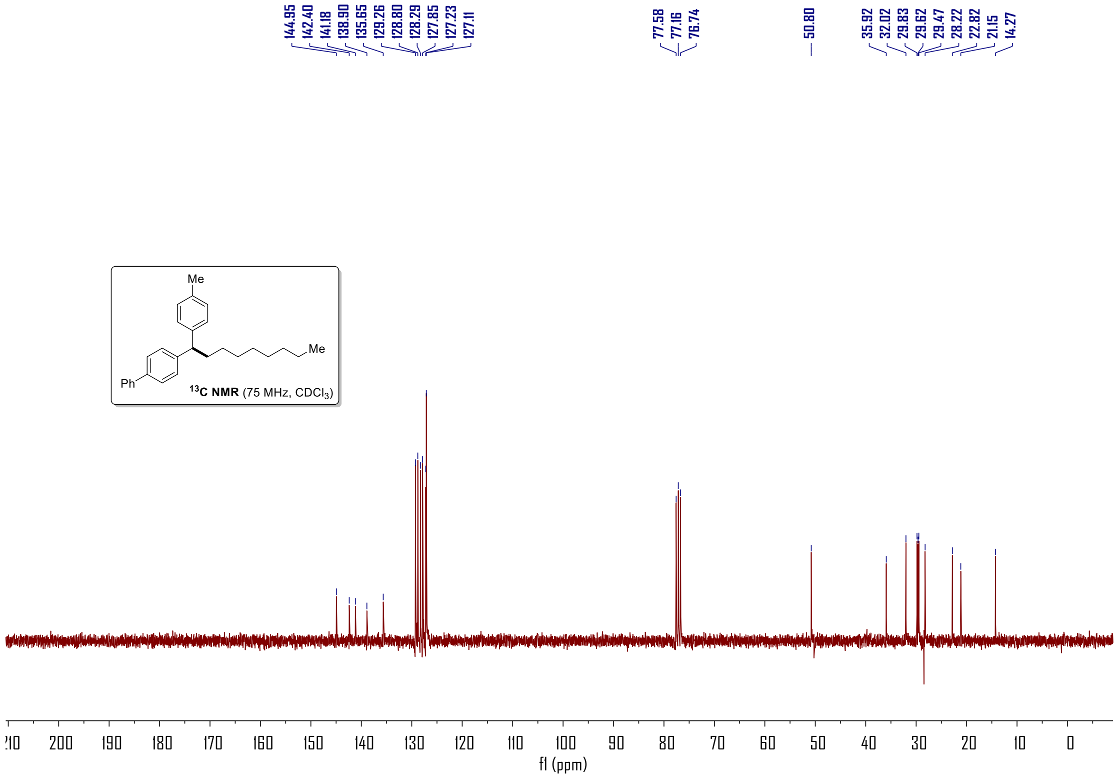

9-Octyl-9*H*-xanthene (3aj)

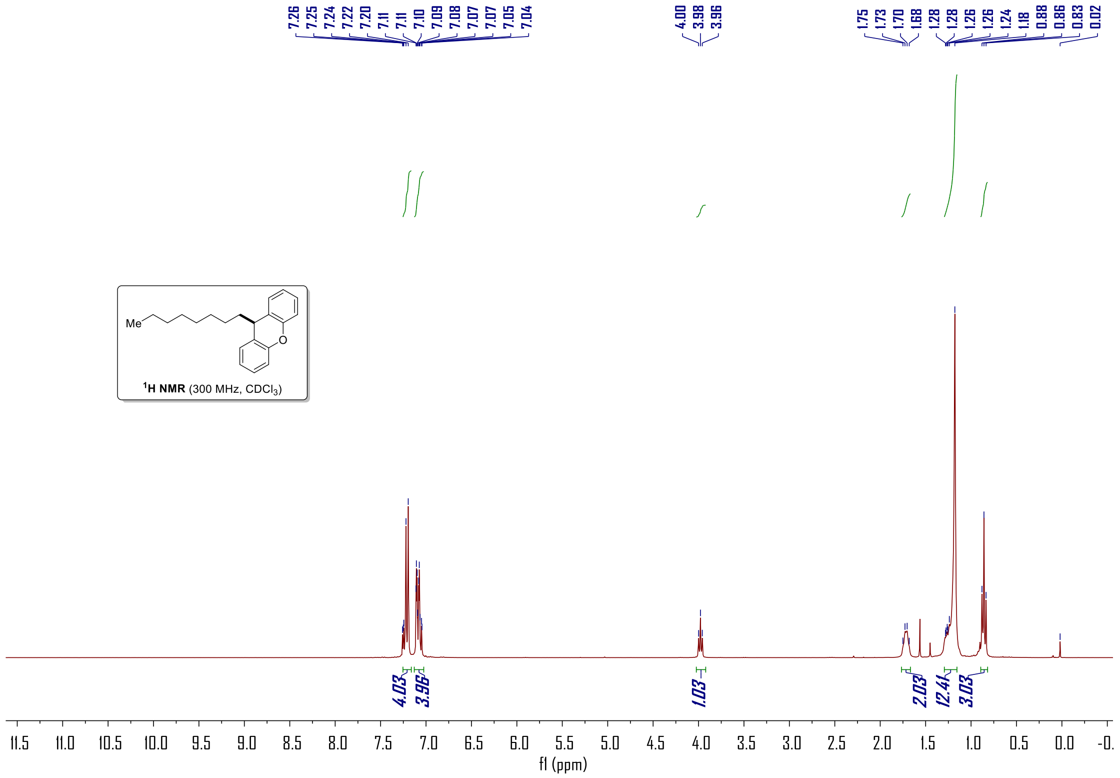

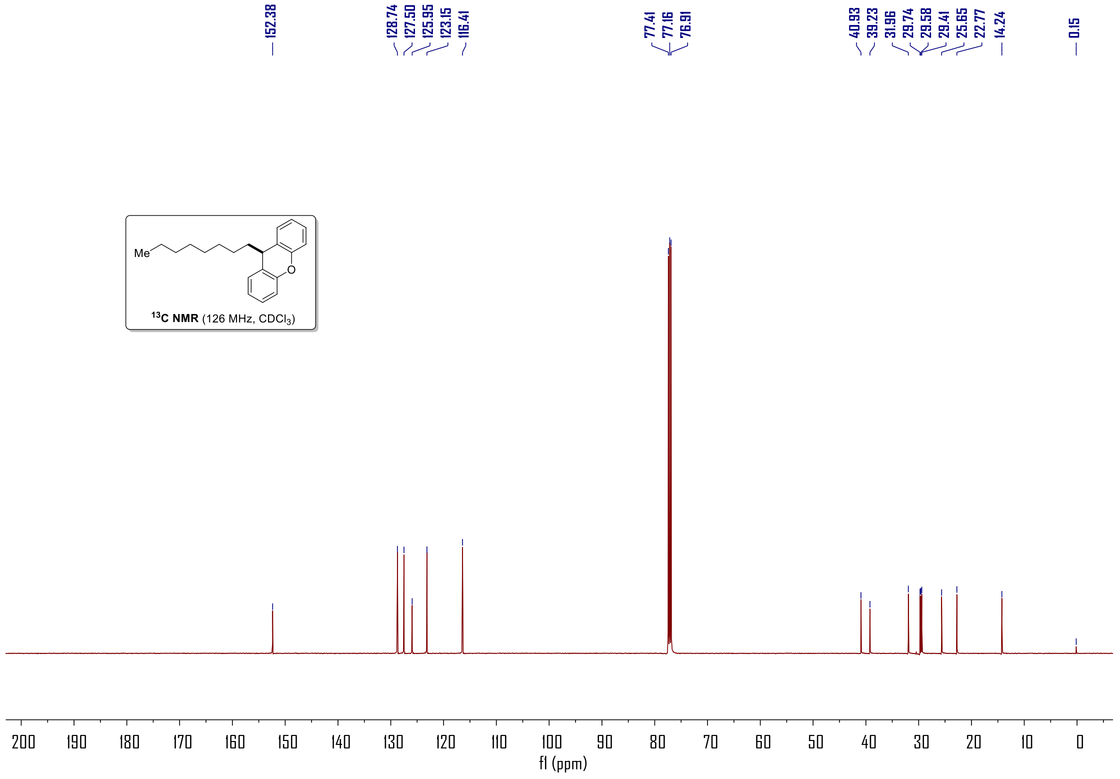

9-Octyl-9,10-dihydroanthracene (3ak)

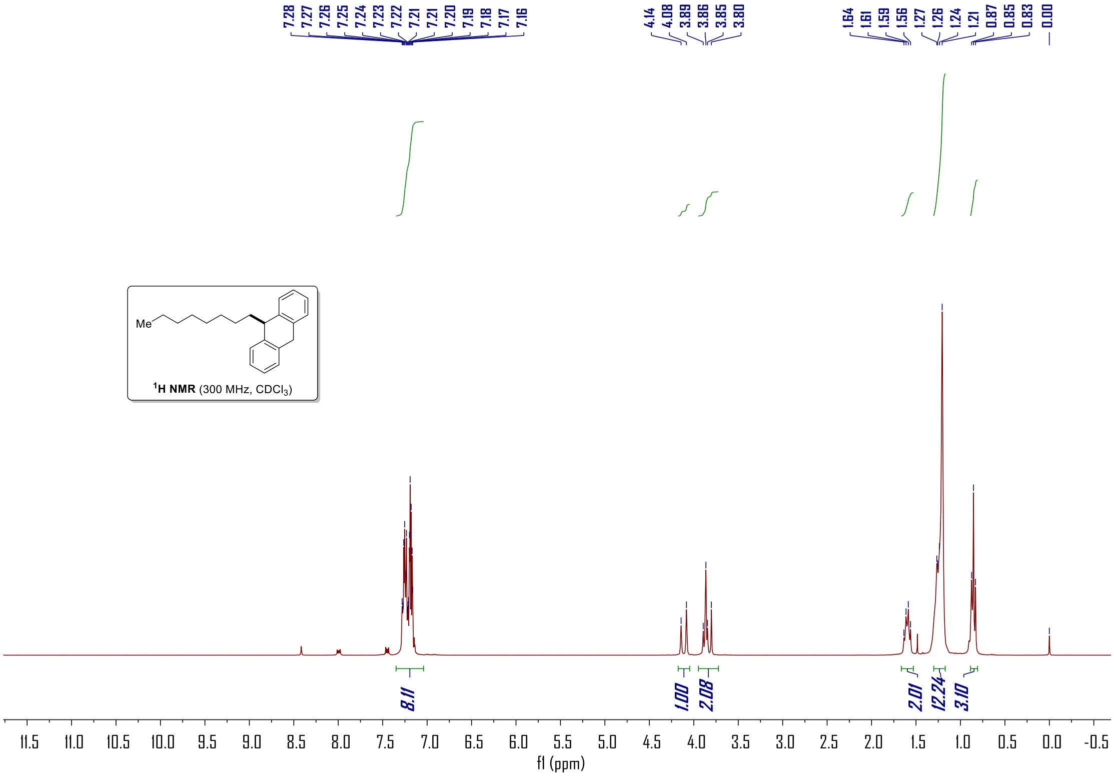

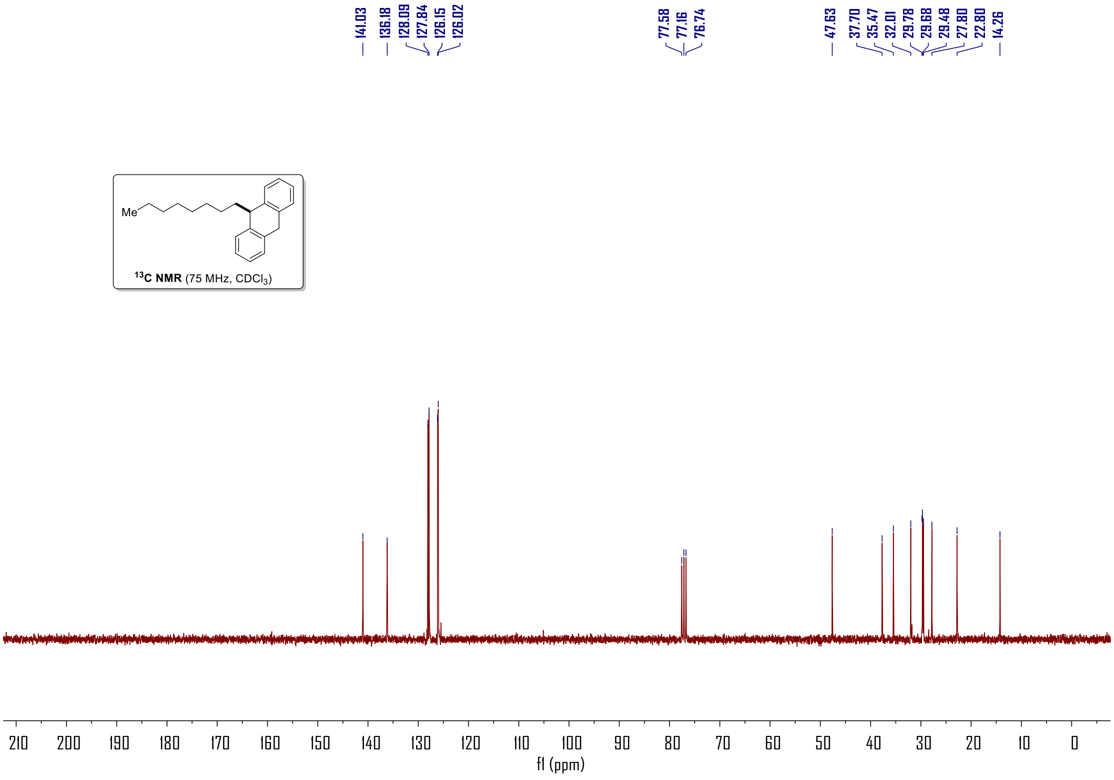

Decane-2,2-diyldibenzene (3al)

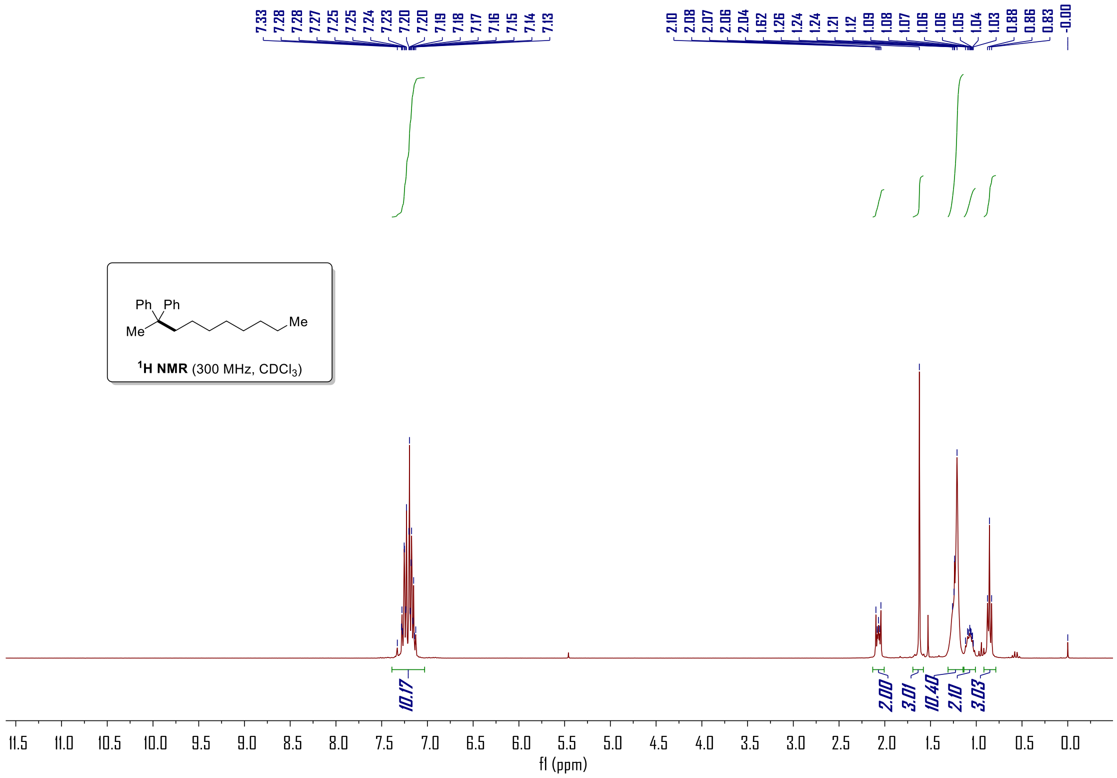

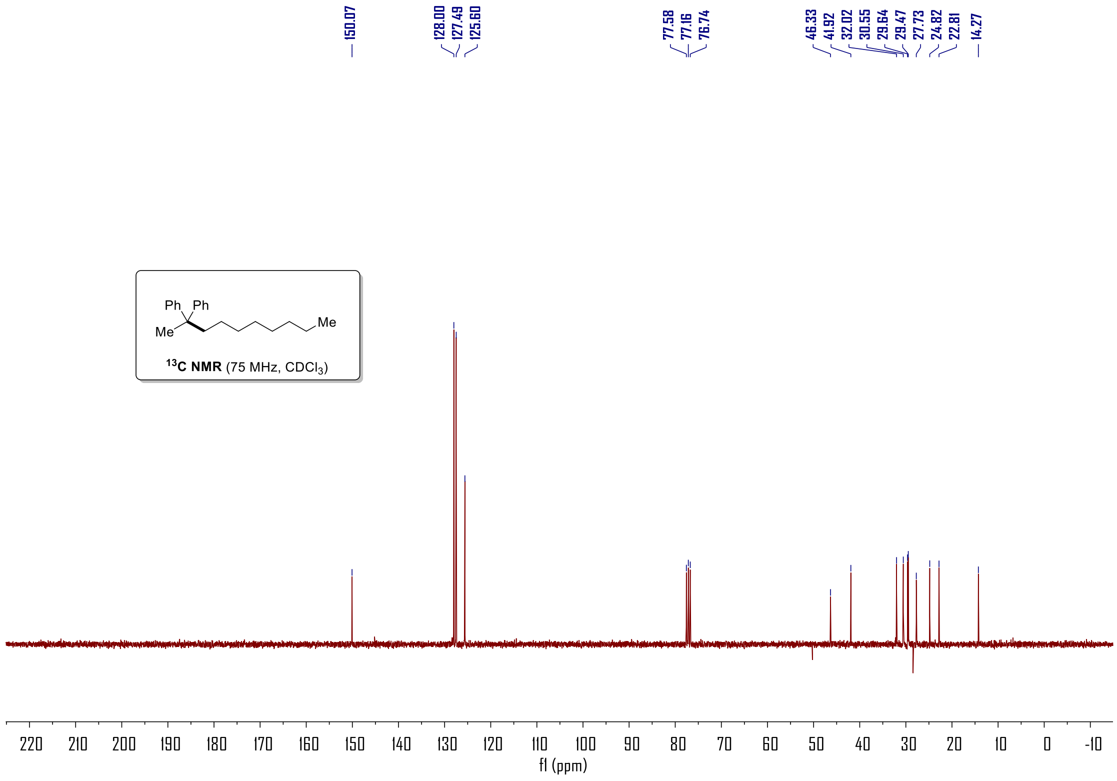

Undecane-3,3-diyldibenzene (3am)

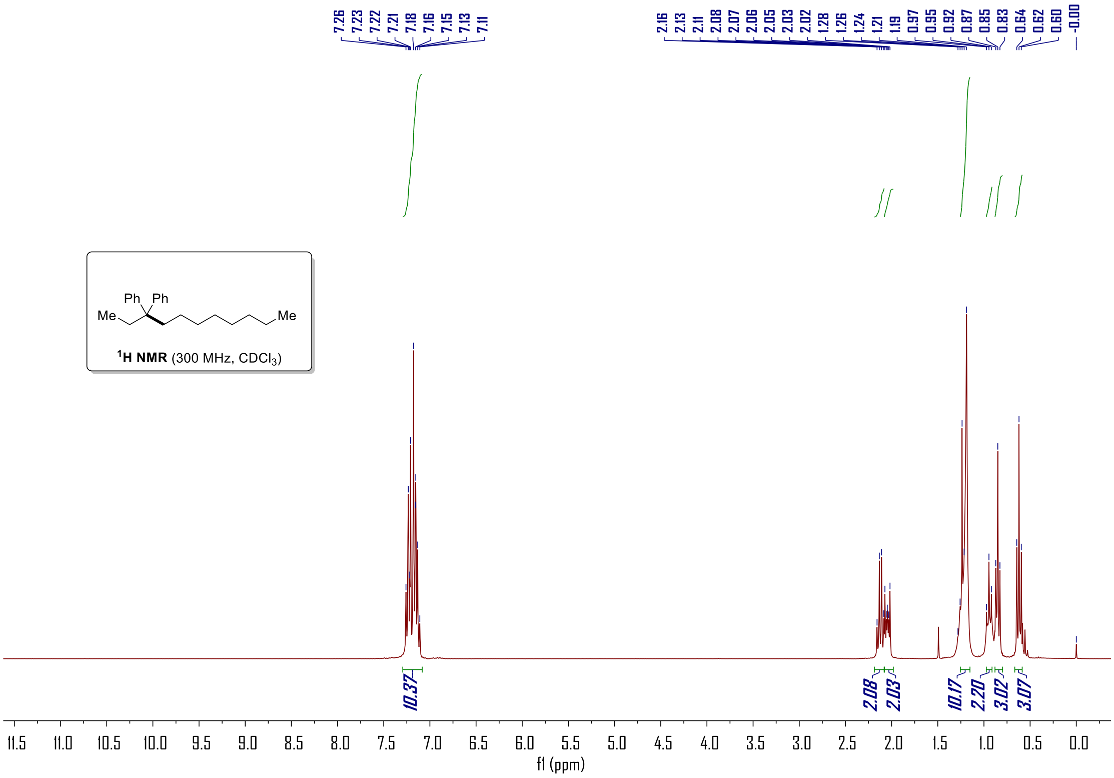

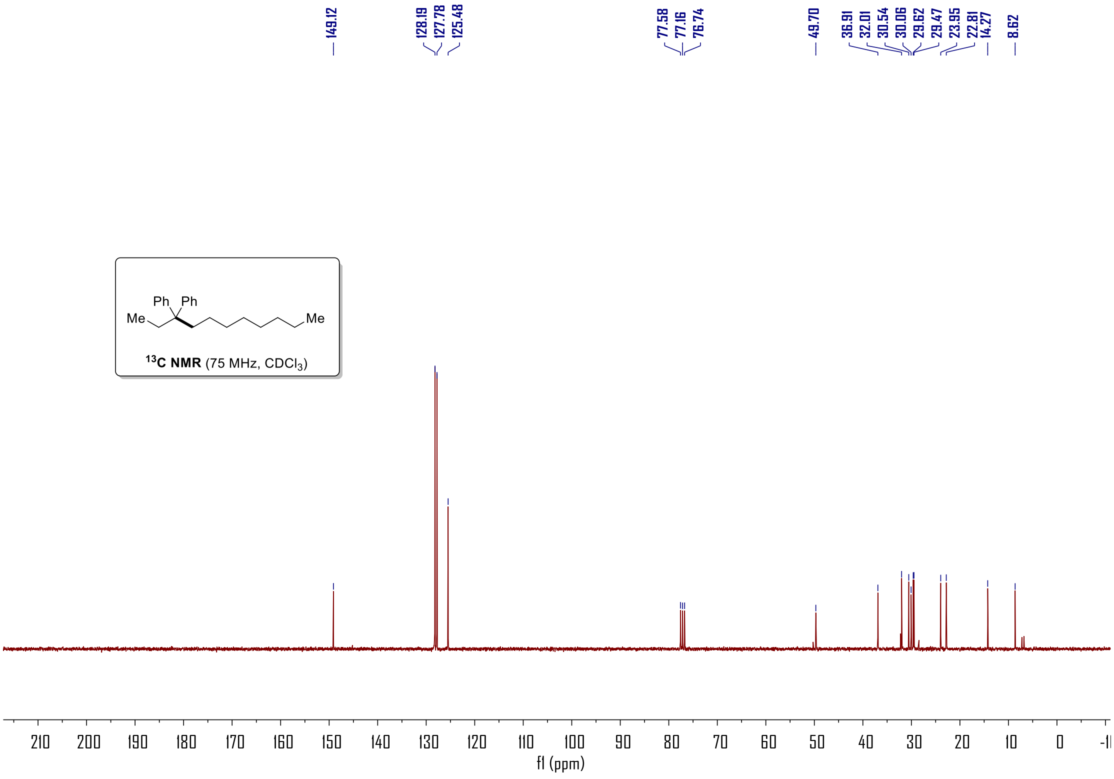

(2-Methyldecan-2-yl)benzene (3an)

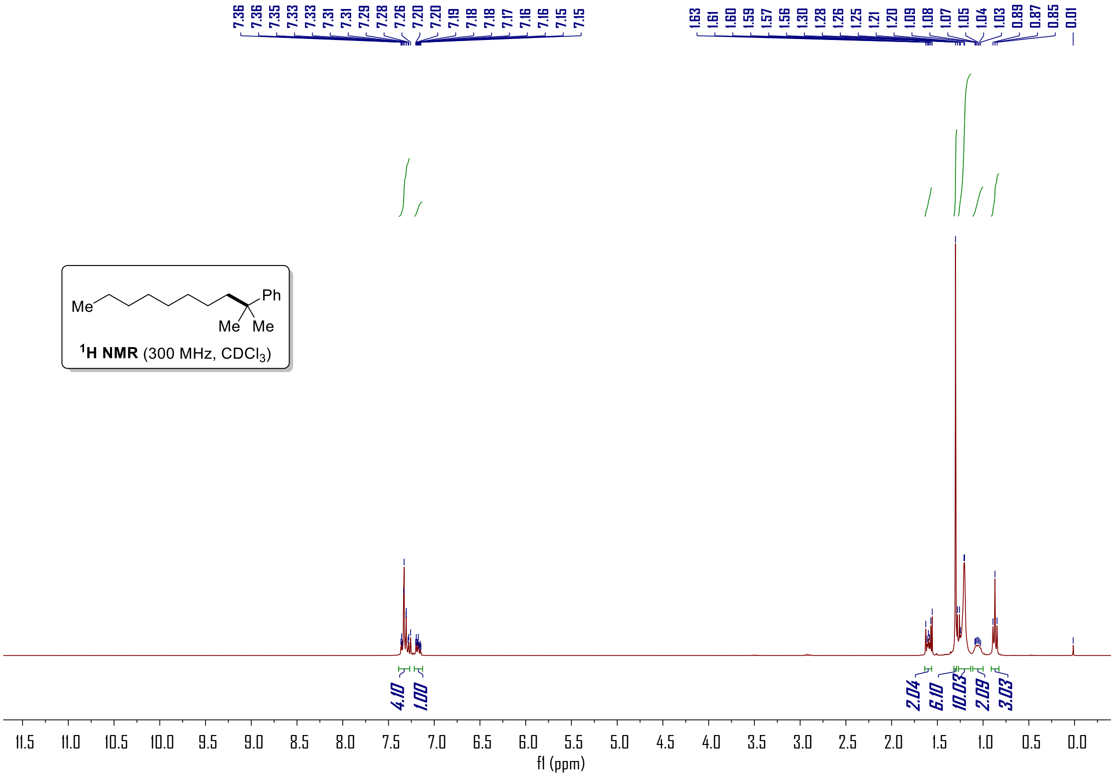

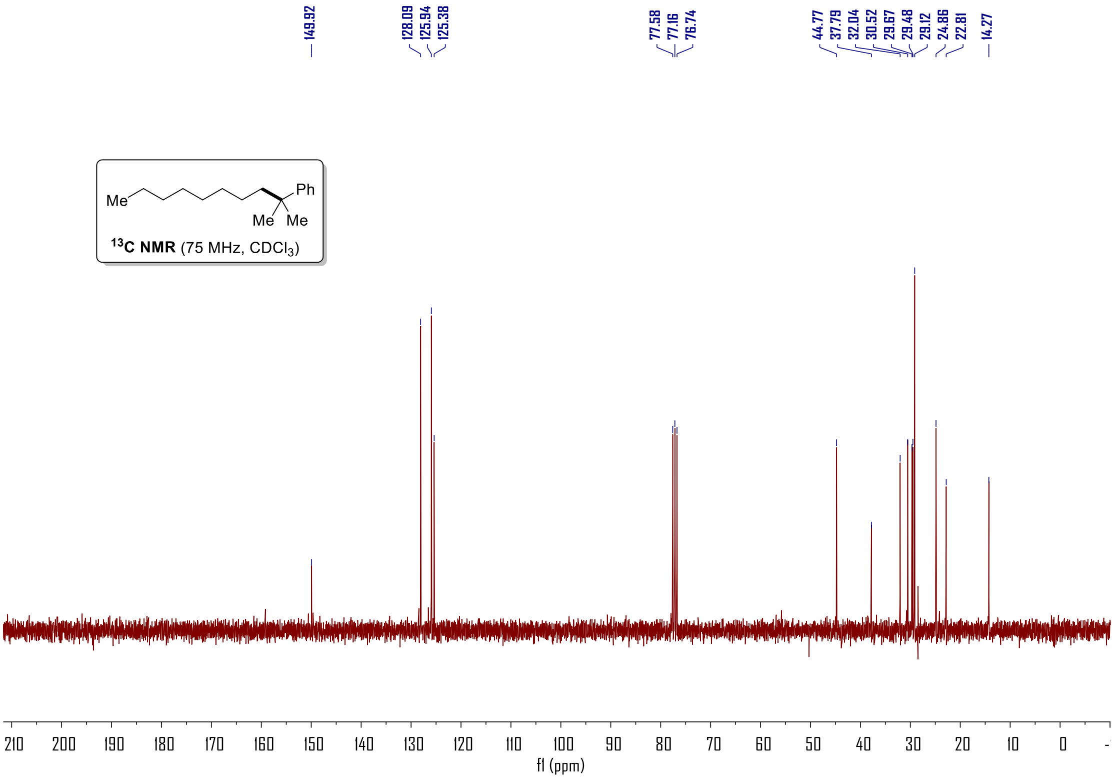

1-(Decan-2-yl)-3-methoxybenzene (3ao)

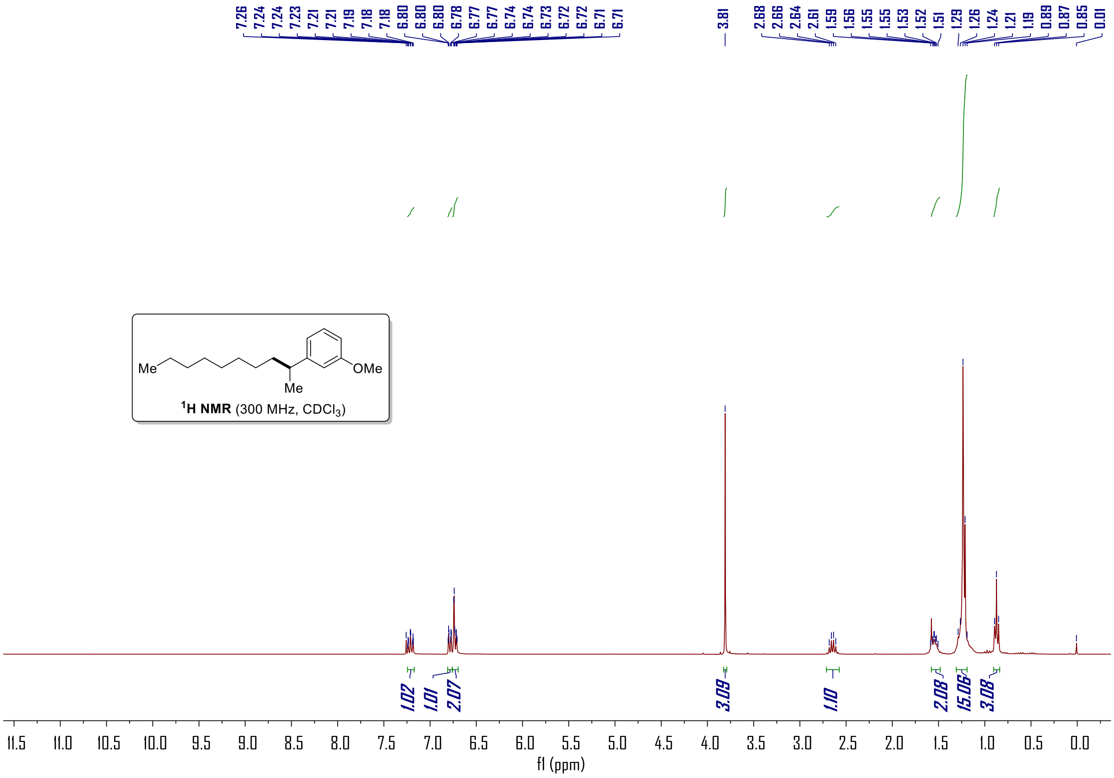

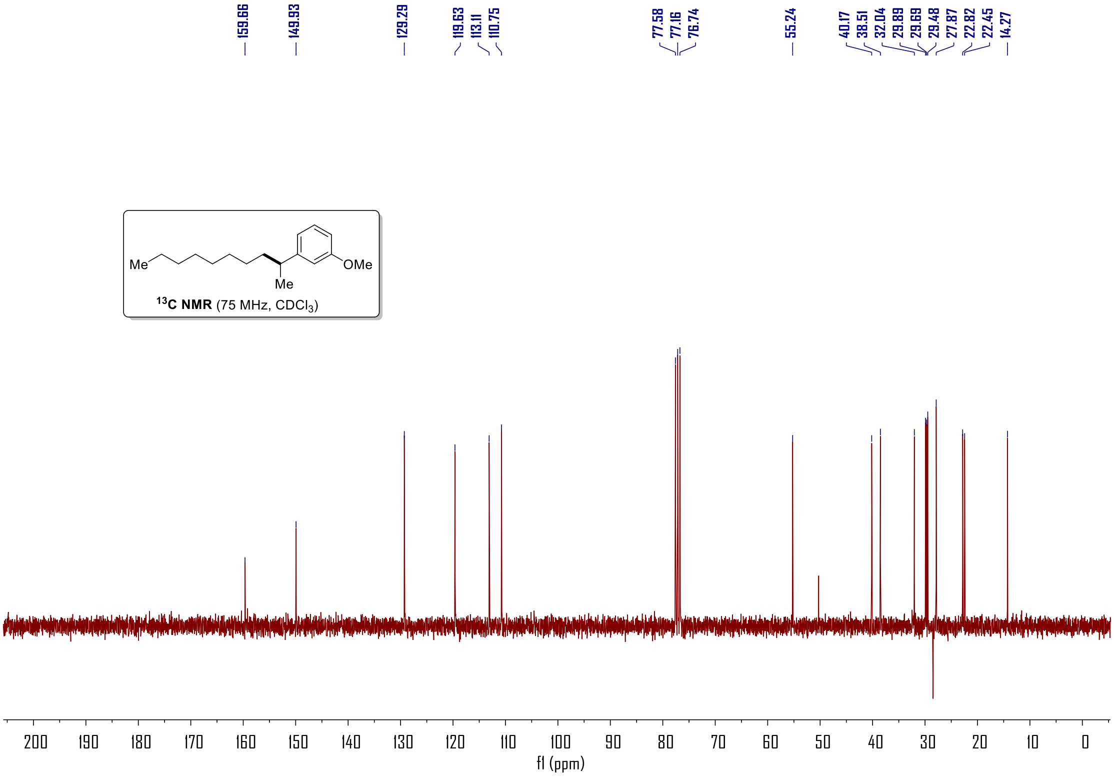

Dodecan-4-ylbenzene (3ap)

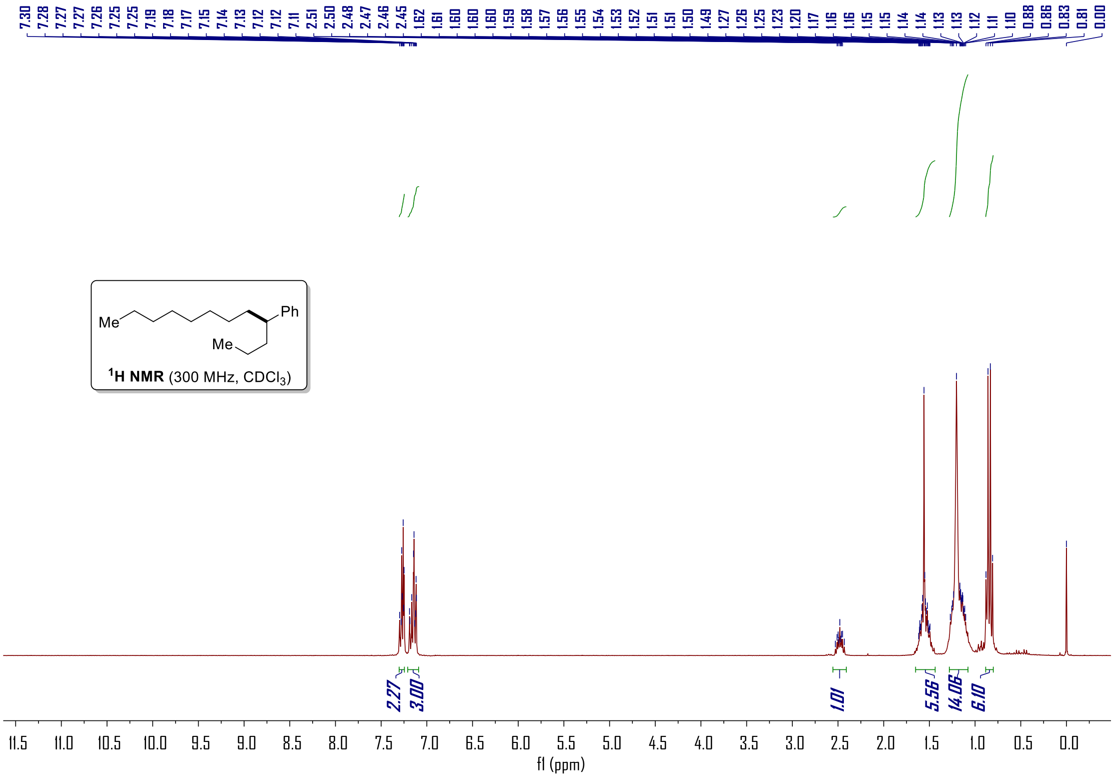

Dodec-1-en-4-ylbenzene (3aq)

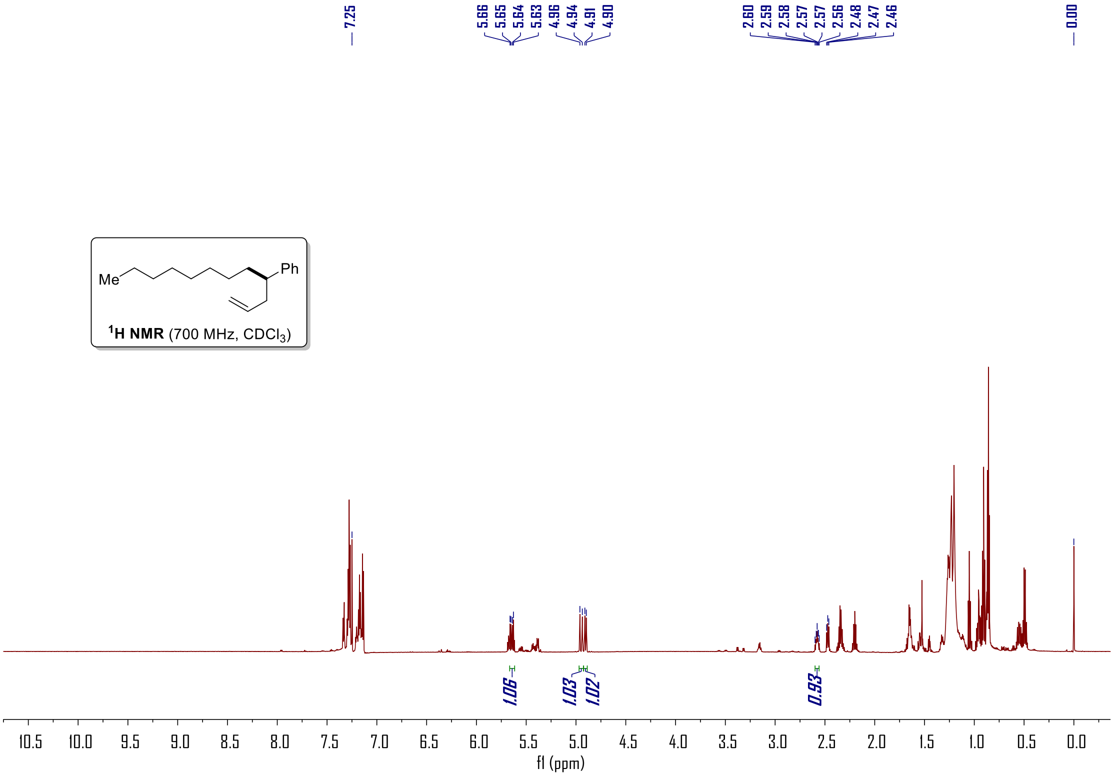

(2-(2-Phenylcyclopropyl)ethane-1,1-diyl)dibenzene (3sb)

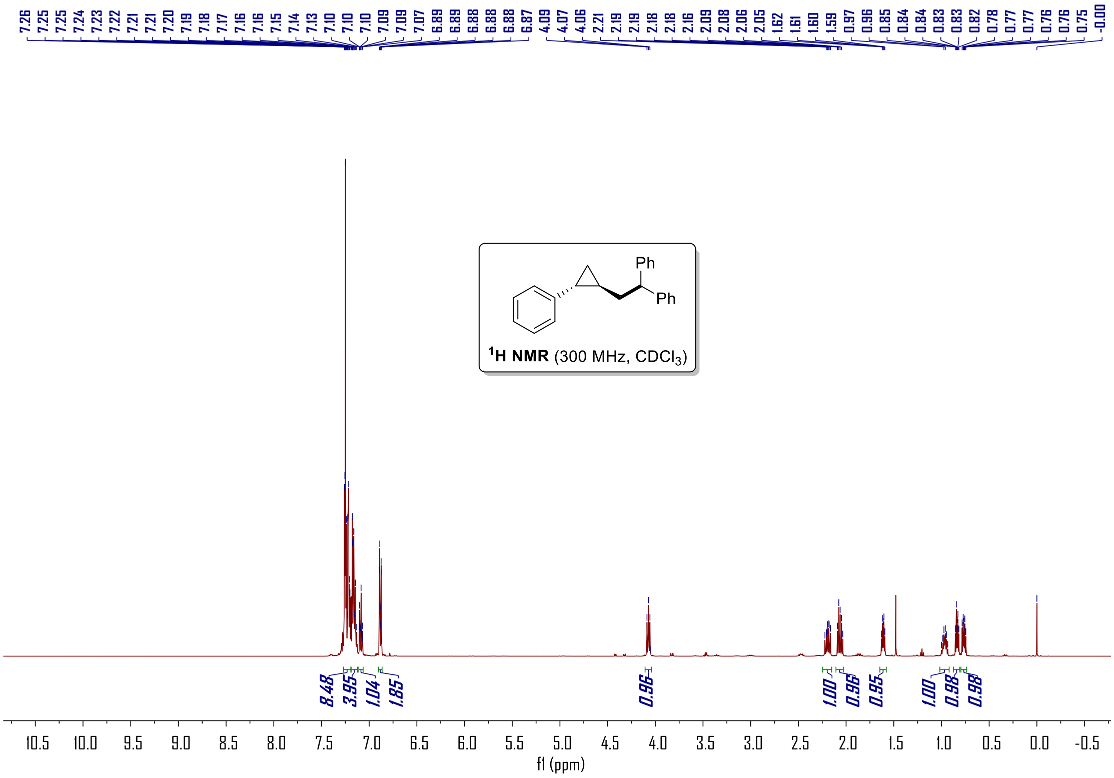

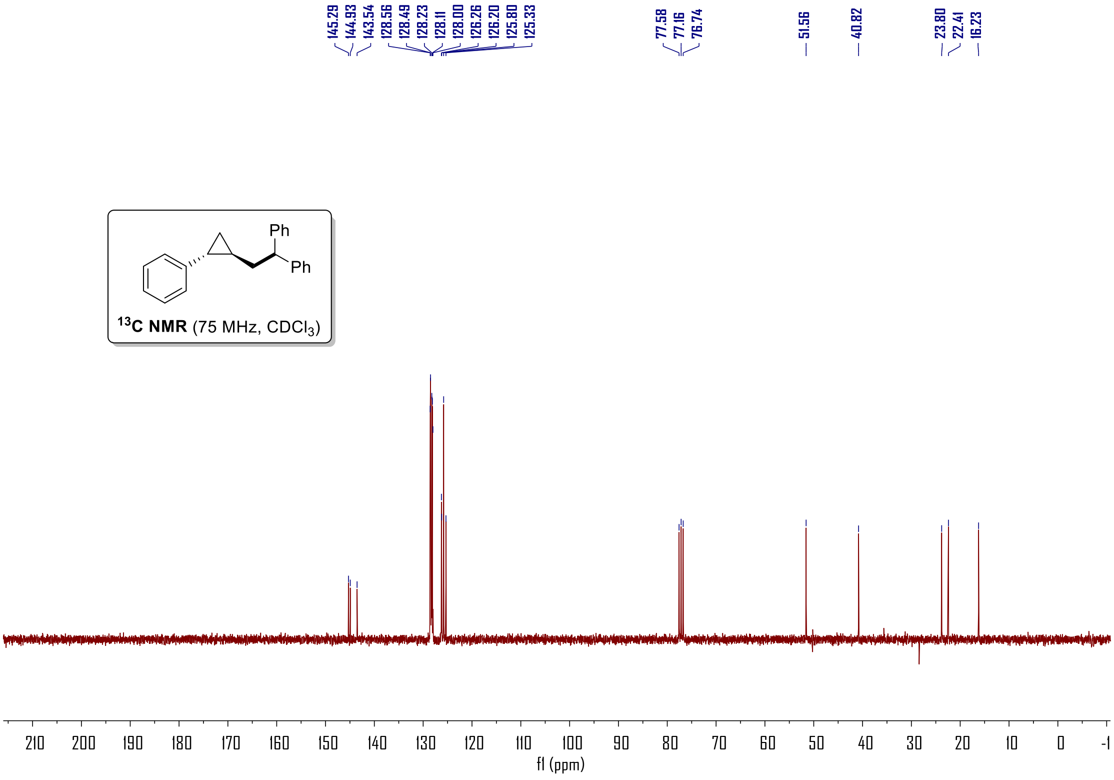

9-((2-Phenylcyclopropyl)methyl)-9*H*-xanthene (3sk)

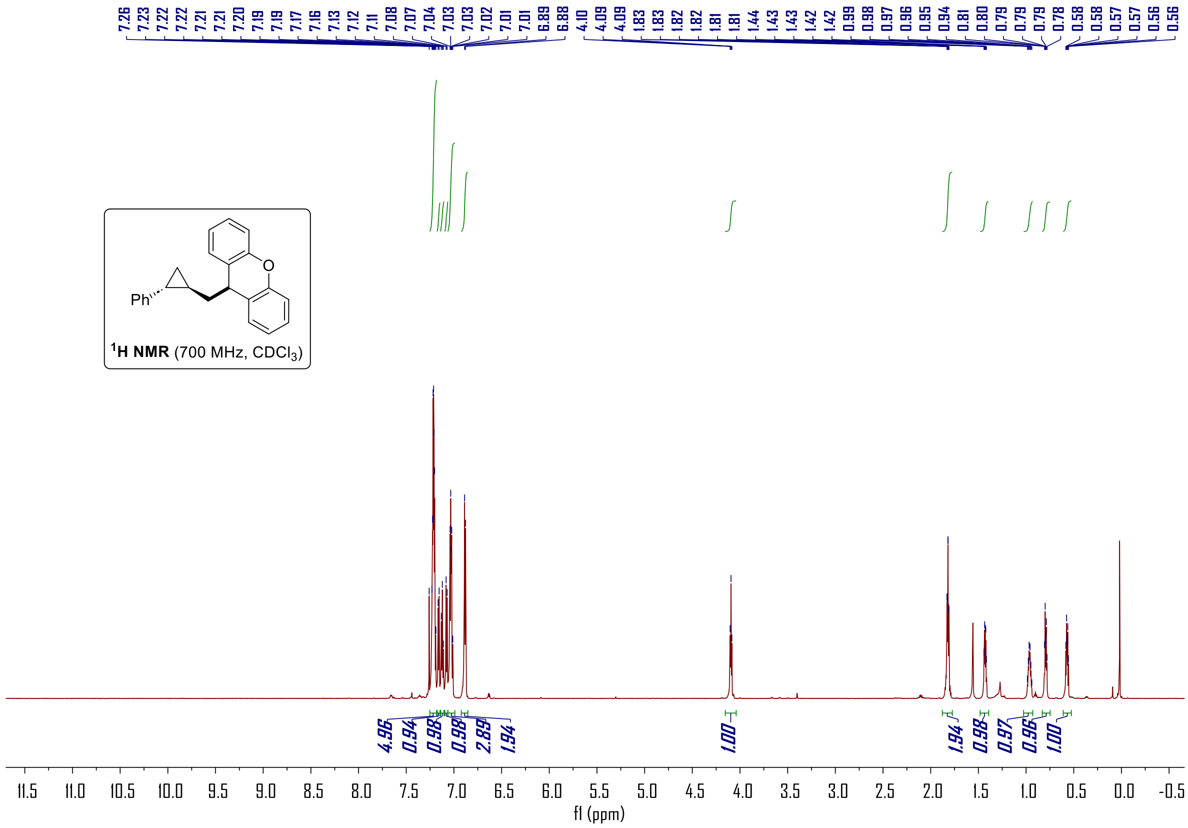

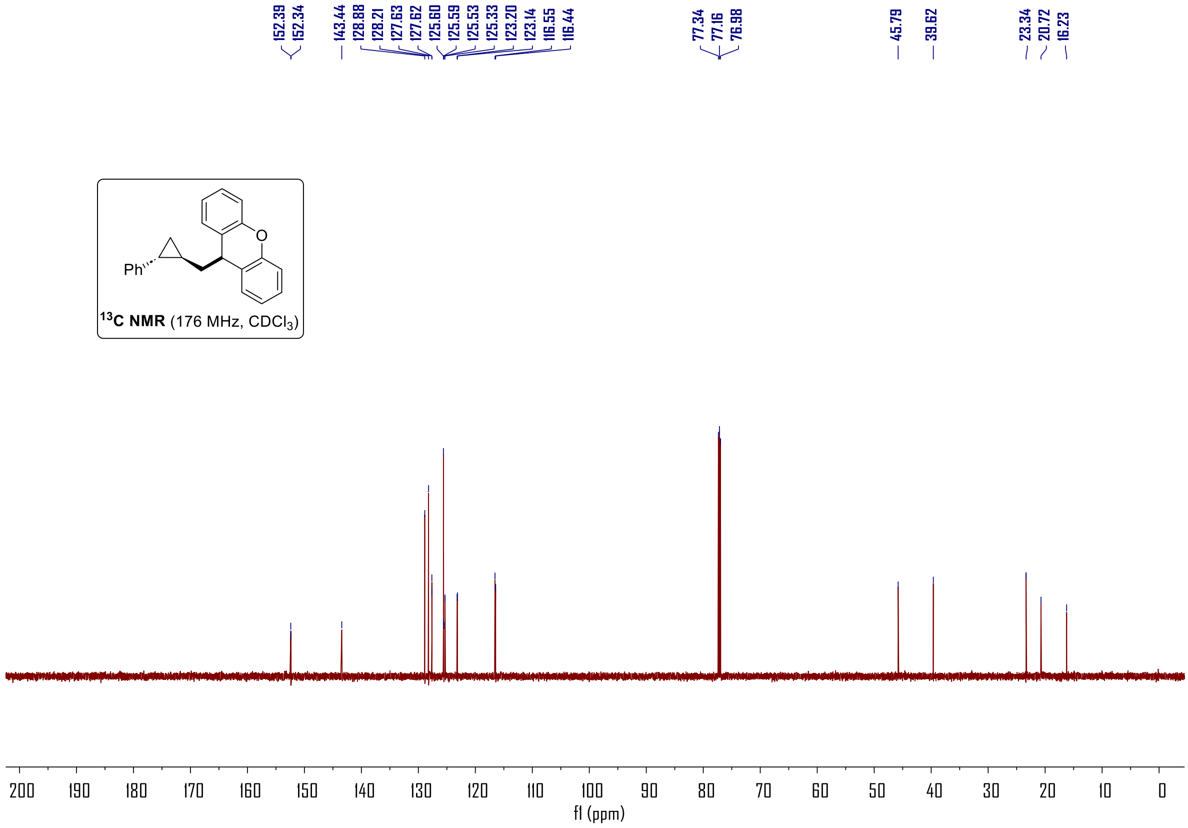

Supplement: SC-015-D4SC04357J-s001 [file SC-015-D4SC04357J-s001.pdf]
